# Investigations of fine-scale phylogeography in *Tigriopus californicus *reveal historical patterns of population divergence

**DOI:** 10.1186/1471-2148-9-139

**Published:** 2009-06-23

**Authors:** Christopher S Willett, Jason T Ladner

**Affiliations:** 1 Department of Biology, University of North Carolina, Chapel Hill, Chapel Hill NC 27599-3280, USA; 2 Current address: Hopkins Marine Station of Stanford University, Department of Biological Sciences, Oceanview Blvd, Pacific Grove 93950, USA

## Abstract

**Background:**

The intertidal copepod *Tigriopus californicus *is a model for studying the process of genetic divergence in allopatry and for probing the nature of genetic changes that lead to reproductive isolation. Although previous studies have revealed a pattern of remarkably high levels of genetic divergence between the populations of this species at several spatial scales, it is not clear what types of historical processes are responsible. Particularly lacking are data that can yield insights into population history from the finest scales of geographic resolution.

**Results:**

Sequence variation in both cytochrome b (*CYTB*, mtDNA) and the rieske iron-sulfur protein (*RISP*, nuclear) are examined at a fine scale within four different regions for populations of *T. californicus*. High levels of genetic divergence are seen for both genes at the broader scale, and genetic subdivision is apparent at nearly all scales in these populations for these two genes. Patterns of polymorphism and divergence in both *CYTB *and *RISP *suggest that selection may be leading to non-neutral evolution of these genes in several cases but a pervasive pattern of neither selection nor coadaptation is seen for these markers.

**Conclusion:**

The use of sequence data at a fine-scale of resolution in this species has provided novel insights into the processes that have resulted in the accumulation of genetic divergence among populations. This divergence is likely to result from an interplay between a limited dispersal ability for this copepod and the temporal instability of copepod habitat. Both shorter-term processes such as the extinction/recolonization dynamics of copepod pools and longer-term processes such as geological uplift of coastline and sea level changes appear to have impacted the patterns of differentiation. Some patterns of sequence variation are consistent with selection acting upon the loci used in this study; however, it appears that most phylogeographic patterns are the result of history and not selection on these genes in this species.

## Background

Genetically divergent populations of the intertidal copepod *Tigriopus californicus *have become an important model system for studying how populations diverge in allopatry and how this genetic divergence can lead to the accumulation of reproductive isolation between populations [1-7]. Important questions still remain to be answered in this species concerning the nature of the historical processes that have led to the dramatic levels of genetic divergence between populations. Studies of variation in DNA sequences can be a powerful tool for intra-specific phylogeography and can help reveal historical patterns of population differentiation but have not yet been applied to the finest scales of resolution in this species, the scale over which gene flow is likely to be occurring [2,3,5,8,9]. An examination of this type of data may help reveal the nature of the interplay between gene flow, geography, geological processes, and extinction that contribute to genetic divergence in this system.

Despite the apparent ephemeral nature of their habitat (high intertidal rock pools that individually dry up on a regular basis), data from *T. californicus *populations show convincing evidence of long-term persistence within an outcrop and limited gene flow between rocky outcrops that results in the accumulation of substantial genetic differentiation between these populations [2]. Over the range of this copepod species from southern Alaska to central Baja California, Mexico, nucleotide sequences can show high levels of genetic divergence between regions on the scale of 100 km apart or less, which in mtDNA-encoded genes can exceed twenty percent [3,5,10,11]. Studies of allozyme variation [12,13] and transplant experiments [9] reveal that between rocky outcrops, within a region, there is also little gene flow. In fact, longer-term monitoring of differences in allozyme frequencies between outcrops (some as close as 500 m apart) reveal that differences remained stable for at least 18 years, potentially more than 100 copepod generations [2]. However, over time local population extinctions are likely, due to the ephemeral nature of copepod habitat [14,15] and long distance gene flow must occur occasionally as evidenced by the recolonization of previously glaciated regions [5]. Most of this previous work primarily utilized allozyme markers at the finest scales of geographic resolution with the result that patterns of DNA sequence variation have not yet been studied at the geographic scale necessary to yield insights into the longer-term historical processes of extinction and recolonization.

The high levels of mtDNA divergence between populations of *T. californicus *reflect large numbers of differences in both synonymous and non-synonymous (or amino acid changing) sites. For example in the cytochrome b gene (*CYTB*, 1128 bp) between two California populations (Santa Cruz, SCN and Abalone Cove, AB) there are 197 synonymous differences and 28 amino acid changes [10]. These high levels of divergence are likely to reflect both long periods of isolation between populations and high mutation rates. Willett and Burton [10] showed that mtDNA is evolving at a much more rapid rate than nuclear genes in this species, between 26 and 38-fold higher at synonymous sites. Although higher rates of mtDNA than nuclear DNA evolution are found in other taxa, especially vertebrates [16], the ratio of mtDNA to nuclear DNA rates of evolution in *Tigriopus *appears to be high when compared with other invertebrates and arthropods [10,17,18]. There is some evidence for positive selection acting upon a limited number of sites in one mtDNA gene in *T. californicus*, cytochrome oxidase subunit 2, COII [11], but in general the rapid accumulation of synonymous differences between these populations weakens the power of these rate-based tests of selection to detect selection on amino acid substitutions.

Despite the limited evidence to date for selection on mtDNA in *T. californicus *from patterns of sequence divergence there are reasons to believe that evolution of mtDNA in this species has resulted in the functional divergence of mtDNA-encoded proteins between populations. A number of experiments in *T. californicus *examining hybrid fitness and enzymatic rates have suggested that a portion of the genetic variation between populations leads to divergence in intergenomic coadaptation [6]. MtDNA-encoded proteins in the electron transport system are part of large, multi-subunit proteins and interact directly with many nuclear-encoded proteins so that intimate co-evolution between these proteins is likely to occur. Studies in *T. californicus *have shown that the interactions between mtDNA-encoded and nuclear-encoded proteins in the electron transport system have resulted in divergence in genomic coadaptation and include examples of the loss of functional coadaptation as measured by examining electron transport system enzyme and gene activities [19-22], impaired mitochondrial function [21], and lowered hybrid copepod fitness [21,23-28]. It is not clear if this coadaptation between mtDNA-encoded and nuclear-encoded proteins of the ETS has lead to consistent selection acting upon mtDNA and if this selection on mtDNA could alter phylogeographic patterns.

In this study we will characterize nucleotide sequence variation in *CYTB *(a mtDNA-encoded protein of complex III of the electron transport system) to study fine-scale phylogeography in *T. californicus *and the potential impacts of non-neutral evolution upon its diversification among these copepod populations. In addition, variation in a nuclear-encoded protein of complex III of the electron transport system, the rieske iron-sulfur protein (RISP) will be studied for use as a nuclear-encoded marker for phylogeographic inferences. A unique aspect of this study is that although there have been a number of studies in *T. californicus *examining fine-scale population structure (within and between adjacent outcrops) using allozymes and broader-scale studies (involving geographically distant populations) using both allozymes and mtDNA/nuclear gene sequences, there are few data available for sequence evolution and differentiation at the finer population scale. These sequence comparisons will have the added advantage of bringing a historical perspective to studies of population subdivision and molecular evolution. We will center these fine-scale studies of genetic differentiation on four different geographic regions (each of which has most likely evolved independently of the other regions for significant periods of time) to determine the extent to which patterns of divergence are repeated across regions.

## Methods

### Population sampling

**Figure 1:**
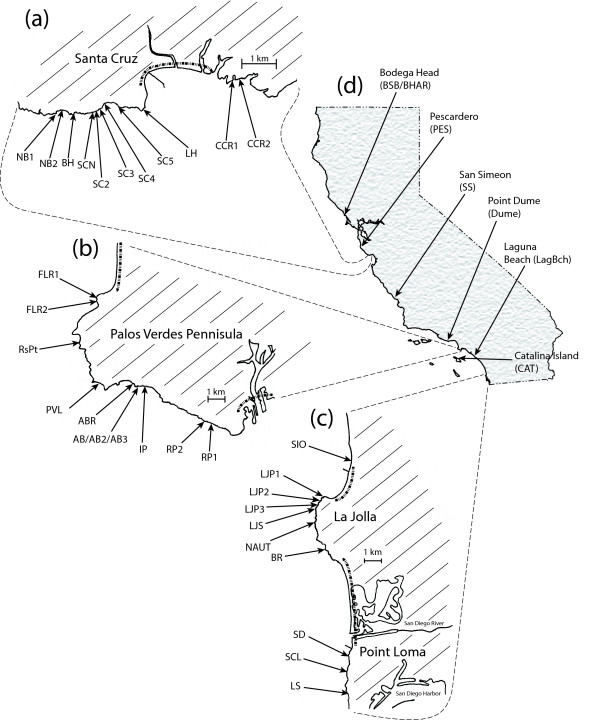
**Map of *T. californicus *sites along the coast in California**. Sites sampled within the (a) Santa Cruz, (b) Palos Verdes, and (c) La Jolla and Point Loma regions are depicted. Dashed lines within each region indicate extensive portions of coastline that consist of low-lying stretches of sandy beach, which are unlikely to have had any copepod habitat in recent time. The location of each of these regions within California is depicted in (d) with the locations of the additional sites. Two other additional sites were included in this study from British Columbia, Canada but are not depicted on this map: Point Atkinson, West Vancouver (BC) and Ucluelet, Vancouver Island (VI).

To investigate genetic divergence in mtDNA and population history among closely spaced *Tigriopus californicus *populations, copepods were collected from high intertidal rock pools spaced along 3–16 km stretches of coastline centered at four rocky outcrops in California that have been used extensively in previous studies of *T. californicus*: San Diego (SD) on Point Loma and La Jolla Point (LJP) both in San Diego County, Abalone Cove (AB) on the Palos Verdes Peninsula in Los Angeles County, and Santa Cruz (SCN) in central California. Sampling at each of these four outcrops (and surrounding regions) was primarily done in one of two months, November 2002 and August 2004. For each sampling site, copepods were combined from several pools from within a single outcrop into a single sample and individuals were randomly selected from this sample for genetic analyses. Previous studies using transplanted individuals have indicated that pools located on the same rocky outcrop will tend to become genetically homogenized over periods as short as months [9]. Between two and ten other sites from disjunct outcrops were sampled from the vicinity of each of the four focal sites within each region (Figure 1 and Additional file 1, Table S1). In general all accessible outcrops with copepod pools immediately adjacent to the focal sites were sampled and then outcrops were sampled at increasing distances away. The sampling on Point Loma was not as extensive as for the other three regions, only two other outcrops to the south of the SD population were sampled.

Additional samples were taken from locations outside of these regions (we will call them "additional sites") to serve as potential outgroups for intra-region comparisons and to help clarify the relationships of regions to one another (Figure 1). With the combination of focal region sites and additional sites, the sampled populations in southern California (Los Angeles and south) include most of the major areas containing *T. californicus *for this region. Near Santa Cruz, the additional site at Pescardero (PES) was sampled for this study, a sampling that is not an exhaustive set of the *T. californicus *sites around central California. Several other geographically distant sites in California and British Columbia, Canada were also included in phylogenetic analyses for *CYTB*.

### Sequencing Cytochrome b and RISP

Complete *CYTB *sequences were obtained from individual copepods from each of the sites (listed in Figure 1) to compare the extent of population divergence within and between sites and regions. Soon after collection, DNA from individual copepods was prepared using a simple proteinase-K cell-lysis method [23]. The complete *CYTB *gene was PCR amplified from these individuals using either a conserved set of primers or unique primer sets developed for the SD, AB, and SCN regions (see Additional file 2, Table S2) that amplified a product of between 1300 to 1800 bp in size depending on the primer sets used. PCR products were directly sequenced using Big-Dye Terminator and run on a capillary sequencing machine. Direct sequencing of the complete *CYTB *(1131 bp) was performed for 10 or more individuals from most sites (see Table 1 for numbers); generally only two individuals were sequenced for *CYTB *from each of the additional sites.

**Table 1:** Polymorphism in *CYTB *within focal regions for *T. californicus*

Region	Site	Indivs.	Hap. #	Syn.^1^	Nonsyn.	π_syn_^2^	π_total_	Taj-D^3^	FW-H^4^
Santa Cruz	NB1	10	1	0	0	0	0	NA	NA
	NB2	10	3	1	1	0.00127	0.00049	-0.69	0.44
	BH	10	2	1	0	0.00127	0.00031	0.01	-1.07
	SCN	15	3	5	3	0.00441	0.00184	-0.57	-1.41
	SC2	5	1	0	0	0	0	N/A	N/A
	SC3	10	3	5	1	0.00618	0.00185	-0.06	-0.71
	SC4	10	2	3	3	0.00214	0.00106	-1.79*	-6.04**
	SC5	10	2	4	1	0.00285	0.00088	-1.74*	-4.44**
	LH	10	2	1	2	0.00198	0.00147	2.06*	0
	CCR1	10	3	6	2	0.00428	0.00141	-1.88*	-2.13
	CCR2	10	3	6	3	0.00487	0.00173	-1.68^+^	-1.42
	Total	110	12	10	6	0.00996	0.00359	1.17	1.11
	Avg.		2.27			0.00266	0.0010		
Palos Verdes	FR1	10	4	3	1	0.00308	0.00094	-0.9	-5.42**
	FR2	10	3	14	1	0.02316	0.00601	1.3	-1.07
	RsPt	10	7	9	0	0.00929	0.00230	0.8	-4.89*
	PVL	10	3	1	2	0.00071	0.00083	-0.43	-1.6
	ABR	10	1	0	0	0	0	N/A	N/A
	AB1	11	5	8	1	0.01003	0.00287	0.22	1.31
	AB2	11	7	10	2	0.00861	0.00281	-0.98	-0.44
	AB3	10	8	12	3	0.00859	0.00267	-2**	-3.2*
	IP	10	5	7	1	0.00559	0.00156	-1.64	-4*
	RP1	10	6	9	2	0.00996	0.00309	-0.46	-2.76^+^
	RP2	10	5	2	3	0.00272	0.00135	0.02	0.71
	Total	112	47	73	14	0.03399	0.00912	-1.08	-40.3***
	Avg.		4.91			0.00743	0.00222		
La Jolla	SIO	14	3	1	1	0.00051	0.00036	-0.96	0.35
	LJP1	20	10	9	4	0.00753	0.00265	-0.66	-2.85^+^
	LJP2	13	5	11	1	0.00973	0.00263	-0.94	-7.02*
	LJP3	10	1	0	0	0	0	N/A	N/A
	LJS	14	6	5	1	0.00299	0.00086	-1.73	-1.09
	NAUT	13	4	10	0	0.00556	0.00136	-2.09**	-2.39*
	BR	14	7	11	1	0.01426	0.00361	0.33	-3.47^+^
	Total	98	32	37	7	0.0173	0.00457	-0.87	-8.9*
	Avg.		5.14			0.00580	0.00164		
Point Loma	SD	20	7	13	0	0.00793	0.00193	-1.46	-1.21
	SCL	7	3	2	1	0.00206	0.00076	-1.35	-0.95
	LS	8	2	1	0	0.00090	0.00022	-1.05	0.21
	Total	35	12	16	1	0.00787	0.00198	-1.39	-0.88
	Avg.		4.00			0.00363	0.00097		

^1^Number of synonymous polymorphisms (syn.) or non-synonymous (nonsyn.)

^2^Average number of pairwise differences between sequences within a population for synonymous (π_syn_), non-synonymous (π_non-syn_), and all (π_total_) sites.

^3^Tajima's D value with significance indicated by an * (P < 0.05) or an ** (P < 0.01), and ^+ ^indicating 0.05 < P < 0.1.

^4^Fay and Wu's H value with significance indicated by an * (P < 0.05), an ** (P < 0.01), or an *** (P < 0.001), and ^+ ^indicating 0.05 < P < 0.1.

For a nuclear gene comparison to *CYTB*, the *RISP *gene was sequenced from copepods from a subset of the above sites. This gene includes an 883 to 1113 bp intron that provides extensive variation between individuals and sites. Primers to amplify nearly the complete coding sequence for this gene were previously developed ([10] and see Additional file 2, Table S2). PCR amplifications were done using *PfuUltra *Hotstart High-Fidelity DNA Polymerase from (Stratagene, Cedar Creek, TX). The resultant *RISP *PCR products were cloned using a zero blunt TOPO cloning kit (Invitrogen, Carlsbad, CA). For most individuals, both direct sequencing of the PCR products and sequencing of cloned sequences were done to both identify the heterozygous sites and the haplotype phases. Sequences of RISP of 1585, 1753, and 1838 bp (size differences result from variation in intron size) were obtained from Palos Verdes, La Jolla, and Santa Cruz region copepods respectively from at least 4 individuals from each site. Two haplotype sequences were constructed for each individual for *RISP*, in a few cases these haplotypes were identical for individuals that were homozygotes. *RISP *and *CYTB *sequences have been submitted to GenBank with the accession numbers GQ140634–GQ141051 and are also included in the additional files (see Additional files 3 and 4).

### Sequence Analysis and Population Structure

Sequences were edited and aligned using Sequencher version 4.7 (Genecodes, Ann Arbor, MI). The program DNAsp version 4.1 [29] was used to examine levels of polymorphism, divergence, and compute statistical metrics of selection (e.g. Tajima's D [30], Fu and Li's D and F [31]), compute R, the recombination parameter [32], and R_m_, the minimum number of recombination events [33] for the *CYTB *and *RISP *sequences. Fay and Wu's H [34] was calculated for each site using the closest available outgroup (PES for Santa Cruz, Dume for Palos Verdes, and LagBch for Point Loma and La Jolla). The significance of H was tested by running coalescent simulations in DNAsp using up to 10 000 replicates with no recombination for *CYTB *and several different parameter values of moderate recombination for *RISP*. McDonald and Kreitman tests (MK test [35]) were performed using divergence and polymorphism data from DNAsp and hand counts. The program Arlequin v3.11 [36] was used to compute analyses of molecular variance (AMOVA) to partition variation between sites, regions, and individuals, and to calculate pairwise F_ST _values between sites based on genetic divergence and their significance (using 10 000 permutation replicates) for *CYTB *and *RISP*. Isolation by distance testing was performed using the IBD web service version 3.02 with the significance assessed using a Mantel test [37]. Nested clade analysis was implemented using the program GeoDis version 2.2 [38] for the *CYTB *dataset using the inference key of Templeton [39]. Multilocus nested clade analysis was not attempted for the combined *RISP/CYTB *datasets due to extensive recombination within regions for *RISP*.

Phylogenetic analyses were conducted using several different methods: Haplotype networks were constructed for *CYTB *by hand using a parsimony method and also using statistical parsimony via the program TCS v1.18 [40]. Gene trees for the *CYTB *sequences were constructed under the parsimony criterion using the program PAUP* version 4.0b10 [41]. A Bayesian tree was also constructed for the same set of *CYTB *sequences using the program MrBayes v3.1.2 [42]. For this analysis, a GTR model with gamma-distributed rate variation and a proportion of invariant sites was run for 800 000 generations with a 200 000 generation burn-in time (sampled every 1000 generations). Alternate models from GTR were not explored with Bayesian analyses. Gene trees for *RISP *were done with PAUP* using a neighbor-joining analysis (NJ). A full parsimony analysis of all sequences could not be completed for *RISP *in a single analysis most likely due to a history of recombination between alleles within regions. Analyses for each region with subsets of sequences from all other major clades could be completed in separate analyses using parsimony and the correspondence between the relationships found in these parsimony analyses and those found in the NJ tree was then determined.

## Results

### CYTB variation within and between regions

**Figure 2:**
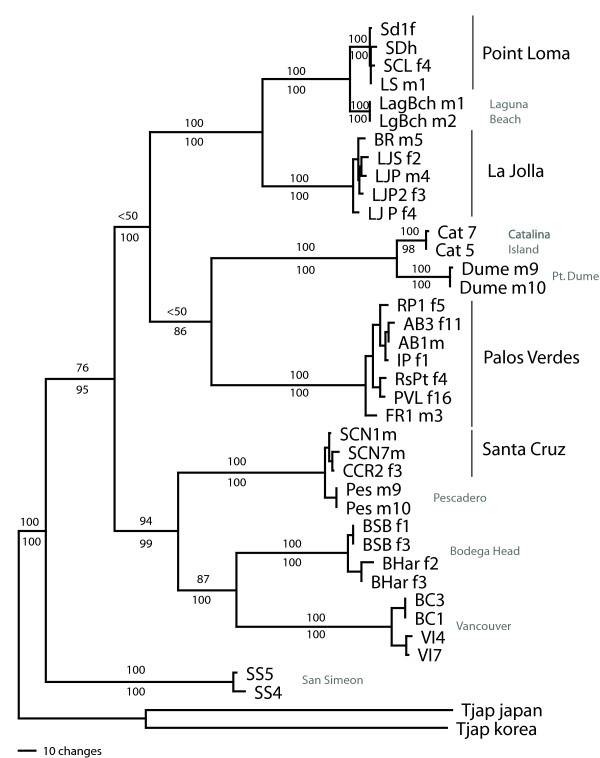
**Gene tree for *CYTB *for selected sites of *T. californicus***. Tree is one of 16 most parsimonious trees based on the complete nucleotide sequence of *CYTB *(1131 bp) for taxa representing the range of diversity found within each of the regional areas and the additional site sequences. Bootstrap numbers based upon 10 000 replicates are given above branches (only those supporting regional clades and branches showing the relationships of additional sites are shown). *T. japonicus *nucleotide *CYTB *sequences from both Korea and Japan are used to root tree (from GenBank accession numbers AY959338 and AB060648 respectively). The relationships between the regions and additional sites are the same as those obtained in a Bayesian phylogenetic analysis. Credibility values from this analysis are given below each branch.

Phylogenetic analyses of *CYTB *sequences reveal deep splits among the four different focal regions, with considerably less variation occurring among sites within each region (Figure 2). Pairwise divergences among regions are between 19 and 22 percent (see Additional file 5, Table S3; with the exception of individuals from the Point Loma and La Jolla sites that have 10.2% average divergence and are only 8 km apart). Although the Palos Verdes region is the closest geographically to each of the CAT, Dume, and LagBch sites, it is highly diverged from each at *CYTB*. In contrast, comparisons of select other geographically distant sites show much lower genetic divergence-LagBch and Point Loma sites (2.3% divergence, 107 km apart), PES and Santa Cruz sites (1.0% divergence, 47 km apart), and Dume and Cat sites (3.1% divergence, 68 km apart). Within each of the four focal regions, pairwise divergences among individuals from different sites are much lower (below 1.3%), and levels of polymorphism within individual sites are relatively low as well (below 0.7%) (Table 1). An analysis of molecular variation (AMOVA) shows that although most variation at *CYTB *is partitioned between regions, there is also significant variation between sites within regions as well (Table 2a).

**Table 2:** Differences between regions, sites, and individuals for *CYTB *and *RISP *in an AMOVA

Comparison^1^	Source	d.f.	SS	Variance Components	% variation	Fixation index	P-value
a. *CYTB*-among regions	Among regions	3	27574	107.70	96.8	F_CT _= 0.968	<0.0001
	Among sites within regions	28	823	2.61	2.34	F_ST _= 0.991	<0.0001
	Within sites	323	312	0.97	0.87		<0.0001
b. *CYTB*-within Palos Verdes	Among non-adjacent groups	2	247.1	4.65	71.19	F_CT _= 0.712	0.0023
	Among sites within non-adjacent groups	5	28.3	0.41	6.24	F_ST _= 0.774	<0.0001
	Within sites	74	109	1.47	22.57		<0.0001
c. *RISP*-among regions	Among regions	2^2^	6595	143.8	96.5	F_CT _= 0.965	0.009
	Among sites within regions	6	91.7	1.44	0.97	F_ST _= 0.975	<0.0001
	Within sites	63	234.4	3.72	2.50		<0.0001

^1^For both (a) and (c) the levels tested are the regions, sites, and individuals. In (b) the Palos Verdes region is considered because for this region it is possible to define subregional grouping based on habitat and sampling schemes [adjacent groups were defined as follows: (AB, AB2, AB3, ABR), (RP1, RP2), and (FR1, FR2). AMOVA were conducted in Arlequin using pairwise distances between populations.

^2^The Point Loma region contained only the SD site for the RISP results and was therefore not included as a separate group.

**Figure 3:**
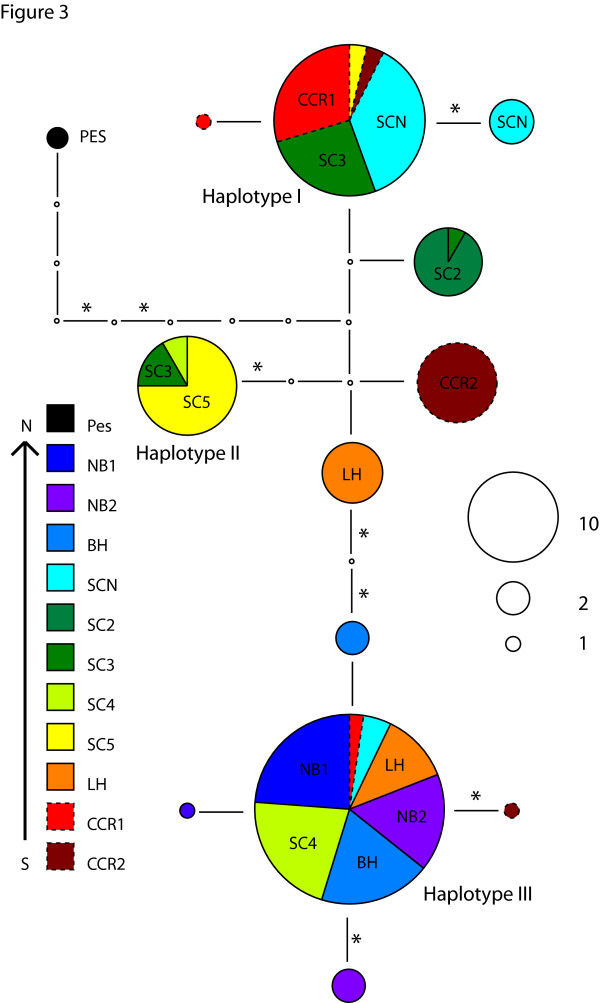
***CYTB *haplotype network for Santa Cruz region *T. californicus***. Each segment represents one nucleotide difference with non-synonymous changes indicated by an *. Size of circle indicates the number of haplotypes while the colors depict the sites from which these haplotypes come. Sites are shown on the network when more than two individuals from one site have the same haplotype. The haplotypes labeled I, II, and III are shared by three or more sites. Dashed lines surrounding some haplotypes are solely for visual clarity.

There is significant genetic structure in each region evident at scales down to the site to site comparisons but also some difference in the degree of haplotype sharing across each region. The Santa Cruz region shows the greatest degree of *CYTB *haplotype sharing across sites with three common haplotypes (Figure 3) that are shared by at least three different sites. Individuals from geographically separated sites share haplotypes (e.g. CCR1/2 and SCN share haplotype I, and NB1/2 and LH share haplotype III), which results in no significant correlation between genetic distance (F_ST_) and geographic distance within this region (r = 0.092, p = 0.25) despite general pairwise F_ST _values among sites higher than 0.5 (all *CYTB *F_ST _values in Additional file 6, Table S4). However, there are two groups of Santa Cruz sites that do not show significant pairwise F_ST _values for comparisons of sites within each group (significance assessed by permutation tests and significance threshold adjusted using a sequential Bonferroni procedure to adjust for multiple tests). One group is the SCN/SC3/CCR1 sites that share haplotype I (SC3 is also not significantly differentiated from the SC2 and SC5 sites), while a second group, the NB1/NB2/SCN4/BH/LH sites, share haplotype III.

**Figure 4:**
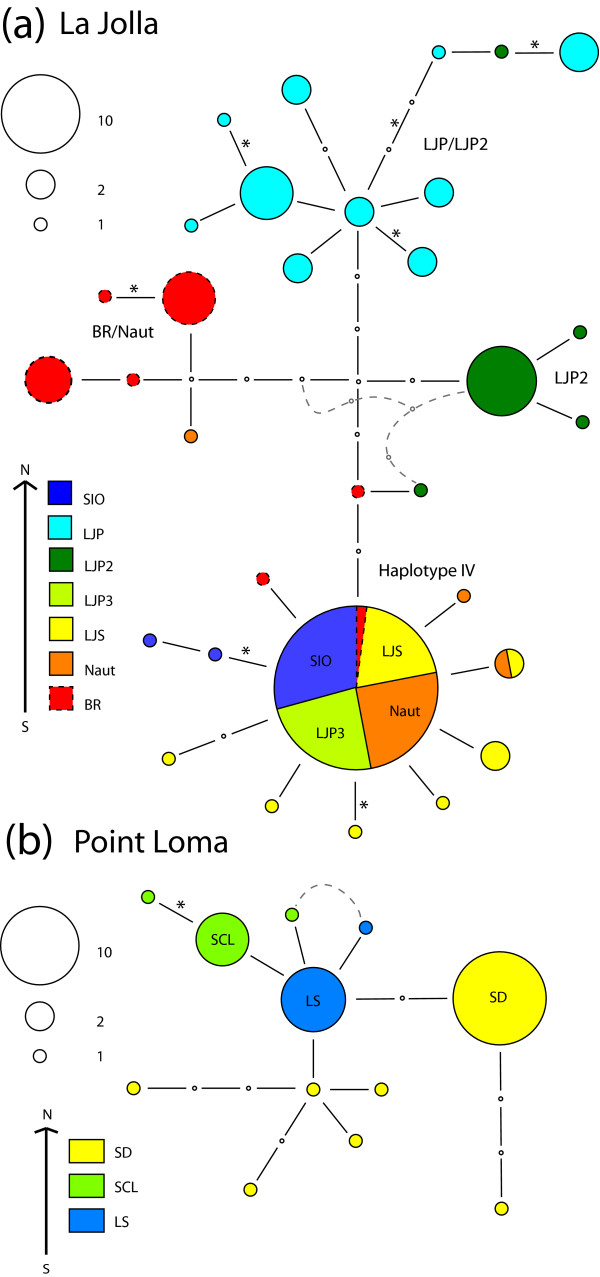
***CYTB *haplotype network for La Jolla (a) and Point Loma (b) regions for *T. californicus***. Network is depicted as described in Figure 3. For La Jolla, the three groups of haplotypes that cluster by geography are labeled in addition to the haplotype IV that is shared by five sites. For Point Loma, haplotypes are labeled by site when more than two individuals share a haplotype from the same site. There are 23 synonymous and 1 non-synonymous substitutions in *CYTB *separating Point Loma and Laguna Beach (and 101 synonymous and 6 non-synonymous substitutions separating Point Loma and La Jolla). Dashed lines indicate alternative haplotype relationships found in statistical parsimony analyses.

The two San Diego county regions show different patterns in their *CYTB *haplotypes than the Santa Cruz region. The La Jolla region displays an intermediate level of haplotype sharing (Figure 4a) with one common haplotype (haplotype IV) shared by five different sites, but clear geographic clustering for three other groups of closely related haplotypes. In contrast to the Santa Cruz haplotypes where several non-synonymous differences are located on internal branches, in the La Jolla region non-synonymous differences are largely found at or near tips and not on the branches separating the four main groups of haplotypes. Although there is less haplotype sharing within this region than the Santa Cruz region, there is no signature of isolation by distance (r = -0.045, p = 0.62). Only one group of sites showed non-significant differentiation in pairwise comparisons of F_ST_, the LJS/NAUT/LJP3/SIO sites, all of which have haplotype IV at high frequency. F_ST _values are much higher (>0.5) for comparisons among all other sites within La Jolla. Although not sampled as extensively, the nearby Point Loma region does not show any shared haplotypes among its three sites, but there is also no discrete clustering of haplotypes by site either (Figure 4b). The F_ST _values between these three sites on Point Loma range from 0.49–0.63.

**Figure 5:**
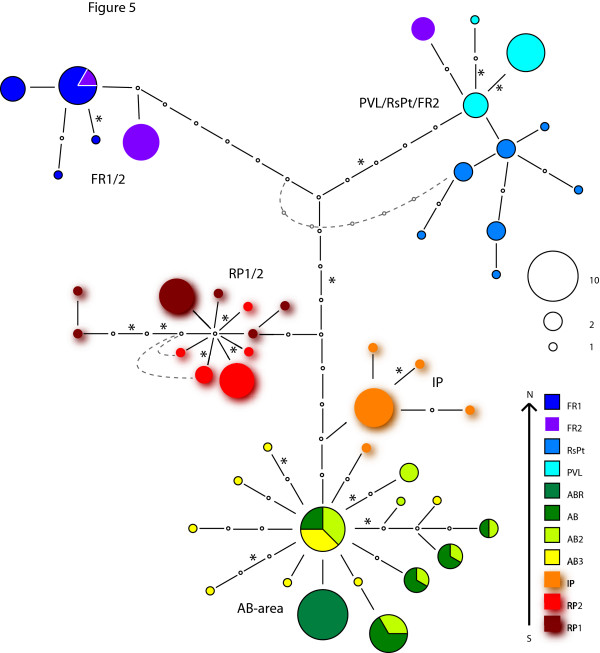
***CYTB *haplotype network for Palos Verdes region *T. californicus***. Network is constructed as depicted in Figure 3. Five clusters of haplotypes that group largely by geography are labeled by sites. Dashed lines indicate alternative haplotype relationships found in statistical parsimony analyses. Haplotypes are shadowed for visual clarity.

The Palos Verdes region shows the greatest intra-region divergences for *CYTB *haplotypes with little haplotype sharing beyond adjacent sites (Figure 5). There are four clusters of haplotypes, which each contain adjacent sites and a fifth cluster of haplotypes that contains the geographically separated PVL/RsPt/FR2 sites (the FR2 site has individuals in two different haplotype clusters). Two of the 24 substitutions separating these five divergent haplotype clusters are non-synonymous. AMOVA for the Palos Verdes region (Table 2b) shows that a large fraction of the genetic variation was between non-adjacent groups and contributes to a strong signal of isolation by distance (r = 0.57 p < 0.0001). Two groups of adjacent sites do not show significant differentiation by F_ST _measures, the AB/AB2/AB3, and RP1/RP2 sites. All other F_ST _values for pairwise comparisons among sites were higher than 0.3.

There is no evidence for major short-term temporal shifts in haplotypes within sites from the replicate sampling of individual sites at different time points. *CYTB *sequences obtained from copepods collected two years apart from the SCN, AB2, and LJP sites were not appreciably different from one another within a site (results not shown). This suggestion of short-term stability mirrors the longer-term stability in haplotype frequency (18 years or more) found for many of these same outcrops using allozyme markers [12].

### Nested clade analyses for CYTB

Nested clade analyses using *CYTB *within each of the four studied regions show patterns of differentiation that are consistent with inferences of restricted gene flow and fragmentation (Table 3). A number of clade levels appear to show the signature of past long distance colonization, fragmentation, or past contiguous range expansion (seven total) indicating the importance of historical events on current population structure. For an equal number of cases (seven), the patterns of variation are consistent with either historical separation or lowered levels of gene flow (e.g., isolation by distance or restricted gene flow).

**Table 3:** Nested clade analyses inferences for *T. californicus *sites based on *CYTB*.

Inference	Description of clades involved
Santa Cruz and PES	
Inconclusive	Haplotype I, CCR1, and SCN
Fragmentation	SC3/4/5 and CCR2
Restricted gene flow or isolation by distance	CCR1/2 and SCN/3/5
Restricted gene flow or isolation by distance	Haplotype II group and SC3/4/5/CCR2
Inconclusive	PES and Haplotype I group
Palos Verdes	
Contiguous range expansion	AB to ABR
Contiguous range expansion or restricted gene flow	RP1 and RP2
Contiguous range expansion or restricted gene flow	AB/AB2/AB3 and AB2/AB3
Long distance colonization	FR2 and PVL
Contiguous range expansion	FR1/FR2 and FR2
Fragmentation or isolation by distance	PVL/FR2 and RsPt
Long distance colonization	AB/IP and IP groups
Fragmentation or isolation by distance	RP, FR, and PVL/RsPt groups
La Jolla	
Long distance colonization or range expansion	BR group and haplotype III group
Contiguous range expansion or restricted gene flow	Among LJP
Point Loma and LgBch	
Contiguous range expansion	SCL and LS
Long distance colonization	SD and SD/SCL/LS

The historical and demographic inferences are listed with a general description of the clade/nesting groups involved.

### Tests for Selection acting upon CYTB

MK tests [35] reveal a pattern of variation consistent with excess non-synonymous polymorphism for 3 of the 4 regions (Table 4). For variation in *CYTB *in *T. californicus *Tajima's D values are biased towards negative values, 22 negative (5 significant) and 6 positive (1 significant) (Table 1). Fu and Li's F and D have a similar pattern of skew towards negative values (see Additional file 7, Table S5). If the frequency spectra of replacement sites alone are examined, Tajima's D values become slightly more negative for the Palos Verdes and La Jolla regions as a whole (the same pattern was not found overall across individuals sites; see Additional file 7, Table S5). Fay and Wu's H values [34] are significantly negative for the same five sites as Tajima's D and Fay and Wu's H is also significant at the RsPt, IP, and FR1 sites (Table 1).

**Table 4:** McDonald/Kreitman tests of polymorphism versus divergence for CYTB.

Region	Outgroup	Fixed syn.	Fixed non-syn.	Poly. syn.	Poly. Non-syn.	P-value
Santa Cruz	BSB	148	17	10	6	<0.005
Santa Cruz	BSB^1^	260	17	10	6	<0.005
Santa Cruz	PES	7	2	10	6	>0.5
Palos Verdes	Dume	166	30	73	14	0.14
Palos Verdes	Dume^1^	336	31	73	14	0.03
La Jolla	LgBch	92	5	37	7	0.04
Point Loma	LgBch	23	1	16	1	>0.5

^1^A Jukes/Cantor correction for multiple hits has been applied to the divergence results for these rows

### Variation for RISP within and among Regions

**Figure 6:**
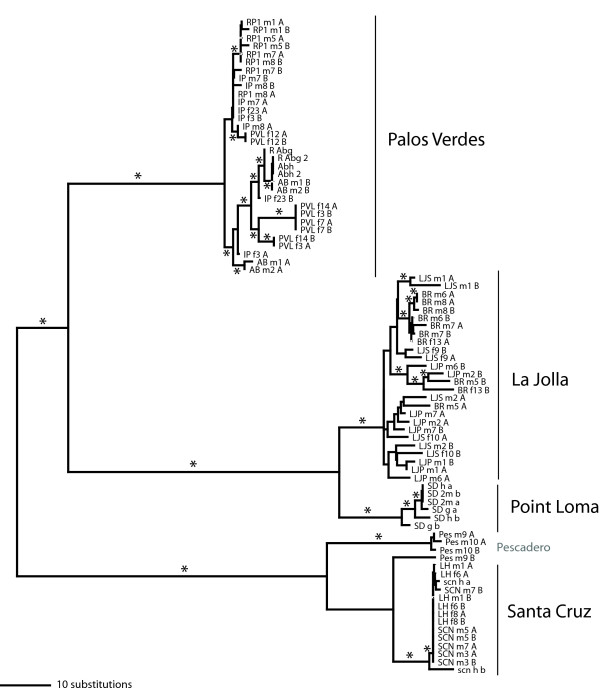
**NJ tree of *RISP *haplotypes from four focal regions for *T. californicus***. Tree is unrooted. Branches that were also supported by consensus trees of partial parsimony analyses of these *RISP *haplotypes are indicated by an *. We were unable to complete a parsimony analysis for the entire dataset but could complete analyses for pruned datasets to examine relationships between regions. Secondarily, the relationships of haplotypes within each region were separately analyzed using a parsimony method and compared to the relationships obtained from the NJ analysis. Intragenic recombination and reticulation of sequences within regions could make parsimony analyses difficult by yielding numerous equally parsimonious trees.

We have sequenced *RISP *from copepods from a subset of the regions and sites that capture much of the diversity of haplotypes found for *CYTB*. A NJ tree constructed from the *RISP *sequences illustrates the general patterns of divergence (Figure 6). There is substantial divergence among regions, but absolute levels of sequence divergence between regions for *RISP *are not nearly as high as those seen for *CYTB*. For example, for synonymous site comparisons between the Palos Verdes region and Santa Cruz region uncorrected pairwise divergence is 70 percent for *CYTB *and 5.5 percent for *RISP*. For the *RISP *gene tree (Figure 6) within each region there is much less clustering of haplotypes for single sites or groups of sites than was seen for *CYTB *(Figure 2).

With the exception of the Santa Cruz region there is very little haplotype sharing either within a site or between sites for *RISP*, but individual polymorphisms are often shared across sites within a region. Compared to *CYTB*, for *RISP *there is somewhat less genetic divergence between sites within a region as reflected by the lower percentage of variation among sites within a region in an AMOVA for RISP (Table 2c) and the limited geographic structure within regions evident in the NJ tree (Figure 6). For the two sites in the Santa Cruz region there are only 7 singleton polymorphisms segregating at RISP (and the two sites are not significantly genetically differentiated at *RISP*; all *RISP *pairwise F_ST _values are in Additional file 8, Table S6). In contrast, for the 4 alleles obtained for the additional site to the north, PES, there is much more variation, including an allele (m9-B) that appears to be a past recombinant between PES and Santa Cruz allele-types (see Additional file 9, Table S7). In the La Jolla region, 19 of 22 non-singleton polymorphisms are shared across sites (see Additional file 10, Table S8). The F_ST _values indicate significant but relatively low levels of differentiation between these three La Jolla sites (with LJS and LJP having the lowest F_ST _value: 0.064 despite having an F_ST _of 0.74 for *CYTB*). The Palos Verdes region sites show the least shared polymorphism with only 9 of 21 non-singletons shared across sites (see Additional file 11, Table S9). Compared to the other two regions, pairwise differentiation is highest among Palos Verdes sites as measured by F_ST _values, which range from 0.28 and 0.36 for IP/RP1 and IP/PVL to 0.75 for RP1/AB.

### Patterns of Polymorphism in RISP

All of the polymorphisms within regions for RISP are either in non-coding positions (primarily the large intron) or in synonymous sites in the coding regions (Table 5). As expected from the lack of haplotype variation, the Santa Cruz sites show the lowest levels of nucleotide polymorphism with the average π_tot-sil _10-fold lower than the corresponding value from the La Jolla sites. Estimates of intragenic recombination from both the recombination parameter (R) and minimum number of recombination events (R_m_) are both non-zero in the Palos Verdes and La Jolla samples indicating a history of recombination in these samples of alleles within regions. The lack of variation within the Santa Cruz region precludes an estimation of recombination rate within that region.

**Table 5:** Polymorphism Within Regions and Sites for *RISP*.

Outcrop	Site	Alleles	Hap. #	Nonsyn^1^	Syn	Silent	Indel #	π_syn_^2^	π_tot-sil_	π_total_	Taj-D^3^	FW-H^4^	R^5^	R_m_^6^
Santa Cruz	SCN	8	3	0	2	5	1	0.00346	0.00093	0.00069	-1.60^+^	-1.54	0	0
	LH	6	2	0	0	0	1	0	0	0	N/A	N/A	-	-
	Total	16	6	0	2	5	2	0.00198	0.00053	0.00040			0	0
	Avg.							0.00173	0.00047	0.00035				
Palos Verdes	PVL	8	3	0	2	15	2	0.00574	0.00639	0.00449	1.20	-3.14	0.0028	0
	AB1	8	4	0	0	5	2	0	0.00189	0.00134	0.34	-1.50	0.0004	0
	IP	8	6	0	2	8	3	0.00348	0.00228	0.00162	-0.86	1.71	0.0011	0
	RP1	8	5	0	1	4	1	0.00294	0.00106	0.00075	-1.03	-0.21	-	0
	Total	32	17	0	5	24	4	0.00745	0.00568	0.00404	0.15	-4.19^+^	0.0075	1
	Avg.							0.00304	0.00291	0.00205				
La Jolla	LJP1	8	8	0	1	17	4	0.00170	0.00431	0.00315	-0.89	-5.36*	0.0743	1
	LJS	8	8	0	1	20	3	0.00167	0.00642	0.00462	0.07	-0.29	0.1166	5
	BR	10	6	0	0	18	5	0	0.00430	0.00315	-0.71	-5.24*	0.0009	5
	Total	26	22	0	2	33	7	0.00367	0.00720	0.00425	-0.68	-2.46	0.0514	9
	Avg.							0.00112	0.00501	0.00364				
Point Loma	SD	6	4	0	0	7	0	0	0.00236	0.00173	-0.25	-4.53*	-	-

^1^The number of non-synonymous (nonsyn), synonymous (syn), and the combined non-coding and synonymous (silent).

^2^Average number of pairwise differences between sequences within a population for non-synonymous (π_non-syn_), synonymous (π_syn_), combined synonymous and non-coding (π_tot-sil_), and all (π_total_) sites.

^3^Tajima's D value, none were significant, but an ^+ ^indicates 0.05 < P < 0.1.

^4^Fay and Wu's H value with significance indicated by an * (P < 0.05), and ^+ ^indicating 0.05 < P < 0.1.

^5^Recombination parameter (R) could not be estimated for some sites (-).

^6^Minimum number of recombination events (R_m_) could not be estimated for some sites (-).

The frequency-spectra of polymorphisms for *RISP *in these different sites and regions of *T. californicus *are not as biased towards low frequency polymorphisms as seen for *CYTB *(Table 5). For Tajima's D there are 6 positive values and 3 negative with no significant values. The only significant values for Fu and Li's D and F are from the PVL site and are positive (1.56 D and 1.64 F, p < 0.05 for each; see Additional file 7, Table S5). Significantly negative values of Fay and Wu's H (with low levels of recombination used in coalescent simulations) are found for the BR (-5.24), LJP (-5.36), and SD (-4.53) sites (Table 4).

## Discussion

### Signals of population structure and history at a broad scale

Although striking, the patterns of extreme genetic divergence observed among regional populations of *T. californicus *are not unprecedented, with high levels of mtDNA divergence found among populations in previous studies [3,5,10,11]. The high levels of genetic differentiation between *T. californicus *populations are likely to reflect generally low levels of gene flow between outcrops and their ability to persist on outcrops despite the ephemeral nature of individual copepod pools within a single outcrop during the course of a season [14,15]. In some cases genetic divergence is across short distances, for example there is 10 percent divergence in *CYTB *between the Point Loma and La Jolla regions despite the distance between these regions being equivalent to the distance between the end sites in the La Jolla region (~9 km). At a slightly larger scale, the LagBch and Palos Verdes regions are 20 percent divergent and 53 km apart. Such patterns of high divergence in mtDNA genes have also been found in a congener, *T. japonicus*, with up to 28 percent divergence between populations in *CYTB *among locations in Japan and Korea [43].

Often the presence of highly genetically divergent lineages within a nominal species is thought to reflect the presence of cryptic species. Although defining cryptic species in allopatric populations is a difficult problem, a number of factors suggest that *T. californicus *may be still considered a single biological species (although they could potentially be considered separate species under other species definitions): These populations are able to interbreed in the lab and produce hybrids, including advanced generation hybrid lineages [24,44], there is no evidence for premating isolation in crosses between populations [45,46], and little consistent morphological divergence among populations has been found in studies done to date [12,47]. Other groups of copepods have also been found to have divergent genetic lineages that are often morphologically similar suggesting morphological stasis may be common in copepods [48-50]. In contrast to *Tigriopus*, in a number of other cases, genetically divergent lineages within a nominal species are sometimes found sympatrically distributed suggesting that these lineages do represent cryptic species [51-56]. Not all copepod species show these dramatic patterns of genetic differentiation between populations with some species having world-wide distributions and apparent genetic exchange between ocean basins [54,57].

Even though many comparisons of geographically distant populations of *T. californicus *show high levels of divergence, not all comparisons show this pattern, suggesting that occasionally gene flow can reach over longer spatial scales in this species. For example in the current dataset, LagBch and Point Loma are 2.3 percent divergent and 98 km apart, Dume and CAT are 3.0 percent divergent and 68 km apart, and Santa Cruz and PES are 1.0 percent divergent and 47 km apart. In some cases these population comparisons are not the most geographically proximate, for example LagBch is much closer geographically to the other populations in the Palos Verdes region (34 km) rather than Point Loma populations (107 km). Edmands [5] found that from Oregon north to Alaska there was very little divergence in *COI *sequences among *T. californicus *populations and suggested that this could reflect recolonization after the last ice age. These seemingly contradictory patterns of low divergence in spite of extensive geographic separation between select populations of *T. californicus *within California may be indications of past long distance colonization events; thus, implying that although rare, long distance dispersal also plays a role in the evolutionary history of this species.

The degree of temporal stability of the outcrops and pools that comprise copepod habitat may play a large role in extinction/recolonization dynamics in *T. californicus*. Over short time scales, factors such as the number, size, and location of pools are likely to determine whether copepod habitat persists on an outcrop from year to year. At much longer timescales, sea level changes due to water storage in ice sheets during periods of glaciation will have major impacts on the distribution of available intertidal habitat. In fact, over the past 500 000 years (500 kyr) sea levels have cycled repeatedly with sea levels in interglacial periods at or slightly above current levels and 100–150 m below current sea levels at glacial maxima. During the past 100 kyr sea levels declined unevenly down to at least 100 m below the present sea level at the glacial maximum about 21 kyr [58] and then increased rapidly until about 8 kyr when they reached their present levels (with potentially slightly higher levels in the mid-Holocene [59]). The distribution of available *T. californicus *habitat in the past would have been rather different from the present distribution with the current pool areas only inhabitable for at most 8 kyr. Recolonization of newly available outcrops would likely involve rare long-distance dispersal events from other outcrops. It is possible that some geographically disjunct areas with low levels of *CYTB *sequence divergence seen in the current dataset (e.g. Laguna Beach/Point Loma and Catalina Island/Pt. Dume) could reflect the lack of available habitat in one region during the last glacial maximum followed by dispersal from other distant refugial populations once suitable habitat became available again.

In addition to the shifting availability of copepod habitat associated with sea level changes, the outcrops themselves are not stable over the longer time periods that would be required with standard mtDNA molecular clocks to produce the *CYTB *divergences uncovered among *T. californicus *populations. The uplifted headlands containing the rocky-intertidal regions in this study from Southern California do not appear to be more than one million years (1 myr) old. The Point Loma and La Jolla headlands resulted from uplift associated with the Rose Canyon fault leading to the emergence of these areas first as offshore islands less than one myr ago [60,61]. Similarly, the Palos Verdes region was at one point submerged and uplift resulted in its emergence as an island, also less than 1 myr ago, which was eventually connected to the mainland by the emergence of the Los Angeles basin [62]. Prior to that, it appears likely that both the San Diego and Los Angeles areas were extensively estuarine in nature until roughly 2–3 myr ago [63] and may not have supported much *Tigriopus *habitat. Interestingly, for several other coastal marine or intertidal animal taxa the Los Angeles region is the site of intra-specific phylogeographic breaks [64]. Given the relatively young age of the current rocky headlands in southern California, it is possible that the divergence in *CYTB *between copepods from nearly adjacent regions such as Point Loma and La Jolla (~10 percent) or even Point Loma and Palos Verdes (~20 percent) has accumulated over a period of less than 1 myr if this divergence is not due to colonization from previously isolated source populations. With divergence times of 1 myr or less, these genetic divergences would reflect substantially elevated mutation rates for these copepod lineages (i.e. >10 percent/myr). The observation that mtDNA genes evolve much more rapidly than nuclear genes in *T. californicus *[10], could also support elevated absolute mtDNA rates in this taxon. However, given that long distance dispersal events seem to occur occasionally in this species, it is also possible that dispersal from genetically divergent source populations (perhaps populations now extinct) could explain the high levels of genetic divergence between these populations.

### Signals of population structure and history at a fine scale

This study differs from previous datasets from *T. californicus *by determining the patterns of divergence in DNA sequence at a fine geographic scale (here for both a nuclear and a mtDNA gene). Although mtDNA sequence divergences in *CYTB *are much more modest within regions (<1.3 percent), there is significant genetic structure between the majority of sites in all four regions as revealed by F_ST _distances and AMOVA (Table 2). The nested clade analyses support both historical separation and separation with limited gene flow between many clades within regions (Table 3); however, the reliability of individual inferences generated from nested clade analyses has been questioned [65]. We have done exploratory analyses using likelihood-based IM analyses on selected *T. californicus *sites under a model of isolation with gene flow using a MCMC method [66] and these analyses revealed both strong signals of limited gene flow between some sites and much higher gene flow between other sites consistent with results obtained from F_ST _and nested clade analyses (results not shown). Taken together, the F_ST _analyses, AMOVA, nested clade analyses, and limited IM analyses suggest significant historical separation and limited contemporary gene flow among a large fraction of these sites within each region.

An advantage of using *CYTB *over nuclear genes is that a mtDNA gene tree can yield insights into population history that may be lost due to recombination in nuclear gene trees. In each of the four regions the *CYTB *haplotype networks suggest slightly different patterns of historical isolation. The Palos Verdes region shows haplotype groupings that are largely geographically limited (Figure 5), the major exception to this is the FR2 haplotype that is closely related to the PVL haplotypes (and may be an example of fairly recent long-distance dispersal). These results suggest isolation resulting from geographic separation is dominant in shaping genetic diversity in this region and may reflect substantial isolation between these groups of sites since perhaps the last glacial maximum 21 kya or the return to present sea levels about 8 kya. The La Jolla *CYTB *haplotype network (Figure 4) suggests that there may have been significant historical isolation between at least three outcrop areas with only limited gene flow. For La Jolla, however, there are also several geographically separated sites (SIO, Naut, LJP3, and LJS) that show little evidence of isolation, a pattern that must reflect either on-going gene flow or recent colonization. Of the four sites, the LJS outcrop currently has the most extensive copepod habitat and could serve as a source for recolonization of the other sites after extinctions. Note that the SIO and LJS sites are not adjacent (LJP1/LJP2 sites are closer to SIO) suggesting that migration does not always occur in a stepwise fashion. For the Santa Cruz and Point Loma regions, although there is evidence for limited gene flow from F_ST _measures, *CYTB *haplotypes are either shared across many sites (Santa Cruz, Figure 3) or nested amongst one another (Point Loma, Figure 4). This pattern would seem most likely to reflect limited gene flow over short time scales but more mixing over longer periods, perhaps through occasional extinction and recolonization events of single outcrops.

There are substantial differences in the patterns of sequence divergence found between *CYTB *and *RISP*, with much greater levels of divergence among sites within a region for *CYTB *than for *RISP*. This lack of divergence is not due to a lack of variation in *RISP*, for the La Jolla and Palos Verdes regions, both average and total levels of polymorphism across sites within a region are similar for *RISP *and *CYTB *(Tables 1 and 4). In both of these regions phylogenetically distinct clusters of haplotypes from a limited number of sites are found for *CYTB*, while very few differences are fixed between sites within a region for *RISP*. This difference between the two loci in amount of genetic differentiation suggests that the mtDNA-encoded *CYTB *is behaving as if it has a lower effective population size. If males and females have the same average reproductive success, then mtDNA would be expected to have roughly a fourth the effective population size of a nuclear-encoded gene given its clonal, female-limited inheritance. In some cases the differences between the two markers are extreme (for example the LJP1/LJS comparison shows almost no structure at *RISP*, while LJP1 haplotypes form a distinct clade for *CYTB*, Figures 4 and 6). Geographically-limited selective sweeps occurring anywhere on the linked mtDNA molecule are one possible explanation for a pattern of greater differentiation of mtDNA if the sweep causes polymorphic sites to become fixed within an outcrop for *CYTB *but does not affect *RISP*.

### Signals of non-neutral evolution

Given the evidence outlined in the introduction for the accumulation of functional and fitness differences in mtDNA among populations of *T. californicus *it is instructive to look at the patterns of sequence variation to determine if signatures of non-neutral evolution can be found and if there is any evidence for coupled selection in both *CYTB *and *RISP*. For *CYTB *in the La Jolla and Santa Cruz regions, MK tests suggest an excess of replacement polymorphism, which could be attributable to segregating slightly deleterious mutations. The negative values for Fu and Li's D and F and Tajima's D tests indicate excess low-frequency mutations in many of the sites as well, which could indicate some combination of a recovery from a recent selective sweep, expanding populations, or slightly deleterious mutations retained at a low frequency but unable to increase in frequency. Up to 20 of 29 non-synonymous polymorphisms within the three regions are found on terminal branches suggesting recent origin and many of these (10 of the 20) are singletons, indicating low frequency. It then appears that some fraction of the non-synonymous polymorphism is likely to consist of slightly deleterious mutations that remain at low frequency and are generally quickly lost from populations. Polymorphism in mtDNA often shows an excess of slightly deleterious mutation in comparisons of polymorphism within and divergence within and between species [67]. These rare deleterious mutations are unlikely to impact inferences about population structure and historical relationships; however, they may impact the frequency spectrum of polymorphisms and produce excess low frequency variation that could otherwise suggest the hypothesis of a population expansion. Slightly deleterious mutations in mtDNA could also contribute to intergenomic coadaptation if they occasionally go to fixation (perhaps due to lowered effective population size during a population bottleneck) and then the fitness effects are subsequently compensated by changes in nuclear-encoded proteins of the electron transport system as suggested by the compensatory co-adaptation model [68].

Some of the non-synonymous polymorphisms within regions do not fit into the pattern of rare slightly deleterious mutation, for example, in the Santa Cruz region there are three non-synonymous substitutions of nine total substitutions occurring on the branches between the three most common haplotypes (Figure 2). A comparison of the divergence amongst these three haplotypes to the divergence to the Bodega Head outgroup in a variant of a MK test (6:3 vs. 149:6 synonymous: non-synonymous polymorphism vs. synonymous: non-synonymous fixed; χ^2 ^test, p = 0.03) suggests that non-synonymous changes are in excess amongst the three Santa Cruz haplotypes. These polymorphisms are shared across sites at moderate frequencies which suggests that they are not deleterious mutations. Given the excess replacement variation seen on these internal branches compared to fixation to an outgroup, it is even possible that selection could be acting to maintain some non-synonymous variation at *CYTB *in this region.

Selective sweeps occurring on mtDNA can lower its apparent effective population size further and could contribute to faster fixation of unique haplotypes within species or populations. A few sites show significantly negative values for Fu and Li's F and D, Tajima's D tests, and significant Fay and Wu H tests for CYTB that could indicate recent recovery from a selective sweep and could imply that selection has periodically driven advantageous mutations to near fixation (note that these mutations do not have to be in *CYTB *but could occur anywhere in the mtDNA). However, there are also demographic scenarios that could generate significant values for these tests; for example, significant Fay and Wu's H test values could potentially result from low levels of migration that introduce ancestral alleles back into a population at low frequencies. More extensive sampling of nuclear genes from these populations would be required to determine if a demographic scenario that affects all genes to some degree is likely to explain some of the deviations in allele frequencies found at *CYTB *for this handful of sites.

The RISP protein interacts structurally with CYTB in complex III and functionally as well through its iron-sulfur redox center, which is involved in the transfer of electrons in this complex [69]. Given this close association, evolution in the CYTB protein may be accompanied by coadaptation in RISP. The amino acid sequence of RISP is very well conserved across sites and regions in this dataset, in fact, the only polymorphic or fixed amino acid differences uncovered between sites and regions were the two amino acid differences between regions found previously for the sites AB, SCN, and SD by Willett and Burton [10]. Clearly a large amount of amino acid substitution can occur in CYTB with few changes in RISP amino acid sequence. Within regions we find amino acid changes in CYTB among some high frequency haplotypes (5 total in the Palos Verdes and Santa Cruz regions) and between certain regions (there are 6 amino acid differences in CYTB between La Jolla and Point Loma) with no changes in the RISP protein. These results indicate that structural co-evolution between these two proteins is not occurring within a region or between relatively closely related regions. These results do not rule out the possibility of CYTB coadaptation with other subunits of complex III (there are eight other nuclear-encoded subunits in this complex in most eukaryotes). At a broader scale, between the AB and SD sites, patterns of genetic interactions between *RISP *and two other complex III-associated genes (*CYC *and *CYC1*) suggest that there has been co-evolution between these three nuclear-encoded proteins, but the results also suggested that these interactions potentially did not involve CYTB for those two sites [27].

At the nucleotide sequence level for RISP there is some evidence for non-neutral patterns in allele frequencies in particular with the Fay and Wu's H test (for the BR, LJP, and SD sites). A potential explanation for this pattern is that these sites could have undergone a selective sweep in the region near *RISP *in the genome but demographic effects could provide an alternate explanation. One other unusual pattern for *RISP *is the extremely low level of polymorphism found in the SCN site (Table 4). This is unusual for this site as *RISP *has the lowest levels of silent/non-coding polymorphism of the 11 genes examined to date for the SCN site [10,70]. In an HKA test [71] of polymorphism in these 11 genes at this site compared with divergence to the AB site, the RISP polymorphism is 4-fold lower than expected and contributes to significantly heterogeneous patterns of variation at this site between these 11 genes (results not shown). This could be explained by a selective sweep having occurred near *RISP *in copepods from the Santa Cruz region removing variation in this gene. Overall, for both the *RISP *and *CYTB *genes the analyses of selection and deviations from neutrality indicate that although selection could occasionally impact patterns of variation at these genes in some populations, most of the phylogeographic patterns seen in this dataset are not likely to be the result of selection.

## Conclusion

Given the dramatic patterns of genetic differentiation within and between regions for *T. californicus*, it is clear that gene flow between these sites is limited over short to moderate time spans. It appears likely that both the shorter-term processes of occasional extinction of all pools on single outcrops and subsequent recolonization, and the longer-term processes such as geological uplift and repeated cycles of sea level change with past episodes of glaciation have combined to shape patterns of genetic divergence in this species. If outcrop to outcrop dispersal is limited (as appears likely), the frequency with which all pools on an outcrop go extinct simultaneously on a single outcrop will determine the degree to which an outcrop can diverge from neighboring outcrops. Over longer periods of time, changes in sea levels during cycles of glaciation will radically alter the available intertidal habitat and limit the degree of divergence among outcrops within a region. Among regions the upper limit on the accumulation of genetic divergence appears to be generated by slower acting geological forces such as the uplift of coastline that has generated rocky intertidal areas in southern California over the last million years. However, even these lengths of divergence time between regions are unlikely to explain the high levels of divergence in *CYTB*, and it is possible that there is an unusually high rate of mtDNA substitution in this species if these divergences are not due to colonization of these regions by previously isolated source populations.

A lack of gene flow among outcrops and potentially an elevated mtDNA substitution rate will not explain all of the patterns seen in the data; extinction events followed by long distance recolonization are also likely to play a significant role. The presence of shared haplotypes among multiple outcrops (not exclusively adjacent) is consistent with long-range colonization within regions. Geographically distant populations that show low levels of genetic divergence (some of which are more divergent from more proximate regions) suggest that past long-distance dispersal events occurred between these populations. These patterns set up a bit of a conundrum for this species with evidence for some long-distance dispersal suggesting the potential for gene flow but little gene flow over significant periods of time for comparisons of many nearby sites. One possibility is that long-distance dispersal is a very rare phenomenon and makes little contribution to genetically homogenizing disparate outcrops. It may also be possible that there is a priority effect such that the occasional migrant copepod rarely survives to reproduce upon reaching an inhabited outcrop but may survive and reproduce in uninhabited or sparsely inhabited pools.

Although selection does not appear to dramatically alter phylogeographic patterns at these genes, there are some signs from the data that it could be more subtly impacting the patterns of nucleotide variation in these copepod populations. Non-neutral patterns seen in this dataset are consistent with excess slightly deleterious polymorphism within regions and potential selective sweeps for a few sites. It is possible that these slightly deleterious polymorphisms could occasionally go to fixation during population bottlenecks and then contribute to a compensatory coevolution process between mtDNA and nuclear-encoded proteins. Suggestions of selective sweeps from some sites come from frequency-based tests (Fu and Li's D and F, Tajima's D, and Fay and Wu's H), but for these tests it would be helpful to consider multiple genes to rule out potential demographic effects such as population expansion or contraction. Spatially-limited selective sweeps in mtDNA could potentially contribute to the greater divergence in *CYTB *than *RISP *between some sites. It is possible then that selection upon mtDNA could accentuate genetic divergence between select geographic sites or shift patterns of polymorphism within sites in this system, but it does not appear likely that this selection is radically altering the patterns of genetic variation within and between populations. Therefore, despite accumulating evidence of coadaptation between the nuclear- and mtDNA-encoded proteins of the electron transport system in this species, overall phylogeographic patterns in CYTB and RISP appear to be largely a product of history and not selection.

## Authors' contributions

CSW conceived of the study, collected samples and sequence data, performed some analyses, and drafted manuscript. JTL collected sequence data, conducted analyses, and helped draft and revise the manuscript. Both authors read and approved the final manuscript.

## Supplementary Material

Additional file 1 **Table S1**. Sampling locations for *Tigriopus californicus *in southern and central California. Click here for file

Additional file 2 **Table S2**. Primers used for PCR amplifications for the *CYTB *and *RISP *genes from *T. californicus*. Click here for file

Additional file 3 ***CYTB *sequences in Arlequin format**. This file contains the *CYTB *sequences from each of the four regions and additional sites (in non-interleaved Arlequin format). Click here for file

Additional file 4 ***RISP *sequences in Nexus format**. This file contains the *RISP *sequences in nexus file format (interleaved sequences). Click here for file

Additional file 5 **Table S3**. Pairwise percent sequence divergence between selected haplotypes for *CYTB*. Click here for file

Additional file 6 **Table S4**. F_ST _values for *CYTB *from Arlequin based on pairwise comparisons of populations (pairwise sequence divergence). Click here for file

Additional file 7 **Table S5**. Measures of the departures of the frequency spectra within regions and sites from neutral expectations. Click here for file

Additional file 8 **Table S6**. F_ST _values for *RISP *from Arlequin based on pairwise comparisons of populations (pairwise sequence divergence). Click here for file

Additional file 9 **Table S7**. *RISP *nucleotide and indel variation found in Santa Cruz region and PES for *T. californicus*. Click here for file

Additional file 10 **Table S8**. *RISP *nucleotide and indel variation found in La Jolla region for *T. californicus*. Click here for file

Additional file 11 **Table S9**. *RISP *nucleotide and indel variation found in Palos Verdes region for *T. californicus*. Click here for file
