# Supplementary material for: Investigations of fine-scale phylogeography in Tigriopus californicus reveal historical patterns of population divergence
Source: BMC Evol Biol. 2009 Jun 23;9:139. doi: 10.1186/1471-2148-9-139 (PMC2708153; doi:10.1186/1471-2148-9-139)
Supplement: Additional file 1 — Table S1. Sampling locations for Tigriopus californicus in southern and central California. [file 1471-2148-9-139-S1.pdf]

**Supplemental Table S1. Sampling locations for *Tigriopus californicus* in southern and central California.**

| Region | Site | Location | Collection date | Distance (km)[1] | Description/notes[2] |
|---|---|---|---|---|---|
| West Vancouver | BC | N49˚20'17.2" W123˚15'0.7" | 7/06 | 172.9 | Edge of extensive rocky habitat, scattered pools |
| Vancouver Island | VI | N48˚56'0.7" W125˚32'28.6" | 7/06 | 1199 | Extensive rocky habitat nearby, several dense pools |
| Bodega Head | BSB | N38˚18'18.4" W123˚03'55.5" | 9/04 | 2.46 | Extensive, nearly continuous rocky habitat, many pools |
| | BHAR | N38˚18'16.4" W123˚03'22.2" | 9/04 | 129.1 | Limited rock shelf in harbor, with few pools |
| Pescadero | PES | N37˚15'34.9" W122˚24'50.8" | 9/04 | 46.97 | Site sampled on numerous previous occasions |
| Santa Cruz | NB1 | N36˚56'57.5" W122˚03'40.7" | 9/04 | 0.184 | Numerous pools in large outcrop |
| | NB2 | N36˚57'00.2" W122˚03'34.5" | 9/04 | 0.641 | Numerous pools in large outcrop, 20m beach between NB1 and NB2, copepods sampled on other occasions |
| | BH | N36˚56'57.1" W122˚03'14.5" | 9/04 | 0.877 | One isolated pool with copepods on high shelf |
| | SCN | N36˚56'58.3" W122˚02'49.1" | 2002, 9/04 | 0.205 | Numerous pools in large outcrop, copepod pools present at all of many previous visits |
| | SC2 | N36˚56'59.5" W122˚02'43.4" | 9/04 | 0.129 | Moderate numbers pools in medium-sized outcrop, small sandy stretch separates from SCN |
| | SC3 | N36˚57'01.5" W122˚02'39.8" | 9/04 | 0.185 | Moderate numbers pools in medium-sized outcrop |
| | SC4 | N36˚57'04.5" W122˚02'34.3" | 9/04 | 0.576 | Scattered pools in medium-sized rocky ledge |
| | SC5 | N36˚57'10.3" W122˚02'16.3" | 9/04 | 1.18 | Few pools in small-sized outcrop |
| | LH | N36˚57'03.8" | 9/04 | 5.30 | Moderate number of pools, high shelf above ocean, |

[1] This is the distance between the site and the one below it and is the straight-line distance between sites in different regions or the coastal distance between sites within a region.
[2] Description notes the abundance of pools containing copepods on the sampled date (and in some cases previous observations) and may not reflect long-term available copepod habitat.

| | | | | | |
|---|---|---|---|---|---|
| | | W122˚01'36.8" | | | observed absence of copepods from 9/79-6/80[3] |
| | CCR1 | N36˚57'34.6" W121˚59'24.4" | 9/04 | 0.427 | Few pools in high rocky ledge |
| | CCR2 | N36˚57'36.7" W121˚59'19.1" | 9/04 | 171.3 | Few pools in high rocky ledge, 50m of beach separates from CCR1 |
| San Simeon | SS | N35˚34'53.9" W121˚07'16.2" | 9/04 | 275.3 | Extensive but discontinuous rocky habitat |
| Point Dume | Dume | N34˚00'01.0" W118˚48'24.3" | 9/04 | 43.30 | Note: may not be same as PD site previously sampled from this region |
| Palos Verde | FR1 | N33˚47'49.6" W118˚24'29.8" | 9/04 | 0.333 | Moderate numbers pools in medium-sized outcrop, copepods sampled on numerous occasions |
| | FR2 | N33˚47'41.8" W118˚24'27.8" | 9/04 | 4.74 | Few pools in small-sized outcrop, boulders to FR1 |
| | RsPt | N33˚45'58.8" W118˚25'28.3" | 9/04 | 4.08 | Small number of inhabited pools in medium-sized outcrop |
| | PVL | N33˚44'28.8" W118˚24'27.2" | 9/04 | 3.45 | Small pools in large boulders, limited habitat in area |
| | ABR | N33˚44'26.6" W118˚22'39.5" | 9/04 | 0.672 | Small pools in one large boulder, no other habitat until AB currently available |
| | AB | N33˚44'15.9" W118˚22'31.0" | 2002 | 0.032 | Few pools in small outcrop, separated from AB2 by narrow surge channel, copepods sampled on numerous occasions |
| | AB2 | N33˚44'14.9" W118˚22'30.6" | 2002, 9/04 | 0.146 | Numerous pools in large outcrop |
| | AB3 | N33˚44'12.9" W118˚22'25.9" | 9/04 | 0.697 | Numerous pools in large outcrop, separated from AB2 by narrow surge channel |
| | IP | N33˚44'10.6" W118˚22'10.5" | 9/04 | 6.11 | Numerous pools in large outcrop, beach separates from AB3 |
| | RP2 | N33˚42'56.5" W118˚19'12.0" | 9/04 | 0.338 | Flat rocky outcrop with moderate pool numbers, copepods sampled on numerous occasions |
| | RP1 | N33˚42'50.1" W118˚19'03.2" | 9/04 | 33.58/ 51.73[4] | Medium-sized outcrop outcrop with moderate pool numbers |

[3] Burton and Feldman, 1981
[4] Distance from RP1 to CAT/LagBch

| | | | | | |
|---|---|---|---|---|---|
| Catalina Island | CAT | N33˚26.8’ W118˚28.6’ | 6/06 | 64.58/ 68.47[5] | |
| Laguna Beach | LagBch | N33˚32’41.4” W117˚47’58.4” | 9/04 | 90.48 | Note: samples from S. Crescent Bay and not previously sampled LB site from this region |
| La Jolla | SIO | N32˚52’15.5” W117 15’11.4” | 11/02 | 3.62 | Scattered pools in boulders, limited numbers of pools, extensive beach to LJP1 |
| | LJP1 | N32˚51’04.5” W117˚16’24.3” | 11/02, 9/04 | 0.326 | Large outcrop with a moderate number of pools, copepods sampled on several previous occasions |
| | LJP2 | N32˚50’58.2” W117˚16’33.0” | 9/04 | 0.588 | Small outcrop with small number of pools, rocks and beach to LJP1 |
| | LJP3 | N32˚50’46.3” W117˚16’44.2” | 9/04 | 0.435 | Moderate-sized outcrop with limited numbers of inhabited pools, beach separates from LJP2 and LJS |
| | LJS | N32˚50’36.1” W117˚16’50.9” | 11/02 | 1.30 | Numerous pools in large, flat, expansive outcrop |
| | NAUT | N32˚49’56.9” W117˚16’55.6” | 9/04 | 2.63 | Moderate-sized outcrop with limited numbers of inhabited pools |
| | BR | N32˚48’54.0” W117˚16 23.1” | 11/02 | 7.89 | Isolated large rock platform outcrop |
| Point Loma | SD | N32˚44’44.4” W117˚15’18.0” | 2002 | 1.41 | Large outcrop with a large number of pools, copepod pools present at all of many previous visits |
| | SCL | N32˚43’59.0” W117˚15’25.3” | 9/04 | 1.48 | Moderated-sized ledge with a few large pools |
| | LS | N32˚43’11.2” W117˚15’25.1” | 11/02 | | Small outcrop with shallow pools in boulders |

[5] Distance from CAT to LagBch/Dume
