# Supplementary material for: Investigations of fine-scale phylogeography in Tigriopus californicus reveal historical patterns of population divergence
Source: BMC Evol Biol. 2009 Jun 23;9:139. doi: 10.1186/1471-2148-9-139 (PMC2708153; doi:10.1186/1471-2148-9-139)
Supplement: Additional file 2 — Table S2. Primers used for PCR amplifications for the CYTB and RISP genes from T. californicus. [file 1471-2148-9-139-S2.pdf]

Supplemental Table S2 Primers used for PCR amplifications for the *CYTB* and *RISP* genes from *T. californicus.* Annealing temperatures typically used in the PCR reactions are given as well. The first set of primers were designed to match *CYTB* sequences from each of the four regions, but in some cases, primers specific to a single region were needed to get robust amplifications. Additional details of PCR and sequencing are available upon request from C.S.W..

Gene/Populations	Forward primer	Sequence (5' to 3')	Reverse primer	Sequence (5' to 3')	Annealing temperature
CYTB					
Most populations	COIIdeg.f	TGYGGGGCKAAYCATAGKTTTATACC	ND4deg.r	ACGYTCKGGYTGRTAMCCYCAACC	45˚C
Palos Verdes region, CAT, BC, VI, SS	Cytbf2.AB	TTAAAATTATTGCTCTGGGCAAT	CytbR2in.AB	TTAGGATACCACAAACCCTTGT	52˚C
La Jolla region	Cytbstart.f.LJ	TAATTGCTTTGGGCAGTTTCTA	Cytbstop.r.LJ	ACCTAGGATACCACAAACCCTTA	45˚C
Santa Cruz region	COIIdeg.f	See above	CytbR5.SC	TGCTATAACAAGGCCGAAACTTGC	58˚C
RISP					
All populations	RISPstart.f	CTTGGACTGGTGAACAAGATG	RISPstop.r	TTATCCCACAATGACCATGCCTTC	54˚C
