# Supplementary material for: Investigations of fine-scale phylogeography in Tigriopus californicus reveal historical patterns of population divergence
Source: BMC Evol Biol. 2009 Jun 23;9:139. doi: 10.1186/1471-2148-9-139 (PMC2708153; doi:10.1186/1471-2148-9-139)
Supplement: Additional file 3 — CYTB sequences in Arlequin format. This file contains the CYTB sequences from each of the four regions and additional sites (in non-interleaved Arlequin format). [file 1471-2148-9-139-S3.doc]

#================================

# CYTB sequences from each of the four regions (in non-interleaved Arlequin format).

#Arlequin format CYTB sequences: samples are sites, with haplotype name followed by number of haplotypes of that sequence at that site.

#================================

[Profile]

Title="CYTB_TCAL_all"

NbSamples=41

DataType=DNA

GenotypicData=0

GameticPhase=1

LocusSeparator=NONE

RecessiveData=0

MissingData='?'

[Data]

[[Samples]]

SampleName="SD 1"

SampleSize= 20

SampleData= {

SD5 14 ATTTATAAGGCGTCTCGATTTAGACCATTAACTAGGATTATTCGGCACACGGTAATCACTTTGCCCACTCCTATTAATATTTCAACGTGGTGAAATTTTGGTTCCCTTCTGGGAATTTGTTTGGCGAGCCAGATTTTGAGAGGATTAATTCTAAGGCTTCACTTCACTGCTCATACGGATCTCAGATTTTATGTGGTGATTCAAAGATTTGGGGATGTTTACTATAATTGGTGGTTTCGTGCTCTCCACGCTAATGGGGCCAGTTTCTTTTTTATTGGGCTTTATTTACATATTGCTCGGGGTCTCTATTATGGATCCTACGTTTTCAAGGAAGTTTGGTCCATTGGAGTTGTGATCTTACTTTTGGTAATAGCAAGGGCCTTCTTAGGCTATGTCCTTCCGTGGGGACAGATATCTTTTTGGGGGGCAACGGTTATTACTAGACTATTTTCGGCACTCCCAGTTGTTGGGGGAGATGTGGTGGTTTGATTGTGAGGGGGCTTCTCGGTGGATAATGCTACCTTGACTCGGTTTTTTGGGCTTCATTTCGTGTTGCCCTTCATTGTGGCGGGGCTAGTTGGGCTTCACCTGTTCTTCTTACACGACCAAGGGTCGTCAAATCCTTTAGGGGTGAGATCAGATTTTAATAAGGTTGTTTTTCATCATTATTATGTGCTTAAGGATTTAGTGGGGGCTTGTTTTTTCATTGGCCCCTTACTTGGACTGGTTTACTTTAACCCTTGGCTTCTAGGAGATCCTGAAAATTTCATTGAGGGGAATCCCTTAGTCACACCACATCATATCCAACCTGAATGATATTTTTTATTTGCCTATGCCATTTTGCGGGCCGTACCTAACAAGTTAGGAGGAGTCGTAGCGTTGGCTGCTGCGGTCTTGGTACTGTTTTATATACCAGTAAGACCTAAAACTTCTGTCAAAGGGTTATCTTTTTACCCATGAGGAAAAATTTTGTTTTTTAGGTTGATTGGGGTGTTTTTTCTGCTTACATGGGCAGGGTCTAAGCCGGTAGAAGAACCATATGTGGTGATTAGACAGGTTTTAAGTTTCTTGTATTTTTCCTATTTTGCCTTAATTGGACCCATCACTCGTATTCAAGATAAGTTGTTGTAA

SDl 1 ATTTATAAGGCGTCTCGATTTAGACCATTAACTAGGATTATTCGGCACACGGTAATCACTTTGCCCACTCCTATTAATATTTCAACGTGGTGAAATTTTGGTTCCCTTCTGGGAATTTGTTTGGCGAGCCAGATTTTGAGAGGATTAATTCTAAGGCTTCACTTCACTGCTCATACGGATCTCAGATTTTATGTGGTGATTCAAAGATTTGGGGATGTTTACTATAATTGGTGGTTTCGTGCTCTCCACGCTAATGGGGCCAGTTTCTTTTTTATTGGGCTTTATTTACACATTGCTCGGGGTCTCTATTATGGATCCTACGTTTTCAAGGAAGTTTGGTCCATTGGAGTTGTGATCTTACTTTTGGTAATAGCAAGGGCCTTCTTAGGCTATGTCCTTCCGTGGGGACAGATATCTTTTTGGGGGGCAACGGTTATTACTAGACTATTTTCGGCACTCCCAGTTGTTGGGGGAGATGTGGTGGTTTGATTGTGAGGGGGCTTCTCGGTGGATAATGCTACCTTGACTCGGTTTTTTGGGCTTCATTTCGTGTTGCCCTTCATTGTGGCGGGGCTAGTTGGGCTTCACCTGTTCTTCTTACACGACCAAGGGTCGTCAAATCCTTTAGGGGTGAGATCAGATTTTAATAAGGTTGTTTTTCATCATTATTATGTGCTTAAGGATTTAGTGGGGGCTTGTTTTTTCATTGGCCCCTTACTTGGACTGGTTTACTTTAACCCTTGGCTTCTAGGAGATCCTGAAAATTTCATTGAGGGGAATCCCTTAGTCACACCACACCATATCCAACCTGAATGATATTTTTTATTTGCCTATGCCATTTTGCGGGCCGTACCTAACAAGTTAGGAGGAGTCGTAGCGTTGGCTGCTGCGGTCTTGGTACTGTTTTACATACCAGTAAGACCTAAAACTTCTGTCAAAGGGTTATCTTTTTACCCATGAGGAAAAATTTTGTTTTTTAGGTTGATTGGGGTGTTTTTTCTGCTTACATGGGCAGGGTCTAAGCCGGTAGAAGAACCATATGTGGTGATTAGACAGGTTTTAAGTTTCTTGTATTTTTCCTATTTTGCCTTAATTGGACCCATCACTCGTATTCAAGATAAGTTGTTGTAA

SDm4 1 ATTTATAAGGCGTCTCGATTTAGACCATTAACTAGGATTATTCGGCACACGGTAATCACTTTGCCCACTCCTATCAATATTTCAACGTGGTGAAATTTTGGTTCCCTTCTGGGAATTTGTTTGGCGAGCCAGATTTTGAGAGGATTAATTCTAAGGCTTCACTTCACTGCTCATACGGATCTCAGATTTTATGTGGTGATTCAAAGATTTGGAGATGTTTACTATAATTGGTGGTTTCGTGCTCTCCACGCTAATGGGGCCAGTTTCTTTTTTATTGGGCTTTATTTACATATTGCTCGGGGTCTCTATTATGGATCCTACGTTTTCAAGGAAGTTTGGTCCATTGGAGTTGTGATCTTACTTTTGGTAATAGCAAGGGCCTTCTTAGGCTATGTCCTTCCGTGGGGACAGATATCTTTTTGGGGGGCAACGGTTATTACTAGCCTATTTTCGGCACTCCCAGTTGTTGGGGGGGATGTGGTGGTTTGATTGTGAGGGGGCTTCTCGGTGGATAATGCTACCTTGACTCGGTTTTTTGGGCTTCATTTCGTGTTGCCCTTCATTGTGGCGGGGCTAGTTGGGCTTCACCTGTTCTTCTTACACGACCAAGGGTCGTCAAATCCTTTAGGGGTGAGATCAGATTTTAATAAGGTTGTTTTTCATCATTATTATGTGCTTAAAGATTTAGTGGGGGCTTGTTTTTTCATTGGCCCTTTACTTGGACTGGTTTACTTTAACCCTTGGCTTCTAGGAGATCCTGAAAATTTCATTGAGGGGAATCCCTTAGTCACACCACATCATATCCAACCTGAATGATATTTTTTATTTGCCTATGCCATTTTGCGGGCCGTACCTAACAAGTTAGGAGGAGTCGTAGCGTTGGCTGCTGCGGTCTTGGTACTGTTTTATATACCAGTAAGACCTAAAACTTCTGTCAAAGGGTTATCTTTTTACCCATGAGGAAAAATTTTGTTTTTTAGGTTGATTGGGGTGTTTTTTCTGCTTACATGGGCAGGGTCTAAGCCGGTAGAAGAACCATATGTGGTGATTAGACAGGTTTTAAGTTTCTTGTATTTTTCCTATTTTGCCTTAATTGGACCCATCACTCGTATTCAAGATAAGTTGTTGTAA

SDm1 1 ATTTATAAGGCGTCTCGATTTAGACCATTAACTAGGATTATTCGGCACACGGTAATCACTTTGCCCACTCCTATTAATATTTCAACGTGGTGAAATTTTGGTTCCCTTCTGGGAATTTGTTTGGCGAGCCAGATTTTGAGAGGATTAATTCTAAGGCTTCACTTCACTGCTCATACGGATCTCAGATTTTATGTGGTGATTCAAAGATTTGGGGATGTTTACTATAATTGGTGGTTTCGTGCTCTCCACGCTAATGGGGCCAGTTTCTTTTTTATTGGGCTTTATTTACATATTGCTCGGGGTCTCTATTATGGATCCTACGTTTTCAAGGAAGTTTGGTCCATTGGAGTTGTGATCTTACTTTTGGTAATAGCAAGGGCCTTCTTAGGCTATGTCCTTCCGTGGGGACAGATATCTTTTTGGGGGGCAACGGTTATTACTAGCCTATTTTCGGCACTCCCAGTTGTTGGGGGGGATGTGGTGGTTTGATTGTGAGGGGGCTTCTCGGTGGATAATGCTACCTTGACTCGGTTTTTTGGGCTTCATTTCGTGTTGCCCTTCATTGTGGCGGGGCTAGTTGGGCTTCACCTGTTCTTCCTACACGACCAAGGGTCGTCAAATCCTTTAGGGGTGAGATCAGATTTTAATAAGGTTGTTTTTCATCATTATTATGTGCTTAAGGATTTAGTGGGGGCTTGTTTTTTCATTGGCCCTTTACTTGGACTGGTTTACTTTAACCCTTGGCTTCTAGGAGATCCTGAAAATTTCATTGAGGGGAATCCCTTAGTCACACCACATCATATCCAACCTGAATGATATTTTTTATTTGCCTATGCCATTTTGCGGGCCGTACCTAACAAGTTAGGAGGAGTCGTAGCGTTGGCTGCTGCGGTCTTGGTACTGTTTTATATACCAGTAAGACCTAAAACTTCTGTCAAAGGGTTATCTTTTTACCCATGAGGAAAAATTTTGTTTTTTAGGTTGATTGGGGTGTTTTTTCTGCTTACATGGGCAGGGTCTAAGCCGGTAGAAGAACCATATGTGGTGATTAGACAGGTTTTAAGTTTCTTGTATTTTTCCTATTTTGCCTTAATTGGACCCATCACTCGTATTCAAGATAAGTTGTTGTAA

SDm7 1 ATTTATAAGGCGTCTCGATTTAGACCATTAACTAGGATTATTCGGCACACGGTAATCACTTTGCCCACTCCTATTAATATTTCAACGTGGTGAAATTTTGGTTCCCTTCTGGGAATTTGTTTGGCGAGCCAGATTTTGAGAGGATTAATTCTAAGGCTTCACTTCACTGCTCATACGGATCTCAGATTTTATGTGGTGATTCAAAGATTTGGGGATGTTTACTATAATTGGTGGTTTCGTGCTCTCCACGCTAATGGGGCCAGTTTCTTTTTTATTGGGCTTTATTTACATATTGCTCGGGGTCTCTATTATGGATCCTACGTTTTCAAGGAAGTTTGGTCCATTGGAGTTGTGATCTTACTTTTGGTAATAGCAAGGGCCTTCTTAGGCTATGTCCTTCCGTGGGGACAGATATCTTTTTGGGGGGCAACGGTTATTACTAGCCTATTTTCGGCACTCCCAGTTGTTGGAGGGGATGTGGTGGTTTGATTGTGAGGGGGCTTCTCGGTGGATAATGCTACCTTGACTCGGTTTTTTGGGCTTCATTTCGTGTTGCCCTTCATTGTGGCGGGGCTAGTTGGGCTTCACCTGTTCTTCTTACACGACCAAGGGTCGTCAAATCCTTTAGGGGTGAGATCAGATTTTAATAAGGTTGTTTTTCATCATTATTATGTGCTTAAGGATTTAGTGGGGGCTTGTTTTTTCATTGGCCCTTTACTTGGACTGGTTTACTTTAACCCTTGGCTTCTAGGAGATCCTGAAAATTTCATTGAGGGGAATCCCTTAGTCACACCACATCATATCCAACCTGAATGATATTTTTTATTTGCCTATGCCATTTTGCGGGCCGTACCTAACAAGTTAGGAGGAGTCGTAGCGTTGGCTGCTGCGGTCTTGGTACTGTTTTATATACCAGTAAGACCTAAAACTTCTGTCAAAGGGTTATCTTTTTACCCATGAGGAAAAATTTTGTTTTTTAGGTTGATTGGGGTGTTTTTTCTGCTTACATGGGCAGGGTCTAAGCCGGTAGAAGAACCATATGTGGTGATTAGACAGGTTTTAAGTTTCTTGTATTTTTCCTATTTTGCCTTAATTGGACCCATCACTCGTATTCAAGATAAGTTGTTGTAA

SDh 1 ATTTATAAGGCGTCTCGATTTAGACCATTAACTAGGATTATTCGGCACACGGTAATCACTTTGCCCACTCCTATTAATATTTCAACGTGGTGAAATTTTGGTTCCCTTCTGGGAATTTGTTTGGCGAGCCAGATTTTGAGAGGGTTAATTCTAAGGCTTCACTTCACTGCTCATACGGATCTCAGATTTTATGTGGTGATTCAAAGATTTGGGGATGTTTACTATAATTGGTGGTTTCGTGCTCTCCACGCTAATGGGGCCAGTTTCTTTTTTATTGGGCTTTATTTACATATTGCTCGGGGTCTCTATTATGGATCCTACGTTTTCAAGGAAGTTTGGTCCATTGGAGTTGTGATCTTACTTTTGGTAATAGCAAGGGCCTTCTTAGGCTATGTCCTTCCGTGGGGACAGATATCTTTTTGGGGGGCAACGGTTATTACTAGCCTATTTTCGGCACTCCCAGTTGTTGGGGGGGATGTGGTGGTTTGATTGTGAGGGGGCTTCTCGGTGGATAATGCTACCTTGACTCGGTTTTTTGGGCTTCATTTCGTGTTGCCCTTCATTGTGGCGGGGCTAGTTGGGCTTCATCTGTTCTTCTTACACGACCAAGGGTCGTCAAATCCTTTAGGGGTGAGATCAGATTTTAATAAGGTTGTTTTTCATCATTATTATGTGCTTAAGGATTTAGTGGGGGCTTGTTTTTTCATTGGCCCTTTACTTGGACTGGTTTACTTTAACCCTTGGCTTCTAGGAGATCCTGAAAATTTCATTGAGGGGAATCCCTTAGTCACACCACATCATATCCAACCTGAATGATATTTTTTATTTGCCTATGCCATTTTGCGGGCCGTACCTAACAAGTTAGGAGGAGTCGTAGCGTTGGCTGCTGCGGTCTTGGTACTGTTTTATATACCAGTAAGACCTAAAACTTCTGTCAAAGGGTTATCTTTTTACCCATGAGGAAAAATTTTGTTTTTTAGGTTGATTGGGGTGTTTTTTCTGCTTACATGGGCAGGGTCTAAGCCGGTAGAAGAACCATATGTGGTGATTAGACAGGTTTTAAGTTTCTTGTATTTTTCCTATTTTGCCTTAATTGGACCCATCACTCGTATTCAAGATAAGTTGTTGTAA

SD4 1 ATTTATAAGGCGTCTCGATTTAGACCATTAACTAGGATTATTCGGCACACGGTAATCACTTTGCCCACTCCTATTAATATTTCAACGTGGTGAAATTTTGGTTCCCTTCTGGGAATTTGTTTGGCGAGCCAGATTTTGAGAGGATTAATTCTAAGGCTTCACTTCACTGCTCATACGGATCTCAGATTTTATGTGGTGATTCAAAGATTTGGGGATGTTTACTATAATTGGTGGTTTCGTGCTCTCCACGCTAATGGGGCCAGTTTCTTTTTTATTGGGCTTTATTTACATATTGCTCGGGGTCTCTATTATGGATCCTACGTTTTCAAGGAAGTTTGGTCCATTGGAGTTGTGATCTTACTTTTGGTAATAGCAAGGGCCTTCTTAGGCTATGTCCTTCCGTGGGGACAGATATCTTTTTGGGGGGCAACGGTTATTACTAGCCTATTTTCGGCACTCCCAGTTGTTGGGGGGGATGTGGTGGTTTGATTGTGAGGGGGCTTCTCGGTGGATAATGCTACCTTGACTCGGTTTTTTGGGCTTCATTTCGTGTTGCCCTTCATTGTGGCGGGGCTAGTTGGGCTTCACCTGTTCTTCTTACACGACCAAGGGTCGTCAAATCCTTTAGGGGTGAGATCAGATTTTAATAAGGTTGTTTTTCATCATTATTATGTGCTTAAGGATTTAGTGGGGGCTTGTTTTTTCATTGGCCCTTTACTTGGACTGGTTTACTTTAACCCTTGGCTTCTAGGAGATCCTGAAAATTTCATTGAGGGGAATCCCTTAGTCACACCACATCATATCCAACCTGAATGATATTTTTTATTTGCCTATGCCATTTTGCGGGCCGTACCTAACAAGTTAGGAGGAGTCGTAGCGTTGGCTGCTGCGGTCTTGGTACTGTTTTATATACCAGTAAGACCTAAAACTTCTGTCAAAGGGTTATCTTTTTACCCATGAGGAAAAATTTTGTTTTTTAGGTTGATTGGGGTGTTTTTTCTGCTTACATGGGCAGGGTCTAAGCCGGTAGAAGAACCATATGTGGTGATTAGACAGGTTTTAAGTTTCTTGTATTTTTCCTATTTTGCCTTAATTGGACCCATCACTCGTATTCAAGATAAGTTGTTGTAA }

SampleName="SunCl 2"

SampleSize= 7

SampleData= {

SunCl_f6 1 ATTTATAAGGCGTCTCGATTTAGACCATTAACTAGGATTATTCGGCACACGGTAATCACTTTGCCCACTCCTATTAATATTTCAACGTGGTGAAATTTTGGTTCCCTTCTGGGAATTTGTTTGGCGAGCCAGATTTTGAGAGGATTAATTCTAAGGCTTCACTTCACTGCTCATACGGATCTCAGATTTTATGTGGTGATTCAAAGATTTGGGGATGTTTACTATAATTGGTGGTTTCGTGCTCTCCACGCTAATGGGGCCAGTTTCTTTTTTATTGGGCTTTATTTACATATTGCTCGGGGTCTCTATTATGGATCCTACGTTTTCAAGGAAGTTTGGTCCATTGGAGTTGTGATCTTACTTTTGGTAATAGCAAGGGCCTTCTTAGGCTATGTCCTTCCGTGGGGACAGATATCTTTTTGGGGGGCAACGGTTATTACTAGCCTATTTTCGGCACTCCCAGTTGTTGGGGGGGATGTGGTGGTTTGATTGTGAGGGGGCTTCTCGGTGGATAATGCTACCTTGACTCGGTTTTTTGGGCTTCATTTCGTGTTGCCCTTCATTGTGGCGGGGCTAGTTGGGCTTCACCTGTTCTTCTTACACGACCAAGGGTCGTCAAATCCTTTAGGGGTGAGATCAGATTTTAATAAGGTTGTTTTTCATCATTATTATGTGCTTAAGGATTTAGTGGGGGCTTGTTTTTTCATTGGCCCCTTACTTGGACTGGTTTACTTTAACCCTTGGCTTCTAGGAGATCCTGAAAATTTCATTGAGGGGAATCCCTTAGTCACACCACATCATATCCAACCTGAATGATATTTTTTATTTGCCTATGCCATTTTGCGGGCCGTACCTAACAAGTTAGGAGGAGTCGTAGCGTTGGCTGCTGCGGTCTTGGTACTGTTTTATATACCAGTAAGACCTAAAACTTCTGTCAAAGGGTTATCTTTTTACCCATGAGGAAAAATTTTGTTTTTTAGGTTGATTGGGGTGTTTTTTCTGCTTACATGGGCAGGGTCTAAGCCGGTAGAAGAACCATATGTGGTGATTAGACAGGTTTTAAGTTTCTTGTATTTTTCCTATTTTGCCTTAATTGGACCCATCACTCGCATTCAAGATAAGTTGTTGTAA

SunCl_f2 5 ATTTATAAGGCGTCTCGATTTAGACCATTAACTAGGATTATTCGGCACACGGTAATCACTTTGCCCACTCCTATTAATATTTCAACGTGGTGAAATTTTGGTTCCCTTCTGGGAATTTGTTTGGCGAGCCAGATTTTGAGAGGATTAATTCTAAGGCTTCACTTCACTGCTCATACGGATCTCAGATTTTATGTGGTGATTCAAAGATTTGGGGATGTTTACTATAATTGGTGGTTTCGTGCTCTCCACGCTAATGGGGCCAGTTTCTTTTTTATTGGGCTTTATTTACATATTGCTCGGGGTCTCTATTATGGATCCTACGTTTTCAAGGAAGTTTGGTCCATTGGAGTTGTGATCTTACTTTTGGTAATAGCAAGGGCCTTCTTAGGCTATGTCCTTCCGTGGGGACAGATATCTTTTTGGGGGGCAACGGTTATTACTAGCCTATTTTCGGCACTCCCAGTTGTTGGGGGGGATGTGGTGGTTTGATTGTGAGGGGGCTTCTCGGTGGATAATGCTACCTTGACTCGGTTTTTTGGGCTTCATTTCGTGTTGCCCTTCATTGTGGCGGGGCTAGTTGGGCTTCACCTGTTCTTCTTACACGACCAAGGGTCGTCAAATCCTTTAGGGGTGAGATCAGATTTTAATAAGGTTGTTTTTCATCATTATTATGTGCTTAAGGATTTAGTGGGGGCTTGTTTTTTCATTGGCCCCTTACTTGGACTGGTTTACTTTAACCCTTGGCTTCTAGGAGATCCTGAAAACTTCATTGAGGGGAATCCCTTAGTCACACCACATCATATCCAACCTGAATGATATTTTTTATTTGCCTATGCCATTTTGCGGGCCGTACCTAACAAGTTAGGAGGAGTCGTAGCGTTGGCTGCTGCGGTCTTGGTACTGTTTTATATACCAGTAAGACCTAAAACTTCTGTCAAAGGGTTATCTTTTTACCCATGAGGAAAAATTTTGTTTTTTAGGTTGATTGGGGTGTTTTTTCTGCTTACATGGGCAGGGTCTAAGCCGGTAGAAGAACCATATGTGGTGATTAGACAGGTTTTAAGTTTCTTGTATTTTTCCTATTTTGCCTTAATTGGACCCATCACTCGTATTCAAGATAAGTTGTTGTAA

SCL_f4 1 ATTTATAAGGCGTCTCGATTTAGACCATTAACTAGGAATATTCGGCACACGGTAATCACTTTGCCCACTCCTATTAATATTTCAACGTGGTGAAATTTTGGTTCCCTTCTGGGAATTTGTTTGGCGAGCCAGATTTTGAGAGGATTAATTCTAAGGCTTCACTTCACTGCTCATACGGATCTCAGATTTTATGTGGTGATTCAAAGATTTGGGGATGTTTACTATAATTGGTGGTTTCGTGCTCTCCACGCTAATGGGGCCAGTTTCTTTTTTATTGGGCTTTATTTACATATTGCTCGGGGTCTCTATTATGGATCCTACGTTTTCAAGGAAGTTTGGTCCATTGGAGTTGTGATCTTACTTTTGGTAATAGCAAGGGCCTTCTTAGGCTATGTCCTTCCGTGGGGACAGATATCTTTTTGGGGGGCAACGGTTATTACTAGCCTATTTTCGGCACTCCCAGTTGTTGGGGGGGATGTGGTGGTTTGATTGTGAGGGGGCTTCTCGGTGGATAATGCTACCTTGACTCGGTTTTTTGGGCTTCATTTCGTGTTGCCCTTCATTGTGGCGGGGCTAGTTGGGCTTCACCTGTTCTTCTTACACGACCAAGGGTCGTCAAATCCTTTAGGGGTGAGATCAGATTTTAATAAGGTTGTTTTTCATCATTATTATGTGCTTAAGGATTTAGTGGGGGCTTGTTTTTTCATTGGCCCCTTACTTGGACTGGTTTACTTTAACCCTTGGCTTCTAGGAGATCCTGAAAACTTCATTGAGGGGAATCCCTTAGTCACACCACATCATATCCAACCTGAATGATATTTTTTATTTGCCTATGCCATTTTGCGGGCCGTACCTAACAAGTTAGGAGGAGTCGTAGCGTTGGCTGCTGCGGTCTTGGTACTGTTTTATATACCAGTAAGACCTAAAACTTCTGTCAAAGGGTTATCTTTTTACCCATGAGGAAAAATTTTGTTTTTTAGGTTGATTGGGGTGTTTTTTCTGCTTACATGGGCAGGGTCTAAGCCGGTAGAAGAACCATATGTGGTGATTAGACAGGTTTTAAGTTTCTTGTATTTTTCCTATTTTGCCTTAATTGGACCCATCACTCGTATTCAAGATAAGTTGTTGTAA }

SampleName="LS 3"

SampleSize= 8

SampleData= {

LS_7f 1 ATTTATAAGGCGTCTCGATTTAGACCATTAACTAGGATTATTCGGCACACGGTAATCACTTTGCCCACTCCTATTAATATTTCAACGTGGTGAAATTTTGGTTCCCTTCTGGGAATTTGTTTGGCGAGCCAGATTTTGAGAGGATTAATTCTAAGGCTTCACTTCACTGCTCATACGGATCTCAGATTTTATGTGGTGATTCAAAGATTTGGGGATGTTTACTATAATTGGTGGTTTCGTGCTCTCCACGCTAATGGGGCCAGTTTCTTTTTTATTGGGCTTTATTTACATATTGCTCGGGGTCTCTATTATGGATCCTACGTTTTCAAGGAAGTTTGGTCCATTGGAGTTGTGATCTTACTTTTGGTAATAGCAAGGGCCTTCTTAGGCTATGTCCTTCCGTGGGGACAGATATCTTTTTGGGGGGCAACGGTTATTACTAGCCTATTTTCGGCACTCCCAGTTGTTGGGGGGGATGTGGTGGTTTGATTGTGAGGGGGTTTCTCGGTGGATAATGCTACCTTGACTCGGTTTTTTGGGCTTCATTTCGTGTTGCCCTTCATTGTGGCGGGGCTAGTTGGGCTTCACCTGTTCTTCTTACACGACCAAGGGTCGTCAAATCCTTTAGGGGTGAGATCAGATTTTAATAAGGTTGTTTTTCATCATTATTATGTGCTTAAGGATTTAGTGGGGGCTTGTTTTTTCATTGGCCCCTTACTTGGACTGGTTTACTTTAACCCTTGGCTTCTAGGAGATCCTGAAAATTTCATTGAGGGGAATCCCTTAGTCACACCACATCATATCCAACCTGAATGATATTTTTTATTTGCCTATGCCATTTTGCGGGCCGTACCTAACAAGTTAGGAGGAGTCGTAGCGTTGGCTGCTGCGGTCTTGGTACTGTTTTATATACCAGTAAGACCTAAAACTTCTGTCAAAGGGTTATCTTTTTACCCATGAGGAAAAATTTTGTTTTTTAGGTTGATTGGGGTGTTTTTTCTGCTTACATGGGCAGGGTCTAAGCCGGTAGAAGAACCATATGTGGTGATTAGACAGGTTTTAAGTTTCTTGTATTTTTCCTATTTTGCCTTAATTGGACCCATCACTCG?ATTCAAGATAAGTTGTTGTAA

LS_m1 7 ATTTATAAGGCGTCTCGATTTAGACCATTAACTAGGATTATTCGGCACACGGTAATCACTTTGCCCACTCCTATTAATATTTCAACGTGGTGAAATTTTGGTTCCCTTCTGGGAATTTGTTTGGCGAGCCAGATTTTGAGAGGATTAATTCTAAGGCTTCACTTCACTGCTCATACGGATCTCAGATTTTATGTGGTGATTCAAAGATTTGGGGATGTTTACTATAATTGGTGGTTTCGTGCTCTCCACGCTAATGGGGCCAGTTTCTTTTTTATTGGGCTTTATTTACATATTGCTCGGGGTCTCTATTATGGATCCTACGTTTTCAAGGAAGTTTGGTCCATTGGAGTTGTGATCTTACTTTTGGTAATAGCAAGGGCCTTCTTAGGCTATGTCCTTCCGTGGGGACAGATATCTTTTTGGGGGGCAACGGTTATTACTAGCCTATTTTCGGCACTCCCAGTTGTTGGGGGGGATGTGGTGGTTTGATTGTGAGGGGGCTTCTCGGTGGATAATGCTACCTTGACTCGGTTTTTTGGGCTTCATTTCGTGTTGCCCTTCATTGTGGCGGGGCTAGTTGGGCTTCACCTGTTCTTCTTACACGACCAAGGGTCGTCAAATCCTTTAGGGGTGAGATCAGATTTTAATAAGGTTGTTTTTCATCATTATTATGTGCTTAAGGATTTAGTGGGGGCTTGTTTTTTCATTGGCCCCTTACTTGGACTGGTTTACTTTAACCCTTGGCTTCTAGGAGATCCTGAAAATTTCATTGAGGGGAATCCCTTAGTCACACCACATCATATCCAACCTGAATGATATTTTTTATTTGCCTATGCCATTTTGCGGGCCGTACCTAACAAGTTAGGAGGAGTCGTAGCGTTGGCTGCTGCGGTCTTGGTACTGTTTTATATACCAGTAAGACCTAAAACTTCTGTCAAAGGGTTATCTTTTTACCCATGAGGAAAAATTTTGTTTTTTAGGTTGATTGGGGTGTTTTTTCTGCTTACATGGGCAGGGTCTAAGCCGGTAGAAGAACCATATGTGGTGATTAGACAGGTTTTAAGTTTCTTGTATTTTTCCTATTTTGCCTTAATTGGACCCATCACTCGTATTCAAGATAAGTTGTTGTAA }

SampleName="LJS 4"

SampleSize= 14

SampleData= {

LJS_m10 1 ATTTATAAGGCTTCCCGATTTAGACCACTAACTAGGATTATTCGGCATACAGTAGTCACTTTGCCAACTCCTATTAACATTTCAACGTGGTGAAATTTTGGTTCCCTCCTGGGAATTTGTTTGGCGAGTCAGATTTTTAGAGGGTTGGTTCTGAGACTCCACTTTACTGCTCATACCGATCTTAGTTTTTATGTAGTCATTCAAAGATTTGGAGATGTTTACTACAACTGATGGTTCCGGGCTCTCCACGCTAATGGGGCTAGTTTCTTTTTTATTGGACTTTATTTGCATATTGCTCGAGGTCTCTATTACGGATCTTACCTTTTCAAAGAAGTTTGGTCCATTGGAGTTGTGATCTTGCTTTTGGTTATAGCAAGGGCCTTCTTGGGCTATGTCCTTCCATGGGGACAAATGTCTTTTTGAGGGGCAACAGTTATTACTAGTCTGTTTTCGGCACTCCCTGTTATTGGAGGAGATGTAGTAGTTTGATTGTGAGGGGGCTTCTCGGTAGACAATGCGACGCTAACTCGATTTTTTGGGCTTCATTTCGTATTACCTTTCATTGTGGCGGGGCTAGTAGGGCTCCACCTATTCTTCTTGCACGACCAAGGGTCGTCAAATCCTTTAGGGGTGAGGTCAGATTTTAATAAGGTTGTTTTTCACCACTATTATGTCCTTAAGGATTTAGTGGGGGCTTGTTTTTTTATTGGCCCCTTACTTGGATTAGTTTACTTTAATCCTTGGCTTCTAGGGGATCCCGAAAATTTTATTGAGGGGAACCCACTGGTTACACCACATCATATTCAACCTGAATGGTATTTTTTATTTGCTTATGCTATTTTGCGGGCTGTGCCTAACAAATTAGGAGGAGTAGTAGCGTTGGCCGCGGCGGTCTTGGTGTTGTTTTATATGCCAGTAAGACCTAAAACTTCTGTTAAAGGGCTATCTTTTTATCCGTGAGGAAAAATGTTATTTTTTAGGCTTGTTGGAGTGTTTTTTCTGCTTACTTGGGCAGGATCGAAGCCAGTAGAAGAACCATATGTAGTGATTAGACAGGTTTTAAGTTTCTTGTATTTTTCTTATTTTGCCTTAATTGGACCTATCACTCGTATTCAAGACAAGTTGTTGTAA

LJS_m1 1 ATTTATAAGGCTTCCCGATTTAGACCACTAACTAGGATTATTCGGCATACGGTAGTCACTTTGCCAACTCCTATTAACATTTCAACGTGGTGAAATTTTGGTTCCCTCCTGGGAATTTGTTTGGCGAGTCAGATTTTTAGAGGGTTGGTTCTGAGACTCCACTTTACTGCTCATACCGATCTTAGTTTTTATGTAGTCATTCAAAGATTTGGAGATGTTTACTACAACTGATGGTTCCGGGCTCTCCACGCTAATGGGGCTAGTTTCTTTTTTATTGGACTTTATTTGCATATTGCTCGAGGTCTCTATTACGGATCTTACCTTTTCAAAGAAGTTTGGTCCATTGGAGTTGTGATCTTGCTTTTAGTTATAGCAAGGGCCTTCTTGGGCTATGTCCTTCCATGGGGACAAATGTCTTTTTGAGGGGCAACAGTTATTACTAGTCTGTTTTCGGCACTCCCTGTTATTGGAGGAGATGTAGTAGTTTGATTGTGAGGGGGCTTCTCGGTAGACAATGCGACGCTAACTCGATTTTTTGGGCTTCATTTCGTATTACCTTTCATTGTGGCGGGGCTAGTAGGGCTCCACTTATTCTTCTTGCACGACCAAGGGTCGTCAAATCCTTTAGGGGTGAGGTCAGATTTTAATAAGGTTGTTTTTCACCACTATTATGTCCTTAAGGATTTAGTGGGGGCTTGTTTTTTTATTGGCCCCTTACTTGGATTAGTTTACTTTAATCCTTGGCTTCTAGGGGATCCCGAAAATTTTATTGAGGGGAACCCACTGGTTACACCACATCATATTCAACCTGAATGGTATTTTTTATTTGCTTATGCTATTTTGCGGGCTGTGCCTAACAAATTAGGAGGAGTAGTAGCGTTGGCCGCGGCGGTCTTGGTGTTGTTTTATATGCCAGTAAGACCTAAAACTTCTGTTAAAGGGCTATCTTTTTATCCGTGAGGAAAAATGTTATTTTTTAGGCTTGTTGGAGTGTTTTTTCTGCTTACTTGGGCAGGATCGAAGCCAGTAGAAGAACCATATGTAGTGATTAGACAGGTTTTAAGTTTCTTGTATTTTTCTTATTTTGCCTTAATTGGACCTATCACTCGTATTCAAGACAAGTTGTTGTAA

LJS_f2 1 ATTTATAAGGCTTCCCGATTTAGACCACTAACTAGGATTATTCGGCATACGGTAGTCACTTTGCCAACTCCTATTAACATTTCAACGTGGTGAAATTTTGGTTCCCTCCTGGGAATTTGTTTGGCGAGTCAGATTTTTAGAGGGTTGGTTCTGAGACTCCACTTTACTGCTCATACCGATCTTAGTTTTTATGTAGTCATTCAAAGATTTGGAGATGTTTACTACAACTGATGGTTCCGGGCTCTCCACGCTAATGGGGCTAGTTTCTTTTTTATTGGACTTTATTTGCATATTGCTCGAGGTCTCTATTACGGATCTTACCTTTTCAAAGAAGTTTGGTCCATTGGAGTTGGGATCTTGCTTTTGGTTATAGCAAGGGCCTTCTTGGGCTATGTCCTTCCATGGGGACAAATGTCTTTTTGAGGGGCAACAGTTATTACTAGTCTGTTTTCGGCACTCCCTGTTATTGGAGGAGATGTAGTAGTTTGATTGTGAGGGGGCTTCTCGGTAGACAATGCGACGCTAACTCGATTTTTTGGGCTTCATTTCGTATTACCTTTCATTGTGGCGGGGCTAGTAGGGCTCCACCTATTCTTCTTGCACGACCAAGGGTCGTCAAATCCTTTAGGGGTGAGGTCAGATTTTAATAAGGTTGTTTTTCACCACTATTATGTCCTTAAGGATTTAGTGGGGGCTTGTTTTTTTATTGGCCCCTTACTTGGATTAGTTTACTTTAATCCTTGGCTTCTAGGGGATCCCGAAAATTTTATTGAGGGGAACCCACTGGTTACACCACATCATATTCAACCTGAATGGTATTTTTTATTTGCTTATGCTATTTTGCGGGCTGTGCCTAACAAATTAGGAGGAGTAGTAGCGTTGGCCGCGGCGGTCTTGGTGTTGTTTTATATGCCAGTAAGACCTAAAACTTCTGTTAAAGGGCTATCTTTTTATCCGTGAGGAAAAATGTTATTTTTTAGGCTTGTTGGAGTGTTTTTTCTGCTTACTTGGGCAGGATCGAAGCCAGTAGAAGAACCATATGTAGTGATTAGACAGGTTTTAAGTTTCTTGTATTTTTCTTATTTTGCCTTAATTGGACCTATCACTCGTATTCAAGACAAGTTGTTGTAA

LJS_m3 1 ATTTATAAGGCTTCCCGATTTAGACCACTAACTAGGATTATTCGGCATACGGTAGTCACTTTGCCAACTCCTATTAACATTTCAACGTGGTGAAATTTTGGTTCCCTCCTGGGAATTTGTTTGGCGAGTCAGATTTTTAGAGGGTTGGTTCTGAGACTCCACTTTACTGCTCATACCGATCTTAGTTTTTATGTAGTCATTCAAAGATTTGGAGATGTTTACTACAACTGATGGTTCCGGGCTCTCCACGCTAATGGGGCTAGTTTCTTTTTTATTGGACTTTATTTGCATATTGCTCGAGGTCTCTATTACGGATCTTACCTTTTCAAAGAAGTTTGGTCCATTGGAGTTGTGATCTTGCTTTTGGTTATAGCAAGGGCCTTCTTGGGCTATGTCCTTCCATGGGGACAAATGTCTTTTTGAGGGGCAACAGTTATTACTAGTCTGTTTTCGGCACTCCCTGTTATTGGAGGAGATGTAGTAGTTTGATTGTGAGGGGGCTTCTCGGTAGACAATGCGACGCTAACTCGATTTTTTGGGCTTCATTTCGTATTACCTTTCATTGTGGCGGGGCTAGTAGGGCTCCACCTATTCTTCTTGCACGACCAAGGGTCGTCAAATCCTTTAGGGGTGAGGTCAGATTTTAATAAGGTTGTTTTTCACCACTATTATGTCCTTAAGGATTTAGTGGGGGCTTGTTTTTTTATTGGCCCCTTACTTGGATTAGTTTACTTTAATCCTTGGCTTCTAGGGGATCCCGAAAATTTTATTGAGGGGAACCCACTGGTTACACCACATCATATTCAACCTGAATGGTATTTTTTATTTGCTTATGCTATTTTGCGGGCTGTGCCTAACAAATTAGGAGGAGTAGTAGCGTTGGCCGCGGCGGTCTTGGTGTTGTTTTATATGCCAGTAAGACCTAAAACTTCTGTTAAAGGGCTATCTTTTTATCCGTGAGGAAAAATGTTATTTTTTAGGCTTGTTGGAGTGTTTTTTCTGCTTACTTGGGCAGGATCAAAGCCAGTAGAAGAACCATATGTAGTGATTAGACAGGTTTTAAGTTTCTTGTATTTTTCTTATTTTGCCTTAATTGGACCTATCACTCGTATTCAAGACAAGTTGTTGTAA

LJS_m4 2 ATTTATAAGGCTTCCCGATTTAGACCACTAACTAGGATTATTCGGCATACGGTAGTCACTTTGCCAACTCCTATTAACATTTCAACGTGGTGAAATTTTGGTTCCCTCCTGGGAATTTGTTTGGCGAGTCAGATTTTTAGAGGGTTGGTTCTGAGACTCCACTTTACTGCTCATACCGATCTTAGTTTTTATGTAGTCATTCAAAGATTTGGAGATGTTTACTACAACTGATGGTTCCGGGCTCTCCACGCTAATGGGGCTAGTTTCTTTTTTATTGGACTTTATTTGCATATTGCTCGAGGTCTCTATTACGGATCTTACCTTTTCAAAGAAGTTTGGTCCATTGGAGTTGTGATCTTGCTTTTGGTTATAGCAAGGGCCTTCTTGGGCTATGTCCTTCCATGGGGACAAATGTCTTTTTGAGGGGCAACAGTTATTACTAGTCTGTTTTCGGCACTCCCTGTTATTGGAGGAGATGTAGTAGTTTGATTGTGAGGGGGCTTCTCGGTAGACAATGCGACGCTAACTCGATTTTTTGGGCTTCATTTCGTATTACCTTTCATTGTGGCGGGGCTAGTAGGGCTCCACCTATTCTTCTTGCACGACCAAGGGTCGTCAAATCCTTTAGGGGTGAGGTCAGATTTTAATAAGGTTGTTTTTCACCACTATTATGTCCTTAAGGATTTAGTGGGGGCTTGTTTTTTTATTGGCCCCTTACTTGGATTAGTTTACTTTAATCCTTGGCTTCTAGGGGATCCCGAAAATTTTATTGAGGGGAACCCACTGGTTACACCACATCATATTCAACCTGAATGGTATTTTTTATTTGCTTATGCTATTTTGCGGGCTGTGCCTAACAAATTAGGAGGAGTAGTAGCGTTGGCCGCGGCGGTCTTGGTGTTGTTTTATATGCCAGTAAGACCTAAAACTTCTGTTAAAGGGCTATCTTTTTATCCGTGAGGAAAAATGTTATTTTTTAGGCTTGTTGGAGTGTTTTTTCTGCTTACTTGGGCAGGATCGAAGCCAGTAGAAGAACCATATGTAGTGATTAGACAGGTTTTAAGATTCTTGTATTTTTCTTATTTTGCCTTAATTGGACCTATCACTCGTATTCAAGACAAGTTGTTGTAA

LJS_f3 8 ATTTATAAGGCTTCCCGATTTAGACCACTAACTAGGATTATTCGGCATACGGTAGTCACTTTGCCAACTCCTATTAACATTTCAACGTGGTGAAATTTTGGTTCCCTCCTGGGAATTTGTTTGGCGAGTCAGATTTTTAGAGGGTTGGTTCTGAGACTCCACTTTACTGCTCATACCGATCTTAGTTTTTATGTAGTCATTCAAAGATTTGGAGATGTTTACTACAACTGATGGTTCCGGGCTCTCCACGCTAATGGGGCTAGTTTCTTTTTTATTGGACTTTATTTGCATATTGCTCGAGGTCTCTATTACGGATCTTACCTTTTCAAAGAAGTTTGGTCCATTGGAGTTGTGATCTTGCTTTTGGTTATAGCAAGGGCCTTCTTGGGCTATGTCCTTCCATGGGGACAAATGTCTTTTTGAGGGGCAACAGTTATTACTAGTCTGTTTTCGGCACTCCCTGTTATTGGAGGAGATGTAGTAGTTTGATTGTGAGGGGGCTTCTCGGTAGACAATGCGACGCTAACTCGATTTTTTGGGCTTCATTTCGTATTACCTTTCATTGTGGCGGGGCTAGTAGGGCTCCACCTATTCTTCTTGCACGACCAAGGGTCGTCAAATCCTTTAGGGGTGAGGTCAGATTTTAATAAGGTTGTTTTTCACCACTATTATGTCCTTAAGGATTTAGTGGGGGCTTGTTTTTTTATTGGCCCCTTACTTGGATTAGTTTACTTTAATCCTTGGCTTCTAGGGGATCCCGAAAATTTTATTGAGGGGAACCCACTGGTTACACCACATCATATTCAACCTGAATGGTATTTTTTATTTGCTTATGCTATTTTGCGGGCTGTGCCTAACAAATTAGGAGGAGTAGTAGCGTTGGCCGCGGCGGTCTTGGTGTTGTTTTATATGCCAGTAAGACCTAAAACTTCTGTTAAAGGGCTATCTTTTTATCCGTGAGGAAAAATGTTATTTTTTAGGCTTGTTGGAGTGTTTTTTCTGCTTACTTGGGCAGGATCGAAGCCAGTAGAAGAACCATATGTAGTGATTAGACAGGTTTTAAGTTTCTTGTATTTTTCTTATTTTGCCTTAATTGGACCTATCACTCGTATTCAAGACAAGTTGTTGTAA

}

SampleName="Naut 5"

SampleSize= 13

SampleData= {

LJS_f3 10

LJS_m10 1

naut_f4 1 ATTTATAAGGCTTCCCGATTTAGACCACTAACTAGGATTATTCGGCATACGGTAGTCACTTTGCCAACTCCTATTAACATTTCAACGTGGTGAAATTTTGGTTCCCTCCTGGGAATTTGTTTGGCGAGTCAGATTTTTAGAGGGTTGGTTCTGAGACTCCACTTTACTGCTCATACCGATCTTAGTTTTTATGTAGTCATTCAAAGATTTGGAGATGTTTACTACAACTGATGGTTCCGGGCTCTCCACGCTAATGGGGCTAGTTTCTTTTTTATTGGACTTTATTTGCATATTGCTCGAGGTCTCTATTACGGATCTTACCTTTTCAAAGAAGTTTGGTCCATTGGAGTTGTGATCTTGCTTTTGGTTATAGCAAGGGCCTTCTTGGGCTATGTCCTTCCATGGGGACAAATGTCTTTTTGAGGGGCAACAGTTATTACTAGTCTGTTTTCGGCACTCCCTGTTATTGGAGGAGATGTAGTAGTTTGATTGTGAGGGGGCTTCTCGGTAGACAATGCGACGCTAACTCGATTTTTTGGGCTTCATTTCGTATTACCTTTCATTGTGGCGGGGCTAGTAGGGCTCCACCTATTCTTCTTGCACGACCAAGGGTCGTCAAATCCTTTAGGGGTGAGGTCAGATTTTAATAAGGTTGTTTTTCACCACTATTATGTCCTTAAGGATTTAGTGGGGGCTTGTTTTTTTATTGGCCCCTTACTTGGATTAGTTTACTTTAATCCTTGGCTTCTAGGGGATCCCGAAAATTTTATTGAGGGGAACCCACTGGTTACACCACATCATATTCAACCTGAATGGTATTTTTTATTTGCTTATGCTATTTTGCGGGCTGTGCCTAACAAATTAGGAGGAGTAGTAGCGTTGGCCGCGGCGGTCTTGGTGTTGTTTTATATGCCAGTAAGACCTAAAACTTCTGTTAAAGGGCTATCTTTTTATCCGTGAGGAAAAATGTTATTTTTTAGGCTTGTTGGAGTGTTTTTTCTGCTTACTTGGGCAGGATCGAAGCCAGTAGAAGAACCATATGTAGTGATTAGGCAGGTTTTAAGTTTCTTGTATTTTTCTTATTTTGCCTTAATTGGACCTATCACTCGTATTCAAGACAAGTTGTTGTAA

naut_m3 1 ATTTATAAGGCTTCCCGATTTAGACCACTAACTAGGATTATTCGGCATACGGTAGTTACTTTGCCAACTCCTATTAACATTTCAACGTGGTGAAATTTTGGTTCCCTCCTGGGAATTTGTTTGGCGAGTCAGATTTTTAGAGGGCTGGTTCTGAGACTCCACTTTACTGCTCATACCGATCTTAGTTTTTATGTAGTCATTCAAAGATTTGGAGATGTTTACTACAACTGATGGTTCCGGGCTCTCCACGCTAATGGGGCTAGTTTCTTTTTTATTGGACTTTATTTGCATATTGCTCGAGGTCTCTATTACGGATCTTACCTTTTCAAAGAAGTTTGGTCCATTGGAGTTGTGATCTTGCTTTTGGTTATAGCAAGGGCCTTCTTGGGCTATGTCCTTCCATGGGGACAAATGTCTTTTTGAGGGGCAACGGTTATTACTAGTCTGTTTTCGGCACTCCCTGTTATTGGAGGAGATGTAGTAGTTTGATTGTGAGGGGGCTTCTCGGTAGACAATGCGACGCTAACTCGATTTTTTGGGCTTCATTTCGTATTACCTTTCATTGTGGCGGGGCTAGTGGGGCTCCACCTATTCTTCTTGCACGACCAAGGGTCGTCAAATCCTTTAGGGGTGAGGTCAGATTTTAATAAGGTTGTTTTTCACCACTATTATGTCCTTAAGGATTTAGTGGGGGCTTGTTTTTTTATTGGCCCTTTACTTGGATTAGTTTACTTTAATCCCTGGCTTCTAGGGGATCCCGAAAATTTTATTGAGGGGAACCCACTGGTTACACCACATCATATTCAACCTGAATGGTATTTTTTATTTGCTTATGCTATTTTGCGGGCTGTGCCTAACAAATTAGGAGGAGTAGTAGCGTTGGCCGCGGCGGTCTTGGTGTTGTTTTATATACCAGTGAGACCTAAAACTTCTGTTAAAGGGCTATCTTTTTATCCGTGAGGAAAAATGTTATTTTTTAGGCTTGTTGGAGTGTTTTTTCTGCTTACTTGGGCAGGATCGAAGCCAGTAGAAGAACCATATGTAGTGATTAGACAGGTTTTAAGTTTCTTGTATTTTTCTTATTTTGCCTTAATTGGACCTATCACTCGTATTCAAGACAAGTTGTTGTAA }

SampleName="BR 6"

SampleSize= 14

SampleData= {

LJS_f3 1

Br_f12 5 ATTTATAAGGCTTCCCGATTTAGACCACTAACTAGGATTATTCGGCATACGGTAGTTACTTTGCCAACTCCTATTAACATTTCAACGTGGTGAAATTTTGGTTCCCTCCTGGGAATTTGTTTGGCGAGTCAGATTTTTAGAGGGCTGGTTCTGAGACTCCACTTTACTGCTCATACCGATCTTAGTTTTTATGTAGTCATTCAAAGATTTGGAGATGTTTACTACAACTGATGGTTCCGGGCTCTCCACGCTAATGGGGCTAGTTTCTTTTTTATTGGACTTTATTTGCATATTGCTCGAGGTCTCTATTACGGATCTTACCTTTTCAAAGAAGTTTGGTCCATTGGAGTTGTGATCTTGCTTTTGGTTATAGCAAGGGCCTTCTTGGGCTATGTCCTTCCATGGGGACAAATGTCTTTTTGAGGGGCAACGGTTATTACTAGTCTGTTTTCGGCACTCCCTGTTATTGGAGGAGATGTAGTAGTTTGATTGTGAGGGGGCTTCTCGGTAGACAATGCGACGTTAACTCGATTTTTTGGGCTTCATTTCGTATTACCTTTCATTGTGGCGGGGCTAGTGGGGCTCCACCTATTCTTCTTGCACGACCAAGGGTCGTCAAATCCTTTAGGGGTGAGGTCAGATTTTAATAAGGTTGTTTTTCACCACTATTATGTCCTTAAGGATTTAGTGGGGGCTTGTTTTTTTATTGGCCCTTTACTTGGATTAGTTTACTTTAATCCCTGGCTTCTAGGGGATCCCGAAAATTTTATTGAGGGGAACCCACTGGTTACACCACATCATATTCAACCTGAATGGTATTTTTTATTTGCTTATGCTATTTTGCGGGCTGTGCCTAACAAATTAGGAGGAGTAGTAGCGTTGGCCGCGGCGGTCTTGGTGTTGTTTTATATACCAGTAAGACCTAAAACTTCTGTTAAAGGGCTATCTTTTTATCCGTGAGGAAAAATGTTATTTTTTAGGCTTGTTGGAGTGTTTTTTCTGCTTACTTGGGCAGGATCGAAGCCAGTAGAAGAACCATATGTAGTGATTAGACAGGTTTTAAGTTTCTTGTATTTTTCTTATTTTGCCTTAATTGGACCTATCACTCGTATTCAAGACAAGTTGTTGTAA

BR_f10 1 ATTTATAAGGCTTCCCGATTTAGACCACTAACTAGGATTATTCGGCATACGGTAGTCACTTTGCCAACTCCTATTAACATTTCAACGTGGTGAAATTTTGGTTCCCTCCTGGGAATTTGTTTGGCGAGTCAGATTTTTAGAGGGTTGGTTCTGAGACTCCACTTTACTGCTCATACCGATCTTAGTTTTTATGTAGTCATTCAAAGATTTGGAGATGTTTACTACAACTGATGGTTCCGGGCTCTCCACGCTAATGGGGCTAGTTTCTTTTTTATTGGACTTTATTTGCATATTGCTCGAGGTCTCTATTACGGATCTTACCTTTTCAAAGAAGTTTGGTCCATTGGAGTTGTGATCTTGCTTTTGGTTATAGCAAGGGCCTTCTTGGGCTATGTCCTTCCATGGGGACAAATGTCTTTTTGAGGGGCAACAGTTATTACTAGTCTGTTTTCGGCACTCCCTGTTATTGGAGGAGATGTAGTAGTTTGATTGTGAGGGGGCTTCTCGGTAGACAATGCGACGCTAACTCGATTTTTTGGGCTTCATTTCGTATTACCTTTCATTGTGGCGGGGCTAGTAGGGCTCCACCTATTCTTCTTGCACGACCAAGGGTCGTCAAATCCTTTAGGGGTGAGGTCAGATTTTAATAAGGTTGTTTTTCACCACTATTATGTCCTTAAGGATTTAGTGGGGGCTTGTTTTTTTATTGGCCCCTTACTTGGATTAGTTTACTTTAATCCTTGGCTTCTAGGGGATCCCGAAAATTTTATTGAGGGAAACCCACTGGTTACACCACATCATATTCAACCTGAATGGTATTTTTTATTTGCTTATGCTATTTTGCGGGCTGTGCCTAACAAATTAGGAGGAGTAGTAGCGTTGGCCGCGGCGGTCTTGGTGTTGTTTTATATGCCAGTAAGACCTAAAACTTCTGTTAAAGGGCTATCTTTTTATCCGTGAGGAAAAATGTTATTTTTTAGGCTTGTTGGAGTGTTTTTTCTGCTTACTTGGGCAGGATCGAAGCCAGTAGAAGAACCATATGTAGTGATTAGACAGGTTTTAAGTTTCTTGTATTTTTCTTATTTTGCCTTAATTGGACCTATCACTCGTATTCAAGACAAGTTGTTGTAA

BR_m4 1 ATTTATAAGGCTTCCCGATTTAGACCACTAACTAGGATTATTCGGCATACGGTAGTCACTTTGCCAACTCCTATTAACATTTCAACGTGGTGAAATTTTGGTTCCCTCCTGGGAATTTGTTTGGCGAGTCAGATTTTTAGAGGGTTGGTTCTGAGACTCCACTTTACTGCTCATACCGATCTTAGTTTTTATGTAGTCATTCAAAGATTTGGAGATGTTTACTACAACTGATGGTTCCGGGCTCTCCACGCTAATGGGGCTAGTTTCTTTTTTATTGGACTTTATTTGCATATTGCTCGAGGTCTCTATTACGGATCTTACCTTTTCAAAGAAGTTTGGTCCATTGGAGTTGTGATCTTGCTTTTGGTTATAGCAAGGGCCTTCTTGGGCTATGTCCTTCCATGGGGACAAATGTCTTTTTGAGGGGCAACAGTTATTACTAGTCTGTTTTCGGCACTCCCTGTTATTGGAGGAGATGTAGTAGTTTGATTGTGAGGGGGCTTCTCGGTAGACAATGCGACGCTAACTCGATTTTTTGGGCTTCATTTCGTATTACCTTTCATTGTGGCGGGGCTAGTAGGGCTCCACCTATTCTTCTTGCACGACCAAGGGTCGTCAAATCCTTTAGGGGTGAGGTCAGATTTTAATAAGGTTGTTTTTCACCACTATTATGTCCTTAAGGATTTAGTGGGGGCTTGTTTTTTTATTGGCCCTTTACTTGGATTAGTTTACTTTAATCCCTGGCTTCTAGGGGATCCCGAAAATTTTATTGAGGGGAACCCACTGGTTACACCACATCATATTCAACCTGAATGGTATTTTTTATTTGCTTATGCTATTTTGCGGGCTGTGCCTAACAAATTAGGAGGAGTAGTAGCGTTGGCCGCGGCGGTCTTGGTGTTGTTTTATATGCCAGTAAGACCTAAAACTTCTGTTAAAGGGCTATCTTTTTATCCGTGAGGAAAAATGTTATTTTTTAGGCTTGTTGGAGTGTTTTTTCTGCTTACTTGGGCAGGATCGAAGCCAGTAGAAGAACCATATGTAGTGATTAGACAGGTTTTAAGTTTCTTGTATTTTTCTTATTTTGCCTTAATTGGACCTATCACTCGTATTCAAGACAAGTTGTTGTAA

BR_m5 4 ATTTATAAGGCTTCCCGATTTAGACCACTAACTAGGATTATTCGGCATACAGTAGTTACTTTGCCAACTCCTATTAACATTTCAACGTGGTGAAATTTTGGCTCCCTCCTGGGAATTTGTTTGGCGAGTCAGATTTTTAGAGGGCTGGTTCTGAGACTCCACTTTACTGCTCATACCGATCTTAGTTTTTATGTAGTCATTCAAAGATTTGGAGATGTTTACTACAACTGATGGTTCCGGGCTCTCCACGCTAATGGGGCTAGTTTCTTTTTTATTGGACTTTATTTGCATATTGCTCGAGGTCTCTATTACGGATCTTACCTTTTCAAAGAAGTTTGGTCCATTGGAGTTGTGATCTTGCTTTTGGTTATAGCAAGGGCCTTCTTGGGCTATGTCCTTCCATGGGGACAAATGTCTTTTTGAGGGGCAACGGTTATTACTAGTCTGTTTTCGGCACTCCCTGTTATTGGAGGAGATGTAGTAGTTTGATTGTGAGGGGGCTTCTCGGTAGACAATGCGACGCTAACTCGATTTTTTGGGCTTCATTTCGTATTACCTTTCATTGTGGCGGGGCTAGTGGGGCTCCACCTATTCTTCTTGCACGACCAAGGGTCGTCAAATCCTTTAGGGGTGAGGTCAGATTTTAATAAGGTTGTTTTTCACCACTATTATGTCCTTAAGGATTTAGTGGGGGCTTGTTTTTTTATTGGCCCTTTACTTGGATTAGTTTACTTTAATCCCTGGCTTCTAGGGGATCCCGAAAATTTTATTGAGGGGAACCCACTGGTTACACCACATCATATTCAACCTGAATGGTATTTTTTATTTGCTTATGCTATTTTGCGGGCTGTGCCTAACAAATTAGGAGGAGTAGTAGCGTTGGCCGCGGCGGTCTTGGTGTTGTTTTATATACCAGTAAGACCTAAAACTTCTGTTAAAGGGCTATCTTTTTATCCGTGAGGAAAAATGTTATTTTTTAGGCTTGTTGGAGTGTTTTTTCTGCTTACTTGGGCAGGATCGAAGCCAGTAGAAGAACCATATGTAGTGATTAGACAGGTTTTAAGTTTCTTGTATTTTTCTTATTTTGCCTTAATTGGACCTATCACTCGTATTCAAGACAAGTTGTTGTAA

BR_f13 1 ATTTATAAGGCTTCCCGATTTAGACCACTAACTAGGATTATTCGGCATACGGTAGTTACTTTGCCAACTCCTATTAACATTTCAACGTGGTGAAATTTTGGTTCCCTCCTGGGAATTTGTTTGGCGAGTCAGATTTTTAGAGGGCTGGTTCTGAGACTCCACTTTACTGCTCATACCGATCTTAGTTTTTATGTAGTCATTCAAAGATTTGGAGATGTTTACTACAACTGATGGTTCCGGGCTCTCCACGCTAATGGGGCTAGTTTCTTTTTTATTGGACTTTATTTGCATATTGCTCGAGGTCTCTATTACGGATCTTACCTTTTCAAAGAAGTTTGGTCCATTGGAGTTGTGATCTTGCTTTTGGTTATAGCAAGGGCCTTCTTGGGCTATGTCCTTCCATGGGGACAAATGTCTTTTTGAGGGGCAACGGTTATTACTAGTCTGTTTTCGGCACTCCCTGTTATTGGAGGAGATGTAGTAGTTTGATTGTGAGGGGGCTTCTCGGTAGACAATGCGACGTTAACTCGATTTTTTGGGCTTCATTTCGTATTACCTTTCATTGTGGCGGGGCTAGTGGGGCTCCACCTATTCTTCTTGCACGACCAAGGGTCGTCAAATCCTTTAGGGGTGAGGTCAGATTTTAATAAGGTTGTTTTTCACCACTATTATGTCCTTAAGGATTTAGTGGGGGCTTGTTTTTTTATTGGCCCTTTACTTGGATTAGTTTACTTTAATCCCTGGCTTCTAGGGGATCCCGAAAATTTTATTGAGGGGAACCCACTGGTTACACCACATCATATTCAACCTGAATGGTATTTTTTATTTGCTTATGCTATTTTGCGGGCTGTGCCTAACAAATTAGGAGGAGTAGTAGCGTTGGCCGCGGCGGTCTTGGTGTTGTTTTATATACCAGTAAGACCTAAAACTTCTGTTAAAGGGCTATCTTTTTATCCGTGAGGAAAAATGTTATTTTTTAGGCTTGTTGGAGTGTTTTTTCTGCTTACTTGGGCAGGATCGAAGCCAGTAGAAGAACCATATTTAGTGATTAGACAGGTTTTAAGTTTCTTGTATTTTTCTTATTTTGCCTTAATTGGACCTATCACTCGTATTCAAGACAAGTTGTTGTAA

BR_f4 1 ATTTATAAGGCTTCCCGATTTAGACCACTAACTAGGATTATTCGGCATACAGTAGTTACTTTGCCAACTCCTATTAACATTTCAACGTGGTGAAATTTTGGTTCCCTCCTGGGAATTTGTTTGGCGAGTCAGATTTTTAGAGGGCTGGTTCTGAGACTCCACTTTACTGCTCATACCGATCTTAGTTTTTATGTAGTCATTCAAAGATTTGGAGATGTTTACTACAACTGATGGTTCCGGGCTCTCCACGCTAATGGGGCTAGTTTCTTTTTTATTGGACTTTATTTGCATATTGCTCGAGGTCTCTATTACGGATCTTACCTTTTCAAAGAAGTTTGGTCCATTGGAGTTGTGATCTTGCTTTTGGTTATAGCAAGGGCCTTCTTGGGCTATGTCCTTCCATGGGGACAAATGTCTTTTTGAGGGGCAACGGTTATTACTAGTCTGTTTTCGGCACTCCCTGTTATTGGAGGAGATGTAGTAGTTTGATTGTGAGGGGGCTTCTCGGTAGACAATGCGACGCTAACTCGATTTTTTGGGCTTCATTTCGTATTACCTTTCATTGTGGCGGGGCTAGTGGGGCTCCACCTATTCTTCTTGCACGACCAAGGGTCGTCAAATCCTTTAGGGGTGAGGTCAGATTTTAATAAGGTTGTTTTTCACCACTATTATGTCCTTAAGGATTTAGTGGGGGCTTGTTTTTTTATTGGCCCTTTACTTGGATTAGTTTACTTTAATCCCTGGCTTCTAGGGGATCCCGAAAATTTTATTGAGGGGAACCCACTGGTTACACCACATCATATTCAACCTGAATGGTATTTTTTATTTGCTTATGCTATTTTGCGGGCTGTGCCTAACAAATTAGGAGGAGTAGTAGCGTTGGCCGCGGCGGTCTTGGTGTTGTTTTATATACCAGTAAGACCTAAAACTTCTGTTAAAGGGCTATCTTTTTATCCGTGAGGAAAAATGTTATTTTTTAGGCTTGTTGGAGTGTTTTTTCTGCTTACTTGGGCAGGATCGAAGCCAGTAGAAGAACCATATGTAGTGATTAGACAGGTTTTAAGTTTCTTGTATTTTTCTTATTTTGCCTTAATTGGACCTATCACTCGTATTCAAGACAAGTTGTTGTAA }

SampleName="SIO 7"

SampleSize= 14

SampleData= {

LJS_f3 12

SIO_m18 1 ATTTATAAGGCTTCCCGATTTAGACCACTAACTAGGATTATTCGGCATACGGTAGTCACTTTGCCAACTCCTATTAACATTTCAACGTGGTGAAATTTTGGTTCCCTCCTGGGAATTTGTTTGGCGAGTCAGATTTTTAGAGGGTTGGTTCTGAGACTCCACTTTACTGCTCATACCGATCTTAGTTTTTATGTAGTCATTCAAAGATTTGGAGATGTTTACTACAACTGATGGTTCCGGGCTCTCCACGCTAATGGGGCTAGTTTCTTTTTTATTGGACTTTATTTGCATATTGCTCGAGGTCTCTATTACGGATCTTACCTTTTCAAAGAAGTTTGGTCCATTGGAGTTGTGATCTTGCTTTTGGTTATAGCAAGGGCCTTCTTGGGCTATGTCCTTCCATGGGGACAAATGTCTTTTTGAGGGGCAACAGTTATTACTAGTCTGTTTTCGGCACTCCCTGTTATTGGAGGAGATGTAGTAGTTTGATTGTGAGGGGGCTTCTCGGTAGACAATGCGACGCTAACTCGATTTTTTGGGCTTCATTTCGTATTACCTTTCATTGTGGCGGGGCTAGTAGGGCTCCACCTATTCTTCTTGCACGACCAAGGGTCGTCAAATCCTTTAGGGGTGAGGTCAGATTTTAATAAGGTTGTTTTTCACCACTATTATGTCCTTAAGGATTTAGTGGGGGCTTGTTTTTTTATTGGCCCCTTACTTGGATTAGTTTACCTTAATCCTTGGCTTCTAGGGGATCCCGAAAATTTTATTGAGGGGAACCCACTGGTTACACCACATCATATTCAACCTGAATGGTATTTTTTATTTGCTTATGCTATTTTGCGGGCTGTGCCTAACAAATTAGGAGGAGTAGTAGCGTTGGCCGCGGCGGTCTTGGTGTTGTTTTATATGCCAGTAAGACCTAAAACTTCTGTTAAAGGGCTATCTTTTTATCCGTGAGGAAAAATGTTATTTTTTAGGCTTGTTGGAGTGTTTTTTCTGCTTACTTGGGCGGGATCGAAGCCAGTAGAAGAACCATATGTAGTGATTAGACAGGTTTTAAGTTTCTTGTATTTTTCTTATTTTGCCTTAATTGGACCTATCACTCGTATTCAAGACAAGTTGTTGTAA

SIO_f18 1 ATTTATAAGGCTTCCCGATTTAGACCACTAACTAGGATTATTCGGCATACGGTAGTCACTTTGCCAACTCCTATTAACATTTCAACGTGGTGAAATTTTGGTTCCCTCCTGGGAATTTGTTTGGCGAGTCAGATTTTTAGAGGGTTGGTTCTGAGACTCCACTTTACTGCTCATACCGATCTTAGTTTTTATGTAGTCATTCAAAGATTTGGAGATGTTTACTACAACTGATGGTTCCGGGCTCTCCACGCTAATGGGGCTAGTTTCTTTTTTATTGGACTTTATTTGCATATTGCTCGAGGTCTCTATTACGGATCTTACCTTTTCAAAGAAGTTTGGTCCATTGGAGTTGTGATCTTGCTTTTGGTTATAGCAAGGGCCTTCTTGGGCTATGTCCTTCCATGGGGACAAATGTCTTTTTGAGGGGCAACAGTTATTACTAGTCTGTTTTCGGCACTCCCTGTTATTGGAGGAGATGTAGTAGTTTGATTGTGAGGGGGCTTCTCGGTAGACAATGCGACG?TAACTCGATTTTTTGGGCTTCATTTCGTATTACCTTTCATTGTGGCGGGGCTAGTAGGGCTCCACCTATTCTTCTTGCACGACCAAGGGTCGTCAAATCCTTTAGGGGTGAGGTCAGATTTTAATAAGGTTGTTTTTCACCACTATTATGTCCTTAAGGATTTAGTGGGGGCTTGTTTTTTTATTGGCCCCTTACTTGGATTAGTTTACCTTAATCCTTGGCTTCTAGGGGATCCCGAAAATTTTATTGAGGGGAACCCACTGGTTACACCACATCATATTCAACCTGAATGGTATTTTTTATTTGCTTATGCTATTTTGCGGGCTGTGCCTAACAAATTAGGAGGAGTAGTAGCGTTGGCCGCGGCGGTCTTGGTGTTGTTTTATATGCCAGTAAGACCTAAAACTTCTGTTAAAGGGCTATCTTTTTATCCGTGAGGAAAAATGTTATTTTTTAGGCTTGTTGGAGTGTTTTTTCTGCTTACTTGGGCAGGATCGAAGCCAGTAGAAGAACCATATGTAGTGATTAGACAGGTTTTAAGTTTCTTGTATTTTTCTTATTTTGCCTTAATTGGACCTATCACTCGTATTCAAGACAAGTTGTTGTAA

}

SampleName="LJP 8"

SampleSize= 14

SampleData= {

LJP_f3 1 ATTTATAAGGCTTCCCGATTTAGACCACTAACTAGGATTATTCGGCATACGGTAGTTACTTTGCCAACTCCTATTAACATTTCAACGTGGTGAAATTTTGGTTCCCTCCTGGGAATTTGTTTGGCGAGTCAGATTTTTAGAGGGTTGGTTCTGAGACTCCACTTTACTGCTCATACCGATCTTAGTTTTTATGTAGTCATTCAAAGATTTGGAGATGTTTACTACAACTGATGGTTCCGGGCTCTCCACGCTAATGGGGCTAGTTTCTTTTTTATTGGACTTTATTTGCATATTGCTCGAGGTCTCTATTACGGATCTTACCTTTTCAAAGAAGTTTGGTCCATTGGAGTTGTGATCTTGCTTTTGGTTATAGCAAGGGCCTTCTTGGGCTATGTCCTTCCATGGGGACAAATGTCTTTTTGAGGGGCAACGGTTATTACTAGTCTGTTTTCGGCACTCCCTGTTATTGGAGGAGATGTAGTAGTTTGATTGTGAGGGGGCTTCTCGGTAGACAATGCGACGCTAACTCGATTTTTTGGGCTTCATTTCGTATTACCTTTCATTGTAGCGGGGCTAGTAGGGCTCCACCTATTCTTCTTGCACGACCAAGGGTCGTCAAATCCTTTAGGGGTGAGGTCAGATTTTAATAAGGTTGTTTTTCACCACTATTATGTCCTTAAGGATTTAGTGGGGGCTTGTTTTTTTATTGGCCCTTTACTTGGGTTAGTTTACTTTAATCCCTGGCTTCTAGGGGATCCCGAAAATTTTATTGAGGGGAACCCACTGGTTACACCACATCATATTCAACCTGAATGGTATTTTTTATTTGCTTATGCTATTTTGCGGGCTGTGCCTAACAAATTAGGAGGCGTAGTAGCGTTGGCCGCGGCGGTCTTGGTGTTGTTTTATATACCAGTAAGACCTAAAACTTCTGTTAAAGGGCTATCTTTTTATCCGTGAGGAAAAATGTTGTTTTTTAGGCTTATTGGAGTGTTTTTTCTGCTTACTTGGGCAGGATCGAAGCCAGTAGAAGAACCATATGTAGTGATTAGACAGGTTTTAAGTTTCTTGTATTTTTCTTATTTTGCCTTAATTGGACCTATCACTCGTATTCAAGACAAGTTGTTGTAA

LJP_f7 1 ATTTATAAGGCTTCCCGATTTAGACCACTAACTAGGATTATTCGGCATACGGTAGTTACTTTGCCAACTCCTATTAACATTTCAACGTGGTGAAATTTTGGTTCCCTCCTGGGAATTTGTTTGGCGAGTCAGATTTTTAGAGGGTTGGTTCTGAGACTCCACTTTACTGCTCATACCGATCTTAGTTTTTATGTAGTCATTCAAAGATTTGGAGATGTTTACTACAACTGATGGTTCCGGGCTCTCCACGCTAATGGGGCTAGTTTCTTTTTTATTGGACTTTATTTGCATATTGCTCGAGGTCTCTATTACGGATCTTACCTTTTCAAAGAAGTTTGGTCCATTGGAGTTGTGATCTTGCTTTTGGTTATAGCAAGGGCCTTCTTGGGCTATGTCCTTCCATGGGGACAAATGTCTTTTTGAGGGGCAACGGTTATTACTAGTCTGTTTTCGGCACTCCCTGTTATTGGAGGAGATGTAGTAGTTTGATTGTGAGGGGGCTTCTCGGTAGACAATGCGACGCTAACTCGATTTTTTGGGCTTCATTTCGTATTACCTTTCATTGTAGCGGGGCTAGTAGGGCTCCACCTATTCTTCTTGCACGACCAAGGGTCGTCAAATCCTTTAGGGGTGAGGTCAGATTTTAATAAGGTTGTTTTTCACCACTATTATGTCCTTAAGGATTTAGTGGGGGCTTGTTTTTTTATTGGCCCTTTACTTGGATTAGTTTACTTTAATCCTTGGCTTCTAGGGGATCCCGAAAATTTTATTGAGGGGAACCCACTGGTTACACCACATCATATTCAACCTGAATGGTATTTTTTATTTGCTTATGCTATTTTGCGGGCTGTGCCTAACAAATTAGGAGGCGTAGTAGCGTTGGCCGCGGCGGTCTTGGTGTTGTTTTATATGCCAGTAAGACCTAAAACTTCTGTTAAAGGGCTATCTTTTTATCCGTGAGGAAAAATGTTTTTTTTTAGGCTTGTTGGAGTGTTTTTTCTGCTTACTTGGGCAGGATCGAAGCCAGTAGAAGAACCATATGTAGTGATTAGACAGGTTTTAAGTTTCTTGTATTTTTCTTATTTTGCCTTAATTGGACCTATCACTCGTATTCAAGACAAGTTGTTGTAA

LJP_f2 1 ATTTATAAGGCTTCCCGATTTAGACCACTAACTAGGATTATTCGGCATACGGTAGTTACTTTGCCAACTCCTATTAACATTTCAACGTGGTGAAATTTTGGTTCCCTCCTGGGAATTTGTTTGGCGAGTCAGATTTTTAGAGGGTTGGTTCTGAGACTCCACTTTACTGCTCATACCGATCTTAGTTTTTATGTAGTCATTCAAAGATTTGGAGATGTTTACTACAACTGATGGTTCCGGGCTCTCCACGCTAATGGGGCTAGTTTCTTTTTTATTGGACTTTATTTGCATATTGCTCGAGGTCTCTATTACGGATCTTACCTTTTCAAAGAAGTTTGGTCCATTGGAGTTGTGATCTTGCTTTTGGTTATAGCAAGGGCCTTCTTGGGCTATGTCCTTCCATGGGGACAAATGTCTTTTTGAGGGGCAACGGTTATTACTAGTCTGTTTTCGGCACTCCCTGTTATTGGAGGAGATGTAGTAGTTTGATTGTGAGGGGGCTTCTCGGTAGACAATGCGACGCTAACTCGATTTTTTGGGCTTCATTTCGTATTACCTTTCATTGTAGCGGGGCTAGTAGGGCTCCACCTATTCTTCTTGCACGACCAAGGGTCGTCAAATCCTTTAGGGGTGAGGTCAGATTTTAATAAGGTTGTTTTTCACCACTATTATGTCCTTAAGGATTTAGTGGGGGCTTGTTTTTTTATTGGCCCTTTACTTGGATTAGTTTACTTCAATCCCTGGCTTCTAGGGGATCCCGAAAATTTTATTGAGGGGAACCCACTGGTTACACCACATCATATTCAACCTGAATGGTATTTTTTATTTGCTTATGCTATTTTGCGGGCTGTGCCTAACAAATTAGGAGGCGTAGTAGCGTTGGCCGCGGCGGTCTTGGTGTTGTTTTATATGCCAGTAAGACCTAAAACTTCTGTTAAAGGGCTATCTTTTTATCCGTGAGGAAAAATGTTGTTTTTTAGGCTTGTTGGAGTGTTTTTTCTGCTTACTTGGGCAGGATCAAAGCCAGTAGAAGAACCATATGTAGTGATTAGACAGGTTTTAAGTTTCTTGTATTTTTCTTATTTTGCCTTAATTGGACCTATCACTCGTATTCAAGACAAGTTGTTGTAA

LJP_f8 3 ATTTATAAGGCTTCCCGATTTAGACCACTAACTAGGATTATTCGGCATACGGTAGTTACTTTGCCAACTCCTATTAACATTTCAACGTGGTGAAATTTTGGTTCCCTCCTGGGAATTTGTTTGGCGAGTCAGATTTTTAGAGGGTTGGTTCTGAGACTCCACTTTACTGCTCATACCGATCTTAGTTTTTATGTAGTCATTCAAAGATTTGGAGATGTTTACTACAACTGATGGTTCCGGGCTCTCCACGCTAATGGGGCTAGTTTCTTTTTTATTGGACTTTATTTGCATATTGCTCGAGGTCTCTATTACGGATCTTACCTTTTCAAAGAAGTTTGGTCCATTGGAGTTGTGATCTTGCTTTTGGTTATAGCAAGGGCCTTCTTGGGCTATGTCCTTCCATGGGGACAAATGTCTTTTTGAGGGGCAACGGTTATTACTAGTCTGTTTTCGGCACTCCCTGTTATTGGAGGAGATGTAGTAGTTTGATTGTGAGGGGGCTTCTCGGTAGACAATGCGACGCTAACTCGATTTTTTGGGCTTCATTTCGTATTACCTTTCATTGTAGCGGGGCTAGTAGGGCTCCACCTATTCTTCTTGCACGACCAAGGGTCGTCAAATCCTTTAGGGGTGAGGTCAGATTTTAATAAGGTTGTTTTTCACCACTATTATGTCCTTAAGGATTTAGTGGGGGCTTGTTTTTTTATTGGCCCTTTACTTGGGTTAGTTTACTTTAATCCCTGGCTTCTAGGGGATCCCGAAAATTTTATTGAGGGGAACCCACTGGTTACACCACATCATATTCAACCTGAATGGTATTTTTTATTTGCTTATGCTATTTTGCGGGCTGTGCCTAACAAATTAGGAGGGGTAGTAGCGTTGGCCGCGGCGGTCTTGGTGTTGTTTTATATACCAGTAAGACCTAAAACTTCTCTTAAAGGGCTATCTTTTTATCCGTGAGGAAAAATGTTGTTTTTTAGGCTTATTGGAGTGTTTTTTCTGCTTACTTGGGCAGGATCGAAGCCAGTAGAAGAACCATATGTAGTGATTAGACAGGTTTTAAGTTTCTTGTATTTTTCTTATTTTGCCTTAATTGGACCTATCACTCGTATTCAAGACAAGTTGTTGTAA

LJP_f1 1 ATTTATAAGGCTTCCCGATTTAGACCACTAACTAGGATTATTCGGCATACGGTAGTTACTTTGCCAACTCCTATTAACATTTCAACGTGGTGAAATTTTGGTTCCCTCCTGGGAATTTGTTTGGCGAGTCAGATTTTTAGAGGGTTGGTTCTGAGACTCCACTTTACTGCTCATACCGATCTTAGTTTTTATGTAGTCATTCAAAGATTTGGAGATGTTTACTACAACTGATGGTTCCGGGCTCTCCACGCTAATGGGGCTAGTTTCTTTTTTATTGGACTTTATTTGCATATTGCTCGAGGTCTCTATTACGGATCTTACCTTTTCAAAGAAGTTTGGTCCATTGGAGTTGTGATCTTGCTTTTGGTTATAGCAAGGGCCTTCTTGGGCTATGTCCTTCCATGGGGACAAATGTCTTTTTGAGGGGCAACGGTTATTACTAGTCTGTTTTCGGCACTCCCTGTTATTGGAGGAGATGTAGTAGTTTGATTGTGAGGGGGCTTCTCGGTAGACAATGCGACGCTAACTCGATTTTTTGGGCTTCATTTCGTATTACCTTTCATTGTAGCGGGACTAGTAGGGCTCCACCTATTCTTCTTGCACGACCAAGGGTCGTCAAATCCTTTAGGGGTGAGGTCAGATTTTAATAAGGTTGTTTTTCACCACTATTATGTCCTTAAGGATTTAGTGGGGGCTTGTTTTTTTATTGGCCCTTTACTTGGATTAGTTTACTTTAATCCTTGGCTTCTAGGGGATCCCGAAAATTTTATTGAGGGGAACCCACTGGTTACACCACATCATATTCAACCTGAATGGTATTTTTTATTTGCTTATGCTATTTTGCGGGCTGTGCCTAACAAATTAGGAGGCGTAGTAGCGTTGGCCGCGGCGGTCTTGGTGTTGTTTTATATGCCAGTAAGACCTAAAACTTCTGTTAAAGGGCTATCTTTTTATCCGTGAGGAAAAATGTTGTTTTTTAGGCTTGTTGGAGTGTTTTTTCTGCTTACTTGGGCAGGATCGAAGCCAGTAGAAGAACCATATGTAGTGATTAGACAGGTTTTAAGTTTCTTGTATTTTTCTTATTTTGCCTTAATTGGACCTATCACTCGTATTCAAGACAAGTTGTTGTAA

LJP_m2 1 ATTTATAAGGCTTCCCGATTTAGACCACTAACTAGGATTATTCGGCATACGGTAGTTACTTTGCCAACTCCTATTAACATTTCAACGTGGTGAAATTTTGGTTCCCTCCTGGGAATTTGTTTGGCGAGTCAGATTTTTAGAGGGTTGGTTCTGAGACTCCACTTTACTGCTCATACCGATCTTAGTTTTTATGTAGTCATTCAAAGATTTGGGGATGTTTACTACAACTGATGGTTCCGGGCTCTCCACGCTAATGGGGCTAGTTTCTTTTTTATTGGACTTTATTTGCATATTGCTCGAGGTCTCTATTACGGATCTTACCTTTTCAAAGAAGTTTGGTCCATTGGAGTTGTGATCTTGCTTTTGGTTATAGCAAGGGCCTTCTTGGGCTATGTCCTTCCATGGGGACAAATGTCTTTTTGAGGGGCAACGGTTATTACTAGTCTGTTTTCGGCACTCCCTGTTATTGGAGGAGATGTAGTAGTTTGATTGTGAGGGGGCTTCTCGGTAGACAATGCGACGCTAACTCGATTTTTTGGGCTTCATTTCGTATTACCTTTCATTGTAGCGGGGCTAGTAGGGCTCCACCTATTCTTCTTGCACGACCAAGGGTCGTCAAATCCTTTAGGGGTGAGGTCAGATTTTAATAAGGTTGTTTTTCACCACTATTATGTCCTTAAGGATTTAGTGGGGGCTTGTTTTTTTATTGGCCCTTTACTTGGATTAGTTTACTTTAATCCCTGGCTTCTAGGGGATCCCGAAAATTTTATTGAGGGGAACCCACTGGTTACACCACATCATATTCAACCTGAATGGTATTTTTTATTTGCTTATGCTATTTTGCGGGCTGTGCCTAACAAATTAGGAGGCGTAGTAGCGTTGGCCGCGGCGGTCTTGGTGTTGTTTTATATGCCAGTAAGACCTAAAACTTCTGTTAAAGGGCTATCTTTTTATCCGTGAGGAAAAATGTTGTTTTTTAGGCTTGTTGGAGTGTTTTTTCTGCTTACTTGGGCAGGATCGAAGCCAGTAGAAGAACCATATGTAGTGATTAGACAGGTTTTAAGTTTCTTGTATTTTTCTTATTTTGCCTTAATTGGACCTATCACTCGTATTCAAGACAAGTTGTTGTAA

LJP_f6 2 ATTTATAAGGCTTCCCGATTTAGACCACTAACTAGGATTATTCGGCATACGGTAGTTACTTTGCCAACTCCTATTAACATTTCAACGTGGTGAAATTTTGGTTCCCTCCTGGGAATTTGTTTGGCGAGTCAGATTTTTAGAGGGTTGGTTCTGAGACTCCACTTTACTGCTCATACCGATCTTAGTTTTTATGTAGTCATTCAAAGATTTGGAGATGTTTACTACAACTGATGGTTCCGGGCTCTCCACGCTAATGGGGCTAGTTTCTTTTTTATTGGACTTTATTTGCATATTGCTCGAGGTCTCTATTACGGATCTTACCTTTTCAAAGAAGTTTGGTCCATTGGAGTTGTGATCTTGCTTTTGGTTATAGCAAGGGCCTTCTTGGGCTATGTCCTTCCATGGGGACAAATGTCTTTTTGAGGGGCAACGGTTATTACTAGTCTGTTTTCGGCACTCCCTGTTATTGGAGGAGATGTAGTAGTTTGATTGTGAGGGGGCTTCTCGGTAGACAATGCGACGCTAACTCGATTTTTTGGGCTTCATTTCGTATTACCTTTCATTGTAGCGGGGCTAGTAGGGCTCCACCTATTCTTCTTGCACGACCAAGGGTCGTCAAATCCTTTAGGGGTGAGGTCAGATTTTAATAAGGTTGTTTTTCACCACTATTATGTCCTTAAGGATTTAGTGGGGGCTTGTTTTTTTATTGGCCCTTTACTTGGATTAGTTTACTTTAATCCCTGGCTTCTAGGGGATCCCGAAAATTTTATTGAGGGGAACCCACTGGTTACACCACATCATATTCAACCTGAATGGTATTTTTTATTTGCTTATGCTATTTTGCGGGCTGTGCCTAACAAATTAGGAGGCGTAGTAGCGTTGGCCGCGGCGGTCTTGGTGTTGTTTTATATGCCAGTAAGACCTAAAACTTCTGTTAAAGGGCTATCTTTTTATCCGTGAGGAAAAATGTTGTTTTTTAGGCTTGTTGGAGTGTTTTTTCTGCTTACTTGGGCAGGATCGAAGCCAGTAGAAGAACCATATGTAGTGATTAGACAGGTTTTAAGTTTCTTGTATTTTTCTTATTTTGCCTTAATTGGACCTATCAGTCGTATTCAAGACAAGTTGTTGTAA

LJP_m3 2 ATTTATAAGGCTTCCCGATTTAGACCACTAACTAGGATTATTCGGCATACGGTAGTTACTTTGCCAACTCCTATTAACATTTCAACGTGGTGAAATTTTGGTTCCCTCCTGGGAATTTGTTTGGCGAGTCAGATTTTTAGAGGGTTGGTTCTGAGACTCCACTTTACTGCTCATACCGATCTTAGTTTTTATGTAGTCATTCAAAGATTTGGAGATGTTTACTACAACTGATGGTTCCGGGCTCTCCACGCTAATGGGGCTAGTTTCTTTTTTATTGGACTTTATTTGCATATTGCTCGAGGTCTCTATTACGGATCTTACCTTTTCAAAGAAGTTTGGTCCATTGGAGTTGTGATCTTGCTTTTGGTTATAGCAAGGGCCTTCTTGGGCTATGTCCTTCCATGGGGACAAATGTCTTTTTGAGGGGCAACGGTTATTACTAGTCTGTTTTCGGCACTCCCTGTTATTGGAGGAGATGTAGTAGTTTGATTGTGAGGGGGCTTCTCGGTAGACAATGCGACGCTAACTCGATTTTTTGGGCTTCATTTCGTATTACCTTTCATTGTAGCGGGGCTAGTAGGGCTCCACCTATTCTTCTTGCACGACCAAGGGTCGTCAAATCCTTTAGGGGTGAGGTCAGATTTTAATAAGGTTGTTTTTCACCACTATTATGTCCTTAAGGATTTAGTGGGGGCTTGTTTTTTTATTGGCCCTTTACTTGGATTAGTTTACTTTAATCCCTGGCTTCTAGGGGATCCCGAAAATTTTATTGAGGGGAACCCACTGGTTACACCACATCATATTCAACCTGAATGGTATTTTTTATTTGCTTATGCTATTTTGCGGGCTGTGCCTAACAAATTAGGAGGCGTAGTAGCGTTGGCCGCGGCGGTCTTGGTGTTGTTTTATATGCCAGTAAGACCTAAAACTTCTGTTAAAGGGCTATCTTTTTATCCGTGAGGAAAAATGTTGTTTTTTAGGCTTGTTGGAGTGTTTTTTCTGCTTACTTGGGCAGGATCGAAGCCAGTAGAAGAACCATATGTAGTGATTAGACAGGTTTTAAGTTTCTTGTATTTTTCTTATTTTGCCTTAATTGGACCTATCACTCGTATTCAAGACAAGTTGTTGTAA

LJP_m19 2 ATTTATAAGGCTTCCCGATTTAGACCACTAACTAGGATTATTCGGCATACGGTAGTTACTTTGCCAACTCCTATTAACATTTCAACGTGGTGAAATTTTGGTTCCCTCCTGGGAATTTGTTTGGCGAGTCAGATTTTTAGAGGGTTGGTTCTGAGACTCCACTTTACTGCTCATACCGATCTTAGTTTTTATGTAGTCATTCAAAGATTTGGAGATGTTTACTACAACTGATGGTTCCGGGCTCTCCACGCTAATGGGGCTAGTTTCTTTTTTATTGGACTTTATTTGCATATTGCTCGAGGTCTCTATTACGGATCTTACCTTTTCAAAGAAGTTTGGTCCATTGGAGTTGTGATCTTGCTTTTGGTTATAGCAAGGGCCTTCTTGGGCTATGTCCTTCCATGGGGACAAATGTCTTTTTGAGGGGCAACGGTTATTACTAGTCTGTTTTCGGCACTCCCTGTTATTGGAGGAGATGTAGTAGTTTGATTGTGAGGGGGCTTCTCGGTAGACAATGCGACGCTAACTCGATTTTTTGGGCTTCATTTCGTATTACCTTTCATTGTAGCGGGGCTAGTAGGGCTCCACCTATTCTTCTTGCACGACCAAGGGTCGTCAAATCCTTTAGGGGTGAGGTCAGATTTTAATAAGGTTGTTTTTCACCACTATTATGTCCTTAAGGATTTAGTGGGGGCTTGTTTTTTTATTGGCCCTTTACTTGGATTAGTTTACTTTAATCCTTGGCTTCTAGGGGATCCCGAAAATTTTATTGAGGGGAACCCACTGGTTACACCACATCATATTCAACCTGAATGGTATTTTTTATTTGCTTATGCTATTTTGCGGGCTGTGCCTAACAAATTAGGAGGCGTAGTAGCGTTGGCCGCGGCGGTCTTGGTGTTGTTTTATATGCCAGTAAGACCTAAAACTTCTGTTAAAGGGCTATCTTTTTATCCGTGAGGAAAAATGTTGTTTTTTAGGCTTGTTGGAGTGTTTTTTCTGCTTACTTGGGCAGGATCGAAGCCAGTAGAAGAACCATATGTAGTGATTAGACAGGTTTTAAGTTTCTTGTATTTTTCTTATTTTGCCTTAATTGGACCTATCACTCGTATTCAAGACAAGTTGTTGTAA }

SampleName="LJP2 9"

SampleSize= 13

SampleData= {

LJP2_m11 1 ATTTATAAGGCTTCCCGATTTAGACCACTAACTAGGATTATTCGGCATACGGTAGTTACTTTGCCAACTCCTATTAACATTTCAACGTGGTGAAATTTTGGTTCCCTCCTGGGAATTTGTTTGGCGAGTCAGATTTTTAGAGGGTTGGTTCTGAGACTCCATTTTACTGCTCATACCGATCTTAGTTTTTATGTAGTCATTCAAAGATTTGGAGATGTTTACTACAACTGATGGTTCCGGGCTCTCCACGCTAATGGGGCTAGTTTCTTTTTTATTGGACTTTATTTGCATATTGCTCGAGGTCTCTATTACGGATCTTACCTTTTCAAAGAAGTTTGGTCCATTGGAGTTGTGATCTTGCTTTTGGTTATAGCAAGGGCCTTCTTGGGCTATGTCCTTCCATGGGGACAAATGTCTTTTTGAGGGGCAACGGTTATTACTAGTCTGTTTTCGGCACTCCCTGTTATTGGAGGAGATGTAGTAGTTTGATTGTGAGGGGGCTTCTCGGTAGACAATGCGACGCTAACTCGATTTTTTGGGCTTCATTTCGTATTACCTTTCATTGTGGCGGGGCTAGTAGGGCTCCACCTATTCTTCTTGCACGACCAAGGGTCGTCAAATCCTTTAGGGGTGAGGTCAGATTTTAATAAGGTTGTTTTTCACCACTATTATGTGCTTAAGGATTTAGTGGGGGCTTGTTTTTTTATTGGTCCTTTACTTGGATTAGTTTACTTTAATCCCTGGCTTCTAGGGGATCCCGAAAATTTTATTGAGGGGAACCCACTGGTTACACCACATCATATTCAACCTGAATGGTATTTTTTATTTGCTTATGCTATTTTGCGGGCTGTGCCTAACAAATTAGGAGGAGTAGTAGCGTTGGCCGCGGCGGTCTTGGTGTTGTTTTATATGCCAGTAAGACCTAAAACTTCTGTTAAAGGGCTATCTTTTTATCCGTGAGGAAAAATGTTATTTTTTAGGCTTGTTGGAGTGTTTTTTCTGCTTACTTGGGCAGGATCGAAGCCAGTAGAAGAACCATATGTAGTGATTAGACAGGTTTTAAGTTTCTTGTATTTTTCTTATTTTGCCTTAATTGGACCTATCACTCGTATTCAAGACAAGTTGTTGTAA

LJP2_f12 1 ATTTATAAGGCTTCCCGATTTAGACCACTAACTAGGATTATTCGGCATACGGTAGTTACTTTGCCAACTCCTATTAACATTTCAACGTGGTGAAATTTTGGTTCCCTCCTGGGAATTTGTTTGGCGAGTCAGATTTTTAGAGGGTTGGTTCTGAGACTCCATTTTACTGCTCATACCGATCTTAGTTTTTATGTAGTCATTCAAAGATTTGGAGATGTTTACTACAACTGATGGTTCCGGGCTCTCCACGCTAATGGGGCTAGTTTCTTTTTTATTGGACTTTATTTGCATATTGCTCGAGGTCTCTATTACGGATCTTACCTTTTCAAAGAAGTTTGGTCCATTGGAGTTGTGATCTTGCTTTTGGTTATAGCAAGGGCCTTCTTGGGCTATGTCCTTCCATGGGGACAAATGTCTTTTTGAGGGGCAACGGTTATTACTAGTCTGTTTTCGGCACTCCCTGTTATTGGAGGAGATGTAGTAGTTTGATTGTGAGGGGGCTTCTCGGTAGACAATGCGACGCTAACTCGATTTTTTGGGCTTCATTTCGTATTACCTTTCATTGTGGCGGGGCTAGTAGGGCTCCACCTATTCTTCTTGCACGACCAAGGGTCGTCAAATCCTTTAGGGGTGAGGTCAGATTTTAATAAGGTTGTTTTTCACCACTATTATGTGCTTAAGGATTTAGTGGGGGCTTGTTTTTTTATTGGCCCTTTACTTGGATTAGTTTACTTTAATCCGTGGCTTCTAGGGGATCCCGAAAATTTTATTGAGGGGAACCCACTGGTTACACCACATCATATTCAACCTGAATGGTATTTTTTATTTGCTTATGCTATTTTGCGGGCTGTGCCTAACAAATTAGGAGGAGTAGTAGCGTTGGCCGCGGCGGTCTTGGTGTTGTTTTATATGCCAGTAAGACCTAAAACTTCTGTTAAAGGGCTATCTTTTTATCCGTGAGGAAAAATGTTATTTTTTAGGCTTGTTGGAGTGTTTTTTCTGCTTACTTGGGCAGGATCGAAGCCAGTAGAAGAACCATATGTAGTGATTAGACAGGTTTTAAGTTTCTTGTATTTTTCTTATTTTGCCTTAATTGGACCTATCACTCGTATTCAAGACAAGTTGTTGTAA

LJP2_f16 2 ATTTATAAGGCTTCCCGATTTAGACCACTAACTAGGATTATTCGGCATACGGTAGTTACTTTGCCAACTCCTATTAACATTTCAACGTGGTGAAATTTTGGTTCCCTCCTGGGAATTTGTTTGGCGAGTCAGATTTTTAGAGGGTTGGTTCTGAGACTCCACTTTACTGCTCATACCGATCTTAGTTTTTATGTAGTCATTCAAAGATTTGGAGATGTTTACTACAACTGATGGTTCCGGGCTCTCCACGCTAATGGGGCTAGTTTCTTTTTTATTGGACTTTATTTGCATATTGCTCGAGGTCTCTATTACGGATCTTACCTTTTCAAAGAAGTTTGGTCCATTGGAGTTGTGATCTTGCTTTTGGTTATAGCAAGGGCCTTCTTGGGCTATGTCCTTCCATGGGGACAAATGTCTTTTTGAGGGGCAACGGTTATTACTAGTCTGTTTTCGGCACTCCCTGTTATTGGAGGAGATGTAGTAGTTTGATTGTGAGGGGGCTTCTCGGTAGACAATGCGACGCTAACTCGATTTTTTGGGCTTCATTTCGTATTACCTTTCATTGTAGCGGGGCTAGTAGGGCTCCACCTATTCTTCTTGCACGACCAAGGGTCGTCAAATCCTTTAGGGGTGAGGTCAGATTTTAATAAGGTTGTTTTTCACCACTATTATGTCCTTAAGGATTTAGTGGGGGCTTGTTTTTTTATTGGCCCTTTACTTGGGTTAGTTTACTTTAATCCCTGGCTTCTAGGGGATCCCGAAAATTTTATTGAGGGGAACCCACTGGTTACACCACATCATATTCAACCTGAATGGTATTTTTTATTTGCTTATGCTATTTTGCGGGCTGTGCCTAACAAATTAGGAGGGGTAGTAGCGTTGGCCGCGGCGGTCTTGGTGTTGTTTTATATACCAGTAAGACCTAAAACTTCTGTTAAAGGGCTATCTTTTTATCCGTGAGGAAAAATGTTGTTTTTTAGGCTTATTGGAGTGTTTTTTCTGCTTACTTGGGCAGGATCGAAGCCAGTAGAAGAACCATATGTAGTGATTAGACAGGTTTTAAGTTTCTTGTATTTTTCTTATTTTGCCTTAATTGGACCTATCACTCGTATTCAAGACAAGTTGTTGTAA

LJP2_f1 1 ATTTATAAGGCTTCCCGATTTAGACCACTAACTAGGATTATTCGGCATACGGTAGTCACTTTGCCAACTCCTATTAACATTTCAACGTGGTGAAATTTTGGTTCCCTCCTGGGAATTTGTTTGGCGAGTCAGATTTTTAGAGGGTTGGTTCTGAGACTCCACTTTACTGCTCATACCGATCTTAGTTTTTATGTAGTCATTCAAAGATTTGGAGATGTTTACTACAACTGATGGTTCCGGGCTCTCCACGCTAATGGGGCTAGTTTCTTTTTTATTGGACTTTATTTGCATATTGCTCGAGGTCTCTATTACGGATCTTACCTTTTCAAAGAAGTTTGGTCCATTGGAGTTGTGATCTTGCTTTTGGTTATAGCAAGGGCCTTCTTGGGCTATGTCCTTCCATGGGGACAAATGTCTTTTTGAGGGGCAACAGTTATTACTAGTCTGTTTTCGGCACTCCCTGTTATTGGAGGAGATGTAGTAGTTTGATTGTGAGGGGGCTTCTCGGTAGACAATGCGACGCTAACTCGATTTTTTGGGCTTCATTTCGTATTACCTTTCATTGTGGCGGGGCTAGTAGGGCTCCACCTATTCTTCTTGCACGACCAAGGGTCGTCAAATCCTTTAGGGGTGAGGTCAGATTTTAATAAGGTTGTTTTTCACCACTATTATGTGCTTAAGGATTTAGTGGGGGCTTGTTTTTTTATTGGCCCTTTACTTGGATTAGTTTACTTTAATCCCTGGCTTCTAGGGGATCCCGAAAATTTTATTGAGGGGAACCCACTGGTTACACCACATCATATTCAACCTGAATGGTATTTTTTATTTGCTTATGCTATTTTGCGGGCTGTGCCTAACAAATTAGGAGGAGTAGTAGCGTTGGCCGCGGCGGTCTTGGTGTTGTTTTATATGCCAGTAAGACCTAAAACTTCTGTTAAAGGGCTATCTTTTTATCCGTGAGGAAAAATGTTATTTTTTAGGCTTGTTGGAGTGTTTTTTCTGCTTACTTGGGCAGGATCGAAGCCAGTAGAAGAACCATATGTAGTGATTAGACAGGTTTTAAGTTTCTTGTATTTTTCTTATTTTGCCTTAATTGGACCTATCACTCGTATTCAAGACAAGTTGTTGTAA

LJP2_m12 8 ATTTATAAGGCTTCCCGATTTAGACCACTAACTAGGATTATTCGGCATACGGTAGTTACTTTGCCAACTCCTATTAACATTTCAACGTGGTGAAATTTTGGTTCCCTCCTGGGAATTTGTTTGGCGAGTCAGATTTTTAGAGGGTTGGTTCTGAGACTCCATTTTACTGCTCATACCGATCTTAGTTTTTATGTAGTCATTCAAAGATTTGGAGATGTTTACTACAACTGATGGTTCCGGGCTCTCCACGCTAATGGGGCTAGTTTCTTTTTTATTGGACTTTATTTGCATATTGCTCGAGGTCTCTATTACGGATCTTACCTTTTCAAAGAAGTTTGGTCCATTGGAGTTGTGATCTTGCTTTTGGTTATAGCAAGGGCCTTCTTGGGCTATGTCCTTCCATGGGGACAAATGTCTTTTTGAGGGGCAACGGTTATTACTAGTCTGTTTTCGGCACTCCCTGTTATTGGAGGAGATGTAGTAGTTTGATTGTGAGGGGGCTTCTCGGTAGACAATGCGACGCTAACTCGATTTTTTGGGCTTCATTTCGTATTACCTTTCATTGTGGCGGGGCTAGTAGGGCTCCACCTATTCTTCTTGCACGACCAAGGGTCGTCAAATCCTTTAGGGGTGAGGTCAGATTTTAATAAGGTTGTTTTTCACCACTATTATGTGCTTAAGGATTTAGTGGGGGCTTGTTTTTTTATTGGCCCTTTACTTGGATTAGTTTACTTTAATCCCTGGCTTCTAGGGGATCCCGAAAATTTTATTGAGGGGAACCCACTGGTTACACCACATCATATTCAACCTGAATGGTATTTTTTATTTGCTTATGCTATTTTGCGGGCTGTGCCTAACAAATTAGGAGGAGTAGTAGCGTTGGCCGCGGCGGTCTTGGTGTTGTTTTATATGCCAGTAAGACCTAAAACTTCTGTTAAAGGGCTATCTTTTTATCCGTGAGGAAAAATGTTATTTTTTAGGCTTGTTGGAGTGTTTTTTCTGCTTACTTGGGCAGGATCGAAGCCAGTAGAAGAACCATATGTAGTGATTAGACAGGTTTTAAGTTTCTTGTATTTTTCTTATTTTGCCTTAATTGGACCTATCACTCGTATTCAAGACAAGTTGTTGTAA

}

SampleName="LJP3 10"

SampleSize= 10

SampleData= {

LJS_f3 10

}

SampleName="LJP04 11"

SampleSize= 6

SampleData= {

LJP_m2 1

LJP_m19 3

LJP04_m2 2 ATTTATAAGGCTTCCCGATTTAGACCACTAACTAGGATTATTCGGCATACGGTAGTTACTTTGCCAACTCCTATTAACATTTCAACGTGGTGAAATTTTGGTTCCCTCCTGGGAATTTGTTTGGCGAGTCAGATTTTTAGAGGGTTGGTTCTGAGACTCCACTTTACTGCTCATACCGATCTTAGTTTTTATGTAGTCATTCAAAGATTTGGAGATGTTTACTATAACTGATGGTTCCGGGCTCTCCACGCTAATGGGGCTAGTTTCTTTTTTATTGGACTTTATTTGCATATTGCTCGAGGTCTCTATTACGGATCTTACCTTTTCAAAGAAGTTTGGTCCATTGGAGTTGTGATCTTGCTTTTGGTTATAGCAAGGGCCTTCTTGGGCTATGTCCTTCCATGGGGACAAATGTCTTTTTGAGGGGCAACGGTTATTACTAGTCTGTTTTCGGCACTCCCTGTTATTGGAGGAGATGTAGTAGTTTGATTGTGAGGGGGCTTCTCGGTAGACAATGCGACGCTAACTCGATTTTTTGGGCTTCATTTCGTATTACCTTTCATTGTAGCGGGGCTAGTAGGGCTCCACCTATTCTTCTTGCACGACCAAGGGTCGTCAAATCCTTTAGGGGTGAGGTCAGATTTTAATAAGGTTGTTTTTCACCACTATTATGTCCTTAAGGATTTAGTGGGGGCTTGTTTTTTTATTGGCCCTTTACTTGGATTAGTTTACTTTAATCCCTGGCTTCTAGGGGATCCCGAAAATTTTATTGAGGGGAACCCACTGGTTACACCACATCATATTCAACCTGAATGGTATTTTTTATTTGCTTATGCTATTTTGCGGGCTGTGCCTAACAAATTAGGAGGCGTAGTAGCGTTGGCCGCGGCGGTCTTGGTGTTGTTTTATATGCCAGTAAGACCTAAAACTTCTGTTAAAGGGCTATCTTTTTATCCGTGAGGAAAAATGTTGTTTTTTAGGCTTGTTGGAGTGTTTTTTCTGCTTACTTGGGCAGGATCGAAGCCAGTAGAAGAACCATATGTAGTGATTAGACAGGTTTTAAGTTTCTTGTATTTTTCTTATTTTGCCTTAATTGGACCTATCACTCGTATTCAAGACAAGTTGTTGTAA }

SampleName="RP1 12"

SampleSize= 10

SampleData= {

RP1_f5 5 ATTTATAAGGTTTCCCGGTTTAGGCCGTTAGTATCAGTAGTCCGGCACACCGTTATTACTTTACCTACCCCTATTAATATTTCAACCTGGTGAAATTTTGGTTCACTCCTAGGGGTTTGTCTAGTAATCCAAATTTTTAGGGGGTTAATTTTAAGGCTTCATTTTACGGCTCATACAGATCTCAGATTTTATGTGGTCATTCAAAGGTTTGGAGATGTCTACTACAATTGGTGGTTTCGTGCTTTTCATGCCAATGGTGCTAGGTTCTTTTTTATTGGTTTGTACTTACACATTGCGCGAGGGCTATACTACGGATCTTATGTTTTTAAGGAAGTATGATCTGTTGGAGTGGTAATTTTGCTGGTAGTTATAGCTAGGGCCTTTTTAGGCTATGTTCTCCCATGAGGACAAATATCTTTTTGGGGGGCAACGGTTATTACAAGTTTATTTTCAGCACTCCCCGTTATTGGGAGAGATGTGGTAATCTGATTGTGAGGAGGGTTTTCAGTAGACAACGCTACTCTTACCCGATTTTTTGGGCTTCATTTTGTGTTGCCTTTTATGGTAGCAGGGTTAGCAGGATTGCACCTGTTCTTCCTTCACGACCAAGGGTCGTCAAATCCATTGGGGGTAAGGTCTGATTACAACAAGGTTGTTTTTCATCATTACTATGTGCTTAAAGATTTAGTGGGGGTTTGTTTCTTTTTAGGGCCTTTGCTCGGTCTGGTGTATTTTAACCCCTGGCTTCTGGGGGACCCTGAAAATTTTATTGAAGGAAACCCACTGGTTACACCTCACCATATTCAGCCGGAATGATATTTTCTATTCGCCTACGCTATTTTGCGGGCTGTGCCTAATAAGCTTGGGGGGGTAGTCGCCTTGGCGGCTGCGGTGCTGGTATTATTTTATATACCCTTTAGTCCTAAAACATCGATCAAAGGGTTATCCTTTTACCCGGGGGGTAAGAGTTTGTTTTTTAGAATAATTGGGGTGTTTTTTCTGCTCACCTGGGCGGGCTCTAAGCCGGTTGAAGAGCCCTATGTTGTAATTAGGCAAGTTCTAAGGTTTTTATATTTTGCGTATTTTATTTTAATTGGGCCCGTAACACGGGTTCAAGATAAGTTGCTGTAA

RP1_m8 1 ATTTATAAGGTTTCCCGGTTTAGGCCGTTAGTATCAGTAGTCCGGCACACCGTTATTACTTTACCTACCCCTATTAATATTTCAACCTGGTGAAATTTTGGTTCACTCCTAGGGGTTTGTCTAGTAATCCAAATTTTTAGGGGGTTAATTTTAAGGCTTCATTTTACGGCTCATACAGATCTCAGATTTTATGTGGTCATTCAAAGGTTTGGAGATGTCTATTACAATTGGTGGTTTCGTGCTTTTCATGCCAATGGTGCTAGGTTCTTTTTTATTGGTTTGTACTTACACATTGCGCGAGGGCTATACTACGGATCTTATGTTTTTAAGGAAGTATGATCTGTTGGAGTGGTAATTTTGCTGGTAGTTATAGCTAGGGCCTTTTTAGGCTATGTTCTCCCATGAGGACAAATATCTTTTTGGGGGGCAACGGTTATTACAAGTTTATTTTCAGCACTCCCCGTTATTGGGAGAGATGTGGTAATCTGATTGTGAGGAGGGTTTTCAGTAGACAACGCTACTCTTACCCGATTTTTTGGGCTTCATTTTGTGTTGCCTTTTATGGTAGCAGGGTTAGCAGGATTGCACCTGTTCTTCCTTCACGACCAAGGGTCGTCAAATCCATTGGGGGTAAGGTCTGATTACAACAAGGTTGTTTTTCATCATTACTATGTGCTTAAAGATTTAGTGGGGGTTTGTTTCTTTTTAGGGCCTTTGCTCGGTCTGGTATATTTTAACCCCTGGCTTCTGGGGGACCCTGAAAATTTTATTGAAGGAAACCCACTGGTTACACCCCACCATATTCAGCCGGAATGATATTTTCTATTCGCCTACGCTATTTTGCGGGCTGTGCCTAATAAGCTTGGGGGGGTAGTCGCCTTGGCGGCTGCGGTGCTGGTATTATTTTATATACCCTTTAGTCCTAAAACATCGATCAAAGGGTTATCCTTTTACCCGGGGGGTAAGAGTTTGTTTTTTAGAATAATTGGGGTGTTTTTTCTGCTCACCTGGGCGGGCTCTAAGCCGGTTGAAGAGCCCTATGTTGTAATTAGGCAAGTTCTAAGGTTTTTATATTTTGCGTATTTTATTTTAATTGGGCCCGTAACACGGGTTCAAGATAAGTTGCTGTAA

RP1_f4 1 ATTTATAAGGCTTCCCGGTTTAGGCCGTTAGTATCAGTAGTCCGGCACACCGTTATTACTTTACCTACCCCTATTAATATTTCAACCTGGTGAAATTTTGGTTCACTCCTAGGGGTTTGTCTAGTAATCCAAATTTTTAGGGGGTTAATTTTAAGGCTTCATTTCACGGCTCATACAGATCTCAGATTTTATGTGGTCATTCAAAGGTTTGGAGATGTCTACTACAATTGGTGGTTTCGTGCTTTTCATGCCAATGGTGCTAGGTTCTTTTTTATTGGTTTGTACTTACACATTGGGCGAGGGCTATACTACGGATCTTATGTTTTTAAGGAAGTATGATCTGTTGGAGTGGTAATTTTGCTGGTAGTTATAGCTAGGGCCTTTTTAGGCTATGTTCTCCCATGAGGACAAATATCTTTTTGGGGGGCAACGGTTATTACAAGTTTATTTTCAGCACTCCCCGTTATTGGGAGAGATGTGGTAATCTGATTGTGAGGAGGGTTTTCAGTAGACAACGCTACTCTTACCCGATTTTTTGGGCTTCATTTTGTGTTGCCTTTTATGGTAGCAGGGTTAGCAGGATTGCACCTGTTCTTCCTTCACGACCAAGGGTCGTCAAATCCATTGGGGGTAAGGTCTGATTACAACAAGGTTGTTTTTCATCATTACTATGTGCTTAAAGATTTAGTGGGGGTTTGTTTCTTTTTAGGGCCTTTGCTCGGTCTGGTGTATTTTAACCCCTGGCTTTTGGGGGACCCTGAAAATTTTATTGAAGGAAACCCACTGGTTACACCCCACCATATTCAGCCGGAATGATATTTTCTATTCGCCTACGCTATTTTGCGGGCTGTGCCTAATAAGCTTGGGGGGGTAGTCGCCTTGGCGGCTGCGGTGCTGGTATTATTTTATATACCCTTTAGTCCTAAAACATCGATCAAAGGGTTATCCTTTTACCCGGGGGGTAAGAGTTTGTTTTTTAGAATAATTGGGGTGTTTTTTCTGCTCACCTGGGCGGGCTCTAAGCCGGTTGAGGAGCCCTATGTTGTAATTAGGCAAGTTCTAAGGTTTTTATATTTTGCGTATTTTATTTTAATTGGGCCCGTAACACGGGTTCAAGATAAGTTGCTGTAA

RP1_f1 1 ATTTATAAGGTTTCCCGGTTTAGGCCGTTAGTATCAGTAGTCCGGCACACAGTTATTACTTTACCTACCCCTATTAATATTTCAACCTGGTGAAATTTTGGTTCACTCCTAGGGGTTTGTCTAGTAATCCAAATTTTTAGGGGGTTAATTTTAAGGCTTCATTTTACGGCTCATACAGATCTCAGATTTTATGTGGTCATTCAAAGGTTTGGAGATGTCTACTACAATTGGTGGTTTCGTGCTTTTCATGCCAATGGTGCTAGGTTCTTTTTTATTGGTTTGTACTTACACATTGCGCGAGGGCTATACTACGGATCTTATGTTTTTAAGGAAGTATGATCTGTTGGAGTGGTAATTTTGCTGGTAGTTATAGCTAGGGCCTTTTTAGGCTATGTTCTCCCATGAGGACAAATATCTTTTTGGGGGGCAACGGTTATTACAAGTTTATTTTCAGCACTCCCCGTTATTGGGAGAGATGTGGTAATCTGATTGTGAGGAGGGTTTTCAGTAGACAACGCTACTCTTACCCGATTTTTTGGGCTTCATTTTGTGTTGCCTTTTATGGTAGCAGGGTTAGCAGGATTGCACCTGTTCTTCCTTCACGACCAAGGGTCGTCAAATCCATTGGGGGTAAGGTCTGATTACAACAAGGTTGTTTTTCATCATTACTATGTGCTTAAAGATTTAGTGGGGGTTTGTTTCTTTTTAGGGCCTTTGCTCGGTCTGGTGTATTTTAACCCCTGGCTTCTGGGGGACCCTGAAAATTTTATTGAAGGAAACCCACTGGTTACACCCCACCATATTCAGCCGGAATGATATTTTCTATTCGCCTACGCTATTTTGCGGGCTGTGCCTAATAAGCTTGGGGGGGTAGTCGCCTTGGCGGCTGCGGTGTTGGTATTATTTTATATACCCTTTAGTCCTAAAACATCGATCAAAGGGTTATCCTTTTACCCGGGGGGTAAGAGTTTGTTTTTTAGAATAATTGGGGTGTTTTTTCTGCTCACCTGGGCGGGCTCTAAGCCGGTTGAAGAGCCCTATGTTGTAATTAGGCAAGTTCTAAGGTTTTTATATTTTGCGTATTTTATTTTAATTGGGCCCGTAACACGGGTTCAAGATAAGTTGCTGTAA

RP1_f3 1 ATTTATAAGGCTTCCCGGTTTAGGCCGTTAGTATCAGTAGTCCGGCACACCGTTATTACTTTACCCACCCCTATTAATATTTCAACCTGGTGAAATTTTGGTTCACTCCTAGGGGTTTGTCTAGTAATCCAAATTTTTAGGGGGTTAATTTTAAGGCTTCATTTCACGGCTCATACAGATCTCAGATTTTATGTGGTCATTCAAAGGTTTGGAGATGTCTACTACAATTGGTGGTTTCGTGCTTTTCATGCCAATGGTGCTAGGTTCTTTTTTATTGGTTTGTACTTACACATTGGGCGAGGGCTATACTACGGATCTTATGTTTTTAAGGAAGTATGATCTGTTGGAGTGGTAATTTTGCTGGTAGTTATAGCTAGGGCCTTTTTAGGCTATGTTCTCCCATGAGGACAAATATCTTTTTGGGGGGCAACGGTTATTACAAGTTTATTTTCAGCACTCCCCGTTATTGGGAGAGATGTGGTAATCTGATTGTGAGGAGGGTTTTCAGTAGACAACGCTACTCTTACCCGATTTTTTGGGCTTCATTTTGTGTTGCCTTTTATGGTAGCAGGGTTAGCAGGATTGCACCTGTTCTTCCTTCACGACCAAGGGTCGTCAAATCCATTGGGGGTAAGGTCTGATTACAACAAGGTTGTTTTTCATCATTACTATGTGCTTAAAGATTTAGTGGGGGTTTGTTTCTTTTTAGGGCCTTTGCTCGGTCTGGTGTATTTTAACCCCTGGCTTTTGGGGGACCCTGAAAATTTTATTGAAGGAAACCCACTGGTTACACCCCACCATATTCAGCCGGAATGATATTTTCTATTCGCCTACGCTATTTTGCGGGCTGTGCCTAATAAGCTTGGGGGGGTAGTCGCCTTGGCGGCTGCGGTGCTGGTATTATTTTATATACCCTTTAGTCCTAAAACATCGATCAAAGGGTTATCCTTTTACCCGGGGGGTAAGAGTTTGTTTTTTAGAATAATTGGGGTGTTTTTTCTGCTCACCTGGGCGGGCTCTAAGCCGGTTGAGGAGCCCTATGTTGTAATTAGGCAAGTTCTAAGGTTTTTATATTTTGCGTATTTTATTTTAATTGGGCCCGTAACACGGGTTCAAGATAAGTTGCTGTAA

RP1_m6 1 ATTTATAAGGTTTCCCGGTTTAGGCCGTTAGTATCAGTAGTCCGGCACACCGTTATTACTTTACCTACCCCTATTAATATTTCAACCTGGTGAAATTTTGGTTCACTCCTAGGGGTTTGTCTAGTAATCCAAATTTTTAGGGGGTTAATTTTAAGGCTTCATTTTACGGCTCATACAGATCTCAGATTTTATGTGGTCATTCAAAGGTTTGGAGATGTCTATTACAATTGGTGGTTTCGTGCTTTTCATGCCAATGGTGCTAGGTTCTTTTTTATTGGTTTGTACTTACACATTGCGCGAGGGCTATACTACGGATCTTATGTTTTTAAGGAAGTATGATCTGTTGGAGTGGTAATTTTGCTGGTAGTTATAGCTAGGGCCTTTTTAGGCTATGTTCTCCCATGAGGACAAATATCTTTTTGGGGGGCAACGGTTATTACAAGTTTATTTTCAGCACTCCCCGTTATTGGGAGAGATGTGGTAATCTGATTGTGAGGAGGGTTTTCAGTAGACAACGCTACTCTTACCCGATTTTTTGGGCTTCATTTTGTGTTGCCTTTTATGGTAGCAGGGTTAGCAGGATTGCACCTGTTCTTCCTTCACGACCAAGGGTCGTCAAATCCATTGGGGGTAAGGTCTGATTACAACAAGGTTGTTTTTCATCATTACTATGTGCTTAAAGATTTAGTGGGGGTTTGTTTCTTTTTAGGGCCTTTGCTCGGTCTGGTGTATTTTAACCCCTGGCTTCTGGGGGACCCTGAAAATTTTATTGAAGGAAACCCACTGGTTACACCCCACCATATTCAGCCGGAATGATATTTTCTATTCGCCTACGCTATTTTGCGGGCTGTGCCTAATAAGCTTGGGGGGGTAGTCGCCTTGGCGGCTGCGGTGCTGGTATTATTTTATATACCCTTTAGTCCTAAAACATCGATCAAAGGGTTATCCTTTTACCCGGGGGGTAAGAGTTTGTTTTTTAGAATAATTGGGGTGTTTTTTCTGCTCACCTGGGCGGGCTCTAAGCCGGTTGAAGAGCCCTATGTTGTAATTAGGCAAGTTCTAAGGTTTTTATATTTTGCGTATTTTATTTTAATTGGGCCCGTAACACGGGTTCAAGATAAGTTGCTGTAA

}

SampleName="RP2 13"

SampleSize= 10

SampleData= {

RP2_m7 1 ATTTA?AAGG?TTCCCGGTTTAGGCCGTTAGTATCAGTAGTCCGGCACACCGTTATTACTTTACCTACCCCTATTAATATTTCAACCTGGTGAAATTTTGGTTCACTCCTAGGGGTTTGTCTAGTAATCCAAATTTTTAGGGGGTTAATTTTAAGGCTTCATTTTACGGCTCATACAGATCTCAGATTTTATGTGGTCATTCAAAGGTTTGGAGATGTCTACTACAATTGGTGGTTTCGTGCTTTTCATGCCAATGGTGCTAGGTTCTTTTTTATTGGTTTGTACTTACACATTGCGCGAGGGCTATACTACGGATCTTATGTTTTTAAGGAAGTATGATCTGTTGGAGTGGTAATTTTGCTGGTAGTTATAGCTAGGGCCTTTTTAGGCTATGTTCTCCCATGAGGACAAATATCTTTTTGGGGGGCAACGGTTATTACAAGTTTATTTTCAGCACTCCCCGTTATTGGGAGAGATGTGGTAATCTGATTGTGAGGAGGGTTTTCAGTAGACAACGCTACTCTTACCCGATTTTTTGGGCTTCATTTTGTGTTGCCTTTTATGGTAGCAGGGTTAGCAGGATTGCACCTGTTCTTCCTTCACGACCAAGGGTCGTCAAATCCATTGGGGGTAAGGTCTGATTACAACAAGGTTGTTTTTCATCATTACTATGTGCTTAAAGATTTAGTGGGGGTTTGTCTCTTTTTAGGGCCTTTGCTCGGTCTGGTGTATTTTAACCCCTGGCTTCTGGGGGACCCTGAAAATTTTATTGAAGGAAACCCACTGGTTACACCCCACCATATTCAGCCGGAATGATATTTTCTATTCGCCTACGCTATTTTGCGGGCTGTGCCTAATAAGCTTGGGGGGGTAGTCGCCTTGGCGGCTGCGGTGCTGGTATTATTTTATATACCCTTTAGTCCTAAAACATCGATCAAAGGGTTATCCTTTTACCCGGGGGGTAAGAGTTTGTTTTTTAGAATAATTGGGGTGTTTTTTCTGCTCACCTGGGCGGGCTCTAAGCCGGTTGAAGAGCCCTATGTTGTAATTAGGCAAGTTCTAAGGTTTTTATATTTTGCGTATTTTATTTTAATTGGGCCCGTAACACGGGTTCAAGATAAGTTGCTGTAA

RP2_m2 1 ATTTA?AAGG?TTCCCGGTTTA?GCCG??AGTATCAGTAGTCCGGCACACCGTTATTACTTTACCTACCCCTATTAATATTTCAACCTGGTGAAATTTTGGTTCACTCCTAGGGGTTTGTCTAGTAATCCAAATTTTTAGGGGGTTAATTTTAAGGCTTCATTTTACGGCTCATACAGATCTCAGATTTTATGTGGTCATTCAAAGGTTTGGAGATGTCTACTACAATTGGTGGTTTCGTGCTTTTCATGCCAATGGTGCTAGGTTCTTTTTTATTGGTTTGTACTTACACATTGCGCGAGGGCTATACTACGGATCTTATGTTTTTAAGGAAGTATGATCTGTTGGAGTGGTAATTTTGCTGGTAGTTATAGCTAGGGCCTTTTTAGGCTATGTTCTCCCATGAGGACAAATATCTTTTTGGGGGGCAACGGTTATTACAAGTTTATTTTCAGCACTCCCCGTTATTGGGAGAGATGTAGTAATCTGATTGTGAGGAGGGTTTTCAGTAGACAACGCTACTCTTACCCGATTTTTTGGGCTTCATTTTGTGTTGCCTTTTATGGTAGCAGGGTTAGCAGGATTGCACCTGTTCTTCCTTCACGACCAAGGGTCGTCAAATCCATTGGGGGTAAGGTCTGATTACAACAAGGTTGTTTTTCATCATTACTATGTGCTTAAAGATTTAGTGGGGGTTTGTTTCTTTTTAGGGCCTTTGCTCGGTCTGGTGTATTTTAACCCCTGGCTTCTGGGGGACCCTGAAAATTTTATTGAAGGAAACCCACTGGTTACACCCCACCATATTCAGCCGGAATGATATTTTCTATTCGCCTACGCTATTTTGCGGGCTGTGCCTAATAAGCTTGGGGGGGTAGTCGCCTTGGCGGCTGCGGTGCTGGTATTATTTTATATACCCTTTAGTCCTAAAACATCGATCAAAGGGTTATCCTTTTACCCGGGGGGTAAGAGTTTGTTTTTTAGAATAATTGGGGTGTTTTTTCTGCTCACCTGGGCGGGCTCTAAGCCGGTTGAAGAGCCCTATGTTGTAATTAGGCAAGTTCTAAGGTTTTTATATTTTGCGTATTTTATTTTAATTGGGCCCGTAACACGGGTTCAAGATAAGTTGCTGTAA

RP1_f1 5 ATTTATAAGGTTTCCCGGTTTAGGCCGTTAGTATCAGTAGTCCGGCACACAGTTATTACTTTACCTACCCCTATTAATATTTCAACCTGGTGAAATTTTGGTTCACTCCTAGGGGTTTGTCTAGTAATCCAAATTTTTAGGGGGTTAATTTTAAGGCTTCATTTTACGGCTCATACAGATCTCAGATTTTATGTGGTCATTCAAAGGTTTGGAGATGTCTACTACAATTGGTGGTTTCGTGCTTTTCATGCCAATGGTGCTAGGTTCTTTTTTATTGGTTTGTACTTACACATTGCGCGAGGGCTATACTACGGATCTTATGTTTTTAAGGAAGTATGATCTGTTGGAGTGGTAATTTTGCTGGTAGTTATAGCTAGGGCCTTTTTAGGCTATGTTCTCCCATGAGGACAAATATCTTTTTGGGGGGCAACGGTTATTACAAGTTTATTTTCAGCACTCCCCGTTATTGGGAGAGATGTGGTAATCTGATTGTGAGGAGGGTTTTCAGTAGACAACGCTACTCTTACCCGATTTTTTGGGCTTCATTTTGTGTTGCCTTTTATGGTAGCAGGGTTAGCAGGATTGCACCTGTTCTTCCTTCACGACCAAGGGTCGTCAAATCCATTGGGGGTAAGGTCTGATTACAACAAGGTTGTTTTTCATCATTACTATGTGCTTAAAGATTTAGTGGGGGTTTGTTTCTTTTTAGGGCCTTTGCTCGGTCTGGTGTATTTTAACCCCTGGCTTCTGGGGGACCCTGAAAATTTTATTGAAGGAAACCCACTGGTTACACCCCACCATATTCAGCCGGAATGATATTTTCTATTCGCCTACGCTATTTTGCGGGCTGTGCCTAATAAGCTTGGGGGGGTAGTCGCCTTGGCGGCTGCGGTGTTGGTATTATTTTATATACCCTTTAGTCCTAAAACATCGATCAAAGGGTTATCCTTTTACCCGGGGGGTAAGAGTTTGTTTTTTAGAATAATTGGGGTGTTTTTTCTGCTCACCTGGGCGGGCTCTAAGCCGGTTGAAGAGCCCTATGTTGTAATTAGGCAAGTTCTAAGGTTTTTATATTTTGCGTATTTTATTTTAATTGGGCCCGTAACACGGGTTCAAGATAAGTTGCTGTAA

RP1_f4 2 ATTTATAAGGCTTCCCGGTTTAGGCCGTTAGTATCAGTAGTCCGGCACACCGTTATTACTTTACCTACCCCTATTAATATTTCAACCTGGTGAAATTTTGGTTCACTCCTAGGGGTTTGTCTAGTAATCCAAATTTTTAGGGGGTTAATTTTAAGGCTTCATTTCACGGCTCATACAGATCTCAGATTTTATGTGGTCATTCAAAGGTTTGGAGATGTCTACTACAATTGGTGGTTTCGTGCTTTTCATGCCAATGGTGCTAGGTTCTTTTTTATTGGTTTGTACTTACACATTGGGCGAGGGCTATACTACGGATCTTATGTTTTTAAGGAAGTATGATCTGTTGGAGTGGTAATTTTGCTGGTAGTTATAGCTAGGGCCTTTTTAGGCTATGTTCTCCCATGAGGACAAATATCTTTTTGGGGGGCAACGGTTATTACAAGTTTATTTTCAGCACTCCCCGTTATTGGGAGAGATGTGGTAATCTGATTGTGAGGAGGGTTTTCAGTAGACAACGCTACTCTTACCCGATTTTTTGGGCTTCATTTTGTGTTGCCTTTTATGGTAGCAGGGTTAGCAGGATTGCACCTGTTCTTCCTTCACGACCAAGGGTCGTCAAATCCATTGGGGGTAAGGTCTGATTACAACAAGGTTGTTTTTCATCATTACTATGTGCTTAAAGATTTAGTGGGGGTTTGTTTCTTTTTAGGGCCTTTGCTCGGTCTGGTGTATTTTAACCCCTGGCTTTTGGGGGACCCTGAAAATTTTATTGAAGGAAACCCACTGGTTACACCCCACCATATTCAGCCGGAATGATATTTTCTATTCGCCTACGCTATTTTGCGGGCTGTGCCTAATAAGCTTGGGGGGGTAGTCGCCTTGGCGGCTGCGGTGCTGGTATTATTTTATATACCCTTTAGTCCTAAAACATCGATCAAAGGGTTATCCTTTTACCCGGGGGGTAAGAGTTTGTTTTTTAGAATAATTGGGGTGTTTTTTCTGCTCACCTGGGCGGGCTCTAAGCCGGTTGAGGAGCCCTATGTTGTAATTAGGCAAGTTCTAAGGTTTTTATATTTTGCGTATTTTATTTTAATTGGGCCCGTAACACGGGTTCAAGATAAGTTGCTGTAA

RP2_f6 1 ATTTATAAGG?TTCCCGGTTTAGGCCGTTAGTATCAGTAGTCCGGCACACCGTTATTACTTTACCTACCCCTATTAATATTTCAACCTGGTGAAATTTTGGTTCACTCCTAGGGGTTTGTCTAGTAATCCAAATTTTTAGGGGGTTAATTTTAAGGCTTCATTTTACGGCTCATACAGATCTCAGATTTTATGTGGTCATTCAAAGGTTTGGAGATGTCTACTACAATTGGTGGTTTCGTGCTTTTCATGCCAATGGTGCTAGGTTCTTTTTTATTGGTTTGTACTTACACATTGCGCGAGGGCTATACTACGGATCTTATGTTTTTAAGGAAGTATGATCTGTTGGAGTGGTAATTTTGCTGGTAGTTATAGCTAGGGCCTTTTTAGGCTATGTTCTCCCATGAGGACAAATATCTTTTTGGGGGGCAACGGTTATTACAAGTTTATTTTCAGCAGTCCCCGTTATTGGGAGAGATGTGGTAATCTGATTGTGAGGAGGGTTTTCAGTAGACAACGCTACTCTTACCCGATTTTTTGGGCTTCATTTTGTGTTGCCTTTTATGGTAGCAGGGTTAGCAGGATTGCACCTGTTCTTCCTTCACGACCAAGGGTCGTCAAATCCATTGGGGGTAAGGTCTGATTACAACAAGGTTGTTTTTCATCATTACTATGTGCTTAAAGATTTAGTGGGGGTTTGTTTCTTTTTAGGGCCTTTGCTCGGTCTGGTGTATTTTAACCCCTGGCTTCTGGGGGACCCTGAAAATTTTATTGAAGGAAACCCACTGGTTACACCCCACCATATTCAGCCGGAATGATATTTTCTATTCGCCTACGCTATTTTGCGGGCTGTGCCTAATAAGCTTGGGGGGGTAGTCGCCTTGGCGGCTGCGGTGCTGGTATTATTTTATATACCCTTTAGTCCTAAAACATCGATCAAAGGGTTATCCTTTTACCCGGGGGGTAAGAGTTTGTTTTTTAGAATAATTGGGGTGTTTTTTCTGCTCACCTGGGCGGGCTCTAAGCCGGTTGAAGAGCCCTATGTTGTAATTAGGCAAGTTCTAAGGTTTTTATATTTTGCGTATTTTATTTTAATTGGGCCCGTAACACGGGTTCAAGATAAGTTGCTGTAA

}

SampleName="IP 14"

SampleSize= 10

SampleData= {

IP_f3 6 ATTTACAAGGTTTCCCGGTTTAGGCCGTTAGTATCAGTAGTCCGGCACACCGTTATTACTTTACCTACCCCTATTAATATTTCAACCTGGTGAAATTTTGGTTCACTCCTAGGGGTTTGTCTAGTAATCCAAATTTTTAGGGGGTTAATTCTAAGGCTTCATTTTACGGCTCATACAGATCTCAGATTTTATGTGGTCATTCAAAGGTTTGGAGATGTCTATTACAATTGGTGGTTTCGTGCTTTTCATGCCAATGGTGCTAGGTTCTTTTTTATTGGTTTGTACTTACACATTGCGCGAGGGCTCTACTACGGATCTTATGTTTTTAAGGAAGTATGATCTGTTGGAGTGGTAATTTTGCTGGTAGTTATAGCTAGGGCCTTTTTAGGCTATGTTCTCCCATGAGGACAAATATCTTTTTGGGGGGCAACGGTTATTACAAGTTTATTTTCAGCACTCCCCGTTATTGGGAGAGATGTGGTAATCTGATTGTGAGGAGGGTTTTCAGTAGACAACGCTACTCTTACCCGATTTTTTGGGCTTCATTTTGTGTTGCCTTTTATGGTAGCAGGGTTAGCAGGATTGCACCTGTTCTTCCTTCACGACCAAGGGTCGTCAAATCCATTGGGGGTAAGGTCTGATTACAACAAGGTTGTTTTTCATCATTACTATGTGCTTAAAGATTTAGTGGGGGTTTGTTTCTTTTTAGGGCCTTTGCTCGGTCTGGTGTATTTTAACCCCTGGCTTCTGGGGGACCCTGAAAATTTTATTGAAGGAAACCCACTGGTTACACCCCACCATATTCAGCCGGAATGATATTTTCTATTCGCTTACGCTATTTTGCGGGCTGTGCCTAATAAGCTTGGGGGGGTAGTCGCCTTGGCGGCTGCGGTGCTGGTATTATTTTATATACCCTTTAGTCCTAAAACATCGATCAAAGGGTTATCCTTTTACCCGGGGGGTAAGAGTTTGTTTTTTAGAATAATTGGGGTGTTTTTTCTGCTTACCTGGGCGGGCTCTAAGCCGGTTGAAGAGCCCTATGTTGTAATTAGGCAAGTTCTAAGGTTTTTATATTTTGCGTATTTTATTTTAATTGGGCCCGTAACACGGGTCCAAGATAAGTTGCTGTAA

IP_f4 1 ATTTACAAGGTTTCCCGGTTTAGGCCGTTAGTATCAGTAGTCCGGCACACCGTTATTACTTTACCTACCCCTATTAATATTTCAACCTGGTGAAATTTTGGTTCACTCCTAGGGGTTTGTCTAGTAATCCAAATTTTTAGGGGGTTAATTCTAAGGCTTCATTTTACGGCTCATACAGATCTCAGATTTTATGTGGTCATTCAAAGGTTTGGAGATGTCTATTACAATTGGTGGTTTCGTGCTTTTCATGCCAATGGTGCTAGGTTCTTTTTTATTGGTTTGTACTTACACATTGCGCGAGGGCTCTACTACGGATCTTATGTTTTTAAGGAAGTATGATCTGTTGGAGTGGTAATTTTGCTGGTAGTTATAGCTAGGGCCTTTTTAGGCTATGTTCTCCCATGAGGACAAATATCTTTTTGGGGGGCAACGGTTATTACAAGTTTATTTTCAGCACTCCCCGTTATTGGGAGAGATGTGGTAATCTGATTGTGAGGAGGGTTTTCAGTAGACAACGCTACTCTTACCCGATTTTTTGGGCTTCATTTTGTGTTGCCTTTTATGGTAGCAGGGTTAGCAGGATTGCACCTGTTCTTCCTTCACGACCAAGGGTCGTCAAATCCATTGGGGGTAAGGTCTGATTACAACAAGGTTGTTTTTCATCATTACTATGTGCTTAAAGATTTAGTGGGGGTTTGTTTCTTTTTAGGGCCTTTGCTCGGTCTGGTGTATTTTAACCCTTGGCTTCTGGGGGACCCCGAAAATTTTATTGAAGGAAACCCACTGGTTACACCCCACCATATTCAGCCGGAATGATATTTTCTATTCGCTTACGCTATTTTGCGGGCTGTGCCTAATAAGCTTGGGGGGGTAGTCGCCTTGGCGGCTGCGGTGCTGGTATTATTTTATATACCCTTTAGTCCTAAAACATCGATCAAAGGGTTATCCTTTTACCCGGGGGGTAAGAGTTTGTTTTTTAGAATAATTGGGGTGTTTTTTCTGCTTACCTGGGCGGGCTCTAAGCCGGTTGAAGAGCCCTATGTTGTAATTAGGCAAGTTCTAAGGTTTTTATATTTTGCGTATTTTATTTTAATTGGGCCCGTAACACGGGTCCAAGATAAGTTGCTGTAA

InsPt_m2 1 ATTTACAAGGTTTCCCGGTTTAGGCCGTTAGTATCAGTAGTCCGGCACACCGTTATTACTTTACCTACCCCTATTAATATTTCAACCTGGTGAAATTTTGGTTCACTCCTAGGGGTTTGTCTAGTAATCCAAATTTTTAGGGGGTTAATTCTAAGGCTTCATTTTACGGCTCATACAGATCTCAGATTTTATGTGGTCATTCAAAGGTTTGGAGATGTCTATTACAATTGGTGGTTTCGTGCTTTTCATGCCAATGGTGCTAGGTTCTTTTTTATTGGTTTGTACTTACACATTGCGCGAGGGCTCTACTACGGATCTTATGTTTTTAAGGAAGTATGATCTGTTGGAGTGGTAATTTTGCTGGTAGTTATAGCTAGGGCCTTTTTAGGCTATGTTCTCCCATGAGGACAAATATCTTTTTGGGGGGCAACGGTTATTACAAGTTTATTTTCAGCACTCCCCGTTATTGGGAGAGATGTGGTAATCTGATTGTGAGGAGGGTTTTCAGTAGACAACGCTACTCTTACCCGATTTTTTGGGCTTCATTTTGTGTTGCCTTTTATGGTAGCAGGGTTAGCAGGATTGCACCTGTTCTTCCTTCACGACCAAGGGTCGTCAAATCCATTGGGGGTAAGGTCTGATTACAACAAGGTTGTTTTTCATCATTACTATGTGCTTAAAGATTTAGTGGGGGTTTGTTTCTTTTTAGGGCCTTTGCTCGGTCTGGTGTATTTTAACCCCTGGCTTCTGGGGGACCCTGAAAATTTTATTGAAGGAAACCCACTGGTTACACCCCACCATATTCAGCCGGAATGATATTTTCTATTCTCTTACGCTATTTTGCGGGCTGTGCCTAATAAGCTTGGGGGGGTAGTCGCCTTGGCGGCTGCGGTGCTGGTATTATTTTATATACCCTTTAGTCCTAAAACATCGATCAAAGGGTTATCCTTTTACCCGGGGGGTAAGAGTTTGTTTTTTAGAATAATTGGGGTGTTTTTTCTGCTTACCTGGGCGGGCTCTAAGCCGGTTGAAGAGCCCTATGTTGTAATTAGGCAAGTTCTAAGGTTTTTATATTTTGCGTATTTTATTTTAATTGGGCCCGTAACACGGGTCCAAGATAAGTTGCTGTAA

IP_f5 1 ATTTACAAGGTTTCCCGGTTTAGGCCGTTAGTATCAGTAGTCCGGCACACCGTTATTACTTTACCTACCCCTATTAATATTTCAACCTGGTGAAATTTTGGTTCACTCCTAGGGGTTTGTCTAGTAATCCAAATTTTTAGGGGGTTAATTCTAAGGCTTCATTTTACGGCTCATACAGATCTCAGATTTTATGTGGTCATTCAAAGGTTTGGAGATGTCTATTACAATTGGTGGTTTCGTGCTTTTCATGCCAATGGTGCTAGGTTCTTTTTTATTGGTTTGTACTTACACATTGCGCGAGGGCTCTACTACGGATCTTATGTTTTTAAGGAAGTATGATCTGTTGGAGTGGTAATTTTGCTGGTAGTTATAGCTAGGGCCTTTTTAGGCTATGTTCTCCCATGAGGACAAATATCTTTTTGGGGGGCAACGGTTATTACAAGTTTATTTTCAGCACTCCCCGTTATTGGGAGAGATGTGGTAATCTGATTGTGAGGAGGGTTTTCAGTAGACAACGCTACTCTTACCCGATTTTTTGGGCTTCATTTTGTGTTGCCTTTTATGGTAGCAGGGTTAGCAGGATTGCACTTGTTCTTCCTTCACGACCAAGGGTCGTCAAATCCATTGGGGGTAAGGTCTGATTACAACAAGGTTGTTTTTCATCATTACTATGTGCTTAAAGATTTAGTGGGGGTTTGTTTCTTTTTAGGGCCTTTGCTCGGTCTGGTGTATTTTAACCCCTGGCTTCTGGGGGACCCTGAAAATTTTATTGAAGGAAACCCACTGGTTACACCCCACCATATTCAGCCGGAATGATATTTTCTATTCGCTTACGCTATTTTGCGGGCTGTGCCTAATAAGCTTGGGGGGGTAGTCGCCTTGGCGGCTGCGGTGCTGGTATTATTTTATATACCCTTTAGTCCTAAAACATCGATCAAAGGGTTATCCTTTTACCCGGGGGGTAAGAGTTTGTTTTTTAGAATAATTGGGGTGTTTTTTCTGCTTACCTGGGCGGGCTCTAAGCCGGTTGAAGAGCCCTATGTTGTAATTAGGCAAGTTCTAAGGTTTTTATATTTTGCGTATTTTATTTTAATTGGGCCCGTAACACGGGTCCAAGATAAGTTGCTGTAA

InsPt_m3 1 A??TAC?AGGTTTCCCGGTTTAGGCCGTTAGTATCAGTAGTCCGGCACACCGTTATTACTTTACCTACCCCTATTAATATTTCAACCTGGTGAAATTTTGGTTCACTCCTAGGGGTTTGTCTAGTAATTCAAATTTTTAGGGGGTTAATTCTAAGGCTTCATTTTACGGCTCATACAGATCTCAGATTTTATGTGGTCATTCAAAGGTTTGGAGATGTCTATTACAATTGGTGGTTTCGTGCTTTCCATGCCAATGGTGCTAGGTTCTTTTTTATTGGTTTGTACTTACACATTGCGCGAGGGCTATACTACGGATCTTATGTTTTTAAGGAAGTATGATCTGTTGGAGTGGTAATTTTGCTGGTAGTTATAGCTAGGGCCTTTTTAGGCTATGTTCTCCCATGAGGACAAATATCTTTTTGGGGGGCAACGGTTATTACAAGTTTATTTTCAGCACTCCCCGTTATTGGGAGAGATGTGGTAATCTGATTGTGAGGAGGGTTTTCAGTAGACAACGCTACTCTTACCCGATTTTTTGGGCTTCATTTTGTGTTGCCTTTTATGGTAGCAGGGTTAGCAGGATTGCACTTGTTCTTCCTTCACGACCAAGGGTCGTCAAATCCATTGGGGGTAAGGTCTGATTACAACAAGGTTGTTTTTCATCATTACTATGTGCTTAAAGATTTAGTGGGGGTTTGTTTCTTTTTAGGGCCTTTGCTCGGTCTGGTGTATTTTAACCCCTGGCTTCTGGGGGACCCTGAAAATTTTATTGAAGGAAACCCATTGGTTACACCCCACCATATTCAGCCGGAATGATATTTTCTATTCGCTTACGCTATTTTGCGGGCTGTGCCTAATAAGCTTGGGGGGGTAGTCGCCTTGGCGGCTGCGGTGCTGGTATTATTTTATATACCCTTTAGTCCTAAAACATCGATCAAAGGGTTATCCTTTTACCCGGGGGGTAAGAGTTTGTTTTTTAGAATAATTGGGGTGTTTTTTCTGCTTACCTGGGCGGGCTCTAAGCCGGTTGAAGAGCCCTATGTTGTAATTAGGCAAGTTCTAAGGTTTTTATATTTTGCGTATTTTATTTTAATTGGGCCCGTAACACGGGTCCAA?ATAAGTTGCTGTAA

}

SampleName="AB3 15"

SampleSize= 10

SampleData= {

AB3_f14 1 ATTTAC?AGGTTTCCCGGTTTAGGCCGTTAGTATCAGCAGTCCGGCACACCGTTATTACTTTACCTACCCCTATTAATATTTCAACCTGGTGAAATTTTGGTTCACTCCTAGGGGTTTGTCTAGTAATCCAAATTTTTAGGGGGTTAATTCTAAGGCTTCATTTTACGGCTCATACAGATCTCAGATTTTATGTGGTCATTCAAAGGTTTGGAGATGTCTATTACAATTGGTGGTTTCGTGCTTTCCATGCCAATGGTGCTAGGTTCTTTTTTATTGGTTTGTACTTACACATTGCGCGAGGGCTATACTACGGATCTTATGTTTTTAAGGAAGTATGATCTGTTGGAGTGGTAATTTTGCTGGTAGTTATAGCTAGGGCCTTTTTAGGCTATGTTCTCCCGTGAGGACAAATATCTTTTTGGGGGGCAACGGTTATTACAAGTTTATTTTCAGCACTCCCCGTTATTGGGAGAGATGTGGTAATCTGATTGTGAGGAGGGTTTTCAGTAGACAACGCTACTCTTACCCGATTTTTTGGGCTTCATTTTGTGTTGCCTTTTATGGTAGCAGGGTTAGCAGGATTGCACCTGTTCTTCCTTCACGACCAAGGGTCGTCAAATCCATTGGGGGTAAGGTCTGATTACAACAAGGTTGTTTTTCATCATTACTATGTGCTTAAAGATTTAGTGGGGGTTTGTTTCTTTTTAGGGCCTTTGCTCGGTCTGGTGTATTTTAACCCCTGGCTTCTGGGGGACCCTGAAAATTTTATTGAAGGAAACCCATTGGTTACACCCCACCATATTCAGCCGGAATGATATTTTCTATTCGCTTACGCTATTTTGCGGGCTGTGCCTAATAAGCTTGGGGGGGTAGTCGCCTTGGCGGCTGCGGTGCTGGTATTATTTTATATACCCTTTAGTCCTAAAACATCGATCAAAGGGTTATCCTTTTACCCGGGGGGTAAGAGTTTGTTTTTTAGAATAATTGGGGTGTTTTTTCTGCTTACCTGGGCGGGCTCTAAGCCGGTTGAAGAGCCCTATGTTGTAATTAGGCAAGTTCTAAGGTTTTTATATTTTGCGTATTTTATTTTAATTGGGCCCGTAACACGGGTTCAAGATAAGTTGCTGTAA

AB3_f12 3 ATTTACAAGGTTTCCCGGTTTAGGCCGTTAGTATCAGTAGTCCGGCACACCGTTATTACTTTACCTACCCCTATTAATATTTCAACCTGGTGAAATTTTGGTTCACTCCTAGGGGTTTGTCTAGTAATCCAAATTTTTAGGGGGTTAATTCTAAGGCTTCATTTTACGGCTCATACAGATCTCAGATTTTATGTGGTCATTCAAAGGTTTGGAGATGTCTATTACAATTGGTGGTTTCGTGCTTTCCATGCCAATGGTGCTAGGTTCTTTTTTATTGGTTTGTACTTACACATTGCGCGAGGGCTATACTACGGATCTTATGTTTTTAAGGAAGTATGATCTGTTGGAGTGGTAATTTTGCTGGTAGTTATAGCTAGGGCCTTTTTAGGCTATGTTCTCCCATGAGGACAAATATCTTTTTGGGGGGCAACGGTTATTACAAGTTTATTTTCAGCACTCCCCGTTATTGGGAGAGATGTGGTAATCTGATTGTGAGGAGGGTTTTCAGTAGACAACGCTACTCTTACCCGATTTTTTGGGCTTCATTTTGTGTTGCCTTTTATGGTAGCAGGGTTAGCAGGATTGCACCTGTTCTTCCTTCACGACCAAGGGTCGTCAAATCCATTGGGGGTAAGGTCTGATTACAACAAGGTTGTTTTTCATCATTACTATGTGCTTAAAGATTTAGTGGGGGTTTGTTTCTTTTTAGGGCCTTTGCTCGGTCTGGTGTATTTTAACCCCTGGCTTCTGGGGGACCCTGAAAATTTTATTGAAGGAAACCCATTGGTTACACCCCACCATATTCAGCCGGAATGATATTTTCTATTCGCTTACGCTATTTTGCGGGCTGTGCCTAATAAGCTTGGGGGGGTAGTCGCCTTGGCGGCTGCGGTGCTGGTATTATTTTATATACCCTTTAGTCCTAAAACATCGATCAAAGGGTTATCCTTTTACCCGGGGGGTAAGAGTTTGTTTTTTAGAATAATTGGGGTGTTTTTTCTGCTTACCTGGGCGGGCTCTAAGCCGGTTGAAGAGCCCTATGTTGTAATTAGGCAAGTTCTAAGGTTTTTATATTTTGCGTATTTTATTTTAATTGGGCCCGTAACACGGGTCCAAGATAAGTTGCTGTAA

AB3_f10 1 ATTTACAAGGTTTCCCGGTTTAGGCCGTTAGTATCAGTAGTCCGGCACACCGTTATTACTTTACCTACCCCTATTAATATTTCAACCTGGTGAAATTTTGGTTCACTCCTAGGGGTTTGTCTAGTAATCCAAATTTTTAGGGGGTTAATTCTAAGGCTTCATTTTACGGCTCATACAGATCTCAGATTTTATGTGGTCATTCAAAGGTTTGGAGATGTCTATTACAATTGGTGGTTTCGTGCTTTCCATGCCAATGGTGCTAGGTTCTTTTTTATTGGTTTGTACTTACACATTGCGCGAGGGCTATACTACGGATCTTATGTTTTTAAGGAAGTATGATCTGTTGGAGTGGTAATTTTGCTGGTAGTTATAGCTAGGGCCTTTTTAGGCTATGTTCTCCCATGAGGACAAATATCTTTTTGGGGGGCAACGGTTATTACAAGTTTATTTTCAGCACTCCCCGTTATTGGGAGAGATGTGGTAATCTGATTGTGAGGAGGGTTTTCAGTAGACAACGCTACTCTTACCCGATTTTTTGGGCTTCATTTTGTGTTGCCTTTTATGGTAGCAGGGTTAGCAGGATTGCACCTGTTCTTCCTTCACGACCAAGGGTCGTCAAATCCATTGGGGGTAAGGTCTGATTACAACAAGGTTGCTTTTCATCATTACTATGTGCTTAAAGATTTAGTGGGGGTTTGTTTCTTTTTAGGGCCTTTGCTCGGTCTGGTGTATTTTAACCCCTGGCTTCTGGGGGACCCTGAAAATTTTATTGAAGGAAACCCATTGGTTACACCCCACCATATTCAGCCGGAATGATATTTTCTATTCGCTTACGCTATTTTGCGGGCTGTGCCCAATAAGCTTGGGGGGGTAGTCGCCTTGGCGGCTGCGGTGCTGGTATTATTTTATATACCCTTTAGTCCTAAAACATCGATCAAAGGGTTATCCTTTTACCCGGGGGGTAAGAGTTTGTTTTTTAGAATAATTGGGGTGTTTTTTCTGCTTACCTGGGCGGGCTCTAAGCCGGTTGAAGAGCCCTATGTTGTAATTAGGCAAGTTCTAAGGTTTTTATATTTTGCGTATTTTATTTTAATTGGGCCCGTAACACGGGTCCAAGATAAGTTGCTGTAA

AB3_f1 1 ATTTACAAGGTTTCCCGGTTTAGGCCGTTAGTATCAGTAGTCCGGCACACCGTTATTACTTTACCTACCCCTATTAATATTTCAACCTGGTGAAATTTTGGTTCACTCCTAGGGGTTTGTCTAGTAATCCAAATTTTTAGGGGGTTAATTCTAAGGCTTCATTTTACGGCTCACACAGATCTCAGATTTTATGTGGTCATTCAAAGGTTTGGAGATGTCTATTACAATTGGTGGTTTCGTGCTTTCCATGCCAATGGTGCTAGGTTCTTTTTTATTGGTTTGTACTTACACATTGCGCGAGGGCTATACTACGGATCTTATGTTTTTAAGGAAGTATGATCTGTTGGAGTGGTAATTTTGCTGGTAGTTATAGCTAGGGCCTTTTTAGGCTATGTTCTCCCATGAGGACAAATATCTTTTTGGGGGGCAACGGTTATTACAAGTTTATTTTCAGCACTCCCCGTTATTGGGAGAGATGTGGTAATCTGATTGTGAGGAGGGTTTTCAGTAGACAACGCTACTCTTACCCGATTTTTTGGGCTTCATTTTGTGTTGCCTTTTATGGTAGCAGGGTTAGCAGGATTGCACCTGTTCTTCCTTCACGACCAAGGGTCGTCAAATCCATTGGGGGTAAGGTCTGATTACAACAAGGTTGTTTTTCATCATTACTATGTGCTTAAAGATTTAGTGGGGGTTTGTTTCTTTTTAGGGCCTTTGCTCGGTCTGGTGTATTTTAACCCCTGGCTTCTGGGGGACCCTGAAAATTTTATTGAAGGAAACCCATTGGTTACACCCCACCATATTCAGCCGGAATGATATTTTCTATTCGCTTACGCTATTTTGCGGGCTGTGCCTAATAAGCTTGGGGGGGTAGTCGCCTTGGCGGCTGCGGTGCTGGTATTATTTTATATACCCTTTAGTCCTAAAACATCGATCAAAGGGTTATCCTTTTACCCGGGGGGTAAGAGTTTGTTTTTTAGAATAATTGGGGTGTTTTTTCTGCTTACCTGGGCGGGCTCTAAGCCGGTTGAAGAGCCCTATGTTGTAATTAGGCAAGTTCTAAGGTTTTTATATTTTGCGTATTTTATTTTAATTGGGCCCGTAACACGGGTCCAAGATAAGTTGCTGTAA

AB3_f13 1 ATTTAC?AGGTTTCCCGGTTTAGGCCGTTAGTATCAGTAGTCCGGCACACCGTTATTACTTTACCTACCCCTATTAATATTTCAACCTGGTGAAATTTTGGTTCACTCCTAGGGGTTTGTCTAGTAATCCAAATTTTTAGGGGGTTAATTCTAAGGCTTCATTTTACGGCTCATACAGATCTCAGATTTTATGTGGTCATTCAAAGGTTTGGAGATGTCTATTACAATTGGTGGTTTCGTGCTTTCCATGCCAATGGTGCTAGGTTCTTTTTTATTGGTTTGTACTTACACATTGCGCGAGGGCTATACTACGGATCTTATGTTTTTAAGGAAGTATGATCTGTTGGAGTGGTAATCTTGCTGGTAGTTATAGCTAGGGCCTTTTTAGGCTATGTTCTCCCATGAGGACAAATATCTTTTTGGGGGGCAACGGTTATTACAAGTTTATTTTCAGCACTCCCCGTTATTGGGAGAGATGTGGTAATCTGATTGTGAGGAGGGTTCTCAGTAGACAACGCTACTCTTACCCGATTTTTTGGGCTTCATTTTGTGTTGCCTTTTATGGTAGCAGGGTTAGCAGGATTGCACCTGTTCTTCCTTCACGACCAAGGGTCGTCAAATCCATTGGGGGTAAGGTCTGATTACAACAAGGTTGTTTTTCATCATTACTATGTGCTTAAAGATTTAGTGGGGGTTTGTTTCTTTTTAGGGCCTTTGCTCGGTCTGGTGTATTTTAACCCCTGGCTTCTGGGGGACCCTGAAAATTTTATTGAAGGAAACCCATTGGTTACACCCCACCATATTCAGCCGGAATGATATTTTCTATTCGCTTACGCTATTTTGCGGGCTGTGCCTAATAAGCTTGGGGGGGTAGTCGCCTTGGCGGCTGCGGTGCTGGTATTATTTTATATACCCTTTAGTCCTAAAACATCTATCAAAGGGTTATCCTTTTACCCGGGGGGTAAGAGTTTGTTTTTTAGAATAATTGGGGTGTTTTTTCTGCTTACCTGGGCGGGCTCTAAGCCGGTTGAAGAGCCCTATGTTGTAATTAGGCAAGTTCTAAGGTTTTTATATTTTGCGTATTTTATTTTAATTGGGCCCGTAACACGGGTCCAAGATAAGTTGCTGTAA

AB3_m2 1 ??TTACAAGGTTTCCCGGTTTAGGCCGTTAGTATCAGTAGTCCGGCACACCGTCATTACTTTACCTACCCCTATTAATATTTCAACCTGGTGAAATTTTGGTTCACTCCTAGGGGTTTGTCTAGTAATCCAAATTTTTAGGGGGTTAATTCTAAGGCTTCATTTTACGGCTCATACAGATCTCAGATTTTATGTGGTCATTCAAAGGTTTGGAGATGTCTATTACAATTGGTGGTTTCGTGCTTTCCATGCCAATGGTGCTAGGTTCTTTTTTATTGGTTTGTACTTACACATTGCGCGAGGGCTATACTACGGATCTTATGTTTTTAAGGAAGTATGATCTGTTGGAGTGGTAATTTTGCTGGTAGTTATAGCTAGGGCCTTTTTAGGCTATGTTCTCCCATGAGGACAAATATCTTTTTGGGGGGCAACGGTTATTACAAGTTTATTTTCAGCACTCCCCGTTATTGGGAGAGATGTGGTAATCTGATTGTGAGGAGGGTTTTCAGTAGACAACGCTACTCTTACCCGATTTTTTGGGCTTCATTTTGTGTTGCCTTTTATGGTAGCAGGGTTAGCAGGATTGCACCTGTTCTTCCTTCACGACCAAGGGTCGTCAAATCCATTGGGGGTAAGGTCTGATTACAACAAGGTTGTTTTTCATCATTACTATGTGCTTAAAGATTTAGTGGGGGTTTGTTTCTTTTTAGGGCCTTTGCTCGGTCTGGTGTATTTTAACCCCTGGCTTCTGGGGGACCCTGAAAATTTTATTGAAGGAAACCCATTGGTTACACCCCACCATATTCAGCCGGAATGATATTTTCTATTCGCTTACGCTATTTTGCGGGCTGTGCCTAATAAGCTTGGGGGGGTAGTCGCCTTGGCGGCTGCGGTGTTGGTATTATTTTATATACCCTTTAGTCCTAAAACATCGATCAAAGGGTTATCCTTTTACCCGGGGGGTAAGAGTTTGTTTTTTAGAATAATTGGGGTGTTTTTTCTGCTTACCTGGGCGGGCTCTAAGCCGGTTGAAGAGCCCTATGTTGTAATTAGGCAAGTTCTAAGGTTTTTATATTTTGCGTATTTTATTTTAATTGGGCCCGTAACACGGGTCCAAGATAAGTTGCTGTAA

AB3_f11 1 ??TTAC?AGGTTTCCCGGTTTAGGCCGTTAGTATCAGTAGTCCGGCACACCGTTATTACTTTACCTACCCCTATTAATATTTCAACCTGGTGAAATTTTGGTTCACTCCTAGGGGTTTGTCTAGTAATCCAAATTTTTAGGGGGTTAATTCTAAGGCTTCATTTTACGGCTCATACAGATCTCAGATTTTATGTGGTCATTCAAAGGTTTGGAGATGTCTATTACAATTGGTGGTTTCGTGCTTTCCATGCCAATGGTGCTAGGTTCTTTTTTATTGGTTTGTACTTACACATTGCGCGAGGGCTATACTACGGATCTTATGTTTTTAAGGAAGTATGATCTGTTGGAGTGGTAATTTTGCTGGTAGTTATAGCTAGGGCCTTTTTAGGCTATGTTCTCCCATGAGGACAAATATCTTTTTGGGGGGCAACGGTTATTACAAGTTTATTTTCAGCACTCCCCGTTATTGGGAGAGATGTGGTAATCTGATTGTGAGGAGGGTTTTCAGTAGACAACGCTACTCTTACCCGATTTTTTGGGCTTCATTTTGTGTTGCCTTTTGTGGTAGCAGGGTTAGCAGGATTGCACCTGTTCTTCCTTCACGACCAAGGGTCGTCAAATCCATTGGGGGTAAGGTCTGATTACAACAAGGTTGTTTTTCATCATTACTATGTGCTTAAAGATTTAGTGGGGGTTTGTTTCTTTTTAGGGCCTTTGCTCGGTCTGGTGTATTTTAACCCCTGGCTTCTGGGGGACCCTGAAAACTTTATTGAAGGAAACCCATTGGTTACACCCCACCATATTCAGCCGGAATGATATTTTCTATTCGCTTATGCTATTTTGCGGGCTGTGCCTAATAAGCTTGGGGGGGTAGTCGCCTTGGCGGCTGCGGTGCTGGTATTATTTTATATACCCTTTAGTCCTAAAACATCGATCAAAGGGTTATCCTTTTACCCGGGGGGTAAGAGTTTGTTTTTTAGAATAATTGGGGTGTTTTTTCTGCTTACCTGGGCGGGCTCTAAGCCGGTTGAAGAGCCCTATGTTGTAATTAGGCAAGTTCTAAGGTTTTTATATTTTGCGTATTTTATTTTAATTGGGCCCGTAACACGGGTCCAAGATAAGTTGCTGTAA

AB3_f16 1 ATTTAC?AGGTTTCCCGGTTTAGGCCGTTAGTATCAGTAGTCCGGCACACCGTTATTACTTTACCTACCCCTATTAATATTTCAACCTGGTGAAATTTTGGTTCACTCCTAGGGGTTTGTCTAGTAATCCAAATTTTTAGGGGGTTAATTCTAAGGCTTCATTTTACGGCTCATACAGATCTCAGATTTTATGTGGTCATTCAAAGGTTTGGAGATGTCTATTACAATTGGTGGTTTCGTGCTTTCCATGCCAATGGTGCTAGGTTCTTTTTTATTGGTTTGTACTTACACATTGCGCGAGGGCTATACTACGGATCTTATGTTTTTAAGGAAGTATGATCTGTTGGAGTGGTAATTTTGCTGGTAGTTATAGCTAGGGCCTTTTTAGGCTATGTTCTTCCATGAGGACAAATATCTTTTTGGGGGGCAACGGTTATTACAAGTTTATTTTCAGCACTCCCCGTTATTGGGAGAGATGTGGTAATCTGATTGTGAGGAGGGTTTTCAGTAGACAACGCTACTCTTACCCGATTTTTTGGGCTTCATTTTGTGTTGCCTTTTATGGTAGCAGGGTTAGCAGGATTGCACCTGTTCTTCCTTCACGACCAAGGGTCGTCAAATCCATTGGGGGTAAGGTCTGATTACAACAAGGTTGTTTTTCATCATTACTATGTGCTTAAAGATTTAGTGGGGGTTTGTTTCTTTTTAGGGCCTTTGCTCGGTCTGGTGTATTTTAACCCCTGGCTTCTGGGGGACCCTGAAAATTTTATTGAAGGAAACCCATTGGTTACACCCCACCATATTCAGCCGGAATGATATTTTCTATTCGCTTACGCTATTTTGCGGGCTGTGCCTAATAAGCTTGGGGGGGTAGTCGCCTTGGCGGCTGCGGTGCTGGTATTATTTTATATACCCTTTAGTCCTAAAACATCGATCAAAGGGTTATCCTTTTACCCGGGGGGTAAGAGTTTGTTTTTTAGAATAATTGGGGTGTTTTTTCTGCTTACCTGGGCGGGCTCTAAGCCGGTTGAAGAGCCCTATGTTGTAATTAGGCAAGTTCTAAGGTTTTTATATTTTGCGTATTTTATTTTAATTGGGCCCGTAACACGGGTCCAAGATAAGTTGCTGTAA

}

SampleName="AB2 16"

SampleSize= 11

SampleData= {

AB3_f12 3

AB_2m 1 ATTTACAAGGTTTCCCGGTTTAGGCCGTTAGTATCAGTAGTCCGGCACACCGTTATTACTTTACCTACCCCTATTAATATTTCAACCTGGTGAAATTTTGGTTCACTCCTAGGGGTTTGTCTAGTAATCCAAATTTTTAGGGGGTTAATTCTAAGGCTTCATTTTACGGCTCATACAGATCTCAGATTTTATGTGGTCATTCAAAGGTTTGGAGATGTCTATTACAATTGGTGGTTTCGTGCTTTCCATGCCAATGGTGCTAGGTTCTTTTTTATTGGTTTGTACTTACACATTGCGCGAGGGCTATACTACGGATCTTATGTTTTTAAGGAAGTATGATCTGTTGGAGTGGTAATTTTGCTGGTAGTTATAGCTAGGGCCTTTTTAGGCTATGTTCTCCCATGAGGACAAATATCTTT?TGGGGGGCAACGGTTATTACAAGTTTATTCTCAGCACTCCCCGTTATTGGGAGAGATGTGGTAATCTGATTGTGAGGAGGGTTTTCAGTAGACAACGCTACTCTTACCCGATTTTTTGGGCTTCATTTTGTGTTGCCTTTTATGGTAGCAGGGTTGGCAGGATTGCACCTGTTCTTCCTTCACGACCAAGGGTCGTCAAATCCATTGGGGGTAAGGTCTGATTACAACAAGGTTGTTTTTCATCATTACTATGTGCTTAAAGATTTAGTGGGGGTTTGTTTCTTTTTAGGGCCTTTGCTCGGTCTGGTGTATTTTAACCCCTGGCTTCTGGGGGACCCTGAAAATTTTATTGAAGGAAACCCATTGGTTACACCCCACCATATTCAGCCGGAATGATATTTTCTATTCGCTTACGCTATTTTGCGGGCTGTGCCTAATAAGCTTGGGGGGGTAGTCGCCTTGGCGGCTGCGGTGCTGGTATTATTTTATATACCCTTTAGTCCTAAAACATCGATCAAAGGGTTATCCTTTTACCCGGGGGGTAAGAGTTTGTTTTTTAGAATAATTGGGGTGTTTTTTCTGCTTACCTGGGCGGGCTCTAAGCCGGTTGAAGAGCCCTATGTTGTAATTAGGCAAGTTCTAAGGTTTTTATATTTTGCGTATTTTATTTTAATTGGGCCCGTAACACGGGTCCAAGATAAGTTGCTGTAA

AB2_f1 1 ATTTACAAGGTTTCCCGGTTTAGGCCGTTAGTATCAGCAGTCCGGCACACCGTTATTACTTTACCTACCCCTATTAATATTTCAACCTGGTGAAATTTTGGTTCACTCCTAGGGGTTTGTCTAGTAATCCAAATTTTTAGGGGGTTAATTCTAAGGCTTCATTTTACGGCTCATACAGATCTCAGATTTTATGTGGTCATTCAAAGGTTTGGAGATGTCTATTACAATTGGTGGTTTCGTGCTTTCCATGCCAATGGTGCTAGGTTCTTTTTTATTGGTTTGTACTTACACATTGCGCGAGGGCTATATTACGGATCTTATGTTTTTAAGGAAGTATGATCTGTTGGAGTGGTAATTTTGCTGGTAGTTATAGCTAGGGCCTTTTTAGGCTATGTTCTCCCATGAGGACAAATATCTTTTTGGGGGGCAACGGTTATTACAAGTTTATTTTCAGCACTCCCCGTTATTGGGAGAGATGTGGTAATCTGATTGTGAGGAGGGTTTTCAGTAGACAACGCTACTCTTACCCGATTTTTTGGGCTTCATTTTGTGTTGCCTTTTATGGTAGCAGGGTTAGCAGGATTGCACCTGTTCTTCCTTCACGACCAAGGGTCGTCAAATCCATTGGGGGTAAGGTCTGATTACAACAAGGTTGTTTTTCATCATTACTATGTGCTTAAAGATTTAGTGGGGGTTTGTTTCTTTTTAGGGCCTTTGCTCGGTCTGGTGTATTTTAACCCCTGGCTTCTGGGGGACCCTGAAAATTTTATTGAAGGAAACCCATTGGTTACACCCCACCATATTCAGCCGGAATGATATTTTCTATTCGCTTACGCTATTTTGCGGGCTGTGCCTAATAAGCTTGGGGGGGTAGTCGCCTTGGCGGCTGCGGTGCTGGTATTATTTTATATACCCTTTAGTCCTAAAACATCGATCAAAGGGTTATCCTTTTACCCGGGGGGTAAGAGTTTGTTTTTTAGAATAATTGGGGTGTTTTTTCTGCTTACCTGGGCGGGCTCTAAGCCGGTTGAAGAGCCCTATGTTGTAATTAGGCAAGTTCTAAGGTTTTTATATTTTGCGTATTTTATTTTAATTGGGCCCGTAACACGGGTCCAAGATAAGTTGCTGTAA

AB2_m16 1 ATTTACAAGGTTTCCCGGTTTAGGCCGTTAGTATCAGCAGTCCGGCACACCGTTATTACTTTACCTACCCCTATTAATATTTCAACCTGGTGAAATTTTGGTTCACTCCTAGGGGTTTGTCTAGTAATCCAAATTTTTAGGGGGTTAATTCTAAGGCTTCATTTTACGGCTCATACAGATCTCAGATTTTATGTGGTCATTCAAAGGTTTGGAGATGTCTATTACAATTGGTGGTTTCGTGCTTTCCATGCCAATGGTGCTAGGTTCTTTTTTATTGGTTTGTACTTACACATTGCGCGAGGGCTATACTACGGATCTTATGTTTTTAAGGAAGTATGATCTGTTGGAGTGGTCATTTTGCTGGTAGTTATAGCTAGCGCCTTTTTAGGCTATGTTCTCCCATGAGGACAAATATCTTTTTGGGGGGCAACGGTTATTACAAGTTTATTTTCAGCACTCCCCGTTATTGGGAGAGATGTGGTAATCTGATTGTGAGGAGGGTTTTCAGTAGACAACGCTACTCTTACCCGATTTTTTGGGCTTCATTTTGTGTTGCCTTTTATGGTAGCAGGGTTAGCAGGATTGCACCTGTTCTTCCTTCACGACCAAGGGTCGTCAAATCCATTGGGGGTAAGGTCTGATTACAACAAGGTTGTTTTTCATCATTACTATGTGCTTAAAGATTTAGTGGGGGTTTGTTTCTTTTTAGGGCCTTTGCTCGGTCTGGTGTATTTTAACCCCTGGCTTCTGGGGGACCCTGAAAATTTTATTGAAGGAAACCCATTGGTTACACCCCACCATATTCAGCCGGAATGATATTTTCTATTCGCTTACGCTATTTTGCGGGCTGTGCCTAATAAGCTTGGGGGGGTAGTCGCCTTGGCGGCTGCGGTGCTGGTATTATTTTATATACCCTTTAGTCCTAAAACATCGATCAAAGGGTTATCCTTTTACCCGGGGGGTAAGAGTTTGTTTTTTAGAATAATTGGGGTGTTTTTTCTGCTTACCTGGGCGGGCTCTAAGCCGGTTGAAGAGCCCTATGTTGTAATTAGGCAAGTTCTAAGGTTTTTATATTTTGCGTATTTTATTTTAATTGGGCCCGTAACACGGGTTCAAGATAAGTTGCTGTAA

AB2_2m 2 ATTTACAAGGTTTCCCGGTTTAGGCCGTTAGTATCAGTAGTCCGGCACACCGTTATTACTTTACCTACCCCTATTAATATTTCAACCTGGTGAAATTTTGGTTCACTCCTAGGGGTTTGTCTAGTAATCCAAATTTTTAGGGGGTTAATTCTAAGGCTTCATTTTACGGCTCATACAGATCTCAGATTTTATGTGGTCATTCAAAGGTTTGGAGATGTCTATTACAATTGGTGGTTTCGTGCTTTCCATGCCAATGGTGCTAGGTTCTTTTTTATTGGTTTGTACTTACACATTGCGCGAGGGCTATACTACGGATCTTATGTTTTTAAGGAAGTATGATCTGTTGGAGTGGTAATTTTGCTGGTAGTTATAGCTAGGGCCTTTTTAGGCTATGTTCTCCCATGAGGACAAATATCTTTTTGGGGGGCAACGGTTATTACAAGTTTATTTTCAGCACTCCCCGTTATTGGGAGAGATGTGGTAATCTGATTATGAGGAGGGTTTTCAGTAGACAACGCTACTCTTACCCGATTTTTTGGGCTTCATTTTGTGTTGCCTTTTATGGTAGCAGGGTTAGCAGGATTGCACCTGTTCTTCCTTCACGACCAAGGGTCGTCAAATCCATTGGGGGTAAGGTCTGATTACAACAAGGTTGTTTTTCATCATTACTATGTGCTTAAAGATTTAGTGGGGGTTTGTTTCTTTTTAGGGCCTTTGCTCGGTCTGGTGTATTTTAACCCCTGGCTTCTGGGGGACCCTGAAAATTTTATTGAAGGAAACCCATTGGTTACACCCCACCATATTCAGCCGGAATGATATTTTCTATTCGCTTACGCTATTTTGCGGGCTGTGCCTAATAAGCTTGGGGGGGTAGTCGCCTTGGCGGCTGCGGTGCTGGTATTATTTTATATACCCTTTAGTCCTAAAACATCGATCAAAGGGTTATCCTTTTACCCGGGGGGTAAGAGTTTGT??TTTAGAATAATTGGGGTGTT?TTTCTGCTTACCTGGGCGGGCTCTAAGCCGGTTGAAGAGCCCTATGTTGTAATTAGGCAAGTTCTAAGGTTTATATATTTTGCGTATTTTATTTTAATTGGGCCCGTAACACGGGTCCAAGATAAGTTGCTGTAA

AB2_f3 2 ATTTACAAGGTTTCCCGGTTTAGGCCGTTAGTATCAGTAGTCCGGCACACCGTTATTACTTTACCTACCCCTATTAATATTTCAACCTGGTGAAATTTTGGTTCACTCCTAGGGGTTTGTCTAGTAATCCAAATTTTTAGGGGGTTAATTCTAAGGCTTCATTTTACGGCTCACACAGATCTCAGATTTTATGTGGTCATTCAAAGGTTTGGAGATGTCTATTACAATTGGTGGTTTCGTGCTTTCCATGCCAATGGTGCTAGGTTCTTTTTTATTGGTTTGTACTTACACATTGCGCGAGGGCTATACTACGGATCTTATGTTTTTAAGGAAGTATGATCTGTTGGAGTGGTAATTTTGCTGGTAGTCATAGCTAGGGCCTTTTTAGGCTATGTTCTCCCATGAGGACAAATATCTTTTTGGGGGGCAACGGTTATTACAAGTTTATTTTCAGCACTCCCCGTTATTGGGAGAGATGTGGTAATCTGATTGTGAGGAGGGTTTTCAGTAGACAACGCTACTCTTACCCGATTTTTTGGGCTTCATTTTGTGTTGCCTTTTATGGTAGCAGGGTTAGCAGGATTGCACCTGTTCTTCCTTCACGACCAAGGGTCGTCAAATCCATTGGGGGTAAGGTCTGATTACAACAAGGTTGTTTTTCATCATTACTATGTGCTTAAAGATTTAGTGGGGGTTTGTTTCTTTTTAGGGCCTTTGCTCGGTCTGGTGTATTTTAACCCCTGGCTTCTGGGGGACCCTGAAAATTTTATTGAAGGAAACCCATTGGTTACACCCCACCATATTCAGCCGGAATGATATTTTCTATTCGCTTACGCTATTTTGCGGGCTGTGCCTAATAAGCTTGGGGGGGTAGTCGCCTTGGCGGCTGCGGTGCTGGTATTATTTTATATACCCTTTAGTCCTAAAACATCGATCAAAGGGTTATCCTTTTACCCGGGGGGTAAGAGTTTGTTTTTTAGAATAATTGGGGTGTTTTTTCTGCTTACCTGGGCGGGCTCTAAGCCGGTTGAAGAGCCCTATGTTGTAATTAGGCAAGTTCTAAGGTTTTTATATTTTGCGTATTTTATTTTAATTGGGCCCGTAACACGGGTCCAAGATAAGTTGCTGTAA

AB_1f 1 ATTTACAAGGTTTCCCGGTTTAGGCCGTTAGTATCAGCAGTCCGGCACACCGTTATTACTTTACCTACCCCTATTAATATTTCAACCTGGTGAAATTTTGGTTCACTCCTAGGGGTTTGTCTAGTAATCCAAATTTTTAGGGGGTTAATTCTAAGGCTTCATTTTACGGCTCATACAGATCTCAGATTTTATGTGGTCATTCAAAGGTTTGGAGATGTCTATTACAATTGGTGGTTTCGTGCTTTCCATGCCAATGGTGCTAGGTTCTTTTTTATTGGTTTGTACTTACACATTGCGCGAGGGCTATACTACGGATCTTATGTTTTTAAGGAAGTATGATCTGTTGGAGTGGTAATTTTGCTGGTAGTTATAGCTAGGGCCTTTTTAGGCTATGTTCTCCCATGAGGACAAATATCTTTTTGGGGGGCAACGGTTATTACAAGTTTATTTTCAGCACTCCCCGTTATTGGGAGAGATGTGGTAATCTGATTGTGAGGAGGGTTTTCAGTAGACAACGCTACTCTTACCCGATTTTTTGGGCTTCATTTTGTGTTGCCTTTTATGGTAGCAGGGTTAGCAGGATTGCACCTGTTCTTCCTTCACGACCAAGGGTCGTCAAATCCATTGGGGGTAAGGTCTGATTACAACAAGGTTGTTTTTCATCATTACTATGTGCTTAAAGATTTAGTGGGGGTTTGTTTCTTTTTAGGGCCTTTGCTCGGTCTGGTGTATTTTAACCCCTGGCTTCTGGGGGACCCTGAAAATTTTATTGAAGGAAACCCATTGGTTACACCCCACCATATTCAGCCGGAATGATATTTTCTATTCGCTTACGCTATTTTGCGGGCTGTGCCTAATAAGCTTGGGGGGGTAGTCGCCTTGGCGGCTGCGGTGCTGGTATTATTTTATATACCCTTTAGTCCTAAAACGTCGATCAAAGGGTTATCCTTTTACCCGGGGGGTAAGAGTTTGTTTTTTAGAATAATTGGGGTGTTTTTTCTGCTTACCTGGGCGGGCTCTAAGCCGGTTGAAGAGCCCTATGTTGTAATTAGGCAAGTTCTAAGGTTTTTATATTTTGCGTATTTTATTTTAATTGGGCCCGTAACACGGGTTCAAGATAAGTTGCTGTAA

}

SampleName="AB 17"

SampleSize= 11

SampleData= {

AB3_f12 2

AB2_f3 4

AB_1f 2

AB_2m 2

AB2_m16 1

}

SampleName="ABR 18"

SampleSize= 10

SampleData= {

ABR_m3 10 ATTTACAAGGTTTCCCGGTTTAGGCCGTTAGTATCAGTAGTCCGGCACACCGTTATTACTTTACCTACCCCTATTAATATTTCAACCTGGTGAAATTTTGGTTCACTCCTAGGGGTTTGTCTAGTAATCCAAATTTTTAGGGGGTTAATTCTAAGGCTTCATTTTACGGCTCATACAGATCTCAGATTTTATGTGGTCATTCAAAGGTTTGGAGATGTCTATTACAATTGGTGGTTTCGTGCTTTCCATGCCAATGGTGCTAGGTTCTTTTTTATTGGTTTGTACTTACACATTGCGCGAGGGCTATACTACGGATCTTATGTTTTTAAGGAAGTATGATCTGTTGGAGTGGTAATTTTGCTGGTAGTTATAGCTAGGGCCTTTTTAGGCTATGTTCTCCCATGAGGACAAATATCTTTTTGGGGGGCAACGGTTATTACAAGTTTATTTTCAGCACTCCCCGTTATTGGGAGAGATGTGGTAATCTGATTGTGAGGAGGGTTTTCAGTAGACAACGCTACTCTTACCCGATTTTTTGGGCTTCATTTTGTGTTGCCTTTTATGGTAGCAGGGTTAGCAGGATTGCACCTGTTCTTCCTTCACGACCAAGGGTCGTCAAATCCATTGGGGGTAAGGTCTGATTACAATAAGGTTGTTTTTCATCATTACTATGTGCTTAAAGATTTAGTGGGGGTTTGTTTCTTTTTAGGGCCTTTGCTCGGTCTGGTGTATTTTAACCCCTGGCTTCTGGGGGACCCTGAAAATTTTATTGAAGGAAACCCATTGGTTACACCCCACCATATTCAGCCGGAATGATATTTTCTATTCGCTTACGCTATTTTGCGGGCTGTGCCTAATAAGCTTGGGGGGGTAGTCGCCTTGGCGGCTGCGGTGCTGGTATTATTTTATATACCCTTTAGTCCTAAAACATCGATCAAAGGGTTATCCTTTTACCCGGGGGGTAAGAGTTTGTTTTTTAGAATAATTGGGGTGTTTTTTCTGCTTACCTGGGCGGGCTCTAAGCCGGTTGAAGAGCCCTATGTTGTAATTAGGCAAGTTCTAAGGTTTTTATATTTTGCGTATTTTATTTTAATTGGGCCCGTAACACGGGTCCAAGATAAGTTGCTGTAA

}

SampleName="PVL 19"

SampleSize= 10

SampleData= {

PVL_m11 6 ATTTATAAGGCTTCCCGGTTTAGGCCGTTAGTATCAGTAGTCCGGCACACCGTTATTACTTTACCTACCCCTATTAATATTTCAACCTGGTGAAATTTTGGTTCACTTCTAGGGGTTTGTCTAGTAATCCAAATTTTTAGGGGGTTAATTCTAAGCCTTCATTTTACGGCTCATACAGATCTCAGATTTTATGTGGTCATTCAAAGGTTTGGAGATGTCTATTACAATTGGTGGTTTCGTGCTTTTCATGCCAATGGTGCTAGGTTCTTTTTTATTGGTTTGTACTTACACATTGCGCGAGGGCTATATTACGGATCTTATGTTTTTAAGGAAGTATGATCTGTTGGAGTGGTAATTTTGCTGGTAGTTATAGCTAGGGCCTTTTTAGGCTATGTTCTCCCATGAGGACAAATATCTTTTTGGGGGGCAACGGTTATTACAAGTTTATTTTCAGCACTCCCCGTTATTGGGAGAGATGTGGTAATCTGATTGTGAGGAGGGTTTTCAGTAGACAACGCTACTCTTACACGATTTTTTGGGCTTCATTTTGTGTTGCCTTTTATGGTAGCAGGGTTAGCAGGATTACACCTGTTCTTCCTTCACGACCAAGGGTCGTCAAATCCATTGGGGGTAAGGTCTGATTACAACAAGGTTGTTTTTCATCATTACTATGTGCTTAAAGATTTAGTGGGGGTTTGTTTCTTTTTAGGGCCTTTGGTCGGTCTGGTGTATTTTAACCCCTGGCTTCTGGGGGACCCTGAAAATTTTATTGAAGGAAACCCACTGGTTACACCCCACCATATTCAGCCGGAATGATATTTTCTATTCGCTTACGCTATTTTGCGGGCTGTGCCTAATAAGCTTGGGGGGGTAGTCGCCTTGGCGGCTGCGGTGCTGGTATTATTTTACATACCCTTTAGTCCTAAAACATCGATCAAAGGGTTATCCTTTTACCCGGGGGGTAAGAGTTTGTTTTTTAGAATAATTGGGGTGTTTTTTCTGCTCACCTGGGCGGGTTCTAAGCCGGTTGAAGAGCCCTATGTTGTAATTAGGCAAGTTCTAAGGTTTTTATATTTTGCGTATTTTATTTTAATTGGGCCCGTAACACGGATTCAAGATAAGTTGCTGTAA

PVL_f12 1 ATTTATAGGGCTTCCCGGTTTAGGCCGTTAGTCTCAGTAGTCCGGCACACCGTTATTACTTTACCTACCCCTATTAATATTTCAACCTGGTGAAATTTTGGTTCACTTCTAGGGGTTTGTCTAGTAATCCAAATTTTTAGGGGGTTAATTCTAAGCCTTCATTTTACGGCTCATACAGATCTCAGATTTTATGTGGTCATTCAAAGGTTTGGAGATGTCTATTACAATTGGTGGTTTCGTGCTTTTCATGCCAATGGTGCTAGGTTCTTTTTTATTGGTTTGTACTTACACATTGCGCGAGGGCTATATTACGGATCTTATGTTTTTAAGGAAGTATGATCTGTTGGAGTGGTAATTTTGCTGGTAGTTATAGCTAGGGCCTTTTTAGGCTATGTTCTCCCATGAGGACAAATATCTTTTTGGGGGGCAACGGTTATTACAAGTTTATTTTCAGCACTCCCCGTTATTGGGAGAGATGTGGTAATCTGATTGTGAGGAGGGTTTTCAGTAGACAACGCTACTCTTACACGATTTTTTGGGCTTCATTTTGTGTTGCCTTTTATGGTAGCAGGGTTAGCAGGATTACACCTGTTCTTCCTTCACGACCAAGGGTCGTCAAATCCATTGGGGGTAAGGTCTGATTACAACAAGGTTGTTTTTCATCATTACTATGTGCTTAAAGATTTAGTGGGGGTTTGTTTCTTTTTAGGGCCTTTGCTCGGTCTGGTGTATTTTAACCCCTGGCTTCTGGGGGACCCTGAAAATTTTATTGAAGGAAACCCACTGGTTACACCCCACCATATTCAGCCGGAATGATATTTTCTATTCGCTTACGCTATTTTGCGGGCTGTGCCTAATAAGCTTGGGGGGGTAGTCGCCTTGGCGGCTGCGGTGCTGGTATTATTTTACATACCCTTTAGTCCTAAAACATCGATCAAAGGGTTATCCTTTTACCCGGGGGGTAAGAGTTTGTTTTTTAGAATAATTGGGGTGTTTTTTCTGCTCACCTGGGCGGGTTCTAAGCCGGTTGAAGAGCCCTATGTTGTAATTAGGCAAGTTCTAAGGTTTTTATATTTTGCGTATTTTATTTTAATTGGGCCCGTAACACGGATTCAAGATAAGTTGCTGTAA

PVL_f3 3 A?TTATAAGGCTTCCCGGTTTAGGCCGTTAGTATCAGTAGTCCGGCACACCGTTATTACTTTACCTACCCCTATTAATATTTCAACCTGGTGAAATTTTGGTTCACTTCTAGGGGTTTGTCTAGTAATCCAAATTTTTAGGGGGTTAATTCTAAGCCTTCATTTTACGGCTCATACAGATCTCAGATTTTATGTGGTCATTCAAAGGTTTGGAGATGTCTATTACAATTGGTGGTTTCGTGCTTTTCATGCCAATGGTGCTAGGTTCTTTTTTATTGGTTTGTACTTACACATTGCGCGAGGGCTATATTACGGATCTTATGTTTTTAAGGAAGTATGATCTGTTGGAGTGGTAATTTTGCTGGTAGTTATAGCTAGGGCCTTTTTAGGCTATGTTCTCCCATGAGGACAAATATCTTTTTGGGGGGCAACGGTTATTACAAGTTTATTTTCAGCACTCCCCGTTATTGGGAGAGATGTGGTAATCTGATTGTGAGGAGGGTTTTCAGTAGACAACGCTACTCTTACACGATTTTTTGGGCTTCATTTTGTGTTGCCTTTTATGGTAGCAGGGTTAGCAGGATTACACCTGTTCTTCCTTCACGACCAAGGGTCGTCAAATCCATTGGGGGTAAGGTCTGATTACAACAAGGTTGTTTTTCATCATTACTATGTGCTTAAAGATTTAGTGGGGGTTTGTTTCTTTTTAGGGCCTTTGCTCGGTCTGGTGTATTTTAACCCCTGGCTTCTGGGGGACCCTGAAAATTTTATTGAAGGAAACCCACTGGTTACACCCCACCATATTCAGCCGGAATGATATTTTCTATTCGCTTACGCTATTTTGCGGGCTGTGCCTAATAAGCTTGGGGGGGTAGTCGCCTTGGCGGCTGCGGTGCTGGTATTATTTTACATACCCTTTAGTCCTAAAACATCGATCAAAGGGTTATCCTTTTACCCGGGGGGTAAGAGTTTGTTTTTTAGAATAATTGGGGTGTTTTTTCTGCTCACCTGGGCGGGTTCTAAGCCGGTTGAAGAGCCCTATGTTGTAATTAGGCAAGTTCTAAGGTTTTTATATTTTGCGTATTTTATTTTAATTGGGCCCGTAACACGGATTCAAGATAAGTTGCTGTAA

}

SampleName="RsPt 20"

SampleSize= 10

SampleData= {

RsPt_f1 1 ATTTATAAGGCTTCCCGGTTTAGGCCGTTAGTATCAGTAGTCCGGCACACCGTTATTACTTTACCTACCCCTATTAATATTTCAACCTGGTGAAATTTTGGTTCACTTCTAGGGGTTTGTCTAGTAATCCAAATTTTTAGGGGGTTAATTCTAAGCCTTCATTTTACGGCTCATACAGATCTCAGATTTTATGTGGTCATTCAAAGGTTTGGAGATGTCTATTACAATTGGTGGTTTCGTGCTTTTCATGCCAATGGTGCTAGGTTCTTTTTTATTGGTTTGTACTTACACATTGCGCGAGGGCTATATTACGGATCTTATGTTTTTAAGGAAGTATGATCTGTTGGAGTGGTAATTTTGCTGGTAGTTATAGCTAGGGCCTTTTTAGGCTATGTTCTCCCATGAGGACAAATATCTTTTTGGGGGGCAACGGTTATTACAAGTTTATTTTCAGCACTCCCCGTTATTGGGAGAGATGTGGTAATCTGATTGTGAGGAGGGTTTTCGGTAGACAACGCTACTCTTACACGATTTTTTGGGCTTCATTTTGTGTTGCCTTTTATGGTAGCAGGGTTAGCAGGATTACACCTGTTCTTCCTTCACGACCAAGGGTCGTCAAATCCATTGGGGGTAAGGTCTGATTACAACAAGGTTGTTTTTCATCATTACTATGTGCTTAAAGATTTGGTGGGGGTTTGTTTCTTTTTAGGGCCTTTGCTCGGTCTGGTGTATTTTAACCCCTGGCTTCTGGGGGACCCTGAAAATTTTATTGAAGGAAACCCACTGGTTACACCCCACCATATTCAGCCGGAATGATATTTTCTATTCGCTTACGCTATTTTGCGGGCTGTGCCTAATAAGCTTGGGGGGGTAGTCGCCTTGGCGGCTGCGGTGCTGGTGTTATTTTACATACCCTTTAGTCCTAAAACATCGATCAAAGGGTTATCCTTTTATCCGGGGGGTAAGAGTTTGTTTTTTAGAATAATTGGGGTGTTTTTTCTGCTCACCTGGGCGGGTTCTAAGCCGGTTGAAGAGCCCTATGTTGTAATTAGGCAAGTTCTAAGGTTTTTATATTTTGCGTATTTTATTTTAATTGGGCCCGTAACACGGATTCAAGATAAGTTGCTGTAA

RsPt_m7 1 ATTTATAAGGCTTCCCGGTTTAGGCCGTTAGTATCAGTAGTCCGGCACACCGTTATTACTTTACCTACCCCTATTAATATTTCAACCTGGTGAAATTTTGGTTCACTTCTAGGGGTTTGTCTAGTAATCCAAATTTTTAGGGGGTTAATTCTAAGCCTTCATTTTACGGCTCATACAGATCTCAGATTTTATGTGGTCATTCAAAGGTTTGGAGATGTCTATTACAATTGGTGGTTTCGTGCTTTTCATGCCAATGGTGCTAGGTTCTTTTTTATTGGTTTGTACTTACACATTGCGCGAGGGCTATATTACGGATCTTATGTTTTTAAGGAAGTATGATCTGTTGGAGTGGTAATTTTGCTGGTAGTTATAGCTAGGGCCTTTTTAGGCTATGTTCTCCCATGAGGACAAATATCTTTTTGGGGGGCAACGGTTATTACAAGTTTATTTTCAGCACTCCCCGTTATTGGGAGAGATGTGGTAATCTGATTGTGAGGAGGGTTTTCGGTAGACAACGCTACTCTTACACGATTTTTTGGGCTTCATTTTGTGTTGCCTTTTATGGTAGCAGGGTTAGCAGGATTACACCTGTTCTTCCTTCACGACCAAGGGTCGTCAAATCCATTGGGGGTAAGGTCTGATTACAACAAGGTTGTTTTTCATCATTACTATGTGCTTAAAGATTTAGTGGGGGTTTGTTTCTTTTTAGGGCCTTTGCTCGGTCTGGTGTATTTTAACCCCTGGCTTCTGGGGGACCCTGAAAATTTTATTGAAGGAAACCCACTGGTTACACCCCACCATATTCAGCCGGAATGATATTTTCTATTCGCTTACGCTATTTTGCGGGCTGTGCCTAATAAGCTTGGGGGGGTAGTCGCCTTGGCGGCTGCGGTGCTGGTATTATTTTACATACCCTTTAGTCCTAAAACATCGATCAAAGGGTTATCCTTTTACCCGGGGGGTAAGAGTTTGTTTTTTAGAATAATTGGGGTGTTTTTTCTGCTCACTTGGGCGGGTTCTAAGCCGGTTGAAGAGCCCTATGTTGTAATTAGGCAAGTTCTGAGGTTTTTATATTTTGCGTATTTTATTTTAATTGGGCCCGTAACACGGATTCAAGATAAGTTGCTGTAA

RsPt_m6 2 ATTTATAAGGCTTCCCGGTTTAGGCCGTTAGTATCAGTAGTCCGGCACACCGTTATTACTTTACCTACCCCTATTAATATTTCAACCTGGTGAAATTTTGGTTCACTTCTAGGGGTTTGTCTAGTAATCCAAATTTTTAGGGGGTTAATTCTAAGCCTTCATTTTACGGCTCATACAGATCTCAGATTTTATGTGGTCATTCAAAGGTTTGGAGATGTCTATTACAATTGGTGGTTTCGTGCTTTTCATGCCAATGGTGCTAGGTTCTTTTTTATTGGTTTGTACTTACACATTGCGCGAGGGCTATATTACGGATCTTATGTTTTTAAGGAAGTATGATCTGTTGGAGTGGTAATTTTGCTGGTAGTTATAGCTAGGGCCTTTTTAGGCTATGTTCTCCCATGAGGACAAATATCTTTTTGGGGGGCAACGGTTATTACAAGTTTATTTTCAGCACTCCCCGTTATTGGGAGAGATGTGGTAATCTGATTGTGAGGAGGGTTTTCGGTAGACAACGCTACTCTTACACGATTTTTTGGGCTTCATTTTGTGTTGCCTTTTATGGTAGCAGGGTTAGCAGGATTACACCTGTTCTTCCTTCACGACCAAGGGTCGTCAAATCCATTGGGGGTAAGGTCTGATTACAACAAGGTTGTTTTTCATCATTACTATGTGCTTAAAGATTTAGTGGGGGTTTGTTTCTTTTTAGGGCCTTTGCTCGGTCTGGTGTATTTTAACCCCTGGCTTCTGGGGGACCCTGAAAATTTTATTGAAGGAAACCCACTGGTTACACCCCACCATATTCAGCCGGAATGATATTTTCTATTCGCTTACGCTATTTTGCGGGCTGTGCCTAATAAGCTTGGGGGGGTAGTCGCCTTGGCGGCTGCGGTGCTGGTATTATTTTACATACCCTTTAGTCCTAAAACATCGATCAAAGGGTTATCCTTTTACCCGGGGGGTAAGAGTTTGTTTTTTAGAATAATTGGGGTGTTTTTTCTGCTCACCTGGGCGGGTTCTAAGCCGGTTGAAGAGCCCTATGTTGTAATTAGGCAAGTTCTAAGGTTTTTATATTTTGCGTATTTTATTTTAATTGGGCCCGTAACACGGATTCAAGATAAGTTGCTGTAA

RsPt_f2 2 ATTTATAAGGCTTCCCGGTTTAGGCCGTTAGTATCAGTAGTCCGGCACACCGTTATTACTTTACCTACCCCTATTAATATTTCAACCTGGTGAAATTTTGGTTCACTTCTAGGGGTTTGTCTAGTAATCCAAATTTTTAGGGGGTTAATTCTAAGCCTTCATTTTACGGCTCATACAGATCTCAGATTTTATGTGGTCATTCAAAGGTTTGGAGATGTCTATTACAATTGGTGGTTTCGTGCTTTTCATGCCAATGGTGCTAGGTTCTTTTTTATTGGTTTGTACTTACACATTGCGCGAGGGCTATATTACGGATCTTATGTTTTTAAGGAAGTATGATCTGTTGGAGTGGTAATTTTGCTGGTAGTTATAGCTAGGGCCTTTTTAGGCTATGTTCTCCCATGAGGACAAATATCTTTTTGGGGGGCAACGGTTATTACAAGTTTATTTTCAGCACTCCCCGTTATTGGGAGAGATGTGGTAATCTGATTGTGAGGAGGGTTTTCGGTAGACAACGCTACTCTTACACGATTTTTTGGGCTTCATTTTGTGTTGCCTTTTATGGTAGCAGGGTTAGCAGGATTACACCTGTTCTTCCTTCACGACCAAGGGTCGTCAAATCCATTGGGGGTAAGGTCTGATTACAACAAGGTTGTTTTTCATCATTACTATGTGCTTAAAGATTTGGTGGGGGTTTGTTTCTTTTTAGGGCCTTTGCTCGGTCTGGTGTATTTTAACCCCTGGCTTCTGGGGGACCCTGAAAATTTTATTGAAGGAAACCCACTGGTTACACCCCACCATATTCAGCCGGAATGATATTTTCTATTCGCTTACGCTATTTTGCGGGCTGTGCCTAATAAGCTTGGGGGGGTAGTCGCCTTGGCGGCTGCGGTGCTGGTATTATTTTACATACCCTTTAGTCCTAAAACATCGATCAAAGGGTTATCCTTTTATCCGGGGGGTAAGAGTTTGTTTTTTAGAATAATTGGGGTGTTTTTTCTGCTCACCTGGGCGGGTTCTAAGCCGGTTGAAGAGCCCTATGTTGTAATTAGGCAAGTTCTAAGGTTTTTATATTTTGCGTATTTTATTTTAATTGGGCCCGTAACACGGATTCAAGATAAGTTGCTGTAA

RsPt_f4 1 ATTTATAAGGCTTCCCGGTTTAGGCCGTTAGTATCAGTAGTCCGGCACACCGTTATTACTTTACCTACCCCTATTAATATTTCAACCTGGTGAAATTTTGGTTCACTTCTAGGGGTTTGTCTAGTAATCCAAATTTTTAGGGGGTTAATTCTAAGCCTTCATTTTACGGCTCATACAGATCTCAGATTTTATGTGGTCATTCAAAGGTTTGGAGATGTTTATTACAATTGGTGGTTTCGTGCTTTTCATGCCAATGGTGCTAGGTTCTTTTTTATTGGTTTGTACTTACACATTGCGCGAGGGCTATATTACGGATCTTATGTTTTTAAGGAAGTATGATCTGTTGGAGTGGTAATTTTGCTGGTAGTTATAGCTAGGGCCTTTTTAGGCTATGTTCTCCCATGAGGACAAATATCTTTTTGGGGGGCAACGGTTATTACAAGTTTATTTTCAGCACTCCCCGTTATTGGGAGAGATGTGGTAATCTGATTGTGAGGAGGGTTTTCGGTAGACAACGCTACTCTTACACGATTTTTTGGGCTTCATTTTGTGTTGCCTTTTATGGTAGCAGGGTTAGCAGGATTACACCTGTTCTTCCTTCACGACCAAGGGTCGTCAAATCCATTGGGGGTAAGGTCTGATTACAACAAGGTTGTTTTTCATCATTACTATGTGCTTAAAGATTTAGTGGGGGTTTGTTTCTTTTTAGGGCCTTTGCTCGGTCTGGTGTATTTTAACCCCTGGCTTCTGGGGGACCCTGAAAATTTTATTGAAGGAAACCCACTGGTTACACCCCACCATATTCAGCCGGAATGATATTTTCTATTTGCTTACGCTATTTTGCGGGCTGTGCCTAATAAGCTTGGGGGGGTAGTCGCCTTGGCGGCTGCGGTGCTGGTATTATTTTACATACCCTTTAGTCCTAAAACATCGATCAAAGGGTTATCCTTTTACCCCGGGGGTAAGAGTTTGTTTTTTAGAATAATTGGGGTGTTTTTTCTGCTCACCTGGGCGGGTTCTAAGCCGGTTGAAGAGCCCTATGTTGTAATTAGGCAAGTTCTAAGGTTTTTATATTTTGCGTATTTTATTTTAATTGGGCCCGTAACACGGATTCAAGATAAGTTGCTGTAA

RsPt_m2 1 ATTTATAAGGCTTCCCGGTTTAGGCCGTTAGTATCAGTAGTCCGGCACACCGTTATTACTTTACCTACCCCTATTAATATTTCAACCTGGTGAAATTTTGGTTCACTTCTAGGGGTTTGTCTAGTAATCCAAATTTTTAGGGGGTTAATTCTAAGCCTTCATTTTACGGCTCATACAGATCTCAGATTTTATGTGGTCATTCAAAGGTTTGGAGATGTCTATTACAATTGGTGGTTTCGTGCTTTTCATGCCAATGGTGCTAGGTTCTTTTTTATTGGTTTGTACTTACACATTGCGCGAGGGCTATATTACGGATCTTATGTTTTTAAGGAAGTATGATCTGTTGGAGTGGTAATTTTGCTGGTAGTTATAGCTAGGGCCTTTTTAGGCTATGTTCTCCCATGAGGACAAATATCTTTTTGGGGGGCAACGGTTATTACAAGTTTATTTTCAGCACTCCCCGTTATTGGGAGAGATGTGGTAATCTGATTGTGAGGAGGGTTTTCGGTAGACAACGCTACTCTTACACGATTTTTTGGGCTTCATTTTGTGTTGCCTTTTATGGTAGCAGGGTTAGCAGGATTACACCTGTTCTTCCTTCACGACCAAGGGTCGTCAAATCCATTGGGGGTAAGGTCTGATTACAACAAGGTTGTTTTTCATCATTACTATGTGCTTAAAGATTTAGTGGGGGTTTGTTTCTTTTTAGGGCCTTTGCTCGGTCTGGTGTATTTTAACCCCTGGCTTCTGGGGGACCCTGAAAATTTTATTGAAGGAAACCCACTGGTTACACCCCACCATATTCAGCCGGAATGATATTTTCTATTCGCTTACGCTATTTTGCGGGCTGTGCCTAATAAGCTTGGAGGGGTAGTCGCCTTGGCGGCTGCGGTGCTGGTATTATTTTACATACCCTTTAGTCCTAAAACATCGATCAAAGGGTTATCCTTTTACCCGGGGGGTAAGAGTTTGTTTTTTAGAATAATTGGGGTGTTTTTTCTGCTCACCTGGGCGGGTTCTAAGCCGGTTGAAGAGCCCTATGTTGTAATTAGGCAAGTTCTAAGGTTTTTATATTTTGCGTATTTTATTTTAATTGGGCCCGTAACACGGATTCAAGATAAGTTGCTGTAA

RsPt_f3 2 ATTTATAAGGCTTCCCGGTTTAGGCCGTTAGTATCAGTAGTCCGGCACACCGTTATTACTTTACCTACCCCTATTAATATTTCAACCTGGTGAAATTTTGGTTCACTTCTAGGGGTTTGTCTAGTAATCCAAATTTTTAGGGGGTTAATTCTAAGCCTTCATTTTACGGCTCATACAGATCTCAGATTTTATGTGGTCATTCAAAGGTTTGGAGATGTTTATTACAATTGGTGGTTTCGTGCTTTTCATGCCAATGGTGCTAGGTTCTTTTTTATTGGTTTGTACTTACACATTGCGCGAGGGCTATATTACGGATCTTATGTTTTTAAGGAAGTATGATCTGTTGGAGTGGTAATTTTGCTGGTAGTTATAGCTAGGGCCTTTTTAGGCTATGTTCTCCCATGAGGACAAATATCTTTTTGGGGGGCAACGGTTATTACAAGTTTATTTTCAGCACTCCCCGTTATTGGGAGAGATGTGGTAATCTGATTGTGAGGAGGGTTTTCGGTAGACAACGCTACTCTTACACGATTTTTTGGGCTTCATTTTGTGTTGCCTTTTATGGTAGCAGGGTTAGCAGGATTACACCTGTTCTTCCTTCACGACCAAGGGTCGTCAAATCCATTGGGGGTAAGGTCTGATTACAACAAGGTTGTTTTTCATCATTACTATGTGCTTAAAGATTTAGTGGGGGTTTGTTTCTTTTTAGGGCCTTTGCTCGGTCTGGTGTATTTTAACCCCTGGCTTCTGGGGGACCCTGAAAATTTTATTGAAGGAAACCCACTGGTTACACCCCACCATATTCAGCCGGAATGATATTTTCTATTCGCTTACGCTATTTTGCGGGCTGTGCCTAATAAGCTTGGGGGGGTAGTCGCCTTGGCGGCTGCGGTGCTGGTATTATTTTACATACCCTTTAGTCCTAAAACATCGATCAAAGGGTTATCCTTTTACCCGGGGGGTAAGAGTTTGTTTTTTAGAATAATTGGGGTGTTTTTTCTGCTCACCTGGGCGGGTTCTAAGCCGGTTGAAGAGCCCTATGTTGTAATTAGGCAAGTTCTAAGGTTTTTATATTTTGCGTATTTTATTTTAATTGGGCCCGTAACACGGATTCAAGATAAGTTGCTGTAA

}

SampleName="FR1 21"

SampleSize= 10

SampleData= {

FR1_m3 5 ATTTATAAGGCTTCCCGGTTTAGGCCGTTAGTATCAGTAGTCCGGCACACCGTTATTACTTTACCTACCCCTATTAATATTTCAACCTGGTGAAATTTTGGTTCACTCCTAGGGGTTTGTCTAGTAATCCAAATTTTTAGGGGGTTAATTCTAAGCCTTCATTTTACGGCTCATACAGATCTCAGATTTTATGTGGTCATTCAAAGGTTTGGAGATGTTTATTACAATTGGTGGTTTCGTGCTTTTCATGCCAATGGTGCTAGGTTCTTTTTTATTGGTTTGTACCTACACATTGCGCGAGGGCTATACTACGGATCTTATGTTTTTAAGGAAGTATGATCTGTTGGAGTGGTAATTTTGCTGGTAGTTATAGCTAGGGCCTTTTTAGGCTATGTTCTCCCATGAGGACAAATATCTTTTTGGGGGGCAACGGTTATTACAAGTTTATTTTCAGCACTCCCCGTTATTGGGAGAGATGTGGTAATCTGACTGTGAGGGGGGTTTTCAGTAGACAACGCTACTCTTACCCGATTTTTTGGGCTTCATTTTGTGTTGCCTTTTATGGTAGCAGGGTTAGCAGGATTGCACCTGTTCTTCCTTCACGACCAAGGGTCGTCAAATCCATTAGGGGTAAGGTCTGATTACAACAAGGTTGTTTTTCATCATTACTATGTGCTTAAAGATTTAGTGGGGGTTTGTTTCTTTTTAGGGCCTTTGCTAGGTCTGGTGTATTTTAACCCCTGGCTTCTGGGGGACCCTGAAAATTTTATTGAAGGAAACCCACTGGTTACACCCCACCATATTCAGCCGGAATGATATTTTCTATTCGCTTACGCTATTTTGCGGGCTGTGCCTAATAAGCTTGGGGGGGTAGTCGCCTTGGCGGCTGCGGTGCTGGTATTATTTTACATACCCTTTAGTCCTAAAACATCGATCAAAGGGTTATCCTTTTACCCGGGGGGTAAGAGTTTGTTTTTTAGAATAATTGGGGTGTTTTTTCTGCTCACCTGGGCGGGTTCTAAGCCGGTTGAAGAGCCTTATGTTGTAATTAGGCAAGTTCTAAGGTTTTTATATTTTGCGTATTTTATTTTAATTGGGCCCGTAACACGGGTTCAAGATAAGTTGCTGTAA

FR1_m7 3 ATTTA?AAGG?TTCCCGGTTTAGGCCGTTAGTATCAGTAGTCCGGCACACCGTTATTACTTTACCTACCCCTATTAATATTTCAACCTGGTGAAATTTTGGTTCACTCCTAGGGGTTTGTCTAGTAATCCAAATTTTTAGGGGGTTAATTCTAAGCCTTCATTTTACGGCTCATACAGATCTCAGATTTTATGTGGTCATTCAAAGGTTTGGAGATGTTTATTACAATTGGTGGTTTCGTGCTTTTCATGCCAATGGTGCTAGGTTCTTTTTTATTGGTTTGTACCTACACATTGCGCGAGGGCTATACTACGGATCTTATGTTTTTAAGGAAGTATGATCTGTTGGAGTGGTAATTTTGCTGGTAGTTATAGCTAGGGCCTTTTTAGGCTATGTTCTCCCATGAGGACAAATATCTTTTTGGGGGGCAACGGTTATTACAAGTTTATTTTCAGCACTCCCCGTTATTGGGAGAGATGTGGTAATCTGACTGTGAGGGGGGTTTTCAGTAGACAACGCTACTCTTACCCGATTTTTTGGGCTTCATTTTGTGTTGCCTTTTATGGTAGCAGGGTTAGCAGGATTGCACCTGTTCTTCCTTCACGACCAAGGGTCGTCAAATCCATTAGGGGTAAGGTCTGATTACAACAAGGTTGTTTTTCATCATTACTATGTGCTTAAAGATTTAGTGGGGGTTTGTTTCTTTTTAGGGCCTTTGCTAGGTCTGGTGTATTTTAACCCCTGGCTTCTGGGGGACCCTGAAAATTTTATTGAAGGAAACCCACTGGTTACACCCCACCATATTCAGCCGGAATGATATTTTCTATTCGCTTACGCTATTTTGCGGGCTGTGCCTAATAAGCTTGGGGGGGTAGTCGCCTTGGCGGCTGCGGTGCTGGTATTATTTTACATACCCTTTAGTCCTAAAACATCGATCAAAGGGTTATCCTTTTACCCAGGGGGTAAGAGTTTGTTTTTTAGAATAATTGGGGTGTTTTTTCTGCTCACCTGGGCGGGTTCTAAGCCGGTTGAAGAGCCTTATGTTGTAATTAGGCAAGTTCTAAGGTTTTTATATTTTGCGTATTTTATTTTAATTGGGCCCGTAACACGGGTTCAAGATAAGTTGCTGTAA

FR1_m5 1 ATTTA?AAGG?TTCCCGGTTTAGGCCGTTAGTATCAGTAGTCCGGCACACCGTTATTACTTTACCTACCCCTATTAATATTTCAACCTGGTGAAATTTTGGTTCACTCCTAGGGGTTTGTCTAGTAATCCAAATTTTTAGGGGGTTAATTCTAAGCCTTCATTTTACGGCTCATACAGATCTCAGATTTTATGTGGTCATTCAAAGGTTTGGAGATGTTTATTACAATTGGTGGTTTCGTGCTTTTCATGCCAATGGTGCTAGGTTCTTTTTTATTGGTTTGTACCTACACATTGCGCGAGGGCTATACTACGGATCTTATGTTTTTAAGGAAGTATGATCTGTTGGAGTGGTAATTTTGCTGGTAGTTATAGCTAGGGCCTTTTTAGGCTATGTTCTCCCATGAGGACAAATATCTTTTTGGGGGGCAACGGTTATTACAAGTTTATTTTCAGCACTCCCCGTTATTGGGAGAGATGTGGTAATCTGACTGTGAGGGGGGTTTTCAGTAGACAACGCTACTCTTACCCGATTTTTTGGGCTTCATTTTGTGTTGCCTTTTATGGTAGCAGGGTTAGCAGGATTGCACCTGTTCTTCCTTCACGACCAAGGGTCGTCAAATCCATTAGGGGTAAGGTCTGATTACAACAAGGTTGTTTTTCATCATTACTATGTGCTTAAAGATTTAGTGGGGGTTTGTTTCTTTTTAGGGCCTTTGCTAGGTCTGGTGTATTTTAACCCCTGGCTTCTGGGGGACCCTGAAAATTTTATTGAAGGAAACCCATTGGTTACACCCCACCATATTCAGCCGGAATGATATTTTCTATTCGCTTACGCTATTTTGCGGGCTGTGCCTAATAAGCTTGGGGGGGTAGTCGCCTTGGCGGCTGCGGTGCTGGTATTATTTTACATACCCTTTAGTCCTAAAACATCAATCAAAGGGTTATCCTTTTACCCGGGGGGTAAGAGTTTGTTTTTTAGAATAATTGGGGTGTTTTTTCTGCTCACCTGGGCGGGTTCTAAGCCGGTTGAAGAGCCTTATGTTGTAATTAGGCAAGTTCTAAGGTTTTTATATTTTGCGTATTTTATTTTAATTGGGCCCGTAACACGGGTTCAAGATAAGTTGCTGTAA

FR1_m6 1 ATTTA?AAGG?TTCCCGGTTTAGGCCGTTAGTATCAGTAGTCCGGCACACCGTTATTACTTTACCTACCCCTATTAATATTTCAACCTGGTGAAATTTTGGTTCACTCCTAGGGGTTTGTCTAGTAATCCAAATTTTTAGGGGGTTAATTCTAAGCCTTCATTTTACGGCTCATACAGATCTCAGATTTTATGTGGTCATTCAAAGGTTTGGAGATGTTTATTACAATTGGTGGTTTCGTGCTTTTCATGCCAATGGTGCTAGGTTCTTTTTTATTGGTTTGTACCTACACATTGCGCGAGGGCTATACTACGGATCTTATGTTTTTAAGGAAGTATGATCTGTTGGAGTGGTAATTTTGCTGGTAGTTATAGCTAGGGCCTTTTTAGGCTATGTTCTCCCATGAGGACAAATATCTTTTTGGGGGGCAACGGTTATTACAAGTTTATTTTCAGCACTCCCCGTTATTGGGAGAGATGTGGTAATCTGACTGTGAGGGGGGTTTTCAGTAGACAACGCTACTCTTACCCGATTTTTTGGGCTTCATTTTGTGTTGCCTTTTATGGTAGCAGGGTTAGCAGGATTGCACCTGTTCTTCCTTCACGACCAAGGGTCGTCAAATCCATTAGGGGTAAGGTCTGATTACAACAAGGTTGTTTTTCATCATTACTATGTGCTTAAAGATTTAGTGGGGGTTTGTTTCTTTTTAGGGCCTTTGCTAGGTCTGGTGTATTTTAACCCCTGGCTTCTGGGGGACCCTGAAAATTTTATTGAAGGAAACCCACTGGTTACACCCCACCATATTCAGCCGGAATGATATTTTCTATTCGCTTACGCTATTTTGCGGGCTGTGCCTAATAAGCTTGGGGGGGTAGTCGCCTTGGCGGCTGCGGTGCTGGTATTATTTTACATACCCTTTAGTCCTAAAGCATCGATCAAAGGGTTATCCTTTTACCCGGGGGGTAAGAGTTTGTTTTTTAGAATAATTGGGGTGTTTTTTCTGCTCACCTGGGCGGGTTCTAAGCCGGTTGAAGAGCCTTATGTTGTAATTAGGCAAGTTCTAAGGTTTTTATATTTTGCGTATTTTATTTTAATTGGGCCCGTAACACGGGTTCAAGATAAGTTGCTGTAA

}

SampleName="FR2 22"

SampleSize= 10

SampleData= {

FR1_m3 1

FR2_m8 3 ATTTA?AAGGCTTCCCGGTTTAGGCCGTTAGTATCAGTAGTCCGGCACACCGTTATTACTTTACCTACCCCTATTAATATTTCAACCTGGTGAAATTTTGGTTCACTTCTAGGGGTTTGTCTAGTAATCCAAATTTTTAGGGGGTTAATTCTAAGCCTTCATTTTACGGCTCATACAGATCTCAGATTTTATGTGGTCATTCAAAGGTTTGGAGATGTCTATTACAATTGGTGGTTTCGTGCTTTTCATGCCAATGGTGCTAGGTTCTTTTTTATTGGTTTGTACTTACACATTGCGCGAGGGCTATATTACGGATCTTATGTTTTTAAGGAAGTATGATCTGTTGGAGTGGTAATTTTGCTGGTAGTTATAGCTAGGGCCTTTTTAGGCTATGTTCTCCCATGAGGACAAATATCTTTTTGGGGGGCAACGGTTATTACAAGTTTATTTTCAGCACTCCCCGTTATTGGGAGAGATGTGGTAATCTGATTGTGAGGAGGGTTTTCAGTAGACAACGCTACTCTTACACGATTTTTTGGGCTTCATTTTGTGTTGCCTTTTATGGTAGCAGGATTAGCAGGATTACACCTGTTCTTCCTTCACGACCAAGGGTCGTCAAATCCATTGGGGGTAAGGTCTGATTACAACAAGGTTGTTTTTCATCATTACTATGTGCTTAAAGATTTAGTGGGGGTTTGTTTCTTTTTAGGGCCTTTGCTCGGTCTGGTGTATTTTAACCCCTGGCTTCTGGGGGACCCTGAAAATTTTATTGAAGGAAACCCACTGGTTACACCCCACCATATTCAGCCGGAATGATATTTTCTATTCGCTTACGCTATTTTGCGGGCTGTGCCTAATAAGCTTGGGGGGGTAGTCGCCTTGGCGGCTGCGGTGCTGGTATTATTCTACATACCCTTTAGTCCTAAAACATCGATCAAAGGGTTATCCTTTTACCCGGGGGGTAAGAGTTTGTTTTTTAGAATAATTGGGGTGTTTTTTCTGCTCACCTGGGCGGGTTCTAAGCCGGTTGAAGAGCCCTATGTTGTAATTAGGCAAGTTCTAAGGTTTTTATATTTTGCGTATTTTATTTTAATTGGGCCCGTAACACGGATTCAAGATAAGTTGCTGTAA

FR2_m7 6 ATTTA?AAGGCTTCCCGGTTTAGGCCGTTAGTATCAGTAGTCCGGCACACCGTTATTACTTTACCTACCCCTATTAATATTTCAACCTGGTGAAATTTTGGTTCACTCCTAGGGGTTTGTCTAGTAATCCAAATTTTTAGGGGGTTAATTCTAAGCCTTCATTTTACGGCTCATACAGATCTCAGATTTTATGTGGTCATTCAAAGGTTTGGAGATGTTTATTACAATTGGTGGTTTCGTGCTTTTCATGCCAATGGTGCTAGGTTCTTTTTTATTGGTTTGTACTTACACATTGCGCGAGGGCTATACTACGGATCTTATGTTTTTAAGGAAGTATGATCTGTTGGAGTGGTAATTTTGCTGGTAGTTATAGCTAGGGCCTTTTTAGGCTATGTTCTCCCATGAGGACAAATATCTTTTTGGGGGGCAACGGTTATTACAAGTTTATTTTCAGCACTCCCCGTTATTGGGAGAGATGTGGTAATCTGACTGTGAGGGGGGTTTTCAGTAGACAACGCTACTCTTACCCGATTTTTTGGGCTTCATTTTGTGTTGCCTTTTATGGTAGCAGGGTTAGCAGGATTGCACCTGTTCTTCCTTCACGACCAAGGGTCGTCAAATCCATTAGGGGTAAGGTCTGATTACAACAAGGTTGTTTTTCATCATTACTATGTGCTTAAAGATTTAGTGGGGGTTTGTTTCTTTTTAGGGCCTTTGCTAGGTCTGGTGTATTTTAACCCCTGGCTTCTGGGGGACCCTGAAAATTTTATTGAAGGAAACCCACTGGTTACACCCCACCATATTCAGCCGGAATGATATTTTCTATTCGCTTACGCTATTTTGCGTGCTGTGCCTAATAAGCTTGGGGGGGTAGTCGCCTTGGCGGCTGCGGTGCTGGTATTATTTTACATACCCTTTAGTCCTAAAACATCGATCAAAGGGTTATCCTTTTACCCGGGGGGTAAGAGTTTGTTTTTTAGAATAATTGGGGTGTTTTTTCTGCTCACCTGGGCGGGTTCTAAGCCGGTTGAAGAGCCTTATGTTGTAATTAGGCAAGTTCTAAGGTTTTTATATTTTGCGTATTTTATTTTAATTGGGCCCGTAACACGGGTTCAAGATAAGTTGCTGTAA

}

SampleName="CCR1 23"

SampleSize= 10

SampleData= {

CCR1_f3 8 ATTTATAAGGCTTCTCGATTTAGACCTCTTGCTAGAGTTATTCGCCACACCGTTATTACTTTACCTACGCCGGTTAATATTTCAACATGGTGAAACTTTGGCTCTCTATTAGGTGTTTGTCTGGCAACTCAGATTTTTAGGGGGCTGGTTTTAAGACTACATTTTACAGCTCATACGGATTTAAGGTTTTATGTAGTTATCCAAAGGTTTGGAGATGTTTACTATAATTGGTGATTTCGGGCTCTCCATGCTAATGGAGCTAGTTTCTTTTTTATCGGCCTTTATTTACATATTGCTCGAGGTATATATTATGGGTCTTATGTTTTCAAGGAAGTGTGATCTGTAGGTGTAGTCATCTTGCTTTTAGTAATAGCTAGAGCCTTTTTAGGCTATGTCCTCCCTTGAGGGCAGATATCCTTCTGAGGGGCAACAGTTATTACAAGGTTGTTTTCAGCCCTTCCCATCATTGGGAGAGATGTTGTGATTTGGCTATGAGGGGGGTTTTCGGTGGACAATGCGACACTTACTCGATTTTTTGGTCTTCATTTTCTCTTGCCCTTTACAGTAGCCGGGCTAGCCGGGCTACACTTATTCTTCTTGCACGACCAAGGGTCGTCAAACCCTTTAGGGGTGAGATCCGATTTTAACAAAGTTGTTTTCCATCACTATTACGTGTTAAAAGATCTGGTAGGAGTGTGTTTTTTTGTAGGACCATTACTGGGATTAGTCTATTTTTATCCGTGGCTTCTGGGGGACCCCGAAAATTTTATTGAGGGCAACCCCTTGGTTACACCTCACCACATTCAACCAGAGTGGTACTTTTTGTTTGCTTACGCAATTTTGCGGGCTGTGCCTAACAAATTAGGGGGCGTGGTTGCCCTGGCTGCTGCAGTGTTGATTTTGTTTTATATGCCTTTTAGACCAAAAACATCAATCAAAGGTTTATCTTTTTATCCTTGAGGAAAGGTTTTATTTTTTAGGTTAATTGGGGTATTTATTTTACTTACCTGGGCGGGATCCAAACCGGTGGAAGAGCCTTATGTGGTGGTTAGGCAGGTGCTAAGGTTTTTGTATTTCTTCTATTTCTTACTAATTGGACCATTAACTCGCATTCAGGATAAGTTGTTGTAA

CCR1_f1 1 ATTTATAAGGCTTCTCGATTTAGACCTCTTGCTAGAGTTATTCGCCACACCGTTATTACTTTACCTACGCCGGTTAATATTTCAACATGGTGAAACTTTGGCTCTCTATTAGGTGTTTGTCTGGCAACTCAGATTTTTAGGGGGCTGGTTTTAAGACTACATTTTACAGCTCATACGGATTTAAGGTTTTATGTAGTTATCCAAAGGTTTGGAGATGTTTACTATAATTGGTGATTTCGGGCTCTCCATGCTAATGGAGCTAGTTTCTTTTTTATCGGCCTTTATTTACATATTGCTCGAGGTATATATTATGGGTCTTATGTTTTCAAGGAAGTGTGATCTGTAGGTGTAGTCATCTTGCTTTTAGTAATAGCTAGAGCCTTTTTAGGCTATGTCCTCCCTTGAGGGCAGATATCCTTCTGAGGGGCAACAGTTATTACAAGGTTGTTTTCAGCCCTTCCCATCATTGGGAGAGATGTTGTGATTTGGCTATGAGGGGGGTTTTCGGTGGACAATGCGACACTTACTCGATTTTTTGGTCTTCATTTTCTCTTGCCCTTTACAGTAGCCGGGCTAGCCGGGCTACACTTATTCTTCTTGCACGACCAAGGGTCGTCAAACCCTTTAGGGGTGAGATCCGATTTTAACAAAGTTGTTTTCCATCACTATTACGTGTTAAAAGATCTGGTAGGAGTGTGTTTTTTTGTAGGACCATTACTGGGATTAGTCTATTTTTATCCGTGGCTTCTGGGGGACCCCGAAAATTTTATTGAGGGCAACCCCTTGGTTACACCTCACCACATTCAACCAGAGTGGTATTTTTTGTTTGCTTACGCAATTTTGCGGGCTGTGCCTAACAAATTAGGGGGCGTGGTTGCCCTGGCTGCTGCAGTGTTGATTTTGTTTTATATGCCTTTTAGACCAAAAACATCAATCAAAGGTTTATCTTTTTATCCTTGAGGAAAGGTTTTATTTTTTAGGTTAATTGGGGTATTTATTTTACTTACCTGGGCGGGATCCAAACCGGTGGAAGAGCCTTATGTGGTGGTTAGGCAGGTGCTAAGGTTTTTGTATTTCTTCTATTTCTTACTAATTGGACCATTAACTCGCATTCAGGATAAGTTGTTGTAA

CCR1_f2 1 ATTTATAAGGCTTCTCGATTTAGACCTCTCGCTAGAGTTATTCGCCACACCGTTATTACTTTACCTACGCCGGTTAATATTTCAACATGGTGAAACTTTGGCTCTCTATTAGGTGTTTGTCTGGCAACTCAGATTTTTAGGGGGCTGGTTTTAAGACTACATTTTACAGCTCATACGGATTTAAGGTTTTATGTAGTTATCCAAAGGTTTGGAGACGTTTACTATAATTGGTGATTTCGGGCTCTCCATGCTAATGGAGCTAGTTTCTTTTTTATCGGCCTTTATTTACATATTGCTCGAGGTATATATTATAGGTCTTATGTTTTCAAGGAAGTGTGATCTGTAGGTGTAGTCATCTTGCTTTTAGTAATAGCTAGAGCCTTTTTAGGCTATGTCCTCCCTTGAGGGCAGATATCCTTCTGAGGGGCAACAGTTATTACAAGGTTGTTTTCAGCCCTTCCCATCATTGGGAGCGATGTTGTGATTTGGCTATGAGGGGGGTTTTCGGTGGACAATGCGACACTTACTCGATTTTTTGGTATTCATTTTCTCTTGCCCTTTACAGTAGCCGGGCTAGCCGGGCTACACTTATTCTTCTTGCACGACCAAGGGTCGTCAAACCCTTTAGGGGTGAGATCCGATTTTAACAAAGTTGTTTTCCATCACTATTACGTGTTAAAAGATCTGGTAGGAGTGTGTTTTTTCGTAGGACCATTACTGGGACTAGTCTATTTTTATCCGTGGCTTCTGGGGGACCCCGAAAATTTTATTGAGGGCAACCCCTTGGTTACACCTCACCACATTCAACCAGAGTGGTACTTTTTGTTTGCTTACGCAATTTTGCGGGCTGTGCCTAACAAATTAGGGGGCGTGGTTGCCCTGGCTGCTGCAGTGTTGATTTTGTTTTATATGCCTTTTAGACCAAAAACATCAATCAAAGGTTTATCTTTTTATCCTTGAGGAAAGGTTTTATTTTTTAGGTTAATTGGGGTATTTATTTTACTTACCTGGGCGGGATCCAAACCGGTGGAAGAGCCTTATGTGGTGGTTAGGCAGGTGCTAAGGTTTTTGTATTTCTTCTATTTCTTACTAATTGGACCATTAACTCGCATTCAGGATAAGTTGTTGTAA

}

SampleName="CCR2 24"

SampleSize= 10

SampleData= {

CCR1_f3 1

CCR2_f3 8 ATTTATAAGGCTTCTCGATTTAGACCTCTCGCTAGAGTTATTCGCCACACCGTTATTACTTTACCTACGCCGGTTAATATTTCAACATGGTGAAACTTTGGCTCTCTATTAGGTGTTTGTCTGGCAACTCAGATTTTTAGGGGGCTGGTTTTAAGACTACATTTTACAGCTCATACGGATTTAAGGTTTTATGTAGTTATCCAAAGGTTTGGAGACGTTTACTATAATTGGTGATTTCGGGCTCTCCATGCTAATGGAGCTAGTTTCTTTTTTATCGGCCTTTATTTACATATTGCTCGAGGTATATATTATGGGTCTTATGTTTTCAAGGAAGTGTGATCTGTAGGTGTAGTCATCTTGCTTTTAGTAATAGCTAGAGCCTTTTTAGGCTATGTCCTCCCTTGAGGGCAGATATCCTTCTGAGGGGCAACAGTTATTACAAGGTTGTTTTCAGCCCTTCCCATCATTGGGAGAGATGTTGTGATTTGGCTATGAGGGGGGTTTTCGGTGGACAATGCGACACTTACTCGATTTTTTGGTCTTCATTTTCTCTTGCCCTTTACAGTAGCCGGGCTAGCCGGGCTACATTTATTCTTCTTGCACGACCAAGGGTCGTCAAACCCTTTAGGGGTGAGATCCGATTTTAACAAAGTTGTTTTCCATCACTATTACGTGTTAAAAGATCTGGTAGGAGTGTGTTTTTTCGTAGGACCATTACTGGGATTAGTCTATTTTTATCCGTGGCTTCTGGGGGACCCCGAAAATTTTATTGAGGGCAACCCCTTGGTTACACCTCACCACATTCAACCAGAGTGGTACTTTTTGTTTGCTTACGCAATTTTGCGGGCTGTGCCTAACAAATTAGGGGGCGTGGTTGCCCTGGCTGCTGCAGTGTTGATTTTGTTTTATATGCCTTTTAGACCAAAAACATCAATCAAAGGTTTATCTTTTTATCCTTGAGGAAAGGTTTTATTTTTTAGGTTAATTGGGGTATTTATTTTACTTACCTGGGCGGGATCCAAACCGGTGGAAGAGCCTTATGTGGTGGTTAGGCAGGTGCTAAGGTTTTTGTATTTCTTCTATTTCTTACTAATTGGACCATTAACTCGCATTCAGGATAAGTTGTTGTAA

CCR2_m11 1 ATTTATAAGGCTTCTCGATTTAGACCTCTCGCTAGAGTTATTCGCCACACCGTTATTACTTTACCTGCGCCGGTTAATATTTCAACATGGTGAAACTTTGGCTCTCTATTAGGTGTTTGTCTGGCAACTCAGATTTTTAGGGGGCTGGTTTTAAGACTACATTTTACAGCTCATACGGATTTAAGGTTTTATGTAGTTATCCAAAGGTTTGGAGACGTTTACTATAATTGGTGATTTCGGGCTCTCCATGCTAATGGAGCTAGTTTCTTTTTTATCGGCCTTTATTTACATATTGCTCGAGGTATATATTATAGGTCTTATGTTTTCAAGGAAGTGTGATCTGTAGGTGTAGTCATCTTGCTTTTAGTAATAGCTAGAGCCTTTTTAGGCTATGTCCTCCCTTGAGGGCAGATATCCTTCTGAGGGGCAACAGTTATTACAAGGTTGTTTTCAGCCCTTCCCATCATTGGGAGCGATGTTGTGATTTGGCTATGAGGGGGGTTTTCGGTGGACAATGCGACACTTACTCGATTTTTTGGTATTCATTTTCTCTTGCCCTTTACAGTAGCCGGGCTAGCCGGGCTACACTTATTCTTCTTGCACGACCAAGGGTCGTCAAACCCTTTAGGGGTGAGATCCGATTTTAACAAAGTTGTTTTCCATCACTATTACGTGTTAAAAGATCTGGTAGGAGTGTGTTTTTTCGTAGGACCATTACTGGGACTAGTCTATTTTTATCCGTGGCTTCTGGGGGACCCCGAAAATTTTATTGAGGGCAACCCCTTGGTTACACCTCACCACATTCAACCAGAGTGGTACTTTTTGTTTGCTTACGCAATTTTGCGGGCTGTGCCTAACAAATTAGGGGGCGTGGTTGCCCTGGCTGCTGCAGTGTTGATTTTGTTTTATATGCCTTTTAGACCAAAAACATCAATCAAAGGTTTATCTTTTTATCCTTGAGGAAAGGTTTTATTTTTTAGGTTAATTGGGGTATTTATTTTACTTACCTGGGCGGGATCCAAACCGGTGGAAGAGCCTTATGTGGTGGTTAGGCAGGTGCTAAGGTTTTTGTATTTCTTCTATTTCTTACTAATTGGACCATTAACTCGCATTCAGGATAAGTTGTTGTAA

}

SampleName="LH 25"

SampleSize= 10

SampleData= {

CCR1_f2 5

LH_m5 5 ATTTATAAGGCTTCTCGATTTAGACCTCTCGCTAGAGTTATTCGCCACACCGTTATTACTTTACCTACGCCGGTTAATATTTCAACATGGTGAAACTTTGGCTCTCTATTAGGTGTTTGTCTGGCAACTCAGATTTTTAGGGGGCTGGTTTTAAGACTACATTTTACAGCTCATACGGATTTAAGGTTTTATGTAGTTATCCAAAGGTTTGGAGACGTTTACTATAATTGGTGATTTCGGGCTCTCCATGCTAATGGAGCTAGTTTCTTTTTTATCGGCCTTTATTTACATATTGCTCGAGGTATATATTATGGGTCTTATGTTTTCAAGGAAGTGTGATCTGTAGGTGTAGTCATCTTGCTTTTAGTAATAGCTAGAGCCTTTTTAGGCTATGTCCTCCCTTGAGGGCAGATATCCTTCTGAGGGGCAACAGTTATTACAAGGTTGTTTTCAGCCCTTCCCATCATTGGGAGAGATGTTGTGATTTGGCTATGAGGGGGGTTTTCGGTGGACAATGCGACACTTACTCGATTTTTTGGTCTTCATTTTCTCTTGCCCTTTACAGTAGCCGGGCTAGCCGGGCTACACTTATTCTTCTTGCACGACCAAGGGTCGTCAAACCCTTTAGGGGTGAGATCCGATTTTAACAAAGTTGTTTTCCATCACTATTACGTGTTAAAAGATCTGGTAGGAGTGTGTTTTTTCGTAGGACCATTACTGGGACTAGTCTATTTTTATCCGTGGCTTCTGGGGGACCCCGAAAATTTTATTGAGGGCAACCCCTTGGTTACACCTCACCACATTCAACCAGAGTGGTACTTTTTGTTTGCTTACGCAATTTTGCGGGCTGTGCCTAACAAATTAGGGGGCGTGGTTGCCCTGGCTGCTGCAGTGTTGATTTTGTTTTATATGCCTTTTAGACCAAAAACATCAATCAAAGGTTTATCTTTTTATCCTTGAGGAAAGGTTTTATTTTTTAGGTTAATTGGGGTATTTATTTTACTTACCTGGGCGGGATCCAAACCGGTGGAAGAGCCTTATGTGGTGGTTAGGCAGGTGCTAAGGTTTTTGTATTTCTTCTATTTCTTACTAATTGGACCATTAACTCGCATTCAGGATAAGTTGTTGTAA

}

SampleName="NatBr1 26"

SampleSize= 10

SampleData= {

CCR1_f2 10

}

SampleName="NatBr2 27"

SampleSize= 10

SampleData= {

CCR1_f2 7

natbr2_m9 2 ATTTATAAGGCTTCTCGATTTAGACCTCTCGCTAGAGTTATTCGCCACACCGTTATTACTTTACCTACGCCGGTTAATATTTCAACATGGTGAAACTTTGGCTCTCTATTAGGTGTTTGTCTGGCAACTCAGATTTTTAGGGGGCTGGTTTTAAGACTACATTTTACAGCTCATACGGATTTAAGGTTTTATGTAGTTATCCAAAGGTTTGGAGACGTTTACTATAATTGGTGATTTCGGGCTCTCCATGCTAATGGAGCTAGTTTCTTTTTTATCGGCCTTTATTTACATATTGCTCGAGGTATATATTATAGGTCTTATGTTTTCAAGGAAGTGTGATCTGTAGGTGTAGTCATCTTGCTTTTAGTAATAGCTAGAGCCTTTTTAGGCTATGTCCTCCCTTGAGGGCAGATATCCTTCTGAGGGGCAACAGTTATTACAAGGTTGTTTTCAGCCCTTCCCATCATTGGGAGCGATGTTGTGATTTGGCTATGAGGGGGGTTTTCGGTGGACAATGCGACACTTACTCGATTTTTTGGTATTCATTTTCTCTTGCCCTTTACAGTAGCCGGGCTAGCCGGGCTACACTTATTCTTCTTGCACGACCAAGGGTCGTCAAACCCTTTAGGGGTGAGATCCGATTTTAACAAAGTTGTTTTCCATCACTATTACGTGTTAAAAGATCTGGTAGGAGTGTGTTTTTTCGTAGGACCATTACTGGGACTAGTCTATTTTTATCCGTGGCTTCTGGGGGACCCCGAAAATTTTATTGAGGGCAACCCCTTGGTTACACCTCACCACATTCAACCAGAGTGGTACTTTTTGTTCGCTTACGCAATTTTGCGGGCTGTGCCTAACAAATTAGGGGGCGTGGTTGCCCTGGCTGCTGCAGTGTTGATTTTGTTTTATATGCCTTTTAGACCAAAAACATCAATCAAAGGTTTATCTTTTTATCCTTGAGGAAAGGTTTTATTTTTTAGGTTAATTGGGGTATTTATTTTACTTACCTGGGCGGGATCCAAACCGGTGGAAGAGCCTTATGTGGTGGTTAGGCAGGTGCTAAGGTTTTTGTATTTCTTCTATTTCTTACTAATTGGACCATTAACTCGCATTCAGGATAAGTTGTTGTAA

natbr2_m10 1 ATTTATAAGGCTTCTCGATTTAGACCTCTCGCTAGAGTTATTCGCCACACCGTTATTACTTTACCTACGCCGGTTAATATTTCAACATGGTGAAACTTTGGCTCTCTATTAGGTGTTTGTCTGGCAACTCAGATTTTTAGGGGGCTGGTTTTAAGACTACATTTTACAGCTCATACGGATTTAAGGTTTTATGTAGTTATCCAAAGGTTTGGAGACGTTTACTATAATTGGTGATTTCGGGCTCTCCATGCTAATGGAGCTAGTTTCTTTTTTATCGGCCTTTATTTACATATTGCTCGAGGTATATATTATAGGTCTTATGTTTTCAAGGAAGTGTGATCTGTAGGTGTAGCCATCTTGCTTTTAGTAATAGCTAGAGCCTTTTTAGGCTATGTCCTCCCTTGAGGGCAGATATCCTTCTGAGGGGCAACAGTTATTACAAGGTTGTTTTCAGCCCTTCCCATCATTGGGAGCGATGTTGTGATTTGGCTATGAGGGGGGTTTTCGGTGGACAATGCGACACTTACTCGATTTTTTGGTATTCATTTTCTCTTGCCCTTTACAGTAGCCGGGCTAGCCGGGCTACACTTATTCTTCTTGCACGACCAAGGGTCGTCAAACCCTTTAGGGGTGAGATCCGATTTTAACAAAGTTGTTTTCCATCACTATTACGTGTTAAAAGATCTGGTAGGAGTGTGTTTTTTCGTAGGACCATTACTGGGACTAGTCTATTTTTATCCGTGGCTTCTGGGGGACCCCGAAAATTTTATTGAGGGCAACCCCTTGGTTACACCTCACCACATTCAACCAGAGTGGTACTTTTTGTTTGCTTACGCAATTTTGCGGGCTGTGCCTAACAAATTAGGGGGCGTGGTTGCCCTGGCTGCTGCAGTGTTGATTTTGTTTTATATGCCTTTTAGACCAAAAACATCAATCAAAGGTTTATCTTTTTATCCTTGAGGAAAGGTTTTATTTTTTAGGTTAATTGGGGTATTTATTTTACTTACCTGGGCGGGATCCAAACCGGTGGAAGAGCCTTATGTGGTGGTTAGGCAGGTGCTAAGGTTTTTGTATTTCTTCTATTTCTTACTAATTGGACCATTAACTCGCATTCAGGATAAGTTGTTGTAA

}

SampleName="SC3 28"

SampleSize= 10

SampleData= {

CCR1_f3 7

SC3_m8 1 ATTTATAAGGCTTCTCGATTTAGACCTCTTGCTAGAGTTATTCGCCACACCGTTATTACTTTACCTACGCCGGTTAATATTTCAACATGGTGAAACTTTGGCTCTCTATTAGGTGTTTGTCTGGCAACTCAGATTTTTAGGGGGCTGGTTTTAAGACTGCATTTTACAGCTCATACGGATTTAAGGTTTTATGTAGTTATCCAAAGGTTTGGAGATGTTTACTATAATTGGTGATTTCGGGCTCTCCATGCTAATGGAGCTAGTTTCTTTTTTATCGGCCTTTATTTACATATTGCTCGAGGTATATATTATGGGTCTTATGTTTTCAAGGAAGTGTGATCTGTAGGTGTAGTCATCTTGCTTTTAGTAATAGCTAGAGCCTTTTTAGGCTATGTCCTCCCTTGAGGGCAGATATCCTTCTGAGGGGCAACAGTTATTACAAGGTTGTTTTCAGCCCTTCCCATCATTGGGAGAGATGTTGTGATTTGGCTATGAGGGGGGTTTTCGGTGGACAATGCGACACTTACTCGATTTTTTGGTCTTCATTTTCTCTTGCCCTTTACAGTAGCCGGGCTAGCCGGGCTACACTTATTCTTCTTGCACGACCAAGGGTCGTCAAACCCTTTAGGGGTGAGATCCGATTTTAACAAAGTTGTTTTCCATCACTATTACGTGTTAAAAGATCTGGTAGGAGTGTGTTTTTTCGTAGGACCATTACTGGGATTAGTCTATTTTTATCCGTGGCTTCTGGGGGACCCCGAAAATTTTATTGAGGGCAACCCCTTGGTTACACCTCACCACATTCAACCAGAGTGGTACTTTTTGTTTGCTTACGCAATTTTGCGGGCTGTGCCTAACAAATTAGGGGGCGTGGTTGCCCTGGCTGCTGCAGTGTTGATTTTGTTTTATATGCCTTTTAGACCAAAAACATCAATCAAAGGTTTATCTTTTTATCCTTGAGGAAAGGTTTTATTTTTTAGGTTAATTGGGGTATTTATTTTACTTACCTGGGCGGGATCCAAACCGGTGGAAGAGCCTTATGTGGTGGTTAGGCAGGTGCTAAGGTTTTTGTATTTCTTCTATTTCTTACTAATTGGACCATTAACTCGCATTCAGGATAAGTTGTTGTAA

SC3_f7 2 ATTTATAAGGCTTCTCGATTTAGACCTCTCGCTAGAGTTATTCGCCACACCGTTATTACTTTACCTACGCCGGTTAATATTTCAACATGGTGAAACTTTGGCTCTCTATTAGGTGTTTGTCTGGCAACTCAGATTTTTAGGGGGCTGGTTTTAAGACTACATTTTACAGCTCATACTGATTTAAGGTTTTATGTAGTTATCCAAAGGTTTGGAGACGTTTACTATAATTGGTGATTTCGGGCTCTCCATGCTAATGGAGCTAGTTTCTTTTTTATCGGCCTTTATTTACATATTGCTCGAGGTATATATTATGGGTCTTATGTTTTCAAGGAAGTGTGATCTGTAGGTGTAGTCATCTTGCTTTTAGTAATAGCTAGAGCCTTTTTAGGCTATGTCCTCCCTTGAGGGCAGATATCCTTCTGAGGGGCAACAGTTATTACAAGGTTGTTTTCAGCCCTTCCCATCATTGGGAGAGATGTTGTGATTTGGCTATGAGGGGGGTTTTCGGTGGACAATGCGACACTTACTCGATTTTTTGGTCTTCATTTTCTCTTGCCCTTTACAGTAGCCGGGCTAGCCGGGCTACACTTATTCTTCTTGCACGACCAAGGGTCGTCAAACCCTTTAGGGGTGAGATCCGATTTTAACAAAGTTGTTTTCCATCACTATTACGTGTTAAAAGATCTGGTAGGAGCGTGTTTTTTCGTAGGACCATTACTGGGATTAGTCTATTTTTATCCGTGGCTTCTGGGGGACCCCGAAAATTTTATTGAGGGCAACCCCTTGGTTACACCTCACCACATTCAACCAGAGTGGTACTTTTTGTTTGCTTACGCAATTTTGCGGGCTGTGCCTAACAAATTAGGGGGCGTGGTTGCCCTGGCTGCTGCAGTGTTGATTTTGTTTTATATGCCTTTTAGACCAAAAACATCAATCAAAGGTTTATCTTTTTATCCTTGAGGAAAGGTTTTATTTTTTAGGTTAATTGGGGTATTTATTTTACTTACCTGGGCGGGATCCAAACCGGTGGAAGAGCCTTATGTGGTGGTTAGGCAGGTGCTAAGGTTTTTGTATTTCTTCTATTTCTTACTAATTGGACCATTAACTCGCATTCAGGATAAGTTGTTGTAA

}

SampleName="SC4 29"

SampleSize= 10

SampleData= {

CCR1_f2 9

SC3_f7 1

}

SampleName="SC5 30"

SampleSize= 10

SampleData= {

CCR1_f3 1

SC3_f7 9

}

SampleName="SCN 31"

SampleSize= 15

SampleData= {

CCR1_f2 2

CCR1_f3 10

SCn_2m 3 ATTTATAAGGCTTCTCGATTTAGACCTCTTGCTAGAGTTATTCGCCACACCGTTATTACTTTACCTACGCCGGTTAATATTTCAACATGGTGAAACTTTGGCTCTCTATTAGGTGTTTGTCTGGCAACTCAGATTTTTAGGGGGCTGGTTTTAAGACTACATTTTACAGCTCATACGGATTTAAGGTTTTATGTAGTTATCCAAAGGTTTGGAGATGTTTACTATAATTGGTGATTTCGGGCTCTCCATGCTAATGGAGCTAGTTTCTTTTTTATCGGCCTTTATTTACATATTGCTCGAGGTATATATTATGGGTCTTATGTTTTCAAGGAAGTGTGATCTGTAGGTGTAGTCATCTTGCTTTTAGTAATAGCTAGAGCCTTTTTAGGCTATGTCCTCCCTTGAGGGCAGATATCCTTCTGAGGGGCAACAGTTATTACAAGGTTGTTTTCAGCCCTTCCCATCATTGGGAGAGATGTTGTGATTTGGCTATGAGGGGGGTTTTCGGTGGACAATGCGACACTTACTCGATTTTTTGGTCTTCATTTTCTCTTGCCCTTTACAGTAGCCGGGCTAGCCGGGCTACACTTATTCTTCTTGCACGACCAAGGGTCGTCAAACCCTTTAGGGGTGAGATCCGATTTTAACAAAGTTGTTTTCCATCACTATTACGTGTTAAAAGATCTGGTAGGAGCGTGTTTTTTTGTAGGACCATTACTGGGATTAGTCTATTTTTATCCGTGGCTTCTGGGGGACCCCGAAAATTTTATTGAGGGCAACCCCTTGGTTACACCTCACCACATTCAACCAGAGTGGTACTTTTTGTTTGCTTACGCAATTTTGCGGGCTGTGCCTAACAAATTAGGGGGCGTGGTTGCCCTGGCTGCTGCAGTGTTGATTTTGTTTTATATGCCTTTTAGACCAAAAACATCAATCAAAGGTTTATCTTTTTATCCTTGAGGAAAGGTTTTATTTTTTAGGTTAATTGGGGTATTTATTTTACTTACCTGGGCGGGATCCAAACCGGTGGAAGAGCCTTATGTGGTGGTTAGGCAGGTGCTAAGGTTTTTGTATTTCTTCTATTTCTTACTAATTGGACCATTAACTCGCATTCAGGATAAGTTGTTGTAA

}

SampleName="BH 32"

SampleSize= 10

SampleData= {

CCR1_f2 8

BH_f11 2 ATTTATAAGGCTTCTCGATTTAGACCTCTCGCTAGAGTTATTCGCCACACCGTTATTACTTTACCTACGCCGGTTAATATTTCAACATGGTGAAACTTTGGCTCTCTATTAGGTGTTTGTCTGGCAACTCAGATTTTTAGGGGGCTGGTTTTAAGACTACATTTTACAGCTCATACGGATTTAAGGTTTTATGTAGTTATCCAAAGGTTTGGAGACGTTTACTATAATTGGTGATTTCGGGCTCTCCATGCTAATGGAGCTAGTTTCTTTTTTATCGGCCTTTATTTACATATTGCTCGAGGTATATATTATAGGTCTTATGTTTTCAAGGAAGTGTGATCTGTAGGTGTAGTCATCTTGCTTTTAGTAATAGCTAGAGCCTTTTTAGGCTATGTCCTCCCTTGAGGGCAGATATCCTTCTGAGGGGCAACAGTTATTACAAGGTTGTTTTCAGCCCTTCCCATCATTGGGAGAGATGTTGTGATTTGGCTATGAGGGGGGTTTTCGGTGGACAATGCGACACTTACTCGATTTTTTGGTATTCATTTTCTCTTGCCCTTTACAGTAGCCGGGCTAGCCGGGCTACACTTATTCTTCTTGCACGACCAAGGGTCGTCAAACCCTTTAGGGGTGAGATCCGATTTTAACAAAGTTGTTTTCCATCACTATTACGTGTTAAAAGATCTGGTAGGAGTGTGTTTTTTCGTAGGACCATTACTGGGACTAGTCTATTTTTATCCGTGGCTTCTGGGGGACCCCGAAAATTTTATTGAGGGCAACCCCTTGGTTACACCTCACCACATTCAACCAGAGTGGTACTTTTTGTTTGCTTACGCAATTTTGCGGGCTGTGCCTAACAAATTAGGGGGCGTGGTTGCCCTGGCTGCTGCAGTGTTGATTTTGTTTTATATGCCTTTTAGACCAAAAACATCAATCAAAGGTTTATCTTTTTATCCTTGAGGAAAGGTTTTATTTTTTAGGTTAATTGGGGTATTTATTTTACTTACCTGGGCGGGATCCAAACCGGTGGAAGAGCCTTATGTGGTGGTTAGGCAGGTGCTAAGGTTTTTGTATTTCTTCTATTTCTTACTAATTGGACCATTAACTCGCATTCAGGATAAGTTGTTGTAA

}

SampleName="SC2 33"

SampleSize= 5

SampleData= {

SC3_m8 5

}

SampleName="PES 34"

SampleSize= 2

SampleData= {

Pes_m9 2 ATTTATAAGGCTTCTCGATTTAGACCTCTCGCTAGAGTTATTCGCCACACCGTTATTTCTTTACCTACGCCGGTTAATATTTCAACATGGTGAAACTTTGGCTCTCTATTAGGTGTTTGTCTGGCAACTCAGATTTTTAGGGGGCTGGTTTTAAGACTACATTTTACGGCTCATACGGATTTAAGGTTTTATGTAGTTATCCAAAGGTTTGGAGATGTTTACTATAATTGGTGATTTCGGGCTCTCCATGCTAATGGAGCTAGTTTCTTTTTTATCGGCCTTTATTTACATATTGCTCGAGGTATATATTATGGGTCTTATGTTTTCAAGGAAGTGTGATCTGTAGGTGTAGTCATCTTGCTTTTAGTAATAGCTAGAGCCTTTTTAGGCTATGTCCTCCCTTGAGGGCAGATATCCTTCTGAGGGGCAACAGTTATTACAAGGTTGTTTTCAGCCCTTCCCATCATTGGGAGAGATGTTGTGATTTGGCTATGAGGGGGGTTTTCGGTGGACAATGCGACACTTACTCGATTTTTTGGTCTTCATTTTCTCTTGCCCTTTACAGTAGCCGGGCTAGCCGGGCTACACTTATTCTTCTTGCACGACCAAGGGTCGTCAAACCCTTTAGGGGTGAGATCTGATTTTAACAAAGTTGTTTTCCATCACTATTACGTATTAAAAGATCTGGTAGGAGTGTGTTTTTTCGTAGGACCATTACTGGGATTAGTCTATTTTTACCCGTGGCTTCTGGGGGACCCCGAAAATTTTATTGAGGGCAACCCCTTGGTTACACCTCACCACATTCAACCAGAGTGGTACTTTTTGTTTGCTTACGCAATTTTGCGGGCTGTGCCTAACAAGTTAGGGGGCGTGGTTGCCCTGGCTGCTGCAGTGTTGATTTTGTTTTATATGCCTTTTAGACCAAAAACATCAATCAAAGGTTTATCTTTTTATCCTTGAGGAAAGGTTTTATTTTTTAGGTTAATTGGGGTATTTATTTTACTTACCTGGGCGGGATCCAAACCGGTGGAAGAGCCCTATGTGGTGGTTAGGCAGGTGCTAAGGTTTTTGTATTTCTTCTATTTCTTACTAATTGGACAATTAACTCGCATTCAGGATAAGTTGTTGTAA

}

SampleName="CatIs 35"

SampleSize= 5

SampleData= {

Cat_7 1 ?????????????????????????????GGCTAGAGTAATTCGCCACACTGTAATTACATTGCCTACTCCGGTTAATATCTCAACCTGGTGAAACTTCGGCTCTCTCCTAGGAATTTGCTTAGCGATCCAAATTTTCAGGGGGTTAATTTTAAGGCTCCACTTTACAGCTCATACGGACCTAAGCTTTTATGTAGTTATCCAAAGGTTTGGGGACGTTTACTACAATTGGTGGTTTCGAGCTCTCCATGCTAATGGGGCTAGTTTCTTTTTTATTGGTTTATATTTACACATTGCTCGGGGTTTATACTACGGGTCCTATGTGTTTAAGGAAGTCTGGTCTGTGGGAGTAGTTATTCTTCTAGTGGTTATAGCCAGGGCTTTCTTGGGTTACGTTCTTCCCTGAGGGCAGATGTCCTTCTGAGGGGCAACGGTTATCACAAGGTTGTTTTCGGCACTCCCGGTTATTGGGGGAGATGTTGTAATTTGATTATGGGGAGGTTTTTCTGTAGACAATGCCACGCTAACTCGATTCTTTGGACTCCATTTCGTGTTGCCCTTCATGGTCGCAGGATTAGCTGGACTACACCTATTCTTCTTACACGACCAAGGGTCGTCAAATCCGTTAGGAGTGAGATCTGATTTTAACAAGGTTGTTTTTCATCATTACTATGTGCTTAAAGATTTGGTAGGGGCATGTTTTTTCTTAGGACCCCTATTAGGACTAGTTTATTTTAATCCATGACTTTTGGGGGACCCTGAGAATTTCATTGAGGGAAATCCTCTGGTGACTCC?CATCACATTCAGCCCGAGTGGTATTTTTTATTTGCTTATGCTATTTTGCGAGCTGTTCCCAATAAGCTTGGGGGGGTGGTGGCTTTGGCAGCTGCGG?ACTAGTTTTGTTTTATATGCCATTTAGTCCTAAAGCGTCAATTAAAGGGCTAAGGTTTTATCCTTGGGGAAAATCTTTATTTTTTAGGTTGATTGGGGTTTTTTTTTTACTCACATGGGCAGGGTCTAAGCCGGTAGAGGAACCCTATGTAGTAATTAGACAGGTTTTGAGGTTCTTGTACTTTTTGTATTTTGGATTAATTGGGCCCTTAACTCGCA?CC?????????????????

Cat_2 4 ?????????????????????????????GGCTAGAGTAATTCGCCACACTGTAATTACATTGCCTACTCCGGTTAATATCTCAACCTGGTGAAACTTCGGCTCTCTCCTAGGAATTTGCTTAGCGATCCAAATTTTCAGGGGGTTAATTTTAAGGCTCCACTTTACAGCTCATACGGACCTAAGCTTTTATGTAGTTATCCAAAGGTTTGGGGACGTTTACTACAATTGGTGGTTTCGAGCTCTCCATGCTAATGGGGCTAGTTTCTTTTTTATTGGTTTATATTTACACATTGCTCGGGGTTTATACTACGGGTCCTATGTGTTTAAGGAAGTCTGGTCTGTGGGAGTAGTTATTCTTCTAGTGGTTATAGCCAGGGCTTTCTTGGGTTACGTTCTTCCCTGAGGGCAGATGTCCTTCTGAGGGGCAACGGTTATCACAAGGTTGTTTTCGGCACTCCCGGTTATTGGGGGAGATGTTGTAATTTGATTATGGGGAGGTTTTTCTGTAGACAATGCCACGCTAACTCGATTCTTTGGACTCCATTTCGTGTTGCCCTTCATGGTCGCAGGATTAGCTGGACTACACCTATTCTTCTTACACGACCAAGGGTCGTCAAATCCGTTAGGAGTGAGATCTGATTTTAACAAGGTTGTTTTTCATCATTACTATG?GCTTAAA?ATTTGGTAGGGGCATGTTTTTT?TTAGGACC?CTATTAGGACTAGTTTATTTTAATCCATGACTTTTGGGGGACCCTGAGAATTTCATTGAGGGAAATCCTCTGGTGACTCCTCATCACATTCAGCCCGAG?GG?ATTTTTTATTTGCTTA?GCTATTTTGCGAGCTGTTCCCAATAAGCTTGGGGGGGTGGTGGCTTTGGCAGCTGCGG?ACTAGTTTTGTTTTATATGCCATTTAGTCCTAAAGCATCAATTAAAGGGCTAAGGTTTTATCCTTGGGGAAAATCTTTATTTTTTAGGTTGATTGGGGTTTTTTTTTTACTCACATGGGCAGGGTCTAAGCCGGTAGAGGAACCCTATGTAGTAATTAGACAGGTTTTGAGGTTCTTGTACTTTTTGTATTTTGGATTAATTGGGCCCTTAACTCGCATCCAAGATAAGTTGTTA???

}

SampleName="PTD 36"

SampleSize= 1

SampleData= {

Dume_m10 1 ??????AGGGG?ATCCTAAGGAAGCCTAAGGCTAGAGTAATTCGCCACACTGTAATTACATTACCCACTCCGGTTAATATCTCAACCTGGTGAAACTTCGGCTCTCTCCTAGGAATTTGCTTAGCGATCCAAATTTTCAGGGGGTTAATTTTAAGGCTCCACTTTACAGCTCACACGGACCTAAGCTTTTATGTAGTTATCCAAAGGTTTGGGGATGTTTACTACAATTGGTGGTTTCGAGCTCTCCATGCTAATGGGGCTAGTTTCTTTTTTATTGGTTTATATTTACACATCGCTCGGGGCTTATACTACGGGTCCTATGTGTTTAAGGAAGTCTGGTCTGTTGGAGTAGTTATTCTTCTAGTGGTTATAGCCAGGGCTTTCTTGGGTTACGTTCTTCCTTGAGGACAGATGTCCTTCTGAGGGGCAACGGTTATCACTAGATTGTTTTCGGCACTCCCGGTTATTGGGGGAGATGTTGTAATTTGATTATGGGGAGGTTTTTCTGTAGATAATGCCACACTAACTCGATTTTTTGGACTTCATTTCGTGTTACCCTTCATGGTCGCAGGATTAACTGGACTACACCTATTCTTCTTACACGACCAAGGGTCGTCAAATCCGTTAGGAGTGAGATCTGATTTTAACAAGGTTGTTTTTCATCATTACTATGTGCTTAAAGATTTGGTAGGGGCATGTTTTTTCTTAGGACCCCTATTAGGATTAGTTTATTTTAATCCATGACTTTTAGGGGACCCTGAGAATTTCATTGAGGGAAATCCTTTAGTGACTCCTCATCATATTCAGCCCGAGTGGTATTTTTTATTTGCTTATGCTATTTTGCGAGCTGTTCCTAATAAGCTTGGGGGGGTGGTAGCTTTGGCAGCTGCGGTACTAGTTTTGTTTTATATACCATTTAGTCCTAAAGCATCAATTAAAGGGCTAAGGTTTTATCCATGAGGAAAATCTTTATTTTTTAGGTTGATTGGGGTTTTTTTTTTACTCACATGGGCAGGGTCTAAACCGGTAGAGGAACCTTATGTAGTAATTAGGCAGGTTTTGAGGCTCTTGTACTTTTTGTATTTTGTATTAATTGGGCCCTTAACTCGCATCCTAAAA????????????

Dume_m9 1 ??????AGGGG?ATCCTAAGGAAGCCTAAGGCTAGAGTAATTCGCCACACTGTAATTACATTACCCACTCCGGTTAATATCTCAACCTGGTGAAACTTCGGCTCTCTCCTAGGAATTTGCTTAGCGATCCAAATTTTCAGGGGGTTAATTTTAAGGCTCCACTTTACAGCTCACACGGACCTAAGCTTTTATGTAGTTATCCAAAGGTTTGGGGATGTTTACTACAATTGGTGGTTTCGAGCTCTCCATGCTAATGGGGCTAGTTTCTTTTTTATTGGTTTATATTTACACATCGCTCGGGGCTTATACTACGGGTCCTATGTGTTTAAGGAAGTCTGGTCTGTTGGAGTAGTTATTCTTCTAGTGGTTATAGCCAGGGCTTTCTTGGGTTACGTTCTTCCTTGAGGACAGATGTCCTTCTGAGGGGCAACGGTTATCACTAGATTGTTTTCGGCACTCCCGGTTATTGGGGGAGATGTTGTAATTTGATTATGGGGAGGTTTTTCTGTAGATAATGCCACACTAACTCGATTTTTTGGACTTCATTTCGTGTTACCCTTCATGGTCGCAGGATTAACTGGACTACACCTATTCTTCTTACACGACCAAGGGTCGTCAAATCCGTTAGGAGTGAGATCTGATTTTAACAAGGTTGTTTTTCATCATTACTATGTGCTTAAAGATTTGGTAGGGGCATGTTTTTTCTTAGGACCCCTATTAGGATTAGTTTATTTTAATCCATGACTTTTAGGGGACCCTGAGAATTTCATTGAGGGAAATCCTTTAGTGACTCCTCATCATATTCAGCCCGAGTGGTATTTTTTATTTGCTTATGCTATTTTGCGAGCTGTTCCCAATAAGCTTGGGGGGGTGGTAGCTTTGGCAGCTGCGGTACTAGTTTTGTTTTATATACCATTTAGTCCTAAAGCATCAATTAAAGGGCTAAGGTTTTATCCATGAGGAAAATCTTTATTTTTTAGGTTGATTGGGGTTTTTTTTTTACTCACATGGGCAGGGTCTAAACCGGTAGAGGAACCTTATGTAGTAATTAGGCAGGTTTTGAGGCTCTTGTACTTTTTGTATTTTGTATTAATTGGGCCCTTAACTCGCATCCTAAAA????????????

}

SampleName="SS 37"

SampleSize= 6

SampleData= {

SS4 2 ????ATAAGGCTTCTCGATACAGGCCACTCATGAGGGTTATTCGCCATACTGTTGTCACCCTGCCAACACCAGTTAATATTTCAACATGGTGAAATTTTGGGTCTTTATTGGGCATTTGTTTGGCAATTCAGATTTTTAGGGGGTTAGTTCTAAGGCTCCATTTTACGGCCCATACGGATTTAAGATTTTACGTGGTTATCCAAAGGTTTGGGGATGTCTATTATAATTGGTGGTTTCGTGCTCTCCACGCCAATGGGGCTAGCTTCTTCTTTATTGGTCTTTACCTGCATATTGCTCGGGGGATGTATTATGGCTCCTATGTGTTTAAAGAAGTTTGGGCTGTTGGGGTGGTTATCTTATTACTCGTGATAGCTAGGGCTTTTTTAGGCTACGTGTTGCCCTGGGGACAAATGTCCTTTTGGGGGGCAACAGTTATTACAAGCTTATTTTCGGCACTTCCCATGATTGGAGGGGATGTAGTTATTTGGCTGTGAGGGGGGTTTTCTGTGGATAACGCCACTCTTACTCGCTTTTTTGGGCTACACTTTTTACTGCCTTTTGGTGTCGCAGGGCTAGCAGGGCTACACCTGTTTTTCTTACACGACCAAGGGTCGTCAAACCCTTTAGGGGTAAGATCTGATTTTAACAAAGTTGTTTTCCATCATTATTATGTGCTTAAGGATTTAGTCGGAGCATGCTTTTTTATTGGACCTTTACTTGCTTTGGTTTACTTTAACCCCTGACTTTTAGGAGATCCGGAAAATTTTATCGAGGGGAATCCCCTGGTTACACCTCAACATATTCAGCCGGAGTGGTACTTTCTATTTGCTTACGCTATTCTACGGGCTGTACCCAACAAATTAGGGGGGGTAGTCGCTTTAGCGGCCGCAGTGCTTATTTTATTCTACATACCTTTCAGGCCTAAGGCTTCTATTAAAGGATTATCTTTTTATCCTTGTGGGAAAATTCTTTTCTTTAGGTTAGTTGGGGTGTTTATCTTGCTTACTTGGGCAGGAGCTAAACCCGTAGAAGAGCCTTACGTAGCCCTTAGACAGGGGCTTAGGGTTTTATATTTCTTGTATTTCTTAGTGATTGGGCCGTTAACTCGGATCCAAGATAAGTTGTTGTAA

SS5 2 ??TTATAAGGCTTCTCGATACAGGCCACTCATGAGGGTTATTCGCCATACTGTTGTCACCCTGCCAACACCAGTTAATATTTCAACATGGTGAAATTTTGGGTCTTTATTGGGCATTTGTTTGGCAATTCAGATTTTTAGGGGGTTAGTTCTAAGGCTCCATTTTACGGCCCATACGGATTTAAGATTTTACGTGGTTATCCAAAGGTTTGGGGATGTCTATTATAATTGGTGGTTTCGTGCTCTCCACGCCAATGGGGCTAGCTTCTTCTTTATTGGTCTTTACCTGCATATTGCTCGGGGGATGTATTATGGTTCCTATGTGTTTAAAGAAGTTTGGGCTGTTGGGGTAGTTATCTTATTACTGGTGATAGCTAGGGCTTTTTTAGGCTACGTGTTGCCCTGGGGACAAATGTCCTTTTGGGGGGCAACAGTTATCACAAGCTTATTTTCGGCACTTCCCATGATTGGAGGGGATGTAGTTATTTGGCTGTGAGGGGGGTTTTCTGTGGATAACGCCACTCTTACTCGCTTTTTTGGGCTACACTTTTTACTGCCTTTTGGTGTCGCAGGGCTAGCAGGGCTACACCTGTTTTTCTTACACGACCAAGGGTCGTCAAACCCTTTAGGGGTAAGATCTGATTTTAACAAAGTTGTTTTCCATCATTATTATGTGCTTAAGGATTTAGTCGGAGCATGCTTTTTTATTGGACCCTTACTTGCTTTGGTTTACTTTAACCCCTGACTTTTAGGAGATCCGGAAAATTTTATCGAGGGGAATCCCCTGGTTACACCTCAACATATTCAGCCGGAGTGGTACTTTCTATTTGCTTACGCTATTCTACGGGCTGTGCCCAACAAATTAGGGGGGGTAGTCGCTTTAGCGGCTGCAGTGCTTATTTTATTCTACATACCTTTCAGGCCTAAGGCTTCTATTAAAGGATTATCTTTTTATCCTTGTGGGAAAATTCTTTTCTTTAGGTTAGTTGGGGTGTTTATCTTGCTTACTTGGGCAGGAGCTAAACCCGTAGAAGAGCCTTACGTAGCGATTAGACAGGGGCTTAGGGTTTTATATTTCTTGTATTTCTTAGTGATTGGGCCGTTAACTCGGATCCAAGATAAGTTGTTGTAA

SS3 1 ???TATAAGGCTTCTCGATACAGGCCACTCATGAGGGTTATTCGCCATACTGTTGTCACCCTGCCAACACCAGTTAATATTTCAACATGGTGAAATTTTGGGTCTTTATTGGGCATTTGTTTGGCAATTCAGATTTTTAGGGGGTTAGTTCTAAGGCTCCATTTTACGGCCCATACGGATTTAAGATTTTACGTGGTTATCCAAAGGTTTGGGGATGTCTATTATAATTGGTGGTTTCGTGCTCTCCACGCCAATGGGGCTAGCTTCTTCTTTATTGGTCTTTACCTGCATATTGCTCGGGGGATGTATTATGGCTCCTATGTGTTTAAAGAAGTTTGGGCTGTTGGGGTGGTTATCTTATTACTCGTGATAGCTAGGGCTTTTTTAGGCTACGTGTTGCCCTGGGGACAAATGTCCTTTTGGGGGGCAACAGTTATTACAAGCTTATTTTCGGCACTTCCCATGATTGGAGGGGATGTAGTTATTTGGCTGTGAGGGGGGTTTTCTGTGGATAACGCCACTCTTACTCGCTTTTTTGGGCTACACTTTTTACTGCCTTTTGGTGTCGCAGGGCTAGCAGGGCTACACCTGTTTTTCTTACACGACCAAGGGTCGTCAAACCCTTTAGGGGTAAGATCTGATTTTAACAAAGTTGTTTTCCATCATTATTATGTGCTTAAGGATTTAGTCGGAGCATGCTTTTTTATTGGACCCTTACTTGCTTTGGTTTACTTTAACCCCTGACTTTTAGGAGATCCGGAAAATTTTATCGAGGGGAATCCCCTGGTTACACCTCAACATATTCAGCCGGAGTGGTACTTTCTATTTGCTTACGCTATTCTACGGGCTGTGCCCAACAAATTAGGGGGGGTAGTCGCTTTAGCGGCCGCAGTGCTTATTTTATTCTACATACCTTTCAGGCCTAAGGCTTCTATTAAAGGATTATCTTTTTATCCTTGTGGGAAAATTCTTTTCTTTAGGTTAGTTGGGGTGTTTATCTTGCTTACTTGGGCAGGAGCTAAACCCGTAGAAGAGCCTTACGTAGCCATTAGACAGGGGCTTAGGGTTTTATATTTCTTGTATTTCTTAGTGATTGGGCCGTTAACTCGGATCCAAGATAAGTTGTTGTAA

SS1 1 ???????????????????ACAGGCCACTCATGAGGGTTATTCGCCATACTGTTGTCACCCTGCCAACACCAGTTAATATTTCAACATGGTGAAATTTTGGGTCTTTATTGGGCATTTGTTTGGCAATTCAGATTTTTAGGGGGTTAGTTCTAAGGCTCCATTTTACGGCCCATACGGATTTAAGATTTTACGTGGTTATCCAAAGGTTTGGGGATGTCTATTATAATTGGTGGTTTCGTGCTCTCCACGCCAATGGGGCTAGCTTCTTCTTTATTGGTCTTTACCTGCATATTGCTCGGGGGATGTATTATGGTTCCTATGTGTTTAAAGAAGTTTGGGCTGTTGGGGTAGTTATCTTATTACTGGTGATAGCTAGGGCTTTTTTAGGCTACGTGTTGCCCTGGGGACAAATGTCCTTTTGGGGGGCAACAGTTATTACAAGCTTATTTTCGGCACTTCCCATGATTGGAGGGGATGTAGTTATTTGGCTGTGAGGGGGGTTTTCTGTGGATAACGCCACTCTTACTCGCTTTTTTGGGCTACACTTTTTACTGCCTTTTGGTGTCGCAGGGCTAGCAGGGCTACACCTGTTTTTCTTACACGACCAAGGGTCGTCAAACCCTTTAGGGGTAAGATCTGATTTTAACAAAGTTGTTTTCCATCATTATTATGTGCTTAAGGATTTAGTCGGAGCATGCTTTTTTATTGGACCCTTACTTGCTTTGGTTTACTTTAACCCCTGACTTTTAGGAGATCCGGAAAATTTTATCGAGGGGAATCCCCTGGTTACACCTCAACATATTCAGCCGGAGTGGTACTTTCTATTTGCTTACGCTATTCTACGGGCTGTGCCCAACAAATTAGGGGGGGTAGTCGCTTTAGCGGCTGCAGTGCTTATTTTATTCTACATACCTTTCAGGCCTAAGGCTTCTATTAAAGGATTATCTTTTTATCCTTGTGGGAAAATTCTTTTCTTTAGGTTAGTTGGGGTGTTTATCTTGCTTACTTGGGCAGGAGCTAAACCCGTAGAAGAGCCTTACGTAGCGATTAGACAGGGGCTTAGGGTTTTATATTTCTTGTATTTCTTAGTGATTGGGCCGTTAACTCGGATCCAAGATAAGTT???????

}

SampleName="BC 38"

SampleSize= 6

SampleData= {

BC5 6 ??TTATAAGGCTTCCCGATTTAGGCCTCTCATGAGGGTGGTTCGTCACACTGTAATTACCCTACCTACCCCAGTTAATATTTCAACGTGGTGAAACTTTGGTTCACTCCTTGGGGTTTGTCTGGCGATTCAGATTTTTAGAGGGCTAGTTCTAAGGCTTCATTTCACGGCTCACACAGATTTAAGGTTTTATGTGGTTATTCAAAGGTTTGGAGATGTTTATTACAATTGGTGGTTTCGGGCTCTTCACGCCAATGGGGCTAGTTTCTTTTTTATCGGCCTCTACTTACACGTTGCCCGAGGGTTATATTACGGATCCTACGTTTTTAAGGAGGTTTGATCTGTCGGTGTAGTAATCTTGCTTTTAGTTATGGCCAGGGCTTTCTTGGGTTACGTTCTCCCTTGAGGCCAAATATCTTTTTGGGGGGCCACTGTAATTACAAGATTGTTTTCGGCACTCCCGATCATTGGGGGGGATGTGGTAATTTGATTATGAGGGGGGTTTTCCGTGGATAATGCGACTTTGACCCGTTTTTTCGGGCTTCATTTTCTTCTACCTTTTACAGTCGCCGGGTTAGCCGGCCTACACTTATTCTTCTTGCACGACCAAGGGTCGTCAAACCCCCTAGGGGTGAGATCAGATTATAATAAAGTGGTTTTTCATCACTACTATGTACTCAAAGATCTGGTCGGGGCATGTTTTTTTGTGGGACCTCTGCTGGGTTTAGTGTACTTTAATCCTTGGCTTCTAGGGGACCCTGAGAATTTTATTGAGGGTAATCCACTAGTGACTCCTCACCACATCCAGCCGGAATGGTATTTCTTATTTGCCTATGCGATTTTGCGGGCTGTGCCTAACAAATTAGGGGGGGTGGTGGCTCTGGCGGTTGCTGTACTAATTTTGTTGTATATGCCGTTTAGGCCCCAGACGGCGATTAAAGGTCTATCGTTTTATCCATGGGGAAAAGCGTTGTTTTTTAGGCTGATTGGAGTCTTTATTTTACTTACTTGGGCGGGGGCAAAACCGGTGGAAGAGCCTTATGTGGTCGTCAGTCAGGTGTTAAGGTTTTTATATTTTTCGTATTTTCTCTCAATTGGGCCACTGACTCGTATCCAGGACAAGTTGTTGTAA

}

SampleName="VI 39"

SampleSize= 5

SampleData= {

VI4 2 ???TATAAGGCTTCCCGATTTAGGCCTCTCATGAGGGTGGTTCGTCACACTGTAATTACCCTACCTACCCCAGTTAATATTTCAACATGGTGAAACTTTGGTTCACTCCTTGGGGTTTGTCTGGCAATTCAGATTTTTAGAGGGCTAGTTCTAAGGCTTCATTTCACGGCTCACACAGATTTAAGGTTTTATGTAGTTATTCAAAGGTTTGGAGATGTTTATTACAATTGGTGGTTTCGGGCTCTTCACGCCAATGGGGCTAGTTTCTTTTTTATCGGCCTCTACTTACACGTTGCCCGAGGGATATATTACGGATCCTACGTTTTTAAGGAGGTTTGATCTGTCGGTGTAGTAATCTTGCTTTTAGTTATGGCCAGGGCTTTCTTGGGTTACGTTCTCCCTTGAGGCCAAATATCTTTTTGGGGGGCCACTGTAATTACAAGATTGTTTTCGGCACTCCCGATCATTGGGGGGGATGTGGTAATTTGATTATGAGGGGGGTTTTCCGTGGATAATGCGACTTTGACCCGTTTTTTCGGGCTTCATTTTCTTCTACCTTTTACAGTCGCCGGATTAGCCGGCCTACACTTATTCTTCTTGCACGACCAAGGGTCGTCAAACCCCCTAGGGGTGAGATCAGATTATAATAAAGTGGTTTTTCATCACTACTATGTACTCAAAGATCTGGTCGGGGTATGTTTTTTCGTGGGACCTCTGTTGGGTTTAGTGTACTTTAATCCTTGGCTTCTGGGGGACCCTGAGAATTTTATTGAGGGTAACCCACTAGTGACTCCTCACCACATCCAGCCGGAATGGTATTTCTTATTTGCCTATGCGATTTTGCGGGCTGTGCCTAACAAATTAGGGGGGGTGGTCGCTCTGGCGGTTGCTGTACTAATTTTGTTGTACATGCCGTTTAGGCCCCAGACGGCGATCAAAGGTCTATCGTTTTACCCATGGGGAAAAGCGTTGTTTTTTAGGCTGGTTGGAGTCTTTATTTTACTTACTTGGGCGGGGGCAAAACCGGTGGAAGAGCCCTATGTGGTCGTCAGTCAGGTGTTAAGGTTTTTATATTTTTCGTATTTTCTCTTAATTGGGCCACTGACTCGTATCCAGGATAAGCTGTTGTAA

VI7 2 ????ATAAGGCTTCCCGATTTAGGCCTCTCATGAGGGTGGTTCGTCACACTGTAATTACCCTACCTACCCCAGTTAATATTTCAACATGGTGAAACTTTGGTTCACTCCTTGGGGTTTGTCTGGCAATTCAGATTTTTAGAGGGCTAGTTCTAAGGCTTCATTTCACGGCTCACACAGATTTAAGGTTTTATGTGGTTATTCAAAGGTTTGGAGATGTTTATTACAATTGGTGGTTTCGGGCTCTTCACGCCAATGGGGCTAGTTTCTTTTTTATCGGCCTCTACTTACACGTTGCCCGAGGGATATATTACGGATCCTACGTTTTTAAGGAGGTTTGATCTGTCGGTGTAGTAATCTTGCTTTTAGTTATGGCCAGGGCTTTCTTGGGTTACGTTCTCCCTTGAGGCCAAATATCTTTTTGGGGGGCCACTGTAATTACAAGATTGTTTTCGGCACTCCCGATCATTGGGGGGGATGTGGTAATTTGATTATGAGGGGGGTTTTCCGTGGATAATGCGACTTTGACCCGTTTTTTCGGGCTTCATTTTCTTCTACCTTTTACAGTCGCCGGATTAGCCGGCCTACACTTATTCTTCTTGCACGACCAAGGGTCGTCAAACCCCCTAGGGGTGAGATCAGATTATAATAAAGTGGTTTTTCATCACTACTATGTACTCAAAGATCTGGTCGGGGTATGTTTTTTTGTGGGACCTCTGTTGGGTTTAGTGTACTTTAATCCTTGGCTTCTGGGGGACCCTGAGAATTTTATTGAGGGTAACCCACTAGTGACTCCTCACCACATCCAGCCGGAATGGTATTTCTTATTTGCCTATGCGATTTTGCGGGCTGTGCCTAACAAATTAGGGGGGGTGGTCGCTCTGGCGGTTGCTGTACTAATTTTGTTGTACATGCCGTTTAGGCCCCAGACGGCGATCAAAGGTCTATCGTTTTACCCATGGGGAAAAGCGTTGTTTTTTAGGCTGATTGGAGTCTTTATTTTACTTACTTGGGCGGGGGCAAAACCGGTGGAAGAGCCCTATGTGGTCGTCAGTCAGGTGTTAAGGTTTATATATTTTTCGTATTTTCTCTTAATTGGGCCACTGACTCGTATCCAGGATAAGCTGTTGTAA

VI5 1 ???TATAAGGCTTCCCGATTTAGGCCTCTCATGAGGGTGGTTCGTCACACTGTAATTACCCTACCTACCCCAGTTAATATTTCAACATGGTGAAACTTTGGTTCACTCCTTGGGGTTTGTCTGGCAATTCAGATTTTTAGAGGGCTAGTTCTAAGGCTTCATTTCACGGCTCACACAGATTTAAGGTTTTATGTGGTTATTCAAAGGTTTGGAGATGTTTATTACAATTGGTGGTTTCGGGCTCTTCACGCCAATGGGGCTAGTTTCTTTTTTATCGGCCTCTACTTACACGTTGCCCGAGGGATATATTACGGATCCTACGTTTTTAAGGAGGTTTGATCTGTCGGTGTAGTAATCTTGCTTTTAGTTATGGCCAGGGCTTTCTTGGGTTACGTTCTCCCTTGAGGCCAAATATCTTTTTGGGGGGCCACTGTAATTACAAGATTGTTTTCGGCACTCCCGATCATTGGGGGGGATGTGGTAATTTGATTATGAGGGGGGTTTTCCGTGGATAATGCGACTTTGACCCGTTTTTTCGGGCTTCATTTTCTTCTACCTTTTACAGTCGCCGGATTAGCCGGCCTACACTTATTCTTCTTGCACGACCAAGGGTCGTCAAACCCCCTAGGGGTGAGATCAGATTATAATAAAGTGGTTTTTCATCACTACTATGTACTCAAAGATCTGGTCGGGGTATGTTTTTTTGTGGGACCTCTGTTGGGTTTAGTGTACTTTAATCCTTGGCTTCTGGGGGACCCTGAGAATTTTATTGAGGGTAACCCACTAGTGACTCCTCACCACATCCAGCCGGAATGGTATTTCTTATTTGCCTATGCGATTTTGCGGGCTGTGCCTAACAAATTAGGGGGGGTGGTCGCTCTGGCGGTTGCTGTACTAATTTTGTTGTACATGCCGTTTAGGCCCCAGACGGCGATCAAAGGTCTATCGTTTTACCCATGGGGAAAAGCGTTGTTTTTTAGGCTGATTGGAGTCTTTATTTTACTTACTTGGGCGGGGGCAAAACCGGTTGAAGAGCCCTATGTGGTCGTCAGTCAGGTGTTAAGGTTTTTATATTTTTCGTATTTTCTCTTAATTGGGCCACTGACTCGTATCCAGGATAAGCTGTTGTAA

}

SampleName="BSB 40"

SampleSize= 2

SampleData= {

BSB_f1 2 ?????????????????ATTTAGGCCCCTCATGAGAGTGGTTCGGCACAGTGTGGTTACCCTACCTACTCCGGTTAATATTTCAACGTGGTGAAACTTTGGTTCACTTCTTGGAGTTTGTCTGGCAATCCAGATTTTTAGGGGGCTGGTTTTAAGACTTCATTTTACAGCCCACACAGATTTAAGGTTTTATGTGGTTATCCAAAGGTTTGGAGATGTTTACTACAATTGGTGGTTTCGGGCTCTCCATGCTAACGGGGCTAGCTTCTTTTTTATCGGTCTCTATTTGCATATTGCCCGAGGAATATATTATGGATCCTACGTCTTTAAAGAGGTTTGATCGGTGGGTGTTGTCATTTTGCTTCTTGTTATAGCAAGGGCTTTCTTAGGTTATGTCCTTCCGTGAGGGCAAATATCTTTCTGGGGGGCAACTGTGATTACAAGCTTGTTTTCTGCACTTCCTGTAATTGGGAGGGATGTGGTAATCTGATTGTGAGGGGGGTTTTCGGTGGACAATGCGACTCTTACTCGATTTTTTGGGCTTCACTTTCTCTTGCCCTTTACAGTAGCCGGGCTAGCCGGGCTACATTTATTCTTCTTACACGACCAAGGGTCGTCAAATCCTCTAGGGGTGAGATCTGATTTCAACAAAGTTGTTTTCCATCACTATTATGTGCTCAAAGACCTGGTAGGGGCCTGTTTTTTTGTAGGACCTTTATTGGCTTTAGTATACTTTAATCCCTGGCTCCTGGGGGACCCTGAAAATTTCATTGAAGGCAATCCCTTAGTGACACCTCACCACATTCAACCGGAATGGTATTTTCTATTTGCTTACGCGATCTTGCGGGCTGTGCCTAACAAATTAGGGGGTGTGGTTGCCTTAGGGGTCGCAGTATTGATCTTGTTGTACATACCCTTCAGGCCTCGTACATCTATTAAAGGTTTGTCATTTTACCCATGAGGTAAGGTCATGTTTTTTAGGCTAATTGGAGTGTTTATTTTACTTACTTGGGCGGGGGCTAAACCCGTGGAAGAGCCTTATGTGGTAGTTAGACAGGTGTTAAGGTTTTTGTATTTCTCGTATTTTCTACTAATCGGACCTCTGACCCGTATCCAGGATAAGTTGTTGTAA

}

SampleName="LagBch 41"

SampleSize= 2

SampleData= {

LagBch_m1 2 ATTTATAAGGCTTCTCGATTTAGACCATTAACTAGGATTATTCGGCACACGGTAATCACTTTGCCCACTCCTATTAATATTTCAACGTGGTGAAATTTTGGTTCCCTTCTGGGAATTTGTTTGGCGAGCCAGATTTTGAGAGGATTGATTCTAAGGCTTCACTTCACTGCTCATACGGATCTCAGTTTTTATGTGGTGATTCAAAGATTTGGAGATGTTTACTATAATTGGTGGTTTCGTGCTCTCCACGCTAATGGGGCCAGTTTCTTTTTTATTGGACTTTATTTACATATTGCTCGGGGTCTCTATTATGGATCCTACGTTTTCAAGGAAGTTTGGTCCATTGGAGTTGTGATCTTACTTTTGGTAATAGCAAGTGCCTTCTTAGGCTACGTCCTTCCGTGGGGACAGATATCTTTTTGGGGGGCAACGGTTATTACTAGCCTATTTTCGGCACTCCCAGTTGTTGGGGGAGATGTGGTGGTTTGATTGTGAGGGGGCTTCTCGGTGGATAATGCTACCTTGACTCGGTTTTTTGGCCTTCATTTCGTGTTGCCCTTTATTGTGGCGGGGTTAGTTGGGCTTCACTTGTTCTTCTTACACGACCAAGGGTCGTCAAATCCTTTAGGGGTGAGATCAGATTTTAATAAGGTTGTTTTTCATCATTATTATGTGCTCAAGGATTTAGTGGGGGCTTGTTTTTTCATTGGTCCCTTACTTGGACTGGTTTACTTTAATCCTTGGCTTCTAGGAGATCCTGAAAATTTTATTGAGGGGAATCCCTTGGTTACACCACATCATATCCAACCTGAATGATATTTTTTATTTGCCTATGCCATTTTGCGGGCCGTACCTAACAAATTAGGAGGAGTCGTAGCGTTGGCTGCTGCGGTCTTGGTATTGTTTTATATACCAGTAAGACCTAAAACTTCTGTCAAAGGGTTATCTTTTTACCCGTGAGGAAAAATGTTGTTTTTTAGGTTGATTGGGGTGTTTTTTCTGCTTACATGGGCAGGGTCTAAGCCGGTAGAAGAACCATATGTGGTGATTAGACAGGTTTTAAGTTTCTTGTATTTTTCCTATTTTGCCTTAATTGGGCCTATCACTCGTATTCAAGACAAGTTGTTGTAA

}

[[Structure]]

StructureName="New Edited Structure"

NbGroups=4

Group={

"SD 1"

"SunCl 2"

"LS 3"

}

Group={

"LJS 4"

"Naut 5"

"BR 6"

"SIO 7"

"LJP 8"

"LJP2 9"

"LJP3 10"

"LJP04 11"

}

Group={

"RP1 12"

"RP2 13"

"IP 14"

"AB3 15"

"AB2 16"

"AB 17"

"ABR 18"

"PVL 19"

"RsPt 20"

"FR1 21"

"FR2 22"

}

Group={

"CCR1 23"

"CCR2 24"

"LH 25"

"NatBr1 26"

"NatBr2 27"

"SC3 28"

"SC4 29"

"SC5 30"

"SCN 31"

"BH 32"

"SC2 33"

}
