# Supplementary material for: Investigations of fine-scale phylogeography in Tigriopus californicus reveal historical patterns of population divergence
Source: BMC Evol Biol. 2009 Jun 23;9:139. doi: 10.1186/1471-2148-9-139 (PMC2708153; doi:10.1186/1471-2148-9-139)
Supplement: Additional file 4 — RISP sequences in Nexus format. This file contains the RISP sequences in nexus file format (interleaved sequences). [file 1471-2148-9-139-S4.doc]

#NEXUS

[RISP sequences in nexus file format (interleaved sequences). File generated by DnaSP Ver. 4.10.9]

begin data;

dimensions ntax=82 nchar=1892;

format datatype=dna gap=- interleave=yes MISSING=? ;

matrix

[ 10 20 30 40 ]

[ * * * * ]

'RP1_m1_A' ?????CCAAAACCGCCAATGTGCTCGGCCAAAAGGCTGTGCCAGGTAA

'RP1_m1_B' ?????CCAAAACCGCCAATGTGCTCGGCCAAAAGGCTGTGCCAGGTAA

'RP1_m5(A)' ?????CCAAAACCGCCAATGTGCTCGGCCAAAAGGCTGTGCCAGGTAA

'RP1_m5_B' ?????CCAAAACCGCCAATGTGCTCGGCCAAAAGGCTGTGCCAGGTAA

'RP1_m7_A' ATGGACCAAAACCGCCAATGTGCTCGGCCAAAAGGCTGTGCCAGGTAA

'RP1_m7_B' ATGGACCAAAACCGCCAATGTGCTCGGCCAAAAGGCTGTGCCAGGTAA

'RP1_m8_A' ATGGACCAAAACCGCCAATGTGCTCGGCCAAAAGGCTGTGCCAGGTAA

'RP1_m8_B' ATGGACCAAAACCGCCAATGTGCTCGGCCAAAAGGCTGTGCCAGGTAA

'R_Abg' ?????CCA?AACCGCCAATGTGCTCGGCCAAAAGGCTGTGCCAGGTAA

'R_Abg_2' ?????CCA?AACCGCCAATGTGCTCGGCCAAAAGGCTGTGCCAGGTAA

'AB_m1_A' ATGGACCAAAACCGCCAATGTGCTCGGCCAAAAGGCTGTGCCAGGTAA

'AB_m1_B' ATGGACCAAAACCGCCAATGTGCTCGGCCAAAAGGCTGTGCCAGGTAA

Abh ?????????????????ATGTG?TCGGCCAAAAGGCTGTGCCAGGTAA

'Abh_2' ?????????????????ATGTG?TCGGCCAAAAGGCTGTGCCAGGTAA

'AB_m2_A' ATGGACCAAAACCGCCAATGTGCTCGGCCAAAAGGCTGTGCCAGGTAA

'AB_m2_B' ATGGACCAAAACCGCCAATGTGCTCGGCCAAAAGGCTGTGCCAGGTAA

'IP_m8_A' ATGGACCAAAACCGCCAATGTGCTCGGCCAAAAGGCTGTGCCAGGTAA

'IP_m8_B' ATGGACCAAAACCGCCAATGTGCTCGGCCAAAAGGCTGTGCCAGGTAA

'IP_f3_A' ATGGACCAAAACCGCCAATGTGCTCGGCCAAAAGGCTGTGCCAGGTAA

'IP_f3_B' ATGGACCAAAACCGCCAATGTGCTCGGCCAAAAGGCTGTGCCAGGTAA

'IP_f23_A' ATGGACCAAAACCGCCAATGTGCTCGGCCAAAAGGCTGTGCCAGGTAA

'IP_f23_B' ATGGACCAAAACCGCCAATGTGCTCGGCCAAAAGGCTGTGCCAGGTAA

'IP_m7_A' ATGGACCAAAACCGCCAATGTGCTCGGCCAAAAGGCTGTGCCAGGTAA

'IP_m7_B' ATGG?CCAAAACCGCC?ATGTGCTCGGCCAAAAGGCTGTGCCAGGTAA

'PVL_f14_A' ATGGACCAAAACCGCCAATGTGCTCGGCCAAAAGGCTGTGCCAGGTAA

'PVL_f14_B' ATGGACCAAAACCGCCAATGTGCTCGGCCAAAAGGCTGTGCCAGGTAA

'PVL_f12_A' ATGGACCAAAACCGCCAATGTGCTCGGCCAAAAGGCTGTGCCAGGTAA

'PVL_f12_B' ATGGACCAAAACCGCCAATGTGCTCGGCCAAAAGGCTGTGCCAGGTAA

'PVL_f3_A' ATGGACCAAAACCGCCAATGTGCTCGGCCAAAAGGCTGTGCCAGGTAA

'PVL_f3_B' ATGGACCAAAACCGCCAATGTGCTCGGCCAAAAGGCTGTGCCAGGTAA

'PVL_f7_A' ATGGACCAAAACCGCCAATGTGCTCGGCCAAAAGGCTGTGCCAGGTAA

'PVL_f7_B' ATGGACCAAAACCGCCAATGTGCTCGGCCAAAAGGCTGTGCCAGGTAA

'Pes_m9_A' ATGGACCAAAACCGCCAATGTGCTCGGCCAAAAGGCTGTGCCAGGTAA

'Pes_m9_B' ATGGACCAAAACCGCCAATGTGCTCGGCCAAAAGGCTGTGCCAGGTAA

'Pes_m10_A' ATGGACCAAAACCGCCAATGTGCTCGGCCAAAAGGCTGTGCCAGGTAA

'Pes_m10_B' ATGGACCAAAACCGCCAATGTGCTCGGCCAAAAGGCTGTGCCAGGTAA

'LH_m1_A' ATGGACCAAAACCGCCAATGTGCTCGGCCAAAAGGCTGTGCCAGGTAA

'LH_m1_B' ATGGACCAAAACCGCCAATGTGCTCGGCCAAAAGGCTGTGCCAGGTAA

'LH_f6_A' ATGGACCAAAACCGCCAATGTGCTCGGCCAAAAGGCTGTGCCAGGTAA

'LH_f6_B' ATGGACCAAAACCGCCAATGTGCTCGGCCAAAAGGCTGTGCCAGGTAA

'LH_f8_A' ATGGACCAAAACCGCCAATGTGCTCGGCCAAAAGGCTGTGCCAGGTAA

'LH_f8_B' ATGGACCAAAACCGCCAATGTGCTCGGCCAAAAGGCTGTGCCAGGTAA

'scn_h_a' ?????????????????ATGTGCTCGGCCAAAAGGCTGTGCCAGGTAA

'scn_h_b' ?????????????????ATGTGCTCGGCCAAAAGGCTGTGCCAGGTAA

'scn_m5_A' ATGGACCAAAACCGCCAATGTGCTCGGCCAAAAGGCTGTGCCAGGTAA

'scn_m5_B' ATGGACCAAAACCGCCAATGTGCTCGGCCAAAAGGCTGTGCCAGGTAA

'scn_m7_A' ATGGACCAAAACCGCCAATGTGCTCGGCCAAAAGGCTGTGCCAGGTAA

'scn_m7_B' ATGGACCAAAACCGCCAATGTGCTCGGCCAAAAGGCTGTGCCAGGTAA

'scn_m3_A' ATGGACCAAAACCGCCAATGTGCTCGGCCAAAAGGCTGTGCCAGGTAA

'scn_m3_B' ATGGACCAAAACCGCCAATGTGCTCGGCCAAAAGGCTGTGCCAGGTAA

'LJS_m1_A' ATGGACCAAAACCGCCAATGTGCTCGGCCAAAAGGCTGTGCCAGGTAA

'LJS_m1_B' ATGGACCAAAACCGCCAATGTGCTCGGCCAAAAGGCTGTGCCAGGTAA

'LJS_m2_A' ATGGACCAAAACCGCCAATGTGCTCGGCCAAAAGGCTGTGCCAGGTAA

'LJS_m2_B' ATGGACCAAAACCGCCAATGTGCTCGGCCAAAAGGCTGTGCCAGGTAA

'LJS_f10_A' ATGGACCAAAACCGCCAATGTGCTCGGCCAAAAGGCTGTGCCAGGTAA

'LJS_f10_B' ATGGACCAAAACCGCCAATGTGCTCGGCCAAAAGGCTGTGCCAGGTAA

'LJS_f9_B' ATGGACCAAAACCGCCAATGTGCTCGGCCAAAAGGCTGTGCCAGGTAA

'LJS_f9_A' ATGGACCAAAACCGCCAATGTGCTCGGCCAAAAGGCTGTGCCAGGTAA

'LJP_m7_B' ATGGACCAAAACCGCCAATGTGCTCGGCCAAAAGGCTGTGCCAGGTAA

'LJP_m7_A' ATGGACCAAAACCGCCAATGTGCTCGGCCAAAAGGCTGTGCCAGGTAA

'LJP_m6_B' ATGGACCAAAACCGCCAATGTGCTCGGCCAAAAGGCTGTGCCAGGTAA

'LJP_m6_A' ATGGACCAAAACCGCCAATGTGCTCGGCCAAAAGGCTGTGCCAGGTAA

'LJP_m2_B' ATGGACCAAAACCGCCAATGTGCTCGGCCAAAAGGCTGTGCCAGGTAA

'LJP_m2_A' ATGGACCAAAACCGCCAATGTGCTCGGCCAAAAGGCTGTGCCAGGTAA

'LJP_m1_B' ATGGACCAAAACCGCCAATGTGCTCGGCCAAAAGGCTGTGCCAGGTAA

'LJP_m1_A' ATGGACCAAAACCGCCAATGTGCTCGGCCAAAAGGCTGTGCCAGGTAA

'BR_m5_A' ATGGACCAAAACCGCCAATGTGCTCGGCCAAAAGGCTGTGCCAGGTAA

'BR_m5_B' ATGGACCAAAACCGCCAATGTGCTCGGCCAAAAGGCTGTGCCAGGTAA

'BR_m6_A' ATGGACCAAAACCGCCAATGTGCTCGGCCAAAAGGCTGTGCCAGGTAA

'BR_m6_B' ATGGACCAAAACCGCCAATGTGCTCGGCCAAAAGGCTGTGCCAGGTAA

'BR_m7_A' ATGGACCAAAACCGCCAATGTGCTCGGCCAAAAGGCTGTGCCAGGTAA

'BR_m7_B' ATGGACC??AACCGCCAATGTGCTCGGCCAAAAGGCTG?GCCAGGTAA

'BR_f13_B' ATGGACCAAAACCGCCAATGTGCTCGGCCAAAAGGCTGTGCCAGGTAA

'BR_f13_A' ATGGACCAAAACCGCCAATGTGCTCGGCCAAAAGGCTGTGCCAGGTAA

'BR_m8_B' ATGGACCAAAACCGCCAATGTGCTCGGCCAAAAGGCTGTGCCAGGTAA

'BR_m8_A' ATGGACCAAAACCGCCAATGTGCTCGGCCAAAAGGCTGTGCCAGGTAA

'SD_h_a' ?????????????????ATGTGCTCGGCCAAAAGGCTGTGCCAGGTAA

'SD_h_b' ?????????????????ATGTGCTCGGCCAAAAGGCTGTGCCAGGTAA

'SD_g_a' ATGGACCAAAACCGCCAATGTGCTCGGCCAAAAGGCTGTGCCAGGTAA

'SD_g_b' ATGGACCAAAACCGCCAATGTGCTCGGCCAAAAGGCTGTGCCAGGTAA

'SD_2m_b' ??GGACCAAAACCGCCAATGTGCTCGGCCAAAAGGCTGTGCCAGGTAA

'SD_2m_a' ??GGACCAAAACCGCCAATGTGCTCGGCCAAAAGGCTGTGCCAGGTAA

[ 50 60 70 80 90 ]

[ * * * * * ]

'RP1_m1_A' GTGACCCCTGTGTGTCAATCTCAGGATCCATGGGAGCCCGTCCTCCAT

'RP1_m1_B' GTGACCCCTGTGTGTCAATCTCACGATCCATGGGAGCCCGTCCTCCAT

'RP1_m5(A)' GTGACCCCTGTGTGTCAATCTCAGGATCCATGGGAGCCCGTCCTCCAT

'RP1_m5_B' GTGACCCCTGTGTGTCAATCTGAGGATCCATGGGAGCCCGTCCTCCAT

'RP1_m7_A' GTGACCCCTGTGTGTCAATCTCAGGATCCATGGGAGCCCGTCCTCCAT

'RP1_m7_B' GTGACCCCTGTGTGTCAATCTCAGGATCCATGGGAGCCCGTCCTCCAT

'RP1_m8_A' GTGACCCCTGTGTGTCAATCTCAGGATCCATGGGAGCCCGTCCTCCAT

'RP1_m8_B' GTGACCCCTGTGTGTCAATCTCAGGATCCATGGGAGCCCGTCCTCCAT

'R_Abg' GTGACTCCTGTGTGTCAATCTCAGGATCCATGGGAGCCCGTCCTCCGT

'R_Abg_2' GTGACTCCTGTGTGTCAATCTCAGGATCCATGGGAGCCCGTCCTCCGT

'AB_m1_A' GTGATTCCTGTGTGTCAATCTCAGGATCCATGGGAGCCCGTCCTCCGT

'AB_m1_B' GTGACTCCTGTGTGTCAATCTCAGGATCCATGGGAGCCCGTCCTCCGT

Abh GTGACTCCTGTGTGTCAATCTCAGGATCCATGGGAGCCCGTCCTCCGT

'Abh_2' GTGACTCCTGTGTGTCAATCTCAGGATCCATGGGAGCCCGTCCTCCGT

'AB_m2_A' GTGACTCCTGTGTGTCAATCTCAGGATCCATGGGAGCCCGTCCTCCGT

'AB_m2_B' GTGACTCCTGTGTGTCAATCTCAGGATCCATGGGAGCCCGTCCTCCGT

'IP_m8_A' GTGACCCCTGTGTGTCAATCTCAGGATCCATGGGAGCCCGTCCTCCAT

'IP_m8_B' GTGACCCCTGTGTGTCAATCTCAGGATCCATGGGAGCCCGTCCTCCAT

'IP_f3_A' GTGACTCCTGTGTGTCAATCTCAGGATCCATGGGAGCCCGTCCTCCGT

'IP_f3_B' GTGACCCCTGTGTGTCAATCTCAGGATCCATGGGAGCCCGTCCTCCAT

'IP_f23_A' GTGACCCCTGTGTGTCAATCTCAGGATCCATGGGAGCCCGTCCTCCAT

'IP_f23_B' GTGACTCCTGTGTGTCAATCTCAGGATCCATGGGAGCCCGTCCTCCGT

'IP_m7_A' GTGACCCCTGTGTGTCAATCTCAGGATCCATGGGAGCCCGTCCTCCAT

'IP_m7_B' GTGACCCCTGTGTGTCAATCTCAGGATCCATGGGAGCCCGTCCTCCAT

'PVL_f14_A' GTGACTCCTGTGTGTCAATCTCAGGATCCATGGGAGCCCGTCCTCCGT

'PVL_f14_B' GTGACTCCTGTGTGTCAATCTCAGGATCCATGGGAGCCCGTCCTCCGT

'PVL_f12_A' GTGACCCCTGTGTGTCAATCTCAGGATCCATGGGAGCCCGTCCTCCAT

'PVL_f12_B' GTGACCCCTGTGTGTCAATCTCAGGATCCATGGGAGCCCGTCCTCCAT

'PVL_f3_A' GTGACTCCTGTGTGTCAATCTCAGGATCCATGGGAGCCCGTCCTCCGT

'PVL_f3_B' GTGACTCCTGTGTGTCAATCTCAGGATCCATGGGAGCCCGTCCTCCGT

'PVL_f7_A' GTGACTCCTGTGTGTCAATCTCAGGATCCATGGGAGCCCGTCCTCCGT

'PVL_f7_B' GTGACTCCTGTGTGTCAATCTCAGGATCCATGGGAGCCCGTCCTCCGT

'Pes_m9_A' GTGACCCCTGTGTGTCTATCTCAGGACCCATGGGAGCCCGTCCTCCAT

'Pes_m9_B' GTGACCCCTGTGTGTCTATCTCAGGACCCATGGGAGCCCGTCCTCCAT

'Pes_m10_A' GTGACCCCTGTGTGTCTATCTCAGGACCCATGGGAGCCCGTCCTCCAT

'Pes_m10_B' GTGACCCCTGTGTGTCTATCTCAGGACCCATGGGAGCCCGTCCTCCAT

'LH_m1_A' GTGACCCCTGTGTGTCTATCTCAGGACCCATGGGAGCCCGTCCTCCAT

'LH_m1_B' GTGACCCCTGTGTGTCTATCTCAGGACCCATGGGAGCCCGTCCTCCAT

'LH_f6_A' GTGACCCCTGTGTGTCTATCTCAGGACCCATGGGAGCCCGTCCTCCAT

'LH_f6_B' GTGACCCCTGTGTGTCTATCTCAGGACCCATGGGAGCCCGTCCTCCAT

'LH_f8_A' GTGACCCCTGTGTGTCTATCTCAGGACCCATGGGAGCCCGTCCTCCAT

'LH_f8_B' GTGACCCCTGTGTGTCTATCTCAGGACCCATGGGAGCCCGTCCTCCAT

'scn_h_a' GTGACCCCTGTGTGTCTATCTCAGGACCCATGGGAGCCCGTCCTCCAT

'scn_h_b' GTGACCCCTGTGTGTCTATCTCAGGACCCATGGGAGCCCGTCCTCCAT

'scn_m5_A' GTGACCCCTGTGTGTCTATCTCAGGACCCATGGGAGCCCGTCCTCCAT

'scn_m5_B' GTGACCCCTGTGTGTCTATCTCAGGACCCATGGGAGCCCGTCCTCCAT

'scn_m7_A' GTGACCCCTGTGTGTCTATCTCAGGACCCATGGGAGCCCGTCCTCCAT

'scn_m7_B' GTGACCCCTGTGTGTCTATCTCAGGACCCATGGGAGCCCGTCCTCCAT

'scn_m3_A' GTGACCCCTGTGTGTCTATCTCAGGACCCATGGGAGCCCGTCCTCCAT

'scn_m3_B' GTGACCCCTGTGTGTCTATCTCAGGACCCATGGGAGCCCGTCCTCCAT

'LJS_m1_A' GTGACCCCTGTGTGTCAATCCCAGGACCCATGGGAGCTCGTCCTCCAT

'LJS_m1_B' GTGACCCCTGTGTGTCAATCCCAGGACCCATGGGAGCTCGTCCTCCAT

'LJS_m2_A' GTGACCCCTGTGTGTCAATCCCAGGACCCATGGGAGCTCGTCCTCCAT

'LJS_m2_B' GTGACCCCTGTGTGTCAATCCCAGGACCCATGGGAGCTCGTCCTCCGT

'LJS_f10_A' GTGACCCCTGTGTGTCAATCCCAGGACCCATGGGAGCTCGTCCTCCAT

'LJS_f10_B' GTGACCCCTGTGTGTCAATCCCAGGACCCATGGGAGCTCGTCCTCCAT

'LJS_f9_B' GTGACCCCTGTGTGTCAATCCCAGGACCCATGGGAGCTCGTCCTCCAT

'LJS_f9_A' GTGACCCCTGTGTGTCAATCCCAGGACCCATGGGAGCTCGTCCTCCAT

'LJP_m7_B' GTGACCCCTGTGTGTCAATCCCAGGACCCATGGGAGCTCGTCCTCCAT

'LJP_m7_A' GTGACCCCTGTGTGTCAATCCCAGGACCCATGGGAGCTCGTCCTCCAT

'LJP_m6_B' GTGACCCCTGTGTGTCAATCCCAGGACCCATGGGAGCTCGTCCTCCAT

'LJP_m6_A' GTGACCCCTGTGTGTCAATCCCAGGACCCATGGGAGCTCGTCCTCCGT

'LJP_m2_B' GTGACCCCTGTGTGTCAATCCCAGGACCCATGGGAGCTCGTCCTCCAT

'LJP_m2_A' GTGACCCCTGTGTGTCAATCCCAGGACCCATGGGAGCTCGTCCTCCAT

'LJP_m1_B' GTGACCCCTGTGTGTCAATCCCAGGACCCATGGGAGCTCGTCCTCCAT

'LJP_m1_A' GTGACCCCTGTGTGTCAATCCCAGGACCCATGGGAGCTCGTCCTCCAT

'BR_m5_A' GTGACCCCTGTGTGTCAATCCCAGGACCCATGGGAGCTCGTCCTCCAT

'BR_m5_B' GTGACCCCTGTGTGTCAATCCCAGGACCCATGGGAGCTCGTCCTCCAT

'BR_m6_A' GTGACCCCTGTGTGTCAATCCCAGGACCCATGGGAGCTCGTCCTCCAT

'BR_m6_B' GTGACCCCTGTGTGTCAATCCCAGGACCCATGGGAGCTCGTCCTCCAT

'BR_m7_A' GTGACCCCTGTGTGTCAATCCCAGGACCCATGGGAGCTCGTCCTCCAT

'BR_m7_B' GTGACCCCTGTGTGTCAATCCCAGGACCCATGGGAGCTCGTCCTCCAT

'BR_f13_B' GTGACCCCTGTGTGTCAATCCCAGGACCCATGGGAGCTCGTCCTCCAT

'BR_f13_A' GTGACCCCTGTGTGTCAATCCCAGGACCCATGGGAGCTCGTCCTCCAT

'BR_m8_B' GTGACCCCTGTGTGTCAATCCCAGGACCCATGGGAGCTCGTCCTCCAT

'BR_m8_A' GTGACCCCTGTGTGTCAATCCCAGGACCCATGGGAGCTCGTCCTCCAT

'SD_h_a' GTGACCCCTGTGTGTCAATCCCAGGACCCATGGGAGCTCGTCCTCCAT

'SD_h_b' GTGACCCCTGTGTGTCAATCCCAGGACCCATGGGAGCTCGTCCTCCAT

'SD_g_a' GTGACCCCTGTGTGTCAATCCCAGGACCCATGGGAGCTCGTCCTCCAT

'SD_g_b' GTGACCCCTGTGTGTCAATCCCAGGACCCATGGGAGCTCGTCCTCCAT

'SD_2m_b' GTGACCCCTGTGTGTCAATCCCAGGACCCATGGGAGCTCGTCCTCCAT

'SD_2m_a' GTGACCCCTGTGTGTCAATCCCAGGACCCATGGGAGCTCGTCCTCCAT

[ 100 110 120 130 140 ]

[ * * * * * ]

'RP1_m1_A' GTATGCAAACGATGACAGATCCAAGGCAAATACTAACAATTGATTATA

'RP1_m1_B' GTATGCAAACGATGACAGATCCAAGGCAAATACTAACAATTGATTATA

'RP1_m5(A)' GTATGCAAACGATGACAGATCCAAGGCAAATACTAACAATTGATTATA

'RP1_m5_B' GTATGCAAACGATGACAGATCCAAGGCAAATACTAACAATTGATTATA

'RP1_m7_A' GTATGCAAACGATGACAGATCCAAGGCAAATACTAACAATTGATTATA

'RP1_m7_B' GTATGCAAACGATGACAGATCCAAGGCAAATACTAACAATTGATTATA

'RP1_m8_A' GTATGCAAACGATGACAGATCCAAGGCAAATACTAACAATTGATTATA

'RP1_m8_B' GTATGCAAACGATGACAGATCCAAGGCAAATACTAACAATTGATTATA

'R_Abg' GTATGCAAACGATGACAGATCCAAGGCAAATACTAACAATTGATTATA

'R_Abg_2' GTATGCAAACGATGACAGATCCAAGGCAAATACTAACAATTGATTATA

'AB_m1_A' GTATGCAAACGATGACAGATCCAAGGCAAATACTAACAATTGATTATA

'AB_m1_B' GTATGCAAACGATGACAGATCCAAGGCAAATACTAACAATTGATTATA

Abh GTATGCAAACGATGACAGATCCAAGGCAAATACTAACAATTGATTATA

'Abh_2' GTATGCAAACGATGACAGATCCAAGGCAAATACTAACAATTGATTATA

'AB_m2_A' GTATGCAAACGATGACAGATCCAAGGCAAATACTAACAATTGATTATA

'AB_m2_B' GTATGCAAACGATGACAGATCCAAGGCAAATACTAACAATTGATTATA

'IP_m8_A' GTATGCAAACGATGACAGATCCAAGGCAAATACTAACAATTGATTATA

'IP_m8_B' GTATGCAAACGATGACAGATCCAAGGCAAATACTAACAATTGATTATA

'IP_f3_A' GTATGCAAACGATGACAGATCCAAGGCAAATACTAACAATTGATTATA

'IP_f3_B' GTATGCAAACGATGACAGATCCAAGGCAAATACTAACAATTGATTATA

'IP_f23_A' GTATGCAAACGATGACAGATCCAAGGCAAATACTAACAATTGATTATA

'IP_f23_B' GTATGCAAACGATGACAGATCCAAGGCAAATACTAACAATTGATTATA

'IP_m7_A' GTATGCAAACGATGACAGATCCAAGGCAAATACTAACAATTGATTATA

'IP_m7_B' GTATGCAAACGATGACAGATCCAAGGCAAATACTAACAATTGATTATA

'PVL_f14_A' GTATGCAAACGATGACAGATCCAAGGCAAATACTAACAATTGATTATA

'PVL_f14_B' GTATGCAAACGATGACAGATCCAAGGCAAATACTAACAATTGATTATA

'PVL_f12_A' GTATGCAAACGATGACAGATCCAAGGCAAATACTAACAATTGATTATA

'PVL_f12_B' GTATGCAAACGATGACAGATCCAAGGCAAATACTAACAATTGATTATA

'PVL_f3_A' GTATGCAAACGATGACAGATCCAAGGCAAATACTAACAATTGATTATA

'PVL_f3_B' GTATGCAAACGATGACAGATCCAAGGCAAATACTAACAATTGATTATA

'PVL_f7_A' GTATGCAAACGATGACAGATCCAAGGCAAATACTAACAATTGATTATA

'PVL_f7_B' GTATGCAAACGATGACAGATCCAAGGCAAATACTAACAATTGATTATA

'Pes_m9_A' GTATGCAAACGATGGCAGATCCAAGGCAAATACTAACAATTGATTATA

'Pes_m9_B' GTATGCAAACGATGGCAGATCCAAGGCAAATACTAACAATGGATTATA

'Pes_m10_A' GTATGCAAACGATGGCAGATCCAAGGCAAATACTAACAATTGATTATA

'Pes_m10_B' GTATGCAAACGATGGCAGATCCAAGGCAAATACTAACAATTGATTATA

'LH_m1_A' GTATGCAAACGATGGCAGATCCAAGGCAAATACTAACAATGGATTATA

'LH_m1_B' GTATGCAAACGATGGCAGATCCAAGGCAAATACTAACAATGGATTATA

'LH_f6_A' GTATGCAAACGATGGCAGATCCAAGGCAAATACTAACAATGGATTATA

'LH_f6_B' GTATGCAAACGATGGCAGATCCAAGGCAAATACTAACAATGGATTATA

'LH_f8_A' GTATGCAAACGATGGCAGATCCAAGGCAAATACTAACAATGGATTATA

'LH_f8_B' GTATGCAAACGATGGCAGATCCAAGGCAAATACTAACAATGGATTATA

'scn_h_a' GTATGCAAACGATGGCAGATCCAAGGCAAATACTAACAATGGATTATA

'scn_h_b' GTATGCAAACGATGGCAGATCCAAGGCAAATACTAACAATGGATTATA

'scn_m5_A' GTATGCAAACGATGGCAGATCCAAGGCAAATACTAACAATGGATTATA

'scn_m5_B' GTATGCAAACGATGGCAGATCCAAGGCAAATACTAACAATGGATTATA

'scn_m7_A' GTATGCAAACGATGGCAGATCCAAGGCAAATACTAACAATGGATTATA

'scn_m7_B' GTATGCAAACGATGGCAGATCCAAGGCAAATACTAACAATGGATTATA

'scn_m3_A' GTATGCAAACGATGGCAGATCCAAGGCAAATACTAACAATGGATTATA

'scn_m3_B' GTATGCAAACGATGGCAGATCCAAGGCAAATACTAACAATGGATTATA

'LJS_m1_A' GAATGCAAACCATGGCAGATCCGAGGCAAATACTAACAATTGATCATA

'LJS_m1_B' GAATGCAAACCATGGCAGATCCGAGGCAAATACTAACAATTGATCATA

'LJS_m2_A' GAATGCAAACCATGGCAGATCCGAGGCAAATACTAACAATTGATCATA

'LJS_m2_B' GAATGCAAACCATGGCAGATCCGAGGCAAATACTAACAATTGATCATA

'LJS_f10_A' GAATGCAAACCATGGCAGATCCGAGGCAAATACTAACAATTGATCATA

'LJS_f10_B' GAATGCAAACCATGGCAGATCCGAGGCAAATACTAACAATTGATCATA

'LJS_f9_B' GAATGCAAACCATGGCAGATCCGAGGCAAATACTAACAATTGATCATA

'LJS_f9_A' GAATGCAAACCATGGCAGATCCGAGGCAAATACTAACAATTGATCATA

'LJP_m7_B' GAATGCAAACCATGGCAGATCCGAGGCAAATACTAACAATTGATCATA

'LJP_m7_A' GAATGCAAACCATGGCAGATCCGAGGCAAATACTAACAATTGATCATA

'LJP_m6_B' GAATGCAAACCATGGCAGATCCGAGGCAAATACTAACAATTGATCATA

'LJP_m6_A' GAATGCAAACCATGGCAGATCCAAGGCAAATACTAACAATTGATCATA

'LJP_m2_B' GAATGCAAACCATGGCAGATCCGAGGCAAATACTAACAATTGATCATA

'LJP_m2_A' GAATGCAAACCATGGCAGATCCGAGGCAAATACTAACAATTGATCATA

'LJP_m1_B' GAATGCAAACCATGGCAGATCCGAGGCAAATACTAACAATTGATCATA

'LJP_m1_A' GAATGCAAACCATGGCAGATCCGAGGCAAATACTAACAATTGATCATA

'BR_m5_A' GAATGCAAACCATGGCAGATCCGAGGCAAATACTAACAATTGATCATA

'BR_m5_B' GAATGCAAACCATGGCAGATCCGAGGCAAATACTAACAATTGATCATA

'BR_m6_A' GAATGCAAACCATGGCAGATCCGAGGCAAATACTAACAATTGATCATA

'BR_m6_B' GAATGCAAACCATGGCAGATCCGAGGCAAATACTAACAATTGATCATA

'BR_m7_A' GAATGCAAACCATGGCAGATCCGAGGCAAATACTAACAATTGATCATA

'BR_m7_B' GAATGCAAACCATGGCAGATCCGAGGCAAATACTAACAATTGATCATA

'BR_f13_B' GAATGCAAACCATGGCAGATCCGAGGCAAATACTAACAATTGATCATA

'BR_f13_A' GAATGCAAACCATGGCAGATCCGAGGCAAATACTAACAATTGATCATA

'BR_m8_B' GAATGCAAACCATGGCAGATCCGAGGCAAATACTAACAATTGATCATA

'BR_m8_A' GAATGCAAACCATGGCAGATCCGAGGCAAATACTAACAATTGATCATA

'SD_h_a' GAATGCAAACCATGGCAGATCCAAGGCAAATACTAACAATTGATCATA

'SD_h_b' GAATGCAAACCATGGCAGATCCAAGGCAAATACTAACAATTGATCATA

'SD_g_a' GAATGCAAACCATGGCAGATCCAAGGCAAATACTAACAATTGATCATA

'SD_g_b' GAATGCAAACCATGGCAGATCCAAGGCAAATACTAACAATTGATCATA

'SD_2m_b' GAATGCAAACCATGGCAGATCCAAGGCAAATACTAACAATTGATCATA

'SD_2m_a' GAATGCAAACCATGGCAGATCCAAGGCAAATACTAACAATTGATCATA

[ 150 160 170 180 190 ]

[ * * * * * ]

'RP1_m1_A' CAGTCTTTAAAATCAAATTAA--AAGCTAAATAGAAGGCATTTCGGTT

'RP1_m1_B' CAGTCTTTAAAATCAAATTAA--AAGCTAAATAGAAGGCATTTCGGTT

'RP1_m5(A)' CAGTCTTTAAAATCAAATTAA--AAGCTAAATAGAAGGCATTTCGGTT

'RP1_m5_B' CAGTCTTTAAAATCAAATTAA--AAGCTAAATAGAAGGCATTTCGGTT

'RP1_m7_A' CAGTCTTTAAAATCAAATTAA--AAGCTAAATAGAAGGCATTTCGGTT

'RP1_m7_B' CAGTCTTTAAAATCAAATTAACTTAGCTAAATAGAAGGCATTTCGGTT

'RP1_m8_A' CAGTCTTTAAAATCAAATTAA--AAGCTAAATAGAAGGCATTTCGGTT

'RP1_m8_B' CAGTCTTTAAAATCAAATTAA--AAGCTAAATAGAAGGCATTTCGGTT

'R_Abg' CAGTCTTTAAAATCAAATTAA--AAGCTAAATAGAAGGCATTTCGGTT

'R_Abg_2' CAGTCTTTAAAATCAAATTAA--AAGCTAAATAGAAGGCATTTCGGTT

'AB_m1_A' CAGTCTTTAAAATCAAATTAA--AAGCTAAATAGAAGGCATTTCGGTT

'AB_m1_B' CAGTCTTTAAAATCAAATTAA--AAGCTAAATAGAAGGCATTTCGGTT

Abh CAGTCTTTAAAATCAAATTAA--AAGCTAAATAGAAGGCATTTCGGTT

'Abh_2' CAGTCTTTAAAATCAAATTAA--AAGCTAAATAGAAGGCATTTCGGTT

'AB_m2_A' CAGTCTTTAAAATCAAATTAA--AAGCTAAATAGAAGGCATTTCGGTT

'AB_m2_B' CAGTCTTTAAAATCAAATTAA--AAGCTAAATAGAAGGCATTTCGGTT

'IP_m8_A' CAGTCTTTAAAATCAAATGAA--AAGCTAAATAGAAGGCATTTCGGTT

'IP_m8_B' CAGTCTTTAAAATCAAATTAA--AAGCTAAATAGAAGGCATTTCGGTT

'IP_f3_A' CAGTCTTTAAAATCAAATTAA--AAGCTAAATAGAAGGCATTTCGGTT

'IP_f3_B' CAGTCTTTAAAATCAAATTAA--AAGCTAAATAGAAGGCATTTCGGTT

'IP_f23_A' CAGTCTTTAAAATCAAATTAA--AAGCTAAATAGAAGGCATTTCGGTT

'IP_f23_B' CAGTCTTTAAAATCAAATTAA--AAGCTAAATAGAAGGCATTTCGGTT

'IP_m7_A' CAGTCTTTAAAATCAAATTAA--AAGCTAAATAGAAGGCATTTCGGTT

'IP_m7_B' CAGTCTTTAAAATCAAATTAA--AAGCTAAATAGAAGGCATTTCGGTT

'PVL_f14_A' CAGTCTTTAAAATCAAATTAA--AAGCTAAATAGAAGGCATTTCGGTT

'PVL_f14_B' CAGTCTTTAAAATCAAATTAA--AAGCTAAATAGAAGGCATTTCGGTT

'PVL_f12_A' CAGTCTTTAAAATCAAATGAA--AAGCTAAATAGAAGGCATTTCGGTT

'PVL_f12_B' CAGTCTTTAAAATCAAATGAA--AAGCTAAATAGAAGGCATTTCGGTT

'PVL_f3_A' CAGTCTTTAAAATCAAATTAA--AAGCTAAATAGAAGGCATTTCGGTT

'PVL_f3_B' CAGTCTTTAAAATCAAATTAA--AAGCTAAATAGAAGGCATTTCGGTT

'PVL_f7_A' CAGTCTTTAAAATCAAATTAA--AAGCTAAATAGAAGGCATTTCGGTT

'PVL_f7_B' CAGTCTTTAAAATCAAATTAA--AAGCTAAATAGAAGGCATTTCGGTT

'Pes_m9_A' CAGTGTTTAAAAGCTA-------------ATTGGAAGGCATTTAGGTT

'Pes_m9_B' CAGTCTTTAAAATCAAATTAA--AAGGTAATTGGAAGGCATTTAGGTT

'Pes_m10_A' CAGTGTTTAAAAGCTA-------------ATTGGAAGGCATTTAGGTT

'Pes_m10_B' CAGTGTTTAAAAGCTA-------------ATTGGAAGGCATTTAGGTT

'LH_m1_A' CAGTCTTTAAAATCAAATTAA--AAGGTAATTGGAAGGCATTTAGGTT

'LH_m1_B' CAGTCTTTAAAATCAAATTAA--AAGGTAATTGGAAGGCATTTAGGTT

'LH_f6_A' CAGTCTTTAAAATCAAATTAA--AAGGTAATTGGAAGGCATTTAGGTT

'LH_f6_B' CAGTCTTTAAAATCAAATTAA--AAGGTAATTGGAAGGCATTTAGGTT

'LH_f8_A' CAGTCTTTAAAATCAAATTAA--AAGGTAATTGGAAGGCATTTAGGTT

'LH_f8_B' CAGTCTTTAAAATCAAATTAA--AAGGTAATTGGAAGGCATTTAGGTT

'scn_h_a' CAGTCTTTAAAATCAAATTAA--AAGGTAATTGGAAGGCATTTAGGTT

'scn_h_b' CAGTCTTTAAAATCAAATTAA--AAGGTAATTGGAAGGCATTTAGGTT

'scn_m5_A' CAGTCTTTAAAATCAAATTAA--AAGGTAATTGGAAGGCATTTAGGTT

'scn_m5_B' CAGTCTTTAAAATCAAATTAA--AAGGTAATTGGAAGGCATTTAGGTT

'scn_m7_A' CAGTCTTTAAAATCAAATTAA--AAGGTAATTGGAAGGCATTTAGGTT

'scn_m7_B' CAGTCTTTAAAATCAAATTAA--AAGGTAATTGGAAGGCATTTAGGTT

'scn_m3_A' CAGTCTTTAAAATCAAATTAA--AAGGTAATTGGAAGGCATTTAGGTT

'scn_m3_B' CAGTCTTTAAAATCAAATTAA--AAGGTAATTGGAAGGCATTTAGGTT

'LJS_m1_A' CAGTCTTTACTATGAAATTAA--AACCGAAATGGAAGGCATTTAGGTT

'LJS_m1_B' CAGTCTTTACTATGAAATTAA--AACCGAAATGGAAGGCATTTAGGTT

'LJS_m2_A' CAGTCTTTACTATGAAATTAA--AACCGAAATGGAAGGCATTTAGGTT

'LJS_m2_B' CAGTCTTTACTATGAAATTAA--AACCGAAATGGAAGGCATTTAGGTT

'LJS_f10_A' CAGTCTTTACTATGAAATTAA--AACCGAAATGGAAGGCATTTAGGTT

'LJS_f10_B' CAGTCTTTACTATGAAATTAA--AACCGAAATGGAAGGCATTTAGGTT

'LJS_f9_B' CAGTCTTTACTATGAAATTAA--AACCGAAATGGAAGGCATTTAGGTT

'LJS_f9_A' CAGTCTTTACTATGAAATTAA--AACCGAAATGGAAGGCATTTAGGTT

'LJP_m7_B' CAGTCTTTACTATGAAATTAA--AACCGAAATGGAAGGCATTTAGGTT

'LJP_m7_A' CAGTCTTTACTATGAAATTAA--AACCGAAATGGAAGGCATTTAGGTT

'LJP_m6_B' CAGTCTTTACTATGAAATTAA--AACCGAAATGGAAGGCATTTAGGTT

'LJP_m6_A' CAGTCTTTACTATGAAATTAA--AACCGAAATGGAAGGCATTTAGGTT

'LJP_m2_B' CAGTCTTTACTATGAAATTAA--AACCGAAATGGAAGGCATTTAGGTT

'LJP_m2_A' CAGTCTTTACTATGAAATTAA--AACCGAAATGGAAGGCATTTAGGTT

'LJP_m1_B' CAGTCTTTACTATGAAATTAA--AACCGAAATGGAAGGCATTTAGGTT

'LJP_m1_A' CAGTCTTTACTATGAAATTAA--AACCGAAATGGAAGGCATTTAGGTT

'BR_m5_A' CAGTCTTTACTATGAAATTAA--AACCGAAATGGAAGGCATTTAGGTT

'BR_m5_B' CAGTCTTTACTATGAAATTAA--AACCGAAATGGAAGGCATTTAGGTT

'BR_m6_A' CAGTCTTTACTATGAAATTAA--AACCGAAATGGAAGGCATTTAGGTT

'BR_m6_B' CAGTCTTTACTATGAAATTAA--AACCGAAATGGAAGGCATTTAGGTT

'BR_m7_A' CAGTCTTTACTATGAAATTAA--AACCGAAATGAAGGGCATTTAGGTT

'BR_m7_B' CAGTCTTTACTATGAAATTAA--AACCGAAATGGAAGGCATTTAGGTT

'BR_f13_B' CAGTCTTTACTATGAAATTAA--AACCGAAATGGAAGGCATTTAGGTT

'BR_f13_A' CAGTCTTTACTATGAAATTAA--AACCGAAATGGAAGGCATTTAGGTT

'BR_m8_B' CAGTCTTTACTATGAAATTAA--AACCGAAATGGAAGGCATTTAGGTT

'BR_m8_A' CAGTCTTTACTATGAAATTAA--AACCGAAATGGAAGGCATTTAGGTT

'SD_h_a' CAGTCTTTACAATGAAATAAA-AACCCGAAATGGAAGGCATTTAGGTT

'SD_h_b' CAGTCTTTACAATGAAATAAA-AACCCGAAATGGAAGGCATTTAGGTT

'SD_g_a' CAGTCTTTACAATGAAATAAA-AACCCGAAATGGAAGGCATTTAGATT

'SD_g_b' CAGTCTTTACAATGAAATAAA-AACCCGAAATGGAAGGCATTTAGGTT

'SD_2m_b' CAGTCTTTACAATGAAATAAA-AACCCGAAATGGAAGGCATTTAGGTT

'SD_2m_a' CAGTCTTTACAATGAAATAAA-AACCCGAAATGGAAGGCATTTAGGTT

[ 200 210 220 230 240]

[ * * * * *]

'RP1_m1_A' AATCATCTATCCGGAAAGAGCTGT----ACATCTAATTGGTGCCCGAT

'RP1_m1_B' AATCATCTATCCGGAAAGAGCTGT----ACATCTAATTGGTGCCCGAT

'RP1_m5(A)' AATCATCTATCCGGAAAGAGCTGT----ACATCTAATTGGTGCCCGAT

'RP1_m5_B' AATCATCTATCCGGAAAGAGCTGT----ACATCTAATTGGTGCCCGAT

'RP1_m7_A' AATCATCTATCCGGAAAGAGCTGT----ACATCTAATTGGTGCCCGAT

'RP1_m7_B' AATCATCTATCCGGAAAGAGCTGT----ACATCTAATTGGTGCCCGAT

'RP1_m8_A' AATCATCTATCCGGAAAGAGCTGT----ACATCTAATTGGTGCCCGAT

'RP1_m8_B' AATCATCTATCCGGAAAGAGCTGT----ACATCTAATTGGTGCCCGAT

'R_Abg' AATCATCTATCCGGAAAGAGCTGT----ACATCTAATTGGTGCCCGAT

'R_Abg_2' AATCATCTATCCGGAAAGAGCTGT----ACATCTAATTGGTGCCCGAT

'AB_m1_A' AATCATCTATCCGGAAAGAGCTGT----ACATCTAATTGGTGCCCGAT

'AB_m1_B' AATCATCTATCCGGAAAGAGCTGT----ACATCTAATTGGTGCCCGAT

Abh AATCATCTATCCGGAAAGAGCTGT----ACATCTAATTGGTGCCCGAT

'Abh_2' AATCATCTATCCGGAAAGAGCTGT----ACATCTAATTGGTGCCCGAT

'AB_m2_A' AATCATCTATCCGGAAAGAGCTGT----ACATCTAATTGGTGCCCGAT

'AB_m2_B' AATCATCTATCCGGAAAGAGCTGT----ACATCTAATTGGTGCCCGAT

'IP_m8_A' AATCATCTATCCGGAAAGAGCTGT----ACATCTAATTGGTGCCCGAT

'IP_m8_B' AATCATCTATCCGGAAAGAGCTGT----ACATCTAATTGGTGCCCGAT

'IP_f3_A' AATCATCTATCCGGAAAGAGCTGT----ACATCTAATTGGTGCCCGAT

'IP_f3_B' AATCATCTATCCGGAAAGAGCTGT----ACATCTAATTGGTGCCCGAT

'IP_f23_A' AATCATCTATCCGGAAAGAGCTGT----ACATCTAATTGGTGCCCGAT

'IP_f23_B' AATCATCTATCCGGAAAGAGCTGT----ACATCTAATTGGTGCCCGAT

'IP_m7_A' AATCATCTATCCGGAAAGAGCTGT----ACATCTAATTGGTGCCCGAT

'IP_m7_B' AATCATCTATCCGGAAAGAGCTGT----ACATCTAATTGGTGCCCGAT

'PVL_f14_A' AATCATCTATCCGGAAAGAGCTGT----ACATCTAATTGGTGCCCGAT

'PVL_f14_B' AATCATCTATCCGGAAAGAGCTGT----ACATCTAATTGGTGCCCGAT

'PVL_f12_A' AATCATCTATCCGGAAAGAGCTGT----ACATCTAATTGGTGCCCGAT

'PVL_f12_B' AATCATCTATCCGGAAAGAGCTGT----ACATCTAATTGGTGCCCGAT

'PVL_f3_A' AATCATCTATCCGGAAAGAGCTGT----ACATCTAATTGGTGCCCGAT

'PVL_f3_B' AATCATCTATCCGGAAAGAGCTGT----ACATCTAATTGGTGCCCGAT

'PVL_f7_A' AATCATCTATCCGGAAAGAGCTGT----ACATCTAATTGGTGCCCGAT

'PVL_f7_B' AATCATCTATCCGGAAAGAGCTGT----ACATCTAATTGGTGCCCGAT

'Pes_m9_A' AATCATCTATCCGGAAAGAGCCGT----ACATCTAAT-----------

'Pes_m9_B' AATCATCTATCCGGAAAGAGCCGTCCGTACATCTAAT-----------

'Pes_m10_A' AATCATCTATCCGGAAAGAGCCGT----ACATCTAAT-----------

'Pes_m10_B' AATCATCTATCCGGAAAGAGCCGT----ACATCTAAT-----------

'LH_m1_A' AATCATCTATCCGGAAAGAGCCGTCCGTACATCTAAT-----------

'LH_m1_B' AATCATCTATCCGGAAAGAGCCGTCCGTACATCTAAT-----------

'LH_f6_A' AATCATCTATCCGGAAAGAGCCGTCCGTACATCTAAT-----------

'LH_f6_B' AATCATCTATCCGGAAAGAGCCGTCCGTACATCTAAT-----------

'LH_f8_A' AATCATCTATCCGGAAAGAGCCGTCCGTACATCTAAT-----------

'LH_f8_B' AATCATCTATCCGGAAAGAGCCGTCCGTACATCTAAT-----------

'scn_h_a' AATCATCTATCCGGAAAGAGCCGTCCGTACATCTAAT-----------

'scn_h_b' AATCATCTATCCGGAAAGAGCCGTCCGTACATCTAAT-----------

'scn_m5_A' AATCATCTATCCGGAAAGAGCCGTCCGTACATCTAAT-----------

'scn_m5_B' AATCATCTATCCGGAAAGAGCCGTCCGTACATCTAAT-----------

'scn_m7_A' AATCATCTATCCGGAAAGAGCCGTCCGTACATCTAAT-----------

'scn_m7_B' AATCATCTATCCGGAAAGAGCCGTCCGTACATCTAAT-----------

'scn_m3_A' AATCATCTATCCGGAAAGAGCCGTCCGTACATCTAAT-----------

'scn_m3_B' AATCATCTATCCGGAAAGAGCCGTCCGTACATCTAAT-----------

'LJS_m1_A' CGTCATCTATCCGGTAAGAGCCGT----ACATCTAATTGGTGCCCGAT

'LJS_m1_B' CGTCATCTATCCGGTAAGAGCCGT----ACATCTAATTGGTGCCCGAT

'LJS_m2_A' CGTCATCTATCCGGTAAGAGCCGT----ACATCTAATTGGTGCCCGAT

'LJS_m2_B' CGTCATCTATCCGGTAAGAGCCGT----ACATCTAATTGGTGCCCGAT

'LJS_f10_A' CGTCATCTATCCGGTAAGAGCCGT----ACATCTAATTGGTGCCCGAT

'LJS_f10_B' CGTCATCTATCCGGTAAGAGCCGT----ACATCTAATTGGTGCCCGAT

'LJS_f9_B' CGTCATCTATCCGGTAAGAGCCGT----ACATCTAATTGGTGCCCGAT

'LJS_f9_A' CGTCATCTATCCGGTAAGAGCCGT----ACATCTAATTGGTGCCCGAT

'LJP_m7_B' TGTCATCTATCCGGTAAGAGCCGT----ACATCTAATTGGTGCCCGAT

'LJP_m7_A' CGTCATCTATCCGGTAAGAGCCGT----ACATCTAATTGGTGCCCGAT

'LJP_m6_B' CGTCATCTATCCGGTAAGAGCCGT----ACATCTAATTGGTGCCCGAT

'LJP_m6_A' CGTCATCTATCCGGTAAGAGCCGT----ACATCTAATTGGTGCCCGAT

'LJP_m2_B' CGTCATCTATCCGGTAAGAGCCGT----ACATCTAATTGGTGCCCGAT

'LJP_m2_A' CGTCATCTATCCGGTAAGAGCCGT----ACATCTAATTGGTGCCCGAT

'LJP_m1_B' CGTCATCTATCCGGTAAGAGCCGT----ACATCTAATTGGTGCCCGAT

'LJP_m1_A' CGTCATCTATCCGGTAAGAGCCGT----ACATCTAATTGGTGCCCGAT

'BR_m5_A' CGTCATCTATCCGGTAAGAGCCGT----ACATCTAATTGGTGCCCGAT

'BR_m5_B' CGTCATCTATCCGGTAAGAGCCGT----ACATCTAATTGGTGCCCGAT

'BR_m6_A' CGTCATCTATCCGGTAAGAGCCGT----ACATCTAATTGGTGCCCGAT

'BR_m6_B' CGTCATCTATCCGGTAAGAGCCGT----ACATCTAATTGGTGCCCGAT

'BR_m7_A' CGTCATCTATCCGGTAAGAGCCGT----ACATCTAATTGGTGCCCGAT

'BR_m7_B' CGTCATCTATCCGGTAAGAGCCGT----ACATCTAATTGGTGCCCGAT

'BR_f13_B' CGTCATCTATCCGGTAAGAGCCGT----ACATCTAATTGGTGCCCGAT

'BR_f13_A' CGTCATCTATCCGGTAAGAGCCGT----ACATCTAATTGGTGCCCGAT

'BR_m8_B' CGTCATCTATCCGGTAAGAGCCGT----ACATCTAATTGGTGCCCGAT

'BR_m8_A' CGTCATCTATCCGGTAAGAGCCGT----ACATCTAATTGGTGCCCGAT

'SD_h_a' CATCATCTATCCGGTAAGAGCCGT----ACATCTAATTGGTGCCCTAT

'SD_h_b' CGTCATCTATCCGGTAAGAGCCGT----ACATCTAATTGGTGCCCTAT

'SD_g_a' CATCATCTATCCGGTAAGAGCCGT----ACATCTAATTGGTGCCCTAT

'SD_g_b' CGTCATCTATCCGGTAAGAGCCGT----ACATCTAATTGGTGCCCTAT

'SD_2m_b' CATCATCTATCCGGTAAGAGCCGT----ACATCTAATTGGTGCCCTAT

'SD_2m_a' CATCATCTATCCGGTAAGAGCCGT----ACATCTAATTGGTGCCCTAT

[ 250 260 270 280 ]

[ * * * * ]

'RP1_m1_A' ATGCCTGCACGGCTTGCACGCCGATGGTATGGAATCCTTGAAGTCTTC

'RP1_m1_B' ATGCCTGCACGGCTTGCACGCCGATGGTATGGAATCCTTGAAGTCTTC

'RP1_m5(A)' ATGCCTGCACGGCTTGCACGCCGATGGTATGGAATCCTTGAAGTCTTC

'RP1_m5_B' ATGCCTGCACGGCTTGCACGCCGATGGTATGGAATCCTTGAAGTCTTC

'RP1_m7_A' ATGCCTGCACGGCTTGCACGCCGATGGTATGGAATCCTTGAAGTCTTC

'RP1_m7_B' ATGCCTGCACGGCTTGCACGCCGATGGTATGGAATCCTTGAAGTCTTC

'RP1_m8_A' ATGCCTGCACGGCTTGCACGCCGATGGTATGGAATCCTTGAAGTCTTC

'RP1_m8_B' ATGCCTGCACGGCTTGCACGCCGATGGTATGGAATCCTTGAAGTCTTC

'R_Abg' ATGCCTGCACGGCCTGCACGCCGAGGGTATGGAATCCTTGAAGTCTTC

'R_Abg_2' ATGCCTGCACGGCCTGCACGCCGAGGGTATGGAATCCTTGAAGTCTTC

'AB_m1_A' ATGCCTGCACGGCTTGCACGCCGATGGTATGGAATCCTTGAAGTCTTC

'AB_m1_B' ATGCCTGCACGGCCTGCACGCCGAGGGTATGGAATCCTTGAAGTCTTC

Abh ATGCCTGCACGGCCTGCACGCCGAGGGTATGGAATCCTTGAAGTCTTC

'Abh_2' ATGCCTGCACGGCCTGCACGCCGAGGGTATGGAATCCTTGAAGTCTTC

'AB_m2_A' ATGCCTGCACGGCTTGCACGCCGATGGTATGGAATCCTTGAAGTCTTC

'AB_m2_B' ATGCCTGCACGGCCTGCACGCCGAGGGTATGGAATCCTTGAAGTCTTC

'IP_m8_A' ATGCCTGCACGGCTTGCACGCCGATGGTATGGAATCCTTGAAGTCTTC

'IP_m8_B' ATGCCTGCACGGCTTGCACGCCGATGGTATGGAATCCTTGAAGTCTTC

'IP_f3_A' ATGCCTGCACGGCTTGCACGCCGATGGTATGGAATCCTTGAAGTCTTC

'IP_f3_B' ATGCCTGCACGGCTTGCACGCCGATGGTATGGAATCCTTGAAGTCTTC

'IP_f23_A' ATGCCTGCACGGCTTGCACGCCGATGGTATGGAATCCTTGAAGTCTTC

'IP_f23_B' ATGCCTGCACGGCCTGCACGCCGAGGGTATGGAATCCTTGAAGTCTTC

'IP_m7_A' ATGCCTGCACGGCTTGCACGCCGATGGTATGGAATCCTTGAAGTCTTC

'IP_m7_B' ATGCCTGCACGGCTTGCACGCCGATGGTATGGAATCCTTGAAGTCTTC

'PVL_f14_A' ATGCCTGCACGGCCTGCACGCCGAGGGTATGGAATCCTTGAAGTCTTC

'PVL_f14_B' ATGCCTGCACGGCCTGCACGCCGAGGGTATGGAATCCTTGAAGTCTTC

'PVL_f12_A' ATGCCTGCACGGCTTGCACGCCGATGGTATGGAATCCTTGAAGTCTTC

'PVL_f12_B' ATGCCTGCACGGCTTGCACGCCGATGGTATGGAATCCTTGAAGTCTTC

'PVL_f3_A' ATGCCTGCACGGCCTGCACGCCGAGGGTATGGAATCCTTGAAGTCTTC

'PVL_f3_B' ATGCCTGCACGGCCTGCACGCCGAGGGTATGGAATCCTTGAAGTCTTC

'PVL_f7_A' ATGCCTGCACGGCCTGCACGCCGAGGGTATGGAATCCTTGAAGTCTTC

'PVL_f7_B' ATGCCTGCACGGCCTGCACGCCGAGGGTATGGAATCCTTGAAGTCTTC

'Pes_m9_A' --------------TGGAATCCGAAGGTATGGAATTCTTGAAGTGTTC

'Pes_m9_B' --------------TGGAATCCGAAGGTATGGAATCCTTGAATTGTTC

'Pes_m10_A' --------------TGGAATCCGAAGGTATGGAATTCTTGAAGTGTTC

'Pes_m10_B' --------------TGGAATCCGAAGGTATGGAATTCTTGAAGTGTTC

'LH_m1_A' --------------TGGAATCCGAAGGTATGGAATCCTTGAATTGTTC

'LH_m1_B' --------------TGGAATCCGAAGGTATGGAATCCTTGAATTGTTC

'LH_f6_A' --------------TGGAATCCGAAGGTATGGAATCCTTGAATTGTTC

'LH_f6_B' --------------TGGAATCCGAAGGTATGGAATCCTTGAATTGTTC

'LH_f8_A' --------------TGGAATCCGAAGGTATGGAATCCTTGAATTGTTC

'LH_f8_B' --------------TGGAATCCGAAGGTATGGAATCCTTGAATTGTTC

'scn_h_a' --------------TGGAATCCGAAGGTATGGAATCCTTGAATTGTTC

'scn_h_b' --------------TGGAATCCGAAGGTATGGAATCCTTGAATTGTTC

'scn_m5_A' --------------TGGAATCCGAAGGTATGGAATCCTTGAATTGTTC

'scn_m5_B' --------------TGGAATCCGAAGGTATGGAATCCTTGAATTGTTC

'scn_m7_A' --------------TGGAATCCGAAGGTATGGAATCCTTGAATTGTTC

'scn_m7_B' --------------TGGAATCCGAAGGTATGGAATCCTTGAATTGTTC

'scn_m3_A' --------------TGGAATCCGAAGGTATGGAATCCTTGAATTGTTC

'scn_m3_B' --------------TGGAATCCGAAGGTATGGAATCCTTGAATTGTTC

'LJS_m1_A' ACGCC---------TGCACGCCGATGGCATGGAATCCTTGAAGTCTAC

'LJS_m1_B' ACGCC---------TGCACGCCGATGGCATGGAATCCTTGAAGTCTAC

'LJS_m2_A' ACGCC---------TGCACGCCGAGGGCATGGAATCCTTGAAGTCTAC

'LJS_m2_B' ACGCC---------TGCACGCCGATGGCATGGAATCCTTGAAGTCTAC

'LJS_f10_A' ACGCC---------TGCACGCCGATGGCATGGAATCCTTGAAGTCTAC

'LJS_f10_B' ACGCC---------TGCACGCCGATGGCATGGAATCCTTGAAGTCTAC

'LJS_f9_B' ACGCC---------TGCACGCCGATGGCATGGAATCCTTGAAGTCTAC

'LJS_f9_A' ACGCC---------TGCACGCCGATGGCATGGAATCCTTGAAGTCTAC

'LJP_m7_B' ACGCC---------TGCACGCCGAGGGCATGGAATCCTTGAAGTCTAC

'LJP_m7_A' ACGCC---------TGCACGCCGATGGCATGGAATCCTTGAAGTCTAC

'LJP_m6_B' ACGCC---------TGCACGCCGATGGCATGGAATCCTTGAAGTCTAC

'LJP_m6_A' ACGCC---------TGCACGCCGATGGCATGGAATCCTTGAAGTCTAC

'LJP_m2_B' ACGCC---------TGCACGCCGAGGGCATGGAATCCTTGAAGTCTAC

'LJP_m2_A' ACGCC---------TGCACGCCGAGGGCATGGAATCCTTGAAGTCTAC

'LJP_m1_B' ACGCC---------TGCACGCCGATGGCATGGAATCCTTGAAGTCTAC

'LJP_m1_A' ACGCC---------TGCACGCCGATGGCATGGAATCCTTGAAGTCTAC

'BR_m5_A' ACGCC---------TGCACGCCGAGGGCATGGAATCCTTGAAGTCTAC

'BR_m5_B' ACGCC---------TGCACGCCGAGGGCATGGAATCCTTGAAGTCTAC

'BR_m6_A' ACGCC---------TGCACGCCGAGGGCATGGAATCCTTGAAGTCTAC

'BR_m6_B' ACGCC---------TGCACGCCGAGGGCATGGAATCCTTGAAGTCTAC

'BR_m7_A' ACGCC---------TGCACGCCGAGGGCATGGAATCCTTGAAGTCTAC

'BR_m7_B' ACGCC---------TGCACGCCGAGGGCATGGAATCCTTGAAGTCTAC

'BR_f13_B' ACGCC---------TGCACGCCGATGGCATGGAATCCTTGAAGTCTAC

'BR_f13_A' ACGCC---------TGCACGCCGAGGGCATGGAATCCTTGAAGTCTAC

'BR_m8_B' ACGCC---------TGCACGCCGAGGGCATGGAATCCTTGAAGTCTAC

'BR_m8_A' ACGCC---------TGCACGCCGAGGGCATGGAATCCTTGAAGTCTAC

'SD_h_a' ACGCC---------TGCACGCCGATGGTATGGAATCCTTGAAGTCTTC

'SD_h_b' ACGCC---------TGCACGCCGATGGTATGGAATCCTTGAAGTCTTC

'SD_g_a' ACGCC---------TGCACGCCGATGGTATGGAATCCTTGAAGTCTTC

'SD_g_b' ACGCC---------TGCACGCCGATGGTATGGAATCCTTGAAGTCTTC

'SD_2m_b' ACGCC---------TGCACGCCGATGGTATGGAATCCTTGAAGTCTTC

'SD_2m_a' ACGCC---------TGCACGCCGATGGTATGGAATCCTTGAAGTCTTC

[ 290 300 310 320 330 ]

[ * * * * * ]

'RP1_m1_A' -AAACTTTGACTAATGATCGACTTTACGATGAAGGATAGCTTTTGTGG

'RP1_m1_B' -AAACTTTGACTAATGATCGACTTTACGATGAAGGATAGCTTTTGTGG

'RP1_m5(A)' -AAACTTTGACTAATGATCGACTTTACGATGAAGGATAGCTTTTGTGG

'RP1_m5_B' -AAACTTTGACTAATGATCGACTTTACGATGAAGGATAGCTTTTGTGG

'RP1_m7_A' -AAACTTTGACTAATGATCGACTTTACGATGAAGGATAGCTTTTGTGG

'RP1_m7_B' -AAACTTTGACTAATGATCGACTTTACGATGAAGGATAGCTTTTGTGG

'RP1_m8_A' -AAACTTTGACTAATGATCGACTTTACGATGAAGGATAGCTTTTGTGG

'RP1_m8_B' -AAACTTTGACTAATGATCGACTTTACGATGAAGGATAGCTTTTGTGG

'R_Abg' -AAACTTTGACTAATG-TCGACTTTACGATGAAGGATAGCTTTTGTGG

'R_Abg_2' -AAATTTTGACTAATG-TCGACTTTACGATGAAGGATAGCTTTTGTGG

'AB_m1_A' -AAACTTTGACTAATGATCGACTTTACGATGAAGGATAGC-TTTGTGG

'AB_m1_B' -AAATTTTGACTAATG-TCGACTTTACGATGAAGGATAGCTTTTGTGG

Abh -AAATTTTGACTAATG-TCGACTTTACGATGAAGGATAGCTTTTGTGG

'Abh_2' -AAATTTTGACTAATG-TCGACTTTACGATGAAGGATAGCTTTTGTGG

'AB_m2_A' -AAACTTTGACTAATGATCGACTTTACGATGAAGGATAGC-TTTGTGG

'AB_m2_B' -AAATTTTGACTAATG-TCGACTTTACGATGAAGGATAGCTTTTGTGG

'IP_m8_A' -AAACTTTGACTAATGATCGACTTTACGATGAAGGATAGC-TTTGTGG

'IP_m8_B' -AAACTTTGACTAATGATCGACTTTACGATGAAGGATAGC-TTTGTGG

'IP_f3_A' -AAACTTTGACTAATG-TCGACTTTACGATGAAGGATAGCTTTTGTGG

'IP_f3_B' -AAACTTTGACTAATGATCGACTTTACGATGAAGGATAGC-TTTGTGG

'IP_f23_A' -AAACTTTGACTAATGATCGACTTTACGATGAAGGATAGC-TTTGTGG

'IP_f23_B' -AAACTTTGACTAATG-TCGACTTTACGATGAAGGATAGCTTTTGTGG

'IP_m7_A' -AAACTTTGACTAATGATCGACTTTACGATGAAGGATAGC-TTTGTGG

'IP_m7_B' -AAACTTTGACTAATGATCGACTTTACGATGAAGGATAGCTTTTGTGG

'PVL_f14_A' -AAACTTTGACTAATG-TCGACTTTACGATGAAGGATAGCTTTTGTGG

'PVL_f14_B' -AAACTTTGACTAATG-TCGACTTTACGATGAAGGATAGCTTTTGTGG

'PVL_f12_A' -AAACTTTGACTAATGATCGACTTTACGATGAAGGATAGC-TTTGTGG

'PVL_f12_B' -AAACTTTGACTAATGATCGACTTTACGATGAAGGATAGC-TTTGTGG

'PVL_f3_A' -AAACTTTGACTAATG-TCGACTTTACGATGAAGGATAGCTTTTGTGG

'PVL_f3_B' -AAACTTTGACTAATG-TCGACTTTACGATGAAGGATAGCTTTTGTGG

'PVL_f7_A' -AAACTTTGACTAATG-TCGACTTTACGATGAAGGATAGCTTTTGTGG

'PVL_f7_B' -AAACTTTGACTAATG-TCGACTTTACGATGAAGGATAGCTTTTGTGG

'Pes_m9_A' -AAACTTTGACAAATG-TCGACTTTACGATGAAGGATAGC-TTTGTGG

'Pes_m9_B' -AAACTTTGACAAATA-TCGACTTTACGATGAAGGATAGC-TTTGTGG

'Pes_m10_A' -AAACTTTGACAAATG-TCGACTTTACGATGAAGGATAGC-TTTGTGG

'Pes_m10_B' -AAACTTTGACAAATG-TCGACTTTACGATGAAGGATAGC-TTTGTGG

'LH_m1_A' -AAACTTTGACAAATA-TCGACTTTACGATGAAGGATAGC-TTTGTGG

'LH_m1_B' -AAACTTTGACAAATA-TCGACTTTACGATGAAGGATAGC-TTTGTGG

'LH_f6_A' -AAACTTTGACAAATA-TCGACTTTACGATGAAGGATAGC-TTTGTGG

'LH_f6_B' -AAACTTTGACAAATA-TCGACTTTACGATGAAGGATAGC-TTTGTGG

'LH_f8_A' -AAACTTTGACAAATA-TCGACTTTACGATGAAGGATAGC-TTTGTGG

'LH_f8_B' -AAACTTTGACAAATA-TCGACTTTACGATGAAGGATAGC-TTTGTGG

'scn_h_a' -AAACTTTGACAAATA-TCGACTTTACGATGAAGGATAGC-TTTGTGG

'scn_h_b' -AAACTTTGACAAATA-TCGACTTTACGATGAAGGATAGC-TTTGTGG

'scn_m5_A' -AAACTTTGACAAATA-TCGACTTTACGATGAAGGATAGC-TTTGTGG

'scn_m5_B' -AAACTTTGACAAATA-TCGACTTTACGATGAAGGATAGC-TTTGTGG

'scn_m7_A' -AAACTTTGACAAATA-TCGACTTTACGATGAAGGATAGC-TTTGTGG

'scn_m7_B' -AAACTTTGACAAATA-TCGACTTTACGATGAAGGATAGC-TTTGTGG

'scn_m3_A' -AAACTTTGACAAATA-TCGACTTTACGATGAAGGATAGC-TTTGTGG

'scn_m3_B' -AAACTTTGACAAATA-TCGACTTTACGATGAAGGATAGC-TTTGTGG

'LJS_m1_A' AAAAATTTGACGAATG-TCCACTTTACGATGAAGGATAGC-TTTGTGG

'LJS_m1_B' AAAAATTTGACGAATG-TCCACTTTACGATGAAGGATAGC-TTTGTGG

'LJS_m2_A' AAAAATTTGACGAATG-TCCACTTTACGATGAAGGATAGC-TTTGTGG

'LJS_m2_B' AAAAATTTGACGAATG-TCCACTTTACGATGAAGGATAGC-TTTGTGG

'LJS_f10_A' AAAAATTTGACGAATG-TCCACTTTACGATGAAGGATAGC-TTTGTGG

'LJS_f10_B' AAAAATTTGACGAATG-TCCACTTTACGATGAAGGATAGC-TTTGTGG

'LJS_f9_B' AAAAATTTGACGAATG-TCCACTTTACGATGAAGGATAGC-TTTGTGG

'LJS_f9_A' AAAAATTTGACGAATG-TCCACTTTACGATGAAGGATAGC-TTTGTGG

'LJP_m7_B' AAAAATTTGACGAATG-TCCACTTTACGATGAAGGATAGC-TTTGTGG

'LJP_m7_A' AAAAATTTGACGAATG-TCCACTTTACGATGAAGGATAGC-TTTGTGG

'LJP_m6_B' AAAAATTTGACGAATG-TCCACTTTACGATGAAGGATAGC-TTTGTGG

'LJP_m6_A' AAAAATTTGACGAATG-TCCACTTTACGATGAAGGATAGC-TTTGTGG

'LJP_m2_B' AAAAATTTGACGAATG-TCCACTTTACGATGAAGGATAGC-TTTGTGG

'LJP_m2_A' AAAAATTTGACGAATG-TCCACTTTACGATGAAGGATAGC-TTTGTGG

'LJP_m1_B' AAAAATTTGACGAATG-TCCACTTTACGATGAAGGATAGC-TTTGTGG

'LJP_m1_A' AAAAATTTGACGAATG-TCCACTTTACGATGAAGGATAGC-TTTGTGG

'BR_m5_A' AAAATTTTGACGAATG-TCCACTTTACGATGAAGGATAGC-TTTGTGG

'BR_m5_B' AAAAATTTGACGAATG-TCCACTTTACGATGAAGGATAGC-TTTGTGG

'BR_m6_A' AAAAATTTGACGAATG-TCCACTTTACGATGAAGGATAGC-TTTGTGG

'BR_m6_B' AAAAATTTGACGAATG-TCCACTTTACGATGAAGGATAGC-TTTGTGG

'BR_m7_A' AAAAATTTGACGAATG-TCCACTTTACGATGAAGGATAGC-TTTGTGG

'BR_m7_B' AAAAATTTGACGAATG-TCCACTTTACGATGAAGGATAGC-TTTGTGG

'BR_f13_B' AAAAATTTGACGAATG-TCCACTTTACGATGAAGGATAGC-TTTGTGG

'BR_f13_A' AAAAATTTGACGAATG-TCCACTTTACGATGAAGGATAGC-TTTGTGG

'BR_m8_B' AAAAATTTGACGAATG-TCCACTTTACGATGAAGGATAGC-TTTGTGG

'BR_m8_A' AAAAATTTGACGAATG-TCCACTTTACGATGAAGGATAGC-TTTGTGG

'SD_h_a' -AAACTTAGACGAATG-TCCACTTTACGATGAAGGATAGC-TTTGTGG

'SD_h_b' -AAACTTAGACGAATG-TCCACTTTACGATGAAGGATAGC-TTTGTGG

'SD_g_a' -AAACTTAGACGAATG-TCCACTTTACGATGAAGGATAGC-TTTGTGG

'SD_g_b' -AAACTTTGACGAATG-TCCACTTTACGATGAAGGATAGC-TTTGTGG

'SD_2m_b' -AAACTTAGACGAATG-TCCACTTTACGATGAAGGATAGC-TTTGTGG

'SD_2m_a' -AAACTTAGACGAATG-TCCACTTTACGATGAAGGATAGC-TTTGTGG

[ 340 350 360 370 380 ]

[ * * * * * ]

'RP1_m1_A' TCAATGGAAGTATCCAATAGTTCAACGTTGT---------GTCCGATC

'RP1_m1_B' TCAATGGAAGTATCCAATAGTTCAACGTTGT---------GTCCGATC

'RP1_m5(A)' TCAATGGAAGTATCCAATAGTTCAACGTTGT---------GTCCGATC

'RP1_m5_B' TCAATGGAAGTATCCAATAGTTCAACGTTGT---------GTCCGATC

'RP1_m7_A' TCAATGGAAGTATCCAATAGTTCAACGTTGT---------GTCCGATC

'RP1_m7_B' TCAATGGAAGTATCCAATAGTTCAACGTTGT---------GTCCGATC

'RP1_m8_A' TCAATGGAAGTATCCAATAGTTCAACGTTGT---------GTCCGATC

'RP1_m8_B' TCAATGGAAGTATCCAATAGTTCAACGTTGT---------GTCCGATC

'R_Abg' TCAATGGAAGTATCTAATAGTTCAACGTTGT---------GTCCGATC

'R_Abg_2' TCAATGGAAGTATCTAATAGTTCAACGTTGT---------GTCCGATC

'AB_m1_A' TCAATGGAAGTATCCAATAGTTCAACGTTGT---------GTCCGATC

'AB_m1_B' TCAATGGAAGTATCTAATAGTTCAACGTTGT---------GTCCGATC

Abh TCAATGGAAGTATCTAATAGTTCAACGTTGT---------GTCCGATC

'Abh_2' TCAATGGAAGTATCTAATAGTTCAACGTTGT---------GTCCGATC

'AB_m2_A' TCAATGGAAGTATCCAATAGTTCAACGTTGT---------GTCCGATC

'AB_m2_B' TCAATGGAAGTATCTAATAGTTCAACGTTGT---------GTCCGATC

'IP_m8_A' TCAATGGAAGTATCCAATAGTTCAACGTTGT---------GTCCGATC

'IP_m8_B' TCAATGGAAGTATCCAATAGTTCAACGTTGT---------GTCCGATC

'IP_f3_A' TCAATGGAAGTATCTAATAGTTCAACGTTGT---------GTCCGATC

'IP_f3_B' TCAATGGAAGTATCCAATAGTTCAACGTTGT---------GTCCGATC

'IP_f23_A' TCAATGGAAGTATCCAATAGTTCAACGTTGT---------GTCCGATC

'IP_f23_B' TCAATGGAAGTATCTAATAGTTCAACGTTGT---------GTCCGATC

'IP_m7_A' TCAATGGAAGTATCCAATAGTTCAACGTTGT---------GTCCGATC

'IP_m7_B' TCAATGGAAGTATCCAATAGTTCAACGTTGT---------GTCCGATC

'PVL_f14_A' TCAATGGAAGTATCTAATAGTTCAACGTTGT---------GTCCGATC

'PVL_f14_B' TCAATGGAAGTATCTAATAGTTCAACGTTGT---------GTCCGATC

'PVL_f12_A' TCAATGGAAGTATCCAATAGTTCAACGTTGT---------GTCCGATC

'PVL_f12_B' TCAATGGAAGTATCCAATAGTTCAACGTTGT---------GTCCGATC

'PVL_f3_A' TCAATGGAAGTATCTAATAGTTCAACGTTGT---------GTCCGATC

'PVL_f3_B' TCAATGGAAGTATCTAATAGTTCAACGTTGT---------GTCCGATC

'PVL_f7_A' TCAATGGAAGTATCTAATAGTTCAACGTTGT---------GTCCGATC

'PVL_f7_B' TCAATGGAAGTATCTAATAGTTCAACGTTGT---------GTCCGATC

'Pes_m9_A' TCAATGGAAGTAACCAATAGTTCAAAGTTGT---------GTCCGATC

'Pes_m9_B' TCAATGGAAGTAACCAATAGTTCAAAGTTGT---------GTCCGATC

'Pes_m10_A' TCAATGGAAGTAACCAATAGTTCAAAGTTGT---------GTCCGATC

'Pes_m10_B' TCAATGGAAGTAACCAATAGTTCAAAGTTGT---------GTCCGATC

'LH_m1_A' TCAATGGAAGTAACCAATAGTTCAACGTTGT---------GTCCGATC

'LH_m1_B' TCAATGGAAGTAACCAATAGTTCAACGTTGT---------GTCCGATC

'LH_f6_A' TCAATGGAAGTAACCAATAGTTCAACGTTGT---------GTCCGATC

'LH_f6_B' TCAATGGAAGTAACCAATAGTTCAACGTTGT---------GTCCGATC

'LH_f8_A' TCAATGGAAGTAACCAATAGTTCAACGTTGT---------GTCCGATC

'LH_f8_B' TCAATGGAAGTAACCAATAGTTCAACGTTGT---------GTCCGATC

'scn_h_a' TCAATGGAAGTAACCAATAGTTCAACGTTGT---------GTCCGATC

'scn_h_b' TCAATGGAAGTAACCAATAGTTCAACGTTGT---------GTCCGATC

'scn_m5_A' TCAATGGAAGTAACCAATAGTTCAACGTTGT---------GTCCGATC

'scn_m5_B' TCAATGGAAGTAACCAATAGTTCAACGTTGT---------GTCCGATC

'scn_m7_A' TCAATGGAAGTAACCAATAGTTCAACGTTGT---------GTCCGATC

'scn_m7_B' TCAATGGAAGTAACCAATAGTTCAACGTTGT---------GTCCGATC

'scn_m3_A' TCAATGGAAGTAACCAATAGTTCAACGTTGT---------GTCCGATC

'scn_m3_B' TCAATGGAAGTAACCAATAGTTCAACGTTGT---------GTCCGATC

'LJS_m1_A' TCAATGGAAGTATCCAATAGTTCAACCTTGTGTCCAATAGGTCCGATC

'LJS_m1_B' TCAATGGAAGTATCCAATAGTTCAACCTTGTGTCCAATAGGTCCGATC

'LJS_m2_A' TCAATGGAAGTATCCAATAGTTCAACCTTGTGTCCAATAGGTCCGATC

'LJS_m2_B' TCAATGGAAGTATCCAATAGTTCAACCTTGTGTCCAATAGGTCCGATC

'LJS_f10_A' TCAATGGAAGTATCCAATAGTTCAACCTTGTGTCCAATAGGTCCGATC

'LJS_f10_B' TCAATGGAAGTATCCAATAGTTCAACCTTGTGTCCAATAGGTCCGATC

'LJS_f9_B' TCAATGGAAGTATCCAATAGTTCAACCTTGTGTCCAATAGGTCCGATC

'LJS_f9_A' TCAATGGAAGTATCCAATAGTTCAACCTTGTGTCCAATAGGTCCGATC

'LJP_m7_B' TCAATGGAAGTATCCAATAGTTCAACCTTGTGTCCAATAGGTCCGATC

'LJP_m7_A' TCAATGGAAGTATCCAATAGTTCAACCTTGTGTCCAATAGGTCCGATC

'LJP_m6_B' TCAATGGAAGTATCCAATAGTTCAACCTTGTGTCCAATAGGTCCGATC

'LJP_m6_A' TCAATGGAAGTATCCAATAGTTCAACCTTGTGTCCAATAGGTCCGATC

'LJP_m2_B' TCAATGGAAGTATCCAATAGTTCAACCTTGTGTCCAATAGGTCCGATC

'LJP_m2_A' TCAATGGAAGTATCCAATAGTTCAACCTTGTGTCCAATAGGTCCGATC

'LJP_m1_B' TCAATGGAAGTATCCAATAGTTCAACCTTGTGTCCAATAGGTCCGATC

'LJP_m1_A' TCAATGGAAGTATCCAATAGTTCAACCTTGTGTCCAATAGGTCCGATC

'BR_m5_A' TCAATGGAAGTATCCAATAGTTCAACCTTGTGTCCAATAGGTCCGATC

'BR_m5_B' TCAATGGAAGTATCCAATAGTTCAACCTTGTGTCCAATAGGTCCGATC

'BR_m6_A' TCAATGGAAGTATCCAATAGTTCAACCTTGTGTCCAATAGGTCCGATC

'BR_m6_B' TCAATGGAAGTATCCAATAGTTCAACCTTGTGTCCAATAGGTCCGATC

'BR_m7_A' TCAATGGAAGTATCCAATAGTTCAACCTTGTGTCCAATAGGTCCGATC

'BR_m7_B' TCAATGGAAGTATCCAATAGTTCAACCTTGTGTCCAATAGGTCCGATC

'BR_f13_B' TCAATGGAAGTATCCAATAGTTCAACCTTGTGTCCAATAGGTCCGATC

'BR_f13_A' TCAATGGAAGTATCCAATAGTTCAACCTTGTGTCCAATAGGTCCGATC

'BR_m8_B' TCAATGGAAGTATCCAATAGTTCAACCTTGTGTCCAATAGGTCCGATC

'BR_m8_A' TCAATGGAAGTATCCAATAGTTCAACCTTGTGTCCAATAGGTCCGATC

'SD_h_a' TCAATGGAAGTATCCAATAGTTCAACCTTGTGTCCGATAGGTCCGATC

'SD_h_b' TCAATGGAAGTATCCAATAGTTCAACCTTGTGTCCGATAGGTCCGATC

'SD_g_a' TCAATGGAAGTATCCAATAGTTCAACCTTGTGTCCGATAGGTCCGATC

'SD_g_b' TCAATGGAAGTATCCAATAGTTCAACCTTGTGTCCGATAGGTCCGATC

'SD_2m_b' TCAATGGAAGTATCCAATAGTTCAACCTTGTGTCCGATAGGTCCGATC

'SD_2m_a' TCAATGGAAGTATCCAATAGTTCAACCTTGTGTCCGATAGGTCCGATC

[ 390 400 410 420 430 ]

[ * * * * * ]

'RP1_m1_A' ATCATTATGCGAAGAGCGAACTTTTTTT-TTCTTCTTTACTGTAGGTC

'RP1_m1_B' ATCATTATGCGAAGAGCGAACTTTTTTT-TTCTTCTTTACTGTAGGTC

'RP1_m5(A)' ATCATTATGCGAAGAGCGAACTTTTTTT-TTCTTCTTTACTGTAGGTC

'RP1_m5_B' ATCATTATGCGAAGAGCGAACTTTTTTT-TTCTTCTTTACTGTAGGTC

'RP1_m7_A' ATCATTATGCGAAGAGCGAACTTTTTTT-TTCTTCTTTACTGTAGGTC

'RP1_m7_B' ATCATTATGCGAAGAGCGAACTTTTTTT-TTCTTCTTTACTGTAGGTC

'RP1_m8_A' ATCATTATGCGAAGAGCGAACTTTTTTT-TTCTTCTTTACTGTAGGTC

'RP1_m8_B' ATCATTATGCGAAGAGCGAACTTTTTTT-TTCTTCTTTACTGTAGGTC

'R_Abg' ATCATTATGCGAAGAGCGAACTTTTTTT-TTCTTCTTTACTGTAGGTC

'R_Abg_2' ATCATTATGCGAAGAGCGAACTTTTTTT-TTCTTCTTTACTGTAGGTC

'AB_m1_A' ATCATTATGCGAAGAGCGAACTTTTTTT-TTCTTCTTTACTGTAGGTC

'AB_m1_B' ATCATTATGCGAAGAGCGAACTTTTTTT-TTCTTCTTTACTGTAGGTC

Abh ATCATTATGCGAAGAGCGAACTTTT???-?TCTTCTT?ACTGTAGGTC

'Abh_2' ATCATTATGCGAAGAGCGAACTTTT???-?TCTTCTT?ACTGTAGGTC

'AB_m2_A' ATCATTATGCGAAGAGCGAACTTTTTTT-TTCTTCTTTACTGTAGGTC

'AB_m2_B' ATCATTATGCGAAGAGCGAACTTTTTTT-TTCTTCTTTACTGTAGGTC

'IP_m8_A' ATCATTATGCGAAGAGCGAACTTTTTTT-TTCTTCTTTACTGTAGGTC

'IP_m8_B' ATCATTATGCGAAGAGCGAACTTTTTTT-TTCTTCTTTACTGTAGGTC

'IP_f3_A' ATCATTATGCGAAGAGCGAACTTTTTTT-TTCTTCTTTACTGTAGGTC

'IP_f3_B' ATCATTATGCGAAGAGCGAACTTTTTTT-TTCTTCTTTACTGTAGGTC

'IP_f23_A' ATCATTATGCGAAGAGCGAACTTTTTTT-TTCTTCTTTACTGTAGGTC

'IP_f23_B' ATCATTATGCGAAGAGCGAACTTTTTTT-TTCTTCTTTACTGTAGGTC

'IP_m7_A' ATCATTATGCGAAGAGCGAACTTTTTTT-TTCTTCTTTACTGTAGGTC

'IP_m7_B' ATCATTATGCGAAGAGCGAACTTTTTTT-TTCTTCTTTACTGTAGGTC

'PVL_f14_A' ATCATTATGCGAAGAGCGAACTTTTTTT-TTCTTCTTTACCAAAGATC

'PVL_f14_B' ATCATTATGCGAAGAGCGAACTTTTTTT-TTCTTCTTTACTGTAGGTC

'PVL_f12_A' ATCATTATGCGAAGAGCGAACTTTTTTT-TTCTTCTTTAGTGTAGGTC

'PVL_f12_B' ATCATTATGCGAAGAGCGAACTTTTTTT-TTCTTCTTTAGTGTAGGTC

'PVL_f3_A' ATCATTATGCGAAGAGCGAACTTTTTTT-TTCTTCTTTACTGTAGGTC

'PVL_f3_B' ATCATTATGCGAAGAGCGAACTTTTTTT-TTCTTCTTTACCAAAGATC

'PVL_f7_A' ATCATTATGCGAAGAGCGAACTTTTTTT-TTCTTCTTTACCAAAGATC

'PVL_f7_B' ATCATTATGCGAAGAGCGAACTTTTTTT-TTCTTCTTTACCAAAGATC

'Pes_m9_A' ATCATTATGCGAAAAACAAAGTTTTTT---TCTTCTTTACTGAAGGTC

'Pes_m9_B' ATCATTATGCGAAAAACAAAGTTTTTT---TCTTCTTTACTGAAGGTC

'Pes_m10_A' ATCATTATGCGAAAAACAAAGTTTTTT---TCTTCTTTACTGAAGGTC

'Pes_m10_B' ATCATTATGCGAAAAACAAAGTTTTTT---TCTTCTTTACTGAAGGTC

'LH_m1_A' ATCATTATGCGAAAAACAAACTTTTTTT-CTCTTCTTTACTGAAGGTC

'LH_m1_B' ATCATTATGCGAAAAACAAACTTTTTTT-CTCTTCTTTACTGAAGGTC

'LH_f6_A' ATCATTATGCGAAAAACAAACTTTTTTT-CTCTTCTTTACTGAAGGTC

'LH_f6_B' ATCATTATGCGAAAAACAAACTTTTTTT-CTCTTCTTTACTGAAGGTC

'LH_f8_A' ATCATTATGCGAAAAACAAACTTTTTTT-CTCTTCTTTACTGAAGGTC

'LH_f8_B' ATCATTATGCGAAAAACAAACTTTTTTT-CTCTTCTTTACTGAAGGTC

'scn_h_a' ATCATTATGCGAAAAACAAACTTTTTTT-CTCTTCTTTACTGAAGGTC

'scn_h_b' ATCATTATGCGAAAAACAAACTTTTTTT-CTCTTCTTTACTGAAGGTC

'scn_m5_A' ATCATTATGCGAAAAACAAACTTTTTTT-CTCTTCTTTACTGAAGGTC

'scn_m5_B' ATCATTATGCGAAAAACAAACTTTTTTT-CTCTTCTTTACTGAAGGTC

'scn_m7_A' ATCATTATGCGAAAAACAAACTTTTTTT-CTCTTCTTTACTGAAGGTC

'scn_m7_B' ATCATTATGCGAAAAACAAACTTTTTTT-CTCTTCTTTACTGAAGGTC

'scn_m3_A' ATCATTATGCGAAAAACAAACTTTTTTT-CTCTTCTTTACTGAAGGTC

'scn_m3_B' ATCATTATGCGAAAAACAAACTTTTTTT-CTCTTCTTTACTGAAGGTC

'LJS_m1_A' ATCATTATGCGGAGAACGAACTTTTTTTCTTCTTCTTTACTAAAGGTC

'LJS_m1_B' ATCATTATGCGGAGACCGAACTTTTTTTCTTCTGCTTTACTAAAGGTC

'LJS_m2_A' ATCATTATGCGGAGAAAGAACTTTTTTTCTTCTGCTTTACTAAAGGTC

'LJS_m2_B' ATCATTATGCGGAGAAAGAACTTTTTTTCTTCTTCTTTACTAAAGGTC

'LJS_f10_A' ATCATTATGCGGAGAAAGAACTTTTTTTCTTCTTCTTTACTAAAGGTC

'LJS_f10_B' ATCATTATGCGGAGAAAGAACTTTTTTTCTTCTTCTTTACTAAAGGTC

'LJS_f9_B' ATCATTATGCGGAGAAAGAACTTTTTTTCTTCTTCTTTACTAAAGGTC

'LJS_f9_A' ATCATTATGCGGAGAAAGAACTTTTTTTCTTCTTCTTTACTAAAGGTC

'LJP_m7_B' ATCATTATGCGGAGAAAGAACTTTTTTTCTTCTTCTTTACTAAAGGTC

'LJP_m7_A' ATCATTATGCGGAGAAAGAACTTTTTTTCTTCTTCTTTACTAAAGGTC

'LJP_m6_B' ATCATTATGCGGAGAACGAACTTTTTTTCTTCTTCTTTACTAAAGGTC

'LJP_m6_A' ATCATTATGCGGAGAAAGAACTTTTTTTCTTCTTCTTTACTAAAGGTC

'LJP_m2_B' ATCATTATGCGGAGAAAGAACTTTTTTTCTTCTGCTTTACTGAAGGTC

'LJP_m2_A' ATCATTATGCGGAGAAAGAACTTTTTTTCTTCTTCTTTACTAAAGGTC

'LJP_m1_B' ATCATTATGCGGAGAAAGAACTTTTTTTCTTCTTCTTTACTAAAGGTC

'LJP_m1_A' ATCATTATGCGGAGAAAGAACTTTTTTTCTTCTTCTTTACTAAAGGTC

'BR_m5_A' ATCATTATGCGGAGAAAGAACTTTTTTTCTTCTTCTTTACTAAAGGTC

'BR_m5_B' ATCATTATGCGGAGAAAGAACTTTTTTTCTTCTTCTTTACTGAAGGTC

'BR_m6_A' ATCATTATGCGGAGAACGAACTTTTTTTCTTCTGCTTTACTAAAGGTC

'BR_m6_B' ATCATTATGCGGAGAACGAACTTTTTTTCTTCTTCTTTACTAAAGGTC

'BR_m7_A' ATCATTATGCGGAGAACGAACTTTTTTTCTTCTTCTTTACTAAAGGTC

'BR_m7_B' ATCATTATGCGGAGAACGAACTTTTTTTCTTCTTCTTTACTAAAGGTC

'BR_f13_B' ATCATTATGCGGAGAACGAACTTTTTTTCTTCTGCTTTACTGAAGGTC

'BR_f13_A' ATCATTATGCGGAGAACGAACTTTTTTTCTTCTTCTTTACTAAAGGTC

'BR_m8_B' ATCATTATGCGGAGAACGAACTTTTTTTCTTCTGCTTTACTAAAGGTC

'BR_m8_A' ATCATTATGCGGAGAACGAACTTTTTTTCTTCTGCTTTACTAAAGGTC

'SD_h_a' ATCATTATGCGGAGAACGAACTTTTTTTCTTCTGCTTTACTAAAGGTC

'SD_h_b' ATCATTATGCGGAGAACGAACTTTTTTTCTTCTGCTTTACTAAAGGTC

'SD_g_a' ATCATTATGCGGAGAACGAACTTTTTTTCTTCTGCTTTACTAAAGGTC

'SD_g_b' ATCATTATGCGGAGAACGAACTTTTTTTCTTCTGCTTTACTAAAGGTC

'SD_2m_b' ATCATTATGCGGAGAACGAACTTTTTTTCTTCTGCTTTACTAAAGGTC

'SD_2m_a' ATCATTATGCGGAGAACGAACTTTTTTTCTTCTGCTTTACTAAAGGTC

[ 440 450 460 470 480]

[ * * * * *]

'RP1_m1_A' AGAGAATGAATGGCAGGCTGGCTCCTGAATGCAT--------------

'RP1_m1_B' AGAGAATGAATGGCAGGCTGGCTCCTGAATGCAT--------------

'RP1_m5(A)' AGAGAATGAATGGCAGGCTGGCTCCTGAATGCAT--------------

'RP1_m5_B' AGAGAATGAATGGCAGGCTGGCTCCTGAATGCAT--------------

'RP1_m7_A' AGAGAATGAATGGCAGGCTGGCTCCTGAATGCAT--------------

'RP1_m7_B' AGAGAATGAATGGCAGGCTGGCTCCTGAATGCAT--------------

'RP1_m8_A' AGAGAATGAATGGCAGGCTGGCTCCTGAATGCAT--------------

'RP1_m8_B' AGAGAATGAATGGCAGGCTGGCTCCTGAATGCAT--------------

'R_Abg' AGAGAATGAATGGCAGGCTGGCTCCTGAATGCAT--------------

'R_Abg_2' AGAGAATGAATGGCAGGCTGGCTCCTGAATGCAT--------------

'AB_m1_A' AGAGAATGAATGGCAGGCTGGCTCCTGAATGCAT--------------

'AB_m1_B' AGAGAATGAATGGCAGGCTGGCTCCTGAATGCAT--------------

Abh AGAGAATGAATGGCAGGCTGGCTCCTGAATGCAT--------------

'Abh_2' AGAGAATGAATGGCAGGCTGGCTCCTGAATGCAT--------------

'AB_m2_A' AGAGAATGAATGGCAGGCTGGCTCCTGAATGCAT--------------

'AB_m2_B' AGAGAATGAATGGCAGGCTGGCTCCTGAATGCAT--------------

'IP_m8_A' AGAGAATGAATGGCAGGCTGGCTCCTGAATGCAT--------------

'IP_m8_B' AGAGAATGAATGGCAGGCTGGCTCCTGAATGCAT--------------

'IP_f3_A' AGAGAATGAATGGCAGGCTGGCTCCTGAATGCAT--------------

'IP_f3_B' AGAGAATGAATGGCAGGCTGGCTCCTGAATGCAT--------------

'IP_f23_A' AGAGAATGAATGGCAGGCTGGCTCCTGAATGCAT--------------

'IP_f23_B' AGAGAATGAATGGCAGGCTGGCTCCTGAATGCAT--------------

'IP_m7_A' AGAGAATGAATGGCAGGCTGGCTCCTGAATGCAT--------------

'IP_m7_B' AGAGAATGAATGGCAGGCTGGCTCCTGAATGCAT--------------

'PVL_f14_A' AGAGAATGAATGGCAGGCTGGCTCCTGAATGCAT--------------

'PVL_f14_B' AGAGAATGAATGGCAGGCTGGCTCCTGAATGCAT--------------

'PVL_f12_A' AGAGAATGAATGGCAGGCTGGCTCCTGAATGCAT--------------

'PVL_f12_B' AGAGAATGAATGGCAGGCTGGCTCCTGAATGCAT--------------

'PVL_f3_A' AGAGAATGAATGGCAGGCTGGCTCCTGAATGCAT--------------

'PVL_f3_B' AGAGAATGAATGGCAGGCTGGCTCCTGAATGCAT--------------

'PVL_f7_A' AGAGAATGAATGGCAGGCTGGCTCCTGAATGCAT--------------

'PVL_f7_B' AGAGAATGAATGGCAGGCTGGCTCCTGAATGCAT--------------

'Pes_m9_A' AAAAAATGAATGGCAGGCTGGCTGCTGAATGCAATTATTGAAAATCTT

'Pes_m9_B' AAAAAATGAATGGCAGGCTGGCTCCTGAATGCAATTAATGAAAATCGT

'Pes_m10_A' AAAAAATGAATGGCAGGCTGGCTGCTGAATGCAATTATTGAAAATCTT

'Pes_m10_B' AAAAAATGAATGGCAGGCTGGCTGCTGAATGCAATTATTGAAAATCTT

'LH_m1_A' AAAAAATGAATGGCAGGCTGGCTCCTGAATGCAATTAATGAAAATCGT

'LH_m1_B' AAAAAATGAATGGCAGGCTGGCTCCTGAATGCAATTAATGAAAATCGT

'LH_f6_A' AAAAAATGAATGGCAGGCTGGCTCCTGAATGCAATTAATGAAAATCGT

'LH_f6_B' AAAAAATGAATGGCAGGCTGGCTCCTGAATGCAATTAATGAAAATCGT

'LH_f8_A' AAAAAATGAATGGCAGGCTGGCTCCTGAATGCAATTAATGAAAATCGT

'LH_f8_B' AAAAAATGAATGGCAGGCTGGCTCCTGAATGCAATTAATGAAAATCGT

'scn_h_a' AAAAAATGAATGGCAGGCTGGCTCCTGAATGCAATTAATGAAAATCGT

'scn_h_b' AAAAAATGAATGGCAGGCTGGCTCCTGAATGCAATTAATGAAAATCGT

'scn_m5_A' AAAAAATGAATGGCAGGCTGGCTCCTGAATGCAATTAATGAAAATCGT

'scn_m5_B' AAAAAATGAATGGCAGGCTGGCTCCTGAATGCAATTAATGAAAATCGT

'scn_m7_A' AAAAAATGAATGGCAGGCTGGCTCCTGAATGCAATTAATGAAAATCGT

'scn_m7_B' AAAAAATGAATGGCAGGCTGGCTCCTGAATGCAATTAATGAAAATCGT

'scn_m3_A' AAAAAATGAATGGCAGGCTGGCTCCTGAATGCAATTAATGAAAATCGT

'scn_m3_B' AAAAAATGAATGGCAGGCTGGCTCCTGAATGCAATTAATGAAAATCGT

'LJS_m1_A' AGAGAATGAATGGCAGGCTGGCTCCTGAATGCAT--------------

'LJS_m1_B' AGAGAATGAATGGCAGGCTGGCTCCTGAATGCAT--------------

'LJS_m2_A' AGAGAATGAATGGCAGGCTGGCTCCTGAATGCAT--------------

'LJS_m2_B' AGAGAATGAATGGCAGGCTGGCTCCTGAATGCAT--------------

'LJS_f10_A' AGAGAATGAATGGCAGGCTGGCTCCTGAATGCAT--------------

'LJS_f10_B' AGAGAATGAATGGCAGGCTGGCTCCTGAATGCAT--------------

'LJS_f9_B' AGAGAATGAATGGCAGGCTGGCTCCTGAATGCAT--------------

'LJS_f9_A' AGAGAATGAATGGCAGGCTGGCTCCTGAATGCAT--------------

'LJP_m7_B' AGAGAATGAATGGCAGGCTGGCTCCTGAATGCAT--------------

'LJP_m7_A' AGAGAATGAATGGCAGGCTGGCTCCTGAATGCAT--------------

'LJP_m6_B' AGAGAATGAATGGCAGGCTGGCTCCTGAATGCAT--------------

'LJP_m6_A' AGAGAATGAATGGCAGGCTGGCTCCTGAATGCAT--------------

'LJP_m2_B' AGAGAATGAATGACAGGCTGGCTCCTGAATGCAT--------------

'LJP_m2_A' AGAGAATGAATGGCAGGCTGGCTCCTGAATGCAT--------------

'LJP_m1_B' AGAGAATGAATGGCAGGCTGGCTCCTGAATGCAT--------------

'LJP_m1_A' AGAGAATGAATGGCAGGCTGGCTCCTGAATGCAT--------------

'BR_m5_A' AGAGAATGAATGGCAGGCTGGCTCCTGAATGCAT--------------

'BR_m5_B' AGAGAATGAATGACAGGCTGGCTCCTGAATGCAT--------------

'BR_m6_A' AGAGAATGAATGGCAGGCTGGCTCCTGAATGCAT--------------

'BR_m6_B' AGAGAATGAATGGCAGGCTGGCTCCTGAATGCAT--------------

'BR_m7_A' AGAGAATGAATGGCAGGCTGGCTCCTGAATGCAT--------------

'BR_m7_B' AGAGAATGAATGGCAGGCTGGCTCCTGAATGCAT--------------

'BR_f13_B' AGAGAATGAATGACAGGCTGGCTCCTGAATGAAT--------------

'BR_f13_A' AGAGAATGAATGGCAGGCTGGCTCCTGAATGCAT--------------

'BR_m8_B' AGAGAATGAATGGCAGGCTGGCTCCTGAATGCAT--------------

'BR_m8_A' AGAGAATGAATGGCAGGCTGGCTCCTGAATGCAT--------------

'SD_h_a' AGAGAATGAATGACAGGCTGGCTCCTGAATGCAT--------------

'SD_h_b' AGAGAATGAATGACAGGCTGGCTCCTGAATGCAT--------------

'SD_g_a' AGAGAATGAATGACAGGCTGGCTCCTGAATGCAT--------------

'SD_g_b' AGAGAATGAATGACAGGCTGGCTCCTGAATGCAT--------------

'SD_2m_b' AGAGAATGAATGACAGGCTGGCTCCTGAATGCAT--------------

'SD_2m_a' AGAGAATGAATGACAGGCTGGCTCCTGAATGCAT--------------

[ 490 500 510 520 ]

[ * * * * ]

'RP1_m1_A' ------------AAATTTATCCAGGCTGGTGAATTTAGGC--------

'RP1_m1_B' ------------AAATTTATCCAGGCTGGTGAATTTAGGC--------

'RP1_m5(A)' ------------AAATTTATCCAGGCTGGTGAATTTAGGC--------

'RP1_m5_B' ------------AAATTTATCCAGGCTGGTGAATTTAGGC--------

'RP1_m7_A' ------------AAATTTATCCAGGCTGGTGAATTTAGGC--------

'RP1_m7_B' ------------AAATTTATCCAGGCTGGTGAATTTAGGC--------

'RP1_m8_A' ------------AAATTTATCCAGGCTGGTGAATTTAGGC--------

'RP1_m8_B' ------------AAATTTATCCAGGCTGGTGAATTTAGGC--------

'R_Abg' ------------AAATTTATCCAGGCTGGTGAATTTAGGC--------

'R_Abg_2' ------------AAATTTATCCAGGCTGGTGAATTTAGGC--------

'AB_m1_A' ------------AAATTTATCCAGGCTGGTGAATTTAGGC--------

'AB_m1_B' ------------AAATTTATCCAGGCTGGTGAATTTAGGC--------

Abh ------------AAATTTATCCAGGCTGGTGAATTTAGGC--------

'Abh_2' ------------AAATTTATCCAGGCTGGTGAATTTAGGC--------

'AB_m2_A' ------------AAATTTATCCAGGCTGGTGAATTTAGGC--------

'AB_m2_B' ------------AAATTTATCCAGGCTGGTGAATTTAGGC--------

'IP_m8_A' ------------AAATTTATCCAGGCTGGTGAATTTAGGC--------

'IP_m8_B' ------------AAATTTATCCAGGCTGGTGAATTTAGGC--------

'IP_f3_A' ------------AAATTTATCCAGGCTGGTGAATTTAGGC--------

'IP_f3_B' ------------AAATTTATCCAGGCTGGTGAATTTAGGC--------

'IP_f23_A' ------------AAATTTATCCAGGCTGGTGAATTTAGGC--------

'IP_f23_B' ------------AAATTTATCCAGGCTGGTGAATTTAGGC--------

'IP_m7_A' ------------AAATTTATCCAGGCTGGTGAATTTAGGC--------

'IP_m7_B' ------------AAATTTATCCAGGCTGGTGAATTTAGGC--------

'PVL_f14_A' ------------AAATTTATCCAGGCTGGTGAATTTAGGC--------

'PVL_f14_B' ------------AAATTTATCCAGGCTGGTGAATTTAGGC--------

'PVL_f12_A' ------------AAATTTATCCAGGCTGGTGAATTTAGGC--------

'PVL_f12_B' ------------AAATTTATCCAGGCTGGTGAATTTAGGC--------

'PVL_f3_A' ------------AAATTTATCCAGGCTGGTGAATTTAGGC--------

'PVL_f3_B' ------------AAATTTATCCAGGCTGGTGAATTTAGGC--------

'PVL_f7_A' ------------AAATTTATCCAGGCTGGTGAATTTAGGC--------

'PVL_f7_B' ------------AAATTTATCCAGGCTGGTGAATTTAGGC--------

'Pes_m9_A' TGCTTATAATAAAAATATACCCAGGCTGGTGAATTAAG---TAT----

'Pes_m9_B' TGCTTAAAATAAAAATATACCCAGGCTGGTGAATTAAA---TAT----

'Pes_m10_A' TGCTTATAATAAAAATATACCCAGGCTGGTGAATTAAG---TAT----

'Pes_m10_B' TGCTTATAATAAAAATATACCCAGGCTGGTGAATTAAG---TAT----

'LH_m1_A' TGCTTAAAATAAAAATATACCCAGGCTGGTGAATTAAA---TAT----

'LH_m1_B' TGCTTAAAATAAAAATATACCCAGGCTGGTGAATTAAA---TAT----

'LH_f6_A' TGCTTAAAATAAAAATATACCCAGGCTGGTGAATTAAA---TAT----

'LH_f6_B' TGCTTAAAATAAAAATATACCCAGGCTGGTGAATTAAA---TAT----

'LH_f8_A' TGCTTAAAATAAAAATATACCCAGGCTGGTGAATTAAA---TAT----

'LH_f8_B' TGCTTAAAATAAAAATATACCCAGGCTGGTGAATTAAA---TAT----

'scn_h_a' TGCTTAAAATAAAAATATACCCAGGCTGGTGAATTAAA---TAT----

'scn_h_b' TGCTTAAAATAAAAATATACCCAGGCTGGTGAATTAAA---TAT----

'scn_m5_A' TGCTTAAAATAAAAATATACCCAGGCTGGTGAATTAAA---TAT----

'scn_m5_B' TGCTTAAAATAAAAATATACCCAGGCTGGTGAATTAAA---TAT----

'scn_m7_A' TGCTTAAAATAAAAATATACCCAGGCTGGTGAATTAAA---TAT----

'scn_m7_B' TGCTTAAAATAAAAATATACCCAGGCTGGTGAATTAAA---TAT----

'scn_m3_A' TGCTTAAAATAAAAATATACCCAGGCTGGTGAATTAAA---TAT----

'scn_m3_B' TGCTTAAAATAAAAATATACCCAGGCTGGTGAATTAAA---TAT----

'LJS_m1_A' ------------AAATTTATCCAGGCTGGTGAATTTAGTAGTAT----

'LJS_m1_B' ------------AAATTTATCCAGGCTGGTGAATTTAGTAGTAT----

'LJS_m2_A' ------------AAATTTATCCAGGCTGGTGAATTTAGTAGTAT----

'LJS_m2_B' ------------AAATTTATCCAGGCTGGTGAATTTAGTAGTAT----

'LJS_f10_A' ------------AAATTTATCCAGGCTGGTGAATTTAGTAGTAT----

'LJS_f10_B' ------------AAATTTATCCAGGCTGGTGAATTTAGTAGTAT----

'LJS_f9_B' ------------AAATTTATCCAGGCTGGTGAATTTAGTAGTAT----

'LJS_f9_A' ------------AAATTTATCCAGGCTGGTGAATTTAGTAGTAT----

'LJP_m7_B' ------------AAATTTATCCAGGCTGGTGAATTTAGTAGTAT----

'LJP_m7_A' ------------AAATTTATCCAGGCTGGTGAATTTAGTAGTAT----

'LJP_m6_B' --------AAATAAA-----------TGGTGAATTTAGTAGTATCATA

'LJP_m6_A' ------------AAATTTATCCAGGCTGGTGAATTTAGTAGTAT----

'LJP_m2_B' --------AAATAAA-----------TGGTGAATTTAGTAGTAT----

'LJP_m2_A' ------------AAATTTATCCAGGCTGGTGAATTTAGTAGTAT----

'LJP_m1_B' ------------AAATTTATCCAGGCTGGTGAATTTAGTAGTAT----

'LJP_m1_A' ------------AAATTTATCCAGGCTGGTGAATTTAGTAGTAT----

'BR_m5_A' ------------AAATTTATCCAGGCTGGTGAATTTAGTAGTAT----

'BR_m5_B' --------AAATAAA-----------TGGTGAATTTAGTAGTAT----

'BR_m6_A' ------------AAATTTATCCAGGCTGGTGAATTTAGTAGTAT----

'BR_m6_B' ------------AAATTTATCCAGGCTGGTGAATTTAGTAGTAT----

'BR_m7_A' ------------AAATTTATCCAGGCTGGTGAATTTAGTAGTAT----

'BR_m7_B' ------------AAATTTATCCAGGCTGGTGAATTTAGTAGTAT----

'BR_f13_B' --------AAATAAA-----------TGGTGAATTTAGTAGTAT----

'BR_f13_A' ------------AAATTTATCCAGGCTGGTGAATTTAGTAGTAT----

'BR_m8_B' ------------AAATTTATCCAGGCTGGTGAATTTAGTAGTAT----

'BR_m8_A' ------------AAATTTATCCAGGCTGGTGAATTTAGTAGTAT----

'SD_h_a' ------------AAATTTATCCAGGCTGGTGAATTTAGTAGTAT----

'SD_h_b' ------------AAATTTATCCAGGCTGGTGAATTTAGTAGTAT----

'SD_g_a' ------------AAATTTATCCAGGCTGGTGAATTTAGTAGTAT----

'SD_g_b' ------------AAATTTATCCAGGCTGGTGAATTTAGTAGTAT----

'SD_2m_b' ------------AAATTTATCCAGGCTGGTGAATTTAGTAGTAT----

'SD_2m_a' ------------AAATTTATCCAGGCTGGTGAATTTAGTAGTAT----

[ 530 540 550 560 570 ]

[ * * * * * ]

'RP1_m1_A' ------------------------------------------------

'RP1_m1_B' ------------------------------------------------

'RP1_m5(A)' ------------------------------------------------

'RP1_m5_B' ------------------------------------------------

'RP1_m7_A' ------------------------------------------------

'RP1_m7_B' ------------------------------------------------

'RP1_m8_A' ------------------------------------------------

'RP1_m8_B' ------------------------------------------------

'R_Abg' ------------------------------------------------

'R_Abg_2' ------------------------------------------------

'AB_m1_A' ------------------------------------------------

'AB_m1_B' ------------------------------------------------

Abh ------------------------------------------------

'Abh_2' ------------------------------------------------

'AB_m2_A' ------------------------------------------------

'AB_m2_B' ------------------------------------------------

'IP_m8_A' ------------------------------------------------

'IP_m8_B' ------------------------------------------------

'IP_f3_A' ------------------------------------------------

'IP_f3_B' ------------------------------------------------

'IP_f23_A' ------------------------------------------------

'IP_f23_B' ------------------------------------------------

'IP_m7_A' ------------------------------------------------

'IP_m7_B' ------------------------------------------------

'PVL_f14_A' ------------------------------------------------

'PVL_f14_B' ------------------------------------------------

'PVL_f12_A' ------------------------------------------------

'PVL_f12_B' ------------------------------------------------

'PVL_f3_A' ------------------------------------------------

'PVL_f3_B' ------------------------------------------------

'PVL_f7_A' ------------------------------------------------

'PVL_f7_B' ------------------------------------------------

'Pes_m9_A' -----CACATCGTTTTCTCCATCCCATAAAATATGACACTGAGTGGGT

'Pes_m9_B' -----CACATCGTTTTCTCCATCCCATAAAATATGACACTGAGTGGGT

'Pes_m10_A' -----CACATCGTTTTCTCCATCCCATAAAATATGACACTGAGTGGGT

'Pes_m10_B' -----CACATCGTTTTCTCCATCCCATAAAATATGACACTGAGTGGGT

'LH_m1_A' -----CACATCGTTTTCTCCATCCCATAAAATATGACACTGAGTGGGT

'LH_m1_B' -----CACATCGTTTTCTCCATCCCATAAAATATGACACTGAGTGGGT

'LH_f6_A' -----CACATCGTTTTCTCCATCCCATAAAATATGACACTGAGTGGGT

'LH_f6_B' -----CACATCGTTTTCTCCATCCCATAAAATATGACACTGAGTGGGT

'LH_f8_A' -----CACATCGTTTTCTCCATCCCATAAAATATGACACTGAGTGGGT

'LH_f8_B' -----CACATCGTTTTCTCCATCCCATAAAATATGACACTGAGTGGGT

'scn_h_a' -----CACATCGTTTTCTCCATCCCATAAAATATGACACTGAGTGGGT

'scn_h_b' -----CACATCGTTTTCTCCATCCCATAAAATATGACACTGAGTGGGT

'scn_m5_A' -----CACATCGTTTTCTCCATCCCATAAAATATGACACTGAGTGGGT

'scn_m5_B' -----CACATCGTTTTCTCCATCCCATAAAATATGACACTGAGTGGGT

'scn_m7_A' -----CACATCGTTTTCTCCATCCCATAAAATATGACACTGAGTGGGT

'scn_m7_B' -----CACATCGTTTTCTCCATCCCATAAAATATGACACTGAGTGGGT

'scn_m3_A' -----CACATCGTTTTCTCCATCCCATAAAATATGACACTGAGTGGGT

'scn_m3_B' -----CACATCGTTTTCTCCATCCCATAAAATATGACACTGAGTGGGT

'LJS_m1_A' -----CATAAGGTTTTCTCTTTCCCATAAAATATGACACTGAGTGGGG

'LJS_m1_B' -----CATTAGGTTTTCTCTTTCCCATAAAATATGACACTGAGTGGGG

'LJS_m2_A' -----CATAAGGTTTTCTCTTTCCCATAAAATATGACACTGAGTGGGG

'LJS_m2_B' -----CATAAGGTTTTCTCTTTCCCATAAAATATGACACTGAGTGGGG

'LJS_f10_A' -----CATATGGTTTTCTCTTTCCCATAAAATATGACACTGAGTGGGG

'LJS_f10_B' -----CATATGGTTTTCTCTTTCCCATAAAATATGACACTGAGTGCGG

'LJS_f9_B' -----CATAAGGTTTTCTCTTTCCCATAAAATATGACACTGAGTGGGG

'LJS_f9_A' -----CATATGGTTTTCTCTTTCCCATAAAATATGACACTGAGTGCGG

'LJP_m7_B' -----CATATGGTTTTCTCTTTCCCATAAAATATGACACTGAGTGGGG

'LJP_m7_A' -----CATAAGGTTTTCTCTTTCCCATAAAATATGACACTGAGTGGGG

'LJP_m6_B' GTATGCATAAGGTTTTCTCTTTCCCATAAAATATGACACTGAGTGGGG

'LJP_m6_A' -----CATAAGGTTTTCTCTTTCCCATAAAATATGACACTGAGTGGGG

'LJP_m2_B' -----CATAAGGTTTTCTCTTTCCCATAAAATATGACACTGAGTGGGG

'LJP_m2_A' -----CATAAGGTTTTCTCTTTCCCATAAAATATGACACTGAGTGGGG

'LJP_m1_B' -----CATAAGGTTTTCTCTTTCCCATAAAATATGACACTGAGTGGGG

'LJP_m1_A' -----CATAAGGTTTTCTCTTTCCCATAAAATATGACACTGAGTGGGG

'BR_m5_A' -----CATAAGGTTTTCTCTTTCCCATAAAATATGACACTGAGTGGGG

'BR_m5_B' -----CATAAGGTTTTCTCTTTCCCATAAAATATGACACTGAGTGGGG

'BR_m6_A' -----CATATGGTTTTCTCTTTCCCATAAAATATGACACTGAGTGGGG

'BR_m6_B' -----CATATGGTTTTCTCTTTCCCATAAAATATGACACTGAGTGGGG

'BR_m7_A' -----CATATGGTTTTCTCTTTCCCATAAAATATGACACTGAGTGGGG

'BR_m7_B' -----CATATGGTTTTCTCTTTCCCATAAAATATGACACTGAGTGGGG

'BR_f13_B' -----CATATGGTTTTCTCTTTCCCATAAAATATGACACTGAGTGGGG

'BR_f13_A' -----CATATGGTTTTCTCTTTCCCATAAAATATGACACTGAGTGGGG

'BR_m8_B' -----CATATGGTTTTCTCTTTCCCATAAAATATGACACTGAGTGGGG

'BR_m8_A' -----CATATGGTTTTCTCTTTCCCATAAAATATGACACTGAGTGGGG

'SD_h_a' -----CATATGGTTTTCTCTTTCCCATAAACTATGACACTGAGTGGGG

'SD_h_b' -----CATATGGTTTTCTCTTTCCCATAAACTATGACACTGAGTGCGG

'SD_g_a' -----CATATGGTTTTCTCTTTCCCATAAACTATGACACTGAGTGGGG

'SD_g_b' -----CATATGGTTTTCTCTTTCCCATAAACTATGACACTGAGTGCGG

'SD_2m_b' -----CATATGGTTTTCTCTTTCCCATAAACTATGACACTGAGTGGGG

'SD_2m_a' -----CATATGGTTTTCTCTTTCCCATAAACTATGACACTGAGTGGGG

[ 580 590 600 610 620 ]

[ * * * * * ]

'RP1_m1_A' ------------------------------------------------

'RP1_m1_B' ------------------------------------------------

'RP1_m5(A)' ------------------------------------------------

'RP1_m5_B' ------------------------------------------------

'RP1_m7_A' ------------------------------------------------

'RP1_m7_B' ------------------------------------------------

'RP1_m8_A' ------------------------------------------------

'RP1_m8_B' ------------------------------------------------

'R_Abg' ------------------------------------------------

'R_Abg_2' ------------------------------------------------

'AB_m1_A' ------------------------------------------------

'AB_m1_B' ------------------------------------------------

Abh ------------------------------------------------

'Abh_2' ------------------------------------------------

'AB_m2_A' ------------------------------------------------

'AB_m2_B' ------------------------------------------------

'IP_m8_A' ------------------------------------------------

'IP_m8_B' ------------------------------------------------

'IP_f3_A' ------------------------------------------------

'IP_f3_B' ------------------------------------------------

'IP_f23_A' ------------------------------------------------

'IP_f23_B' ------------------------------------------------

'IP_m7_A' ------------------------------------------------

'IP_m7_B' ------------------------------------------------

'PVL_f14_A' ------------------------------------------------

'PVL_f14_B' ------------------------------------------------

'PVL_f12_A' ------------------------------------------------

'PVL_f12_B' ------------------------------------------------

'PVL_f3_A' ------------------------------------------------

'PVL_f3_B' ------------------------------------------------

'PVL_f7_A' ------------------------------------------------

'PVL_f7_B' ------------------------------------------------

'Pes_m9_A' CATTTTCCACTTATTTGTAGGCACTTGTACAATC--------------

'Pes_m9_B' CATTTTCCACTTATCTGTAGGCACTTGTACAATCTAAAAACTTGAAAT

'Pes_m10_A' CATTTTCCACTTATTTGTAGGCACTTGTACAATC--------------

'Pes_m10_B' CATTTTCCACTTATTTGTAGGCACTTGTACAATC--------------

'LH_m1_A' CATTTTCCACTTATCTGTAGGCACTTGTACAATCTAAAAACTTGAAAT

'LH_m1_B' CATTTTCCACTTATCTGTAGGCACTTGTACAATCTAAAAACTTGAAAT

'LH_f6_A' CATTTTCCACTTATCTGTAGGCACTTGTACAATCTAAAAACTTGAAAT

'LH_f6_B' CATTTTCCACTTATCTGTAGGCACTTGTACAATCTAAAAACTTGAAAT

'LH_f8_A' CATTTTCCACTTATCTGTAGGCACTTGTACAATCTAAAAACTTGAAAT

'LH_f8_B' CATTTTCCACTTATCTGTAGGCACTTGTACAATCTAAAAACTTGAAAT

'scn_h_a' CATTTTCCACTTATCTGTAGGCACTTGTACAATCTAAAAACTTGAAAT

'scn_h_b' CATTTTCCACTTATCTGTAGGCACTTGTACAATCTAAAAACTTGAAAT

'scn_m5_A' CATTTTCCACTTATCTGTAGGCACTTGTACAATCTAAAAACTTGAAAT

'scn_m5_B' CATTTTCCACTTATCTGTAGGCACTTGTACAATCTAAAAACTTGAAAT

'scn_m7_A' CATTTTCCACTTATCTGTAGGCACTTGTACAATCTAAAAACTTGAAAT

'scn_m7_B' CATTTTCCACTTATCTGTAGGCACTTGTACAATCTAAAAACTTGAAAT

'scn_m3_A' CATTTTCCACTTATCTGTAGGCACTTGTACAATCTAAAAACTTGAAAT

'scn_m3_B' CATTTTCCACTTATCTGTAGGCACTTGTACAATCTAAAAACTTGAAAT

'LJS_m1_A' CGTC----------------------------------AACTTCAGAT

'LJS_m1_B' CGTC----------------------------------AACTTCAGAT

'LJS_m2_A' CGTC----------------------------------AACTTCAGAT

'LJS_m2_B' CGTC----------------------------------AACTTCAGAT

'LJS_f10_A' CGTC----------------------------------AACTTCAGAT

'LJS_f10_B' CGTC----------------------------------AACTTCAGAT

'LJS_f9_B' CGTC----------------------------------AACTTCAGAT

'LJS_f9_A' CGTC----------------------------------AACTTCAGAT

'LJP_m7_B' CGTC----------------------------------AACTTCAGAT

'LJP_m7_A' CGTC----------------------------------AACTTCAGAT

'LJP_m6_B' CGTC----------------------------------AACTTCAGAT

'LJP_m6_A' CGTC----------------------------------AACTTCAGAT

'LJP_m2_B' CGTC----------------------------------AACTTCAGAT

'LJP_m2_A' CGTC----------------------------------AACTTCAGAT

'LJP_m1_B' CGTC----------------------------------AACTTCAGAT

'LJP_m1_A' CGTC----------------------------------AACTTCAGAT

'BR_m5_A' CGTC----------------------------------AACTTCAGAT

'BR_m5_B' CGTC----------------------------------AACTTCAGAT

'BR_m6_A' CGTC----------------------------------AACTTCAGAT

'BR_m6_B' CGTC----------------------------------AACTTCAGAT

'BR_m7_A' CGTC----------------------------------AACTTCAGAT

'BR_m7_B' CGTC----------------------------------AACTTCAGAT

'BR_f13_B' CGTC----------------------------------AACTTCAGAT

'BR_f13_A' CGTC----------------------------------AACTTCAGAT

'BR_m8_B' CGTC----------------------------------AACTTCAGAT

'BR_m8_A' CGTC----------------------------------AACTTCAGAT

'SD_h_a' CGTC----------------------------------AACTTCAGAT

'SD_h_b' TGTC----------------------------------AACTTCAGAT

'SD_g_a' CGTC----------------------------------AACTTCAGAT

'SD_g_b' TGTC----------------------------------AACTTCAGAT

'SD_2m_b' CGTC----------------------------------AACTTCAGAT

'SD_2m_a' CGTC----------------------------------AACTTCAGAT

[ 630 640 650 660 670 ]

[ * * * * * ]

'RP1_m1_A' ------------------------------------------------

'RP1_m1_B' ------------------------------------------------

'RP1_m5(A)' ------------------------------------------------

'RP1_m5_B' ------------------------------------------------

'RP1_m7_A' ------------------------------------------------

'RP1_m7_B' ------------------------------------------------

'RP1_m8_A' ------------------------------------------------

'RP1_m8_B' ------------------------------------------------

'R_Abg' ------------------------------------------------

'R_Abg_2' ------------------------------------------------

'AB_m1_A' ------------------------------------------------

'AB_m1_B' ------------------------------------------------

Abh ------------------------------------------------

'Abh_2' ------------------------------------------------

'AB_m2_A' ------------------------------------------------

'AB_m2_B' ------------------------------------------------

'IP_m8_A' ------------------------------------------------

'IP_m8_B' ------------------------------------------------

'IP_f3_A' ------------------------------------------------

'IP_f3_B' ------------------------------------------------

'IP_f23_A' ------------------------------------------------

'IP_f23_B' ------------------------------------------------

'IP_m7_A' ------------------------------------------------

'IP_m7_B' ------------------------------------------------

'PVL_f14_A' ------------------------------------------------

'PVL_f14_B' ------------------------------------------------

'PVL_f12_A' ------------------------------------------------

'PVL_f12_B' ------------------------------------------------

'PVL_f3_A' ------------------------------------------------

'PVL_f3_B' ------------------------------------------------

'PVL_f7_A' ------------------------------------------------

'PVL_f7_B' ------------------------------------------------

'Pes_m9_A' ------------------------------------------------

'Pes_m9_B' GAGAACAATGTGCCTTAAATTTTCTTTTCGAAGCCTTGAAACAATATA

'Pes_m10_A' ------------------------------------------------

'Pes_m10_B' ------------------------------------------------

'LH_m1_A' GAGAACAATGTGCCTTAAATTTTCTTTTCGAAGCCTTGAAACAATATA

'LH_m1_B' GAGAACAATGTGCCTTAAATTTTCTTTTCGAAGCCTTGAAACAATATA

'LH_f6_A' GAGAACAATGTGCCTTAAATTTTCTTTTCGAAGCCTTGAAACAATATA

'LH_f6_B' GAGAACAATGTGCCTTAAATTTTCTTTTCGAAGCCTTGAAACAATATA

'LH_f8_A' GAGAACAATGTGCCTTAAATTTTCTTTTCGAAGCCTTGAAACAATATA

'LH_f8_B' GAGAACAATGTGCCTTAAATTTTCTTTTCGAAGCCTTGAAACAATATA

'scn_h_a' GAGAACAATGTGCCTTAAATTTTCTTTTCGAAGCCTTGAAACAATATA

'scn_h_b' GAGAACAATGTGCCTTAAATTTTCTTTTCGAAGCCTTGAAACAATATA

'scn_m5_A' GAGAACAATGTGCCTTAAATTTTCTTTTCGAAGCCTTGAAACAATATA

'scn_m5_B' GAGAACAATGTGCCTTAAATTTTCTTTTCGAAGCCTTGAAACAATATA

'scn_m7_A' GAGAACAATGTGCCTTAAATTTTCTTTTCGAAGCCTTGAAACAATATA

'scn_m7_B' GAGAACAATGTGCCTTAAATTTTCTTTTCGAAGCCTTGAAACAATATA

'scn_m3_A' GAGAACAATGTGCCTTAAATTTTCTTTTCGAAGCCTTGAAACAATATA

'scn_m3_B' GAGAACAATGTGCCTTAAATTTTCTTTTCGAAGCCTTGAAACAATATA

'LJS_m1_A' ---AATGAGGTGCC----------------------------------

'LJS_m1_B' ---AATGAGGTGCC----------------------------------

'LJS_m2_A' ---AATGAGGTGCC----------------------------------

'LJS_m2_B' ---AATGAGGTGCC----------------------------------

'LJS_f10_A' ---AATGAGGTGCC----------------------------------

'LJS_f10_B' ---AATGAGGTGCC----------------------------------

'LJS_f9_B' ---AATGAGGTGCC----------------------------------

'LJS_f9_A' ---AATGAGGTGCC----------------------------------

'LJP_m7_B' ---AATGAGGTGCC----------------------------------

'LJP_m7_A' ---AATGAGGTGCC----------------------------------

'LJP_m6_B' ---AATGAGGTGCC----------------------------------

'LJP_m6_A' ---AATGAGGTGCC----------------------------------

'LJP_m2_B' ---AATGAGGTGCC----------------------------------

'LJP_m2_A' ---AATGAGGTGCC----------------------------------

'LJP_m1_B' ---AATGAGGTGCC----------------------------------

'LJP_m1_A' ---AATGAGGTGCC----------------------------------

'BR_m5_A' ---AATGAGGTGCC----------------------------------

'BR_m5_B' ---AATGAGGTGCC----------------------------------

'BR_m6_A' ---AATGAGGTGCC----------------------------------

'BR_m6_B' ---AATGAGGTGCC----------------------------------

'BR_m7_A' ---AATGAGGTGCC----------------------------------

'BR_m7_B' ---AATGAGGTGCC----------------------------------

'BR_f13_B' ---AATGAGGTGCC----------------------------------

'BR_f13_A' ---AATGAGGTGCC----------------------------------

'BR_m8_B' ---AATGAGGTGCC----------------------------------

'BR_m8_A' ---AATGAGGTGCC----------------------------------

'SD_h_a' ---AATGAGGTGCC----------------------------------

'SD_h_b' ---AATGAGGTGCC----------------------------------

'SD_g_a' ---AATGAGGTGCC----------------------------------

'SD_g_b' ---AATGAGGTGCC----------------------------------

'SD_2m_b' ---AATGAGGTGCC----------------------------------

'SD_2m_a' ---AATGAGGTGCC----------------------------------

[ 680 690 700 710 720]

[ * * * * *]

'RP1_m1_A' ------------------------------------------------

'RP1_m1_B' ------------------------------------------------

'RP1_m5(A)' ------------------------------------------------

'RP1_m5_B' ------------------------------------------------

'RP1_m7_A' ------------------------------------------------

'RP1_m7_B' ------------------------------------------------

'RP1_m8_A' ------------------------------------------------

'RP1_m8_B' ------------------------------------------------

'R_Abg' ------------------------------------------------

'R_Abg_2' ------------------------------------------------

'AB_m1_A' ------------------------------------------------

'AB_m1_B' ------------------------------------------------

Abh ------------------------------------------------

'Abh_2' ------------------------------------------------

'AB_m2_A' ------------------------------------------------

'AB_m2_B' ------------------------------------------------

'IP_m8_A' ------------------------------------------------

'IP_m8_B' ------------------------------------------------

'IP_f3_A' ------------------------------------------------

'IP_f3_B' ------------------------------------------------

'IP_f23_A' ------------------------------------------------

'IP_f23_B' ------------------------------------------------

'IP_m7_A' ------------------------------------------------

'IP_m7_B' ------------------------------------------------

'PVL_f14_A' ------------------------------------------------

'PVL_f14_B' ------------------------------------------------

'PVL_f12_A' ------------------------------------------------

'PVL_f12_B' ------------------------------------------------

'PVL_f3_A' ------------------------------------------------

'PVL_f3_B' ------------------------------------------------

'PVL_f7_A' ------------------------------------------------

'PVL_f7_B' ------------------------------------------------

'Pes_m9_A' ---------GGTGATTGGTTGGGAATAACGTAGTCAGATTGAAGATCT

'Pes_m9_B' CCACTAACAGCTGATTGGTTGGGAATAACGTAGTCAGATTGAAGATCT

'Pes_m10_A' ---------GGTGATTGGTTGGGAATAACGTAGTCAGATTGAAGATCT

'Pes_m10_B' ---------GGTGATTGGTTGGGAATAACGTAGTCAGATTGAAGATCT

'LH_m1_A' CCACTAACAGCTGATTGGTTGGGAATAACGTAGTCAGATTGAAGATCT

'LH_m1_B' CCACTAACAGCTGATTGGTTGGGAATAACGTAGTCAGATTGAAGATCT

'LH_f6_A' CCACTAACAGCTGATTGGTTGGGAATAACGTAGTCAGATTGAAGATCT

'LH_f6_B' CCACTAACAGCTGATTGGTTGGGAATAACGTAGTCAGATTGAAGATCT

'LH_f8_A' CCACTAACAGCTGATTGGTTGGGAATAACGTAGTCAGATTGAAGATCT

'LH_f8_B' CCACTAACAGCTGATTGGTTGGGAATAACGTAGTCAGATTGAAGATCT

'scn_h_a' CCACTAACAGCTGATTGGTTGGGAATAACGTAGTCAGATTGAAGATCT

'scn_h_b' CCACTAACAGCTGATTGGTTGGGAATAACGTAGTCAGATTGAAGATCT

'scn_m5_A' CCACTAACAGCTGATTGGTTGGGAATAACGTAGTCAGATTGAAGATCT

'scn_m5_B' CCACTAACAGCTGATTGGTTGGGAATAACGTAGTCAGATTGAAGATCT

'scn_m7_A' CCACTAACAGCTGATTGGTTGGGAATAACGTAGTCAGATTGAAGATCT

'scn_m7_B' CCACTAACAGCTGATTGGTTGGGAATAACGTAGTCAGATTGAAGATCT

'scn_m3_A' CCACTAACAGCTGATTGGTTGGGAATAACGTAGTCAGATTGAAGATCT

'scn_m3_B' CCACTAACAGCTGATTGGTTGGGAATAACGTAGTCAGATTGAAGATCT

'LJS_m1_A' --------AGCCGATTGGTTGGGAATTACGTAGTCAGATTGAGGATCT

'LJS_m1_B' --------AGCCGATTGGTTGGGAATTACGTAGTCAGATTGAGGATCT

'LJS_m2_A' --------AGCCGATTGGTTGGGAATTACGTAGTCAGATTGAGGATCT

'LJS_m2_B' --------AGCCGATTGGTTGGGAATAACGTAGTCAGATTGAGGATCT

'LJS_f10_A' --------AGCCGATTGGTTGGGAATTACGTAGTCAGATTGAGGATCT

'LJS_f10_B' --------AGCCGATTGGTTGGGAATTACGTAGTCAGATTGAGGATCT

'LJS_f9_B' ------------------------------------------------

'LJS_f9_A' --------AGCCGATTGGTTGGGAATTACGTAGTCAGATTGAGGATCT

'LJP_m7_B' --------AGCCGATTGGTTGGGAATTACGTAGTCAGATTGAGGATCT

'LJP_m7_A' --------AGCCGATTGGTTGGGAATTACGTAGTCAGATTGAGGATCT

'LJP_m6_B' --------AGCCGATTGGTTGGGAATTACGTAGTCAGATTGAGGATCT

'LJP_m6_A' --------AGCCGATTGGTTGGGAATTACGTAGTCAGATTGAGGATCT

'LJP_m2_B' --------AGCCGATTGGTTGGGAATTACGTAGTCAGATTGAGGATCT

'LJP_m2_A' --------AGCCGATTGGTTGGGAATTACGTAGTCAGATTGAGGATCT

'LJP_m1_B' --------AGCCGATTGGTTGGGAATTACGTAGTCAGATTGAGGATCT

'LJP_m1_A' --------AGCCGATTGGTTGGGAATTACGTAGTCAGATTGAGGATCT

'BR_m5_A' --------AGCCGATTGGTTGGGAATTACGTAGTCAGATTGAGGATCT

'BR_m5_B' --------AGCCGATTGGTTGGGAATTACGTAGTCAGATTGAGGATCT

'BR_m6_A' --------AGCCGATTGGTTGGGAATAACGTAGTCAGATTGAGGATCT

'BR_m6_B' --------AGCCGATTGGTTGGGAATAACGTAGTCAGATTGAGGATCT

'BR_m7_A' --------AGCCGATTGGTTGGGAATAACGTAGTCAGATTGAGGATCT

'BR_m7_B' --------AGCCGATTGGTTGGGAATAACGTAGTCAGATTGAGGATCT

'BR_f13_B' --------AGCCGATTGGTTGGGAATTACGTAGTCAGATTGAGGATCT

'BR_f13_A' --------AGCCGATTGGTTGGGAATAACGTAGTCAGATTGAGGATCT

'BR_m8_B' --------AGCCGATTGGTTGGGAATAACGTAGTCAGATTGAGGATCT

'BR_m8_A' --------AGCCGATTGGTTGGGAATAACGTAGTCAGATTGAGGATCT

'SD_h_a' --------AGCCGATTGGTTGGGAATTACGTAGTCAGATTGAGGATCT

'SD_h_b' --------AGCCGATTGGTTGGGAATTACGTAGTCAGATTGAGGATCT

'SD_g_a' --------AGCCGATTGGTTGGGAATTACGTAGTCAGATTGAGGATCT

'SD_g_b' --------AGCCGATTGGTTGGGAATTACGTAGTCAGATTGAGGATCT

'SD_2m_b' --------AGCCGATTGGTTGGGAATTACGTAGTCAGATTGAGGATCT

'SD_2m_a' --------AGCCGATTGGTTGGGAATTACGTAGTCAGATTGAGGATCT

[ 730 740 750 760 ]

[ * * * * ]

'RP1_m1_A' ---------------------------------------CTCCTCCGA

'RP1_m1_B' ---------------------------------------CTCCTCCGA

'RP1_m5(A)' ---------------------------------------CTCCTCCGA

'RP1_m5_B' ---------------------------------------CTCCTCCGA

'RP1_m7_A' ---------------------------------------CTCCTCCGA

'RP1_m7_B' ---------------------------------------CTCCTCCGA

'RP1_m8_A' ---------------------------------------CTCCTCCGA

'RP1_m8_B' ---------------------------------------CTCCTCCGA

'R_Abg' ---------------------------------------CTCCTCCGA

'R_Abg_2' ---------------------------------------CTCCTCCGA

'AB_m1_A' ---------------------------------------CTCCTCCGA

'AB_m1_B' ---------------------------------------CTCCTCCGA

Abh ---------------------------------------CTCCTCCGA

'Abh_2' ---------------------------------------CTCCTCCGA

'AB_m2_A' ---------------------------------------CTCCTCCGA

'AB_m2_B' ---------------------------------------CTCCTCCGA

'IP_m8_A' ---------------------------------------CTCCTCCGA

'IP_m8_B' ---------------------------------------CTCCTCCGA

'IP_f3_A' ---------------------------------------CTCCTCCGA

'IP_f3_B' ---------------------------------------CTCCTCCGA

'IP_f23_A' ---------------------------------------CTCCTCCGA

'IP_f23_B' ---------------------------------------CTCCTCCGA

'IP_m7_A' ---------------------------------------CTCCTCCGA

'IP_m7_B' ---------------------------------------CTCCTCCGA

'PVL_f14_A' ---------------------------------------CTCCTCCGA

'PVL_f14_B' ---------------------------------------CTCCTCCGA

'PVL_f12_A' ---------------------------------------CTCCTCCGA

'PVL_f12_B' ---------------------------------------CTCCTCCGA

'PVL_f3_A' ---------------------------------------CTCCTCCGA

'PVL_f3_B' ---------------------------------------CTCCTCCGA

'PVL_f7_A' ---------------------------------------CTCCTCCGA

'PVL_f7_B' ---------------------------------------CTCCTCCGA

'Pes_m9_A' ATGTACCAAA-CTAATACTAAAACCTTATTGCGATAGGTCTCCTCCGA

'Pes_m9_B' ATGTAGCAAAGCTAATACTAAAACCTTATTGCTATAGGCCTCCTCCGA

'Pes_m10_A' ATGTACCAAA-CTAATACTAAAACCTTATTGCGATAGGTCTCCTCCGA

'Pes_m10_B' ATGTACCAAA-CTAATACTAAAACCTTATTGCGATAGGTCTCCTCCGA

'LH_m1_A' ATGTAGCAAAGCTAATACTAAAACCTTATTGCTATAGGCCTCCTCCGA

'LH_m1_B' ATGTAGCAAAGCTAATACTAAAACCTTATTGCTATAGGCCTCCTCCGA

'LH_f6_A' ATGTAGCAAAGCTAATACTAAAACCTTATTGCTATAGGCCTCCTCCGA

'LH_f6_B' ATGTAGCAAAGCTAATACTAAAACCTTATTGCTATAGGCCTCCTCCGA

'LH_f8_A' ATGTAGCAAAGCTAATACTAAAACCTTATTGCTATAGGCCTCCTCCGA

'LH_f8_B' ATGTAGCAAAGCTAATACTAAAACCTTATTGCTATAGGCCTCCTCCGA

'scn_h_a' ATGTAGCAAAGCTAATACTAAAACCTTATTGCTATAGGCCTCCTCCGA

'scn_h_b' ATGTAGCAAAGCTAATACTAAAACCTTATTGCTATAGGCCTCCTCCGA

'scn_m5_A' ATGTAGCAAAGCTAATACTAAAACCTTATTGCTATAGGCCTCCTCCGA

'scn_m5_B' ATGTAGCAAAGCTAATACTAAAACCTTATTGCTATAGGCCTCCTCCGA

'scn_m7_A' ATGTAGCAAAGCTAATACTAAAACCTTATTGCTATAGGCCTCCTCCGA

'scn_m7_B' ATGTAGCAAAGCTAATACTAAAACCTTATTGCTATAGGCCTCCTCCGA

'scn_m3_A' ATGTAGCAAAGCTAATACTAAAACCTTATTGCTATAGGCCTCCTCCGA

'scn_m3_B' ATGTAGCAAAGCTAATACTAAAACCTTATTGCTATAGGCCTCCTCCGA

'LJS_m1_A' GTGTACCAAAGCTAGCACTAAACCCTTATTGCTCTAGGTCTCCTCCGA

'LJS_m1_B' GTGTACCAAAGCTAGCACTAAACCCTTATTGCTCTAGGTCTCCTCCGA

'LJS_m2_A' GTGTACCAAAGCTAGTACTAAACCCTTATTGCTCTAGGTCTCCTCCGA

'LJS_m2_B' GTGTACCAAAGCTAGCACTAAACCCTTATTGCTCTAGGTCTCCTCCGA

'LJS_f10_A' GTGTACCAAAGCTAGTACTAAACCCTTCTTGCTATAGGTCTCCTCCGA

'LJS_f10_B' GTGTACCAAAGCTAGCACTAAACCCTTATTGCTCTAGGTCTCCTCCGA

'LJS_f9_B' -------AAAGCTAGCACTAAACCCTTATTGCTCTAGGTCTCCTCCGA

'LJS_f9_A' GTGTACCAAAGCTAGCACTAAACCCTTATTGCTCTAGGTCTCCTCCGA

'LJP_m7_B' GTGTACCAAAGCTAGTACTAAACCCTTATTGCTCTAGGTCTCCTCCGA

'LJP_m7_A' GTGTACCAAAGCTAGTACTAAACCCTTATTGCTCTAGGTCTCCTCCGA

'LJP_m6_B' GTGTACCAAAGCTAGTACTAAACCCTTATTGCTCTAGGTCTCCTCCGA

'LJP_m6_A' GTGTACCAAAGCTAGCACTAAACCCTTATTGCTCTAGGTCTCCTCCGA

'LJP_m2_B' GTGTACCAAAGCTAGTACTAAACCCTTATTGCTCTAGGTCTCCTCCGA

'LJP_m2_A' GTGTACCAAAGCTAGTACTAAACCCTTATTGCTCTAGGTCTTCTCCGA

'LJP_m1_B' GTGTACCAAAGCTAGCACTAAACCCTTATTGCTCTAGGTCTCCTCCGA

'LJP_m1_A' GTGTACCAAAGCTAGCACTAAACCCTTATTGCTCTAGGTCTCCTCCGA

'BR_m5_A' GTGTACCAAAGCTAGTACTAAACCCTTATTGCTCTAGGTCTCCTCCGA

'BR_m5_B' GTGTACCAAAGCTAGCACTAAACCCTTATTGCTCTAGGTCTCCTCCGA

'BR_m6_A' GTGTACCAAAGCTAGCACTAAACCCTTATTGCTCTAGGTCTCCTCCGA

'BR_m6_B' GTGTACCAAAGCTAGCACTAAACCCTTATTGCTCTAGGTCTCCTCCGA

'BR_m7_A' GTGTACCAAAGCTAGCACTAAACCCTTATTGCTCTAGGTCTCCTCCGA

'BR_m7_B' GTGTACCAAAGCTAGCACTAAACCCTTATTGCTCTAGGTCTCCTCCGA

'BR_f13_B' GTGTACCAAAGCTAGTACTAAACCCTTATTGCTCTAGGTCTCCTCCGA

'BR_f13_A' GTGTACCAAAGCTAGCACTAAACCCTTATTGCTCTAGGTCTCCTCCGA

'BR_m8_B' GTGTACCAAAGCTAGCACTAAACCCTTATTGCTCTAGGTCTCCTCCGA

'BR_m8_A' GTGTACCAAAGCTAGCACTAAACCCTTATTGCTCTAGGTCTCCTCCGA

'SD_h_a' GTGTACCAAAGCTAGTACTAAACCCTTATTGCTCTAGGTCTCCTCCGA

'SD_h_b' GTGTACCAAAGCTAGTACTAAACCCTTATTGCTCTAGGTCTCCTCCGA

'SD_g_a' GTGTACCAAAGCTAGTACTAAACCCTTATTGCTCTAGGTCTCCTCCGA

'SD_g_b' GTGTACCAAAGCTAGTACTAAACCCTTATTGCTCTAGGTCTCCTCCGA

'SD_2m_b' GTGTACCAAAGCTAGTACTAAACCCTTATTGCTCTAGGTCTCCTCCGA

'SD_2m_a' GTGTACCAAAGCTAGTACTAAACCCTTATTGCTCTAGGTCTCCTCCGA

[ 770 780 790 800 810 ]

[ * * * * * ]

'RP1_m1_A' TTGGGACAACTA--------GAATCTGAAGCATTTACAGTGATACATT

'RP1_m1_B' TTGGGACAACTA--------GAATCTGAAGCATTTACAGTGATACATT

'RP1_m5(A)' TTGGGACAACTA--------GAATCTGAAGCATTTACAGTGATACATT

'RP1_m5_B' TTGGGACAACTA--------GAATCTGAAGCATTTACAGTGATACATT

'RP1_m7_A' TTGGGACAACTA--------GAATCTGAAGCATTTACAGTGATACATT

'RP1_m7_B' TTGGGACAACTA--------GAATCTGAAGCATTTACAGTGATACATT

'RP1_m8_A' TTGGGACAACTA--------GAATCTGAAGCATTTACAGTGATACATT

'RP1_m8_B' TTGGGACAACTA--------GAATCTGAAGCATTTACAGTGATACATT

'R_Abg' TTGGGACAACTA--------GAATCTGAAGCATTTACAGTGATACATT

'R_Abg_2' TTGGGACAACTA--------GAATCTGAAGCATTTACAGTGATACATT

'AB_m1_A' TTGGGACAACTA--------GAATCTGAAGCATTTACAGTGATACATT

'AB_m1_B' TTGGGACAACTA--------GAATCTGAAGCATTTACAGTGATACATT

Abh TTGGGACAACTA--------GAATCTGAAGCATTTACAGTGATACATT

'Abh_2' TTGGGACAACTA--------GAATCTGAAGCATTTACAGTGATACATT

'AB_m2_A' TTGGGACAACTA--------GAATCTGAAGCATTTACAGTGATACATT

'AB_m2_B' TTGGGACAACTA--------GAATCTGAAGCATTTACAGTGATACATT

'IP_m8_A' TTGGGACAACTA--------GAATCTGAAGCATTTACAGTGATACATT

'IP_m8_B' TTGGGACAACTA--------GAATCTGAAGCATTTACAGTGATACATT

'IP_f3_A' TTGGGACAACTA--------GAATCTGAAGCATTTACAGTGATACATT

'IP_f3_B' TTGGGACAACTA--------GAATCTGAAGCATTTACAGTGATACATT

'IP_f23_A' TTGGGACAACTA--------GAATCTGAAGCATTTACAGTGATACATT

'IP_f23_B' TTGGGACAACTA--------GAATCTGAAGCATTTACAGTGATACATT

'IP_m7_A' TTGGGACAACTA--------GAATCTGAAGCATTTACAGTGATACATT

'IP_m7_B' TTGGGACAACTA--------GAATCTGAAGCATTTACAGTGATACATT

'PVL_f14_A' TTGGGACAACTA--------GAATCTGAAGCATTTACAGTGATACATT

'PVL_f14_B' TTGGGACAACTA--------GAATCTGAAGCATTTACAGTGATACATT

'PVL_f12_A' TTGGGACAACTA--------GAATCTGAAGCATTTACAGTGATACATT

'PVL_f12_B' TTGGGACAACTA--------GAATCTGAAGCATTTACAGTGATACATT

'PVL_f3_A' TTGGGACAACTA--------GAATCTGAAGCATTTACAGTGATACATT

'PVL_f3_B' TTGGGACAACTA--------GAATCTGAAGCATTTACAGTGATACATT

'PVL_f7_A' TTGGGACAACTA--------GAATCTGAAGCATTTACAGTGATACATT

'PVL_f7_B' TTGGGACAACTA--------GAATCTGAAGCATTTACAGTGATACATT

'Pes_m9_A' TGAGAACAACTA--------GAATCTGAAGCATT--------TACATT

'Pes_m9_B' TTAGAACAACTA--------GAATCTGAAGCATT--------GACCTT

'Pes_m10_A' TGAGAACAACTA--------GAATCTGAAGCATT--------TACATT

'Pes_m10_B' TGAGAACAACTA--------GAATCTGAAGCATT--------TACATT

'LH_m1_A' TTAGAACAACTA--------GAATCTGAAGCATT--------GACCTT

'LH_m1_B' TTAGAACAACTA--------GAATCTGAAGCATT--------GACCTT

'LH_f6_A' TTAGAACAACTA--------GAATCTGAAGCATT--------GACCTT

'LH_f6_B' TTAGAACAACTA--------GAATCTGAAGCATT--------GACCTT

'LH_f8_A' TTAGAACAACTA--------GAATCTGAAGCATT--------GACCTT

'LH_f8_B' TTAGAACAACTA--------GAATCTGAAGCATT--------GACCTT

'scn_h_a' TTAGAACAACTA--------GAATCTGAAGCATT--------GACCTT

'scn_h_b' TTAGAACAACTA--------GAATCTGAAGCATT--------GACCTT

'scn_m5_A' TTAGAACAACTA--------GAATCTGAAGCATT--------GACCTT

'scn_m5_B' TTAGAACAACTA--------GAATCTGAAGCATT--------GACCTT

'scn_m7_A' TTAGAACAACTA--------GAATCTGAAGCATT--------GACCTT

'scn_m7_B' TTAGAACAACTA--------GAATCTGAAGCATT--------GACCTT

'scn_m3_A' TTAGAACAACTA--------GAATCTGAAGCATT--------GACCTT

'scn_m3_B' TTAGAACAACTA--------GAATCTGAAGCATT--------GACCTT

'LJS_m1_A' TTGGGACAGCTACAGCTGTAGAATCTGAAGCATTTTCAGTGATACGTT

'LJS_m1_B' TTGGGACAGCTACAGCTGTAGAATCTGAAGCATTATCAGTGATACGTT

'LJS_m2_A' TTGGGACAGCTACAGCTGTAGAATCTGAAGCATTTTCAGTGATACGTT

'LJS_m2_B' TTGGGACAGCTACAGCTGTAGAATCTGAAGCATTTACAGTGATACATT

'LJS_f10_A' TTGGGACAGCTA--------GAATCTGAAGCATTTACAGTGATACATT

'LJS_f10_B' TTGGGACAGCTACAGCTGTAGAATCTGAAGCATTTACAGTGATACATT

'LJS_f9_B' TTGGGACAGCTACAGCTGTAGAATCTGAAGCATTTACAGTGATACGTT

'LJS_f9_A' TTGGGACAGCTACAGCTGTAGAATCTGAAGCATTTTCAGTGATACGTT

'LJP_m7_B' TTGGGACAGCTACAGCTGTAGAATCTGAAGCATTTACAGTGATACATT

'LJP_m7_A' TTGGGACAGCTACAGCTGTAGAATCTGAAGCATTTACAGTGATACATT

'LJP_m6_B' TTGGGACAGCTACAGCTGTAGAATCTGAAGCATTTACAGTGATACATT

'LJP_m6_A' TTGGGACAGCTACAGCTGTAGAATCTGAAGCATTTACAGTGATACATT

'LJP_m2_B' TTGGGACAGCTACAGCTGTAGAATCTGAAGCATTTACAGTGATACGTT

'LJP_m2_A' TTGGGACAGCTACAGCTGTAGAATCTGAAGCATTTACAGTGATACATT

'LJP_m1_B' TTGGGACAGCTACAGCTGTAGAATCTGAAGCATTTACAGTGATACATT

'LJP_m1_A' TTGGGACAGCTACAGCTGTAGAATCTGAAGCATTTACAGTGATACATT

'BR_m5_A' TTGGGACAGCTACAGCTGTAGAATCTGAAGCATTTACAGTGATATATT

'BR_m5_B' TTGGGACAGCTACAGCTGTAGAATCTGAAGCATTTTCAGTGATACGTT

'BR_m6_A' TTGGGACAGCTACAGCTGTAGAATCTGAAGCATTTTCAGTGATACGTT

'BR_m6_B' TTGGGACAGCTACAGCTGTAGAATCTGAAGCATTTTCAGTGATACGTT

'BR_m7_A' TTGGGACAGCTACAGCTGTAGAATCTGAAGCATTTTCAGTGATACGTT

'BR_m7_B' TTGGGACAGCTACAGCTGTAGAATCTGAAGCATTTTCAGTGATACGTT

'BR_f13_B' TTGGGACAGCTACAGATGTAGAATCTGAAGCATTTTCAGTGATACGTT

'BR_f13_A' TTGGGACAGCTACAGCTGTAGAATCTGAAGCATTTTCAGTGATACGTT

'BR_m8_B' TTGGGACAGCTACAGCTGTAGAATCTGAAGCATTTTCAGTGATACGTT

'BR_m8_A' TTGGGACAGCTACAGCTGTAGAATCTGAAGCATTTTCAGTGATACGTT

'SD_h_a' TTGGGACAGCTA--------GAATCTGAAGCATTTACAGTGATACATT

'SD_h_b' TTGGGACAGCTA--------GAATCTGAAGCATTTACAGTGATACATT

'SD_g_a' TTGGGACAGCTA--------GAATCTGAAGCATTTACAGTGAT?????

'SD_g_b' TTGGGACAGCTA--------GAATCTGAAGCATTTACAGTGAT?????

'SD_2m_b' TTGGGACAGCTA--------GAATCTGAAGCATTTACAGTGATACATT

'SD_2m_a' TTGGGACAGCTA--------GAATCTGAAGCATTTACAGTGATACATT

[ 820 830 840 850 860 ]

[ * * * * * ]

'RP1_m1_A' ACCTTATTTTAT-TTTTTTCATTCTGTCTTGCGACGGATTTACTATTG

'RP1_m1_B' ACCTTATTTTAT-TTTTTTCATTCTGTCTTGCGACGGATTTACTATTG

'RP1_m5(A)' ACCTTATTTTAT-TTTTTTCATTCTGTCTTGCGACGGATTTACTATTG

'RP1_m5_B' ACCTTATTTTAT-TTTTTTCATTCTGTCTTGCGACGGATTTACTATTG

'RP1_m7_A' ACCTTATTTTAT-TTTTTTCATTCTGTCTTGCGACGGATTTACTATTG

'RP1_m7_B' ACCTTATTTTAT-TTTTTTCATTCTGTCTTGCGACGGATTTACTATTG

'RP1_m8_A' ACCTTATTTTAT-TTTTTTCATTCTGTCTTGCGACGGATTTACTATTG

'RP1_m8_B' ACCTTATTTTAT-TTTTTTCATTCTGTCTTGCGACGGATTTACTATTG

'R_Abg' ACCTTATTTTAT-TTTTTTCATTCTGTCTTGCGACGGATTTACTATTG

'R_Abg_2' ACCTTATTTTAT-TTTTTTCATTCTGTCTTGCGACGGATTTACTATTG

'AB_m1_A' ACCTTATTTTAT-TTTTTTCATTCTGTCTTGCGACGGATTTACTATTG

'AB_m1_B' ACCTTATTTTAT-TTTTTTCATTCTGTCTTGCGACGGATTTACTATTG

Abh ACCTTAT???AT-T????TCATTCTGTCTTGCGACGGATTTACTATTG

'Abh_2' ACCTTAT???AT-T????TCATTCTGTCTTGCGACGGATTTACTATTG

'AB_m2_A' ACCTTATTTTAT-TTTTTTCATTCTGTCTTGCGACGGATTTACTATTG

'AB_m2_B' ACCTTATTTTAT-TTTTTTCATTCTGTCTTGCGACGGATTTACTATTG

'IP_m8_A' ACCTTATTTTAT-TTTTTTCATTCTGTCTTGCGACGGATTTACTATTG

'IP_m8_B' ACCTTATTTTAT-TTTTTTCATTCTGTCTTGCGACGGATTTACTATTG

'IP_f3_A' ACCTTATTTTAT-TTTTTTCATTCTGTCTTGCGACGGATTTACTATTG

'IP_f3_B' ACCTTATTTTAT-TTTTTTCATTCTGTCTTGCGACGGATTTACTATTG

'IP_f23_A' ACCTTATTTTAT-TTTTTTCATTCTGTCTTGCGACGGATTTACTATTG

'IP_f23_B' ACCTTATTTTATTTTTTTTCATTCTGTCTTGCGACGGATTTACTATTG

'IP_m7_A' ACCTTATTTTAT-TTTTTTCATTCTGTCTTGCGACGGATTTACTATTG

'IP_m7_B' ACCTTATTTTAT-TTTTTTCATTCTGTCTTGCGACGGATTTACTATTG

'PVL_f14_A' ACCTTATTTTAT-TTTTTTCATTCTGTCTTGCGACAGATTTACTATTG

'PVL_f14_B' ACCTTATTTTAT-TTTTTTCATTCTGTCTTGCGACAGATTTACTATTG

'PVL_f12_A' ACCTTATTTTAT-TTTTTTCATTCTGTCTTGCGACGGATTTACTATTG

'PVL_f12_B' ACCTTATTTTAT-TTTTTTCATTCTGTCTTGCGACGGATTTACTATTG

'PVL_f3_A' ACCTTATTTTAT-TTTTTTCATTCTGTCTTGCGACAGATTTACTATTG

'PVL_f3_B' ACCTTATTTTAT-TTTTTTCATTCTGTCTTGCGACAGATTTACTATTG

'PVL_f7_A' ACCTTATTTTAT-TTTTTTCATTCTGTCTTGCGACAGATTTACTATTG

'PVL_f7_B' ACCTTATTTTAT-TTTTTTCATTCTGTCTTGCGACAGATTTACTATTG

'Pes_m9_A' ACCTTGTTTTA--TTCATTCATTCTGTCTTGCGACACATTTACTATTG

'Pes_m9_B' ACCTTGTTTTA--TTCATTCATTCTGTCTTGCGACAGATTTACTATTG

'Pes_m10_A' ACCTTGTTTTA--TTCATTCATTCTGTCTTGCGACACATTTACTATTG

'Pes_m10_B' ACCTTGTTTTA--TTCATTCATTCTGTCTTGCGACACATTTACTATTG

'LH_m1_A' ACCTTGTTTTA--TTCATTCATTCTGTCTTGCGACAGATTTACTATTG

'LH_m1_B' ACCTTGTTTTA--TTCATTCATTCTGTCTTGCGACAGATTTACTATTG

'LH_f6_A' ACCTTGTTTTA--TTCATTCATTCTGTCTTGCGACAGATTTACTATTG

'LH_f6_B' ACCTTGTTTTA--TTCATTCATTCTGTCTTGCGACAGATTTACTATTG

'LH_f8_A' ACCTTGTTTTA--TTCATTCATTCTGTCTTGCGACAGATTTACTATTG

'LH_f8_B' ACCTTGTTTTA--TTCATTCATTCTGTCTTGCGACAGATTTACTATTG

'scn_h_a' ACCTTGTTTTA--TTCATTCATTCTGTCTTGCGACAGATTTACTATTG

'scn_h_b' ACCTTGTTTTA--TTCATTCATTCTGTCTTGCGACAGATTTACTATTG

'scn_m5_A' ACCTTGTTTTA--TTCATTCATTCTGTCTTGCGACAGATTTACTATTG

'scn_m5_B' ACCTTGTTTTA--TTCATTCATTCTGTCTTGCGACAGATTTACTATTG

'scn_m7_A' ACCTTGTTTTA--TTCATTCATTCTGTCTTGCGACAGATTTACTATTG

'scn_m7_B' ACCTTGTTTTA--TTCATTCATTCTGTCTTGCGACAGATTTACTATTG

'scn_m3_A' ACCTTGTTTTA--TTCATTCATTCTGTCTTGCGACAGATTTACTATTG

'scn_m3_B' ACCTTGTTTTA--TTCATTCATTCTGTCTTGCGACAGATTTACTATTG

'LJS_m1_A' ACCTTATTT------TTTTCATTCTGTCTTGCGACAGATTTACTATTG

'LJS_m1_B' ACCTTATTT------TTTTCATTCTGTCTTGCGACAGATTTACTATTG

'LJS_m2_A' ACCTTATTT------TTTTCATTCTGTCTTGCGACAGATTTACTATTG

'LJS_m2_B' ACCTTA-TT------TTTTCATTCTGTCTTGCGACAGATTTACTATTG

'LJS_f10_A' ACCTTA-TT------TTTTCATTCTGTCTTGCGACAGATTTACTATTG

'LJS_f10_B' ACCTTA-TT------TTTTCATTCTGTCTTGCGACAGATTTACTATTG

'LJS_f9_B' ACCTTATTT------TTTTCATTCTGTCTTGCGACAGATTTACTATTG

'LJS_f9_A' ACCTTATTT------TTTTCATTCTGTCTTGCGACAGATTTACTATTG

'LJP_m7_B' ACCTTA-TT------TTTTCATTCTGTCTTGCGACAGATTTACTATTG

'LJP_m7_A' ACCTTA-TT------TTTTCATTCTGTCTTGCGACAGATTTACTATTG

'LJP_m6_B' ACCTTA-TT------TTTTCATTCTGTCTTGCGACAGATTTACTATTG

'LJP_m6_A' ACCTTA-TT------TTTTCATTCTGTCTTGCGACAGATTTACTATTG

'LJP_m2_B' ACCTTATTT------TTTTCATTCTGTCTTGCGACAGATTTACTATTG

'LJP_m2_A' ACCTTA-TT------TTTTCATTCTGTCTTGCGACAGATTTACTATTG

'LJP_m1_B' ACCTTA-TT------TTTTCATTCTGTCTTGCGACAGATTTACTATTG

'LJP_m1_A' ACCTTA-TT------TTTTCATTCTGTCTTGCGACAGATTTACTATTG

'BR_m5_A' ACCTTA-TT------TTTTCATTCTGTCTTGCGACAGATTTACTATTG

'BR_m5_B' ACCTTATTT------TTTTCATTCTGTCTTGCGACAGATTTACTATTG

'BR_m6_A' ACCTTATTT------TTTTCATTCTGTCTTGCGACAGATTTACTATTG

'BR_m6_B' ACCTTATTT------TTTTCATTCTGTCTTGCGACAGATTTACTATTG

'BR_m7_A' ACCTTATTT------TTTTCATTCTGTCTTGCGACAGATTTACTATTG

'BR_m7_B' ACCTTATTT------TTTTCATTCTGTCTTGCGACAGATTTACTATTG

'BR_f13_B' ACCTTATTT------TTTTCATTCTGTCTTGCGACAGATTTACTATTG

'BR_f13_A' ACCTTATTT------TTTTCATTCTGTCTTGCGACAGATTTACTATTG

'BR_m8_B' ACCTTATTT------TTTTCATTCTGTCTTGCGACAGATTTACTATTG

'BR_m8_A' ACCTTATTT------TTTTCATTCTGTCTTGCGACAGATTTACTATTG

'SD_h_a' ACCTTATTT------TTTTCATTCTGTCTTGCGGCAGATTTACTATTG

'SD_h_b' ACCTTATTT------TTTTCATTCTGTCTTGCGGCAGATTTACTATTG

'SD_g_a' ???????TT------TTTTCATTCTGTCTTGCGGCAGATTTACTATTG

'SD_g_b' ???????TT------TTTTCATTCTGTCTTGCGGCAGATTTACTATTG

'SD_2m_b' ACCTTATTT------TTTTCATTCTGTCTTGCGGCAGATTTACTATTG

'SD_2m_a' ACCTTATTT------TTTTCATTCTGTCTTGCGGCAGATTTACTATTG

[ 870 880 890 900 910 ]

[ * * * * * ]

'RP1_m1_A' ATCATGTAACGAATGACCTAGTACACTGGCTTCTCCATTGAAGTTTGG

'RP1_m1_B' ATCATGTAACGAATGACCTAGTACACTGGCTTCTCCATTGAAGTTTGG

'RP1_m5(A)' ATCATGTAACGAATGACCTAGTACACTGGCTTCTCCATTGAAGTTTGG

'RP1_m5_B' ATCATGTAACGAATGACCTAGTACACTGGCTTCTCCATTGAAGTTTGG

'RP1_m7_A' ATCATGTAACGAATGACCTAGTACACTGGCTTCTCCATTGAAGTTTGG

'RP1_m7_B' ATCATGTAACGAATGACCTAGTACACTGGCTTCTCCATTGAAGTTTGG

'RP1_m8_A' ATCATGTAACGAATGACCTAGTACACTGGCTTCTCCATTGAAGTTTGG

'RP1_m8_B' ATCATGTAACGAATGACCTAGTACACTGGCTTCTCCATTGAAGTTTGG

'R_Abg' ATCATGTAACGAATGACCTAGTACACTGGCTTCTCCATTGAAGTTTGG

'R_Abg_2' ATCATGTAACGAATGACCTAGTACACTGGCTTCTCCATTGAAGTTTGG

'AB_m1_A' ATCATGTAACGAATGACCTAGTACACTGGCTTCTCCATTGAAGTTTGG

'AB_m1_B' ATCATGTAACGAATGACCTAGTACACTGGCTTCTCCATTGAAGTTTGG

Abh ATCATGTAACGAATGACCTAGTACACTGGCTTCTCCATTGAAGTTTGG

'Abh_2' ATCATGTAACGAATGACCTAGTACACTGGCTTCTCCATTGAAGTTTGG

'AB_m2_A' ATCATGTAACGAATGACCTAGTACACTGGCTTCTCCATTGAAGTTTGG

'AB_m2_B' ATCATGTAACGAATGACCTAGTACACTGGCTTCTCCATTGAAGTTTGG

'IP_m8_A' ATCATGTAACGAATGACCTAGTACACTGGCTTCTCCATTGAAGTTTGG

'IP_m8_B' ATCATGTAACGAATGACCTAGTACACTGGCTTCTCCATTGAAGTTTGG

'IP_f3_A' ATCATGTAACGAATGACCTAGTACACTGGCTTCTCCATTGAAGTTTGG

'IP_f3_B' ATCATGTAACGAATGACCTAGTACACTGGCTTCTCCATTGAAGTTTGG

'IP_f23_A' ATCATGTAACGAATGACCTAGTACACTGGCTTCTCCATTGAAGTTTGG

'IP_f23_B' ATCATGTAACGAATGACCTAGTACACTGGCTTCTCCATTGAAGTTTGG

'IP_m7_A' ATCATGTAACGAATGACCTAGTACACTGGCTTCTCCATTGAAGTTTGG

'IP_m7_B' ATCATGTAACGAATGACCTAGTACACTGGCTTCTCCATTGAAGTTTGG

'PVL_f14_A' ATCATGTAACGAATGACCTAGTACACTGGCTTCTCCATTGAAGTTTGG

'PVL_f14_B' ATCATGTAACGAATGACCTAGTACACTGGCTTCTCCATTGAAGTTTGG

'PVL_f12_A' ATCATGTAACGAATGACCTAGTACACTGGCTTCTCCATTGAAGTTTGG

'PVL_f12_B' ATCATGTAACGAATGACCTAGTACACTGGCTTCTCCATTGAAGTTTGG

'PVL_f3_A' ATCATGTAACGAATGACCTAGTACACTGGCTTCTCCATTGAAGTTTGG

'PVL_f3_B' ATCATGTAACGAATGACCTAGTACACTGGCTTCTCCATTGAAGTTTGG

'PVL_f7_A' ATCATGTAACGAATGACCTAGTACACTGGCTTCTCCATTGAAGTTTGG

'PVL_f7_B' ATCATGTAACGAATGACCTAGTACACTGGCTTCTCCATTGAAGTTTGG

'Pes_m9_A' ATCATGTAACGAATGTCATAGTACACTGGCTTCTCCATTGAAGGTTGG

'Pes_m9_B' ATCATGTAACGAATGTCATAGTACACTGGCTTCTCCATTGAAGGTTGG

'Pes_m10_A' ATCATGTAACGAATGTCATAGTACACTGGCTTCTCCATTGAAGGTTGG

'Pes_m10_B' ATCATGTAACGAATGTCATAGTACACTGGCTTCTCCATTGAAGGTTGG

'LH_m1_A' ATCATGTAACGAATGTCATAGTACACTGGCTTCTCCATTGAAGGTTGG

'LH_m1_B' ATCATGTAACGAATGTCATAGTACACTGGCTTCTCCATTGAAGGTTGG

'LH_f6_A' ATCATGTAACGAATGTCATAGTACACTGGCTTCTCCATTGAAGGTT-G

'LH_f6_B' ATCATGTAACGAATGTCATAGTACACTGGCTTCTCCATTGAAGGTTGG

'LH_f8_A' ATCATGTAACGAATGTCATAGTACACTGGCTTCTCCATTGAAGGTTGG

'LH_f8_B' ATCATGTAACGAATGTCATAGTACACTGGCTTCTCCATTGAAGGTTGG

'scn_h_a' ATCATGTAACGAATGTCATAGTACACTGGCTTCTCCATTGAAGGTTGG

'scn_h_b' ATCATGTAACGAATGTCATAGTACACTGGCTTCTCCATTGAAGGTTGG

'scn_m5_A' ATCATGTAACGAATGTCATAGTACACTGGCTTCTCCATTGAAGGTTGG

'scn_m5_B' ATCATGTAACGAATGTCATAGTACACTGGCTTCTCCATTGAAGGTTGG

'scn_m7_A' ATCATGTAACGAATGTCATAGTACACTGGCTTCTCCATTGAAGGTTGG

'scn_m7_B' ATCATGTAACGAATGTCATAGTACACTGGCTTCTCCATTGAAGGTTGG

'scn_m3_A' ATCATGTAACGAATGTCATAGTACACTGGCTTCTCCATTGAAGGTTGG

'scn_m3_B' ATCATGTAACGAATGTCATAGTACACTGGCTTCTCCATTGAAGGTTGG

'LJS_m1_A' ATCATGTAACGAATGAATTAGTACACTGGCTCCTCCATTGAGGTTTGG

'LJS_m1_B' ATCATGTAACGAATGAATTAGTACACTGGCTCCTCCATTGAGGTTTGG

'LJS_m2_A' ATCATGTAACGAATGAATTAGTACACTGGCTCCTCCATTGAGGTTTGG

'LJS_m2_B' ATCATGTAACGAATGAATTAGTACACTGGCTCCTCCATTGAGGTTTGG

'LJS_f10_A' ATCATGTAACGAATGAATTAGTACACTGGCTCCTCCATTGAGGTTTGG

'LJS_f10_B' ATCATGTAACGAATGAATTAGTACACTGGCTCCTCCATTGAGGTTTGG

'LJS_f9_B' ATCATGTAACGAATGAATTAGTACACTGGCTCCTCCATTGAGGTTTGG

'LJS_f9_A' ATCATGTAACGAATGAATTAGTACACTGGCTCCTCCATTGAGGTTTGG

'LJP_m7_B' ATCATGTAACGAATGAATTAGTACACTGGCTCCTCCATTGAGGTTTGG

'LJP_m7_A' ATCATGTAACGAATGAATTAGTACACTGGCTCCTCCATTGAGGTTTGG

'LJP_m6_B' ATCATGTAACGAATGAATTAGTACACTGGCTCCTCCATTGAGGTTTGG

'LJP_m6_A' ATCATGTAACGAATGAATTAGTACACTGGCTCCTCCATTGAGGTTTGG

'LJP_m2_B' ATCATGTAACGAATGAATTAGTACACTGGCTCCTCCATTGAGGTTTGG

'LJP_m2_A' ATCATGTAACGAATGAATTAGTACACTGGCTCCTCCATTGAGGTTTGG

'LJP_m1_B' ATCATGTAACGAATGAATTGGTACACTGGCTCCTCCATTGAGGTTTGG

'LJP_m1_A' ATCATGTAACGAATGAATTAGTACACTGGCTCCTCCATTGAGGTTTGG

'BR_m5_A' ATCATGTAACGAATGAATTAGTACACTGGCTCCTCCATTGAGGTTTGG

'BR_m5_B' ATCATGTAACGAATGAATTAGTACACTGGCTCCTCCATTGAGGTTTGG

'BR_m6_A' ATCATGTAACGAATGAATTAGTACACTGGCTCCTCCATTGAGGTTTGG

'BR_m6_B' ATCATGTAACGAATGAATTAGTACACTGGCTCCTCCATTGAGGTTTGG

'BR_m7_A' ATCATGTAACGAATGAATTAGTACACTGGCTCCTCCATTGAGGTTTGG

'BR_m7_B' ATCATGTAACGAATGAATTAGTACACTGGCTCCTCCATTGAGGTTTGG

'BR_f13_B' ATCATGTAACGAATGAATTAGTACACTGGCTCCTCCATTGAGGTTTGG

'BR_f13_A' ATCATGTAACGAATGAATTAGTACACTGGCTCCTCCATTGAGGTTTGG

'BR_m8_B' ATCATGTAACGAATGAATTAGTACACTGGCTCCTCCATTGAGGTTTGG

'BR_m8_A' ATCATGTAACGAATGAATTAGTACACTGGCTCCTCCATTGAGGTTTGG

'SD_h_a' ATCATGTAACGAATGAATTAGTACACTGGCTCCTCCATTGAGGTTTGG

'SD_h_b' ATCATGTAACGAATGAATTAGTACACTGGCTCCTCCATTGAGGTTTGG

'SD_g_a' ATCATGTAACGAATGAATTAGTACACTGGCTCCTCCATTGAGGTTTGG

'SD_g_b' ATCATGTAACGAATGAATTAGTACACTGGCTCCTCCATTGAGGTTTGG

'SD_2m_b' ATCATGTAACGAATGAATTAGTACACTGGCTCCTCCATTGAGGTTTGG

'SD_2m_a' ATCATGTAACGAATGAATTAGTACACTGGCTCCTCCATTGAGGTTTGG

[ 920 930 940 950 960]

[ * * * * *]

'RP1_m1_A' GGGTTAGGCTATGTTTCTGGAGTTTTTGGTCCGGAACAATCAATGAAG

'RP1_m1_B' GGGTTAGGCTATGTTTCTGGAGTTTTTGGTCCGGAACAATCAATGAAG

'RP1_m5(A)' GGGTTAGGCTATGTTTCTGGAGTTTTTGGTCCGGAACAATCAATGAAG

'RP1_m5_B' GGGTTAGGCTATGTTTCTGGAGTTTTTGGTCCGGAACAATCAATGAAG

'RP1_m7_A' GGGTTAGGCTATGTTTCTGGAGTTTTTGGTCCGGAACAATCAATGAAG

'RP1_m7_B' GGGTTAGGCTATGTTTCTGGAGTTTTTGGTCCGGAACAATCAATGAAG

'RP1_m8_A' GGGTTAGGCTATGTTTCTGGAGTTTTTGGTCCGGAACAATCAATGAAG

'RP1_m8_B' GGGTTAGGCTATGTTTCTGGAGTTTTTGGTCCGGAACAATCAATGAAG

'R_Abg' GGGTTAGGCTATGTTTCTGGAGTTTTTGGTCCGGAACAATCAATGAAG

'R_Abg_2' GGGTTAGGCTATGTTTCTGGAGTTTTTGGTCCGGAACAATCAATGAAG

'AB_m1_A' GGGTTAGGCTATGTTTCTGGAGTTTTTGGTCCGGAACAATCAATGAAG

'AB_m1_B' GGGTTAGGCTATGTTTCTGGAGTTTTTGGTCCGGAACAATCAATGAAG

Abh GGGTTAGGCTATGTTTCTGGAGTTTTTGGTCCGGAACAATCAATGAAG

'Abh_2' GGGTTAGGCTATGTTTCTGGAGTTTTTGGTCCGGAACAATCAATGAAG

'AB_m2_A' GGGTTAGGCTATGTTTCTGGAGTTTTTGGTCCGGAACAATCAATGAAG

'AB_m2_B' GGGTTAGGCTATGTTTCTGGAGTTTTTGGTCCGGAACAATCAATGAAG

'IP_m8_A' GGGTTAGGCTATGTTTCTGGAGTTTTTGGTCCGGAACAATCAATGAAG

'IP_m8_B' GGGTTAGGCTATGTTTCTGGAGTTTTTGGTCCGGAACAATCAATGAAG

'IP_f3_A' GGGTTAGGCTATGTTTCTGGAGTTTTTGGTCCGGAACAATCAATGAAG

'IP_f3_B' GGGTTAGGCTATGTTTCTGGAGTTTTTGGTCCGGAACAATCAATGAAG

'IP_f23_A' GGGTTAGGCTATGTTTCTGGAGTTTTTGGTCCGGAACAATCAATGAAG

'IP_f23_B' GGGTTAGGCTATGTTTCTGGAGTTTTTGGTCCGGAACAATCAATGAAG

'IP_m7_A' GGGTTAGGCTATGTTTCTGGAGTTTTTGGTCCGGAACAATCAATGAAG

'IP_m7_B' GGGTTAGGCTATGTTTCTGGAGTTTTTGGTCCGGAACAATCAATGAAG

'PVL_f14_A' GGGTTAGGCTATGTTTCTGGAGTTTTTGGTCCGGAACAATCGATGAAG

'PVL_f14_B' GGGTTAGGCTATGTTTCTGGAGTTTTTGGTCCGGAACAATCAATGAAG

'PVL_f12_A' GGGTTAGGCTATGTTTCTGGAGTTTTTGGTCCGGAACAATCAATGAAG

'PVL_f12_B' GGGTTAGGCTATGTTTCTGGAGTTTTTGGTCCGGAACAATCAATGAAG

'PVL_f3_A' GGGTTAGGCTATGTTTCTGGAGTTTTTGGTCCGGAACAATCAATGAAG

'PVL_f3_B' GGGTTAGGCTATGTTTCTGGAGTTTTTGGTCCGGAACAATCGATGAAG

'PVL_f7_A' GGGTTAGGCTATGTTTCTGGAGTTTTTGGTCCGGAACAATCGATGAAG

'PVL_f7_B' GGGTTAGGCTATGTTTCTGGAGTTTTTGGTCCGGAACAATCGATGAAG

'Pes_m9_A' GGGTTAGTCTAGGTTTCTTGAGTTTTTGGTCCTAAACAACCAATGAAG

'Pes_m9_B' GGGTTAGTCTAGGTTTCTTGAGTTTTTGGTCCTGAACAATCAATGAAG

'Pes_m10_A' GGGTTAGTCTAGGTTTCTTGAGTTTTTGGTCCTAAACAACCAATGAAG

'Pes_m10_B' GGGTTAGTCTAGGTTTCTTGAGTTTTTGGTCCTAAACAACCAATGAAG

'LH_m1_A' GGGTTAATCTAGGTTTCTTGAGTTTTTGGTCCTGAACAATCAATGAAG

'LH_m1_B' GGGTTAATCTAGGTTTCTTGAGTTTTTGGTCCTGAACAATCAATGAAG

'LH_f6_A' GGGTTAATCTAGGTTTCTTGAGTTTTTGGTCCTGAACAATCAATGAAG

'LH_f6_B' GGGTTAATCTAGGTTTCTTGAGTTTTTGGTCCTGAACAATCAATGAAG

'LH_f8_A' GGGTTAATCTAGGTTTCTTGAGTTTTTGGTCCTGAACAATCAATGAAG

'LH_f8_B' GGGTTAATCTAGGTTTCTTGAGTTTTTGGTCCTGAACAATCAATGAAG

'scn_h_a' GGGTTAATCTAGGTTTCTTGAGTTTTTGGTCCTGAACAATCAATGAAG

'scn_h_b' GGGTTAATCTAGGTTTCTTGAGTTTTTGGTCCTGAACAATCAATGAAG

'scn_m5_A' GGGTTAATCTAGGTTTCTTGAGTTTTTGGTCCTGAACAATCAATGAAG

'scn_m5_B' GGGTTAATCTAGGTTTCTTGAGTTTTTGGTCCTGAACAATCAATGAAG

'scn_m7_A' GGGTTAATCTAGGTTTCTTGAGTTTTTGGTCCTGAACAATCAATGAAG

'scn_m7_B' GGGTTAATCTAGGTTTCTTGAGTTTTTGGTCCTGAACAATCAATGAAG

'scn_m3_A' GGGTTAATCTAGGTTTCTTGAGTTTTTGGTCCTGAACAATCAATGAAG

'scn_m3_B' GGGTTAATCTAGGTTTCTTGAGTTTTTGGTCCTGAACAATCAATGAAG

'LJS_m1_A' GGGTTAGGCTAGGTTTGTTGAGTTTTTGGTCCGGAACAATCAATGAAG

'LJS_m1_B' GGGTTAGGCTAGGTTTGTTGAGTTTTTGGTCCGGAACAATCAATGAAG

'LJS_m2_A' GGGTTAGTCTAGGTTTGTTGAGTTTTTGGTCCGGAACAATCAATGAAG

'LJS_m2_B' GGGTTAGTCTAGGTTTGTTGAGTTTTTGGTCCGGAACAATCAATGAAG

'LJS_f10_A' GGGTTAGGCTAGGTTTGTTGAGTTTTTGGTCCGGAACAATCAATGAAG

'LJS_f10_B' GGGTTAGTCTAGGTTTGTTGAGTTTTTGGTCCGGAACAATCAATGAAG

'LJS_f9_B' GGGTTAGGCTAGGTTTGTTGAGTTTTTGGTCCGGAACAATCAATGAAG

'LJS_f9_A' GGGTTAGGCTAGGTTTGTTGAGTTTTTGGTCCGGAACAATCAATGAAG

'LJP_m7_B' GGGTTAGGCTAGGTTTGTTGAGTTTTTGGTCCGGAACAATCAATGAAG

'LJP_m7_A' GGGTTAGTCTAGGTTTGTTGAGTTTTTGGTCCGGAACAATCAATGAAG

'LJP_m6_B' GGGTTAGTCTAGGTTTGTTGAGTTTTTGGTCCGGAACAATCAATGAAG

'LJP_m6_A' GGGTTAGGCTAGGTTTGTTGAGTTTTTGGTCCGGAACAATCAATGAAG

'LJP_m2_B' GGGTTAGGCTAGGTTTGTTGAGTTTTTGGTCCGGAACAATCAATGAAG

'LJP_m2_A' GGGTTAGGCTAGGTTTGTTGAGTTTTTGGTCCGGAACAATCAATGAAG

'LJP_m1_B' GGGTTAGTCTAGGTTTGTTGAGTTTTTGGTCCGGAACAATCAATGAAG

'LJP_m1_A' GGGTTAGTCTAGGTTTGTTGAGTTTTTGGTCCGGAACAATCAATGAAG

'BR_m5_A' GGGTTAGTCTAGGTTTGTTGAGATTTTGGTCCGGAACAATCAATGAAG

'BR_m5_B' GGGTTAGGCTAGGTTTGTTGAGTTTTTGGTCCGGAACAATCAATGAAG

'BR_m6_A' GGGTTAGGCTAGGTTTGTTGAGTTTTTGGTCCGGAACAATCAATGAAG

'BR_m6_B' GGGTTAGGCTAGGTTTGTTGAGTTTTTGGTCCGGAACAATCAATGAAG

'BR_m7_A' GGGTTAGGCTAGGTTTGTTGAGTTTTTGGTCCGGAACAATCAATGAAG

'BR_m7_B' GGGTTAGGCTAGGTTTGTTGAGTTTTTGGTCCGGAACAATCAATGAAG

'BR_f13_B' GGGTTAGGCTAGGTTTGTTGAGTTTTTGGTCCGGAACAATCAATGAAG

'BR_f13_A' GGGTTAGGCTAGGTTTGTTGAGTTTTTGGTCCGGAACAATCAATGAAG

'BR_m8_B' GGGTTAGGCTAGGTTTGTTGAGTTTTTGGTCCGGAACAATCAATGAAG

'BR_m8_A' GGGTTAGGCTAGGTTTGTTGAGTTTTTGGTCCGGAACAATCAATGAAG

'SD_h_a' GGGTTAGGCTAGGTTTGTCGAGTTTTTGGTCCGGAACAATCAATGAAG

'SD_h_b' GGGTTAGGCTAGGTTTGTCGAGTTTTTGGTCCGGAACAATCAATGAAG

'SD_g_a' GGGTTAGGCTAGGTTTGTCGAGTTTTTGGTCCGGAACAATCAATGAAG

'SD_g_b' GGGTTAGGCTAGGTTTGTTGAGTTTTTGGTCCGGAACAATCAATGAAG

'SD_2m_b' GGGTTAGGCTAGGTTTGTCGAGTTTTTGGTCCGGAACAATCAATGAAG

'SD_2m_a' GGGTTAGGCTAGGTTTGTCGAGTTTTTGGTCCGGAACAATCAATGAAG

[ 970 980 990 1000 ]

[ * * * * ]

'RP1_m1_A' GTTCCGTATTCAAATCTAACCAACATGCCTTGCCACCCGGTACCTCTA

'RP1_m1_B' GTTCCGTATTCAAATCTAACCAACATGCCTTGCCACCCGGTACCTCTA

'RP1_m5(A)' GTTCCGTATTCAAATCTAACCAACATGCCTTGCCACCCGGTACCTCTA

'RP1_m5_B' GTTCCGTATTCAAATCTAACCAACATGCCTTGCCACCCGGTACCTCTA

'RP1_m7_A' GTTCCGTATTCAAATCTAACCAACATGCCTTGCCACCCGGTACCTCTA

'RP1_m7_B' GTTCCGTATTCAAATCTAACCAACATGCCTTGCCACCCGGTACCTCTA

'RP1_m8_A' GTTCCGTATTCAAATCTAACCAACATGCCTTGCCACCCGGTACCTCTA

'RP1_m8_B' GTTCCGTATTCAAATCTAACCAACATGCCTTGCCACCCGGTACCTCTA

'R_Abg' GTTCCGTATTCAAATCTAACCAACATGCCTTGCCACCCGGTACCTCTA

'R_Abg_2' GTTCCGTATTCAAATCTAACCAACATGCCTTGCCACCCGGTACCTCTA

'AB_m1_A' GTTCCGTATTCAAATCTAACCAACATGCCTTGCCACCCGGTACCTCTA

'AB_m1_B' GTTCCGTATTCAAATCTAACCAACATGCCTTGCCACCCGGTACCTCTA

Abh GTTCCGTATTCAAATCTAACC?ACATGCCTTGCCACCCGGTACCTCTA

'Abh_2' GTTCCGTATTCAAATCTAACC?ACATGCCTTGCCACCCGGTACCTCTA

'AB_m2_A' GTTCCGTATTCAAATCTAACCAACATGCCTTGCCACCCGGTACCTCTA

'AB_m2_B' GTTCCGTATTCAAATCTAACCAACATGCCTTGCCACCCGGTACCTCTA

'IP_m8_A' GTTCCGTATTCAAATCTAACCAACATGCCTTGCCACCCGGTACCTCTA

'IP_m8_B' GTTCCGTATTCAAATCTAACCAACATGCCTTGCCACCCGGTACCTCTA

'IP_f3_A' GTTCCGTATTCAAATCTAACCAACATGCCTTGCCACCCGGTACCTCTA

'IP_f3_B' GTTCCGTATTCAAATCTAACCAACATGCCTTGCCACCCGGTACCTCTA

'IP_f23_A' GTTCCGTATTCAAATCTAACCAACATGCCTTGCCACCCGGTACCTCTA

'IP_f23_B' GTTCCGTATTCAAATCTAACCAACATGCCTTGCCACCCGGTACCTCTA

'IP_m7_A' GTTCCGTATTCAAATCTAACCAACATGCCTTGCCACCCGGTACCTCTA

'IP_m7_B' GTTCCGTATTCAAATCTAACCAACATGCCTTGCCACCCGGTACCTCTA

'PVL_f14_A' GTTCCGTATTCAAATCTAACCAACATGCCTTGCCACCCGGTACCTCTA

'PVL_f14_B' GTTCCGTATTCAAATCTAACCAACATGCCTTGCCACCCGGTACCTCTA

'PVL_f12_A' GTTCCGTATTCAAATCTAACCAACATGCCTTGCCACCCGGTACCTCTA

'PVL_f12_B' GTTCCGTATTCAAATCTAACCAACATGCCTTGCCACCCGGTACCTCTA

'PVL_f3_A' GTTCCGTATTCAAATCTAACCAACATGCCTTGCCACCCGGTACCTCTA

'PVL_f3_B' GTTCCGTATTCAAATCTAACCAACATGCCTTGCCACCCGGTACCTCTA

'PVL_f7_A' GTTCCGTATTCAAATCTAACCAACATGCCTTGCCACCCGGTACCTCTA

'PVL_f7_B' GTTCCGTATTCAAATCTAACCAACATGCCTTGCCACCCGGTACCTCTA

'Pes_m9_A' GTTCCGTATTCAAATCTAACCAACTTGACTTGCCACCCGGTACCTCTA

'Pes_m9_B' GTTCCGTATTCAAATCTAACCAACTGGACTTGCCACCCGGTACCTCTA

'Pes_m10_A' GTTCCGTATTCAAATCTAACCAACTTGACTTGCCACCCGGTACCTCTA

'Pes_m10_B' GTTCCGTATTCAAATCTAACCAACTTGACTTGCCACCCGGTACCTCTA

'LH_m1_A' GTTCCGTATTCAAATCTAACCAACTGGACTTGCCACCCGGTACCTCTA

'LH_m1_B' GTTCCGTATTCAAATCTAACCAACTGGACTTGCCACCCGGTACCTCTA

'LH_f6_A' GTTCCGTATTCAAATCTAACCAACTGGACTTGCCACCCGGTACCTCTA

'LH_f6_B' GTTCCGTATTCAAATCTAACCAACTGGACTTGCCACCCGGTACCTCTA

'LH_f8_A' GTTCCGTATTCAAATCTAACCAACTGGACTTGCCACCCGGTACCTCTA

'LH_f8_B' GTTCCGTATTCAAATCTAACCAACTGGACTTGCCACCCGGTACCTCTA

'scn_h_a' GTTCCGTATTCAAATCTAACCAACTGGACTTGCCACCCGGTACCTCTA

'scn_h_b' GTTCCGTATTCAAATCTAACCAAGTGGACTTGCCACCCGGTACCTCTA

'scn_m5_A' GTTCCGTATTCAAATCTAACCAACTGGACTTGCCACCCGGTACCTCTA

'scn_m5_B' GTTCCGTATTCAAATCTAACCAACTGGACTTGCCACCCGGTACCTCTA

'scn_m7_A' GTTCCGTATTCAAATCTAACCAACTGGACTTGCCACCCGGTACCTCTA

'scn_m7_B' GTTCCGTATTCAAATCTAACCAACTGGACTTGCCACCCGGTACCTCTA

'scn_m3_A' GTTCCGTATTCAAATCTAACCAACTGGACTTGCCACCCGGTACCTCTA

'scn_m3_B' GTTCCGTATTCAAATCTAACCAACTGGACTTGCCACCCGGTACCTCTA

'LJS_m1_A' GTTCCGTATTCAAATCTAACCATCTTGCCTTGCCACCCGGTACCTCTA

'LJS_m1_B' GTTCCGTATTCAAATCTAACCATCTTGCCTTGCCACCCGGTACCTCTA

'LJS_m2_A' GTTCCGTATTCAAATCTAACCATCTTGCCTTGCCACCCGGTACCTCTA

'LJS_m2_B' GTTCCGTATTCAAATCTAACCATCTTGCCTTGCCACCCGGTACCTCTA

'LJS_f10_A' GTTCCGTATTCAAATCTAACCATCTTGCCTTGCCACCCGGTACCTCTA

'LJS_f10_B' GTTCCGTATTCAAATCTAACCATCTTGCCTTGCCACCCGGTACCTCTA

'LJS_f9_B' GTTCCGTATTCAAATCTAACCATCTTGCCTTGCCACCCGGTACCTCTA

'LJS_f9_A' GTTCCGTATTCAAATCTAACCATCTTGCCTTGCCACCCGGTACCTCTA

'LJP_m7_B' GTTCCGTATTCAAATCTAACCATCTTGCCTTGCCACCCGGTACCTCTA

'LJP_m7_A' GTTCCGTATTCAAATCTAACCATCTTGCCTTGCCACCCGGTACCTCTA

'LJP_m6_B' GTTCCGTATTCAAATCTAACCATCTTGCCTTGCCACCCGGTACCTCTA

'LJP_m6_A' GTTCCGTATTCAAATCTAACCATCTTGCCTTGCCACCCGGTACCTCTA

'LJP_m2_B' GTTCCGTATTCAAATCTAACCATCTTGCCTTGCCACCCGGTACCTCTA

'LJP_m2_A' GTTCCGTATTCAAATCTAACCATCTTGCCTTGCCACCCGGTACCTCTA

'LJP_m1_B' GTTCCGTATTCAAATCTAACCATCTTGCCTTGCCACCCGGTACCTCTA

'LJP_m1_A' GTTCCGTATTCAAATCTAACCATCTTGCCTTGCCACCCGGTACCTCTA

'BR_m5_A' GTTCCGTATTCAAATCTAACCATCTTGCCTTGCCACCCGGTACCTCTA

'BR_m5_B' GTTCCGTATTCAAATCTAACCATCTTGCCTTGCCACCCGGTACCTCTA

'BR_m6_A' GTTCCGTATTCAAATCTAACCATCTTGCCTTGCCACCCGGTACCTCTA

'BR_m6_B' GTTCCGTATTCAAATCTAACCATCTTGCCTTGCCACCCGGTACCTCTA

'BR_m7_A' GTTCCGTATTCAAATCTAACCATCTTGCCTTGCCACCCGGTACCTCTA

'BR_m7_B' GTTCCGTATTCAAATCTAACCATCTTGCCTTGCCACCCGGTACCTCTA

'BR_f13_B' GTTCCGTATTCAAATCTAACCATCTTGCCTTGCCACCCGGTACCTCTA

'BR_f13_A' GTTCCGTATTCAAATCTAACCATCTTGCCTTGCCACCCGGTACCTCTA

'BR_m8_B' GTTCCGTATTCAAATCTAACCATCTTGCCTTGCCACCCGGTACCTCTA

'BR_m8_A' GTTCCGTATTCAAATCTAACCATCTTGCCTTGCCACCCGGTACCTCTA

'SD_h_a' GTTCCGTATTCAAATCTAACCATCTTGCCTTGCCACCCGGTACCTCTA

'SD_h_b' GTTCCGTATTCAAATCTAACCATCTTGCCTTGCCACCCGGTACCTCTA

'SD_g_a' GTTCCGTATTCAAATCTAACCATCTTGCCTTGCCACCCGGTACCTCTA

'SD_g_b' GTTCCGTATTCAAATCTAACCATCTTGCCTTGCCACCCGGTACCTCTA

'SD_2m_b' GTTCCGTATTCAAATCTAACCATCTTGCCTTGCCACCCGGTACCTCTA

'SD_2m_a' GTTCCGTATTCAAATCTAACCATCTTGCCTTGCCACCCGGTACCTCTA

[ 1010 1020 1030 1040 1050 ]

[ * * * * * ]

'RP1_m1_A' ACCGTCGAAGCCAAAGCCCCAAGTGCATAGTCCGCCACATTTATTATA

'RP1_m1_B' ACCGTCGAAGCCAAAGCCCCAAGTGCATAGTCCGCCACATTTATTATA

'RP1_m5(A)' ACCGTCGAAGCCAAAGCCCCAAGTGCATAGTCCGCCACATTTATTATA

'RP1_m5_B' ACCGTCGAAGCCAAAGCCCCAAGTGCATAGTCCGCCACATTTATTATA

'RP1_m7_A' ACCGTCGAAGCCAAAGCCCCAAGTGCATAGTCCGCCACATTTATTATA

'RP1_m7_B' ACCGTCGAAGCCAAAGCCCCAAGTGCATAGTCCGCCACATTTATTATA

'RP1_m8_A' ACCGTCGAAGCCAAAGCCCCAAGTGCATAGTCCGCCACATTTATTATA

'RP1_m8_B' ACCGTCGAAGCCAAAGCCCCAAGTGCATAGTCCGCCACATTTATTATA

'R_Abg' ACCGTCGAAGCCAAAGCCCCAAGTGCATAGTCCGCCACATTTATTATA

'R_Abg_2' ACCGTCGAAGCCAAAGCCCCAAGTGCATAGTCCGCCACATTTATTATA

'AB_m1_A' ACCGTCGAAGCCAAAGCCCCAAGTGCATAGTCCGCCACATTTATTATA

'AB_m1_B' ACCGTCGAAGCCAAAGCCCCAAGTGCATAGTCCGCCACATTTATTATA

Abh ACCGTCGAAGCCAAAGCCCCAAGTGCATAGTCCGCCACATTTATTATA

'Abh_2' ACCGTCGAAGCCAAAGCCCCAAGTGCATAGTCCGCCACATTTATTATA

'AB_m2_A' ACCGTCGAAGCCAAAGCCCCAAGTGCATAGTCCGCCACATTTATTATA

'AB_m2_B' ACCGTCGAAGCCAAAGCCCCAAGTGCATAGTCCGCCACATTTATTATA

'IP_m8_A' ACCGTCGAAGCCAAAGCCCCAAGTGCATAGTCCGCCACATTTATTATA

'IP_m8_B' ACCGTCGAAGCCAAAGCCCCAAGTGCATAGTCCGCCACATTTATTATA

'IP_f3_A' ACCGTCGAAGCCAAAGCCCCAAGTGCATAGTCCGCCACATTTATTATA

'IP_f3_B' ACCGTCGAAGCCAAAGCCCCAAGTGCATAGTCCGCCACATTTATTATA

'IP_f23_A' ACCGTCGAAGCCAAAGCCCCAAGTGCATAGTCCGCCACATTTATTATA

'IP_f23_B' ACCGTCGAAGCCAAAGCCCCAAGTGCATAGTCCGCCACATTTATTATA

'IP_m7_A' ACCGTCGAAGCCAAAGCCCCAAGTGCATAGTCCGCCACATTTATTATA

'IP_m7_B' ACCGTCGAAGCCAAAGCCCCAAGTGCATAGTCCGCCACATTTATTATA

'PVL_f14_A' ACCGTCGAAGCCAAAGCCCCAAGTGCATAGTCCGCCACATTTATTATA

'PVL_f14_B' ACCGTCGAAGCCAAAGCCCCAAGTGCATAGTCCGCCACATTTATTATA

'PVL_f12_A' ACCGTCGAAGCCAAAGCCCCAAGTGCATAGTCCGCCACATTTATTATA

'PVL_f12_B' ACCGTCGAAGCCAAAGCCCCAAGTGCATAGTCCGCCACATTTATTATA

'PVL_f3_A' ACCGTCGAAGCCAAAGCCCCAAGTGCATAGTCCGCCACATTTATTATA

'PVL_f3_B' ACCGTCGAAGCCAAAGCCCCAAGTGCATAGTCCGCCACATTTATTATA

'PVL_f7_A' ACCGTCGAAGCCAAAGCCCCAAGTGCATAGTCCGCCACATTTATTATA

'PVL_f7_B' ACCGTCGAAGCCAAAGCCCCAAGTGCATAGTCCGCCACATTTATTATA

'Pes_m9_A' TCCGTCGAAGCCAAAGCCCCAAAGGCATAGTCAGCCACATTTATTATA

'Pes_m9_B' TCCGTCGAAGCCAAAGCCCCAAAGGCATAGTCAGCCACATTTATTATA

'Pes_m10_A' TCCGTCGAAGCCAAAGCCCCAAAGGCATAGTCAGCAACATTTATTATA

'Pes_m10_B' TCCGTCGAAGCCAAAGCCCCAAAGGCATAGTCAGCCACATTTATTATA

'LH_m1_A' TCCGTCGAAGCCAAAGCCCCAAGGGCATAGTCAGCCACATTTATTATA

'LH_m1_B' TCCGTCGAAGCCAAAGCCCCAAGGGCATAGTCAGCCACATTTATTATA

'LH_f6_A' TCCGTCGAAGCCAAAGCCCCAAGGGCATAGTCAGCCACATTTATTATA

'LH_f6_B' TCCGTCGAAGCCAAAGCCCCAAGGGCATAGTCAGCCACATTTATTATA

'LH_f8_A' TCCGTCGAAGCCAAAGCCCCAAGGGCATAGTCAGCCACATTTATTATA

'LH_f8_B' TCCGTCGAAGCCAAAGCCCCAAGGGCATAGTCAGCCACATTTATTATA

'scn_h_a' TCCGTCGAAGCCAAAGCCCCAAGGGCATAGTCAGCCACATTTATTATA

'scn_h_b' TCCGTCGAAGCCAAAGCCCCAAGGGCATAGTCAGCCACATTTATTATA

'scn_m5_A' TCCGTCGAAGCCAAAGCCCCAAGGGCATAGTCAGCCACATTTATTATA

'scn_m5_B' TCCGTCGAAGCCAAAGCCCCAAGGGCATAGTCAGCCACATTTATTATA

'scn_m7_A' TCCGTCGAAGCCAAAGCCCCAAGGGCATAGTCAGCCACATTTATTATA

'scn_m7_B' TCCGTCGAAGCCAAAGCCCCAAGGGCATAGTCAGCCACATTTATTATA

'scn_m3_A' TCCGTCGAAGCCAAAGCCCCAAGGGCATAGTCAGCCACATTTATTATA

'scn_m3_B' TCCGTCGAAGCCAAAGCCCCAAGGGCATAGTCAGCCACATTTATTATA

'LJS_m1_A' TCCGTCGAAGCCAAAGCCCCAAGGACATAGTCAGCCACATTTATTATA

'LJS_m1_B' TCCGTCGAAGCCAAAGCCCCAAGGACATAGTCAGCCACATTTATTATA

'LJS_m2_A' TCCGTCGAAGCCAAAGCCCCAAGGACATAGTCAGCCACATTTATTATA

'LJS_m2_B' TCCGTCGAAGCCAAAGCCCCAAGGACATAGTCAGCCACATTTATTATA

'LJS_f10_A' TCCGTCGAAGCCAAAGCCCCAAGGACATAGTCAGCCACATTTATTATA

'LJS_f10_B' TCCGTCGAAGCCAAAGCCCCAAGGACATAGTCAGCCACATTTATTATA

'LJS_f9_B' TCCGTCGAAGCCAAAGCCCCAAGGACATAGTCAGCCACATTTATTATA

'LJS_f9_A' TCCGTCGAAGCCAAAGCCCCAAGGACATAGTCAGCCACATTTATTATA

'LJP_m7_B' TCCGTCGAAGCCAAAGCCCCAAGGACATAGTCAGCCACATTTATTATA

'LJP_m7_A' TCCGTCGAAGCCAAAGCCCCAAGGACATAGTCAGCCACATTTATTATA

'LJP_m6_B' TCCGTCGAAGCCAAAGCCCCAAGGACATAGTCAGCCACATTTATTATA

'LJP_m6_A' TCCGTCGAAGCCAAAGCCCCAAGGACATAGTCAGCCACATTTATTATA

'LJP_m2_B' TCCGTCGAAGCCAAAGCCCCAAGGACATAGTCAGCCACATTTATTATA

'LJP_m2_A' TCCGTCGAAGCCAAAGCCCCAAGGACATAGTCAGCCACATTTATTATA

'LJP_m1_B' TCCGTCGAAGCCAAAGCCCCAAGGACATAGTCAGCCACATTTATTATA

'LJP_m1_A' TCCGTCGAAGCCAAAGCCCCAAGGACATAGTCAGCCACATTTATTATA

'BR_m5_A' TCCGTCGAAGCCAAAGCCCCAAGGACATAGTCAGCCACATTTATTATA

'BR_m5_B' TCCGTCGAAGCCAAAGCCCCAAGGACATAGTCAGCCACATTTATTATA

'BR_m6_A' TCCGTCGAAGCCAAAGCCCCAAGGACATAGTCAGCCACATTTATTATA

'BR_m6_B' TCCGTCGAAGCCAAAGCCCCAAGGACATAGTCAGCCACATTTATTATA

'BR_m7_A' TCCGTCGAAGCCAAAGCCCCAAGGACATAGTCAGCCACATTTATTATA

'BR_m7_B' TCCGTCGAAGCCAAAGCCCCAAGGACATAGTCAGCCACATTTATTATA

'BR_f13_B' TCCGTCGAAGCCAAAGCCCCAAGGACATAGTCAGCCACATTTATTATA

'BR_f13_A' TCCGTCGAAGCCAAAGCCCCAAGGACATAGTCAGCCACATTTATTATA

'BR_m8_B' TCCGTCGAAGCCAAAGCCCCAAGGACATAGTCAGCCACATTTATTATA

'BR_m8_A' TCCGTCGAAGCCAAAGCCCCAAGGACATAGTCAGCCACATTTATTATA

'SD_h_a' TCCGTCGAAGCCAAAGCCCCAAAGACATAGTCAGCCACATTTATTATA

'SD_h_b' TCCGTCGAAGCCAAAGCCCCAAAGACATAGTCAGCCACATTTATTATA

'SD_g_a' TCCGTCGAAGCCAAAGCCCCAAAGACATAGTCAGCCACATTTATTATA

'SD_g_b' TCCGTCGAAGCCAAAGCCCCAAGGACATAGTCAGCCACATTTATTATA

'SD_2m_b' TCCGTCGAAGCCAAAGCCCCAAAGACATAGTCAGCCACATTTATTATA

'SD_2m_a' TCCGTCGAAGCCAAAGCCCCAAAGACATAGTCAGCCACATTTATTATA

[ 1060 1070 1080 1090 1100 ]

[ * * * * * ]

'RP1_m1_A' GGATTGAATGGCGGGCGGAATTTGGTACTTCACTGCTTCGGCCACTCA

'RP1_m1_B' GGATTGAATGGCGGGCGGAATTTGGTACTTCACTGCTTCGGCCACTCA

'RP1_m5(A)' GGATTGAATGGCGGGCGGAATTTGGTACTTCACTGCTTCGGCCACTCA

'RP1_m5_B' GGATTGAATGGCGGGCGGAATTTGGTACTTCACTGCTTCGGCCACTCA

'RP1_m7_A' GGATTGAATGGCGGGCGGAATTTGGTACTTCACTGCTTCGGCCACTCA

'RP1_m7_B' GGATTGAATGGCGGGCGGAATTTGGTACTTCACTGCTTCGGCCACTCA

'RP1_m8_A' GGATTGAATGGCGGGCGGAATTTGGTACTTCACTGCTTCGGCCACTCA

'RP1_m8_B' GGATTGAATGGCGGGCGGAATTTGGTACTTCACTGCTTCGGCCACTCA

'R_Abg' GGATTGAATGGCGGGCGGAATTTGGTACTTCACTGCTTCGGCCACTCA

'R_Abg_2' GGATTGAATGGCGGGCGGAATTTGGTACTTCACTGCTTCGGCCACTCA

'AB_m1_A' GGATTGAATGGCGGGCGGAATTTGGTACTTCACTGCTTCGGCCACTCA

'AB_m1_B' GGATTGAATGGCGGGCGGAATTTGGTACTTCACTGCTTCGGCCACTCA

Abh GGATTGAATGGCGGGCGGAATTTGGTACTTCACTGCTTCGGCCACTCA

'Abh_2' GGATTGAATGGCGGGCGGAATTTGGTACTTCACTGCTTCGGCCACTCA

'AB_m2_A' GGATTGAATGGCGGGCGGAATTTGGTACTTCACTGCTTCGGCCACTCA

'AB_m2_B' GGATTGAATGGCGGGCGGAATTTGGTACTTCACTGCTTCGGCCACTCA

'IP_m8_A' GGATTGAATGGCGGGCGGAATTTGGTACTTCACTGCTTCGGCCACTCA

'IP_m8_B' GGATTGAATGGCGGGCGGAATTTGGTACTTCACTGCTTCGGCCACTCA

'IP_f3_A' GGATTGAATGGCGGGCGGAATTTGGTACTTCACTGCTTCGGCCACTCA

'IP_f3_B' GGATTGAATGGCGGGCGGAATTTGGTACTTCACTGCTTCGGCCACTCA

'IP_f23_A' GGATTGAATGGCGGGCGGAATTTGGTACTTCACTGCTTCGGCCACTCA

'IP_f23_B' GGATTGAATGGCGGGCGGAATTTGGTACTTCACTGCTTCGGCCACTCA

'IP_m7_A' GGATTGAATGGCGGGCGGAATTTGGTACTTCACTGCTTCGGCCACTCA

'IP_m7_B' GGATTGAATGGCGGGCGGAATTTGGTACTTCACTGCTTCGGCCACTCA

'PVL_f14_A' GGATTGAATGGCGGGCGGAATTTGGTACTTCACTGCTTCGGCCACTCA

'PVL_f14_B' GGATTGAATGGCGGGCGGAATTTGGTACTTCACTGCTTCGGCCACTCA

'PVL_f12_A' GGATTGAATGGCGGGCGGAATTTGGTACTTCACTGCTTCGGCCACTCA

'PVL_f12_B' GGATTGAATGGCGGGCGGAATTTGGTACTTCACTGCTTCGGCCACTCA

'PVL_f3_A' GGATTGAATGGCGGGCGGAATTTGGTACTTCACTGCTTCGGCCACTCA

'PVL_f3_B' GGATTGAATGGCGGGCGGAATTTGGTACTTCACTGCTTCGGCCACTCA

'PVL_f7_A' GGATTGAATGGCGGGCGGAATTTGGTACTTCACTGCTTCGGCCACTCA

'PVL_f7_B' GGATTGAATGGCGGGCGGAATTTGGTACTTCACTGCTTCGGCCACTCA

'Pes_m9_A' TGATTGAATGGCGGGCGGAATTTGGTACTTAACTGTTTGGGCGGCTAA

'Pes_m9_B' TGATTGAATGGCGGGCGGAATTTGGTACTTAACTGTTTGGGCGGCTAA

'Pes_m10_A' TGATTGAATGGCGGGCGGAATTTGGTACTTAACTGTTTGGGCGGCTAA

'Pes_m10_B' TGATTGAATGGCGGGCGGAATTTGGTACTTGACTGTTTGGGCGGCTAA

'LH_m1_A' TGATTGAATGGCGGGCGGAATTTGGTACTTTACTGTTTGGGCCGCTAA

'LH_m1_B' TGATTGAATGGCGGGCGGAATTTGGTACTTTACTGTTTGGGCCGCTAA

'LH_f6_A' TGATTGAATGGCGGGCGGAATTTGGTACTTTACTGTTTGGGCCGCTAA

'LH_f6_B' TGATTGAATGGCGGGCGGAATTTGGTACTTTACTGTTTGGGCCGCTAA

'LH_f8_A' TGATTGAATGGCGGGCGGAATTTGGTACTTTACTGTTTGGGCCGCTAA

'LH_f8_B' TGATTGAATGGCGGGCGGAATTTGGTACTTTACTGTTTGGGCCGCTAA

'scn_h_a' TGATTGAATGGCGGGCGGAATTTGGTACTTTACTGTTTGGGCCGCTAA

'scn_h_b' TGATTGAATGGCGGGCGGAATTTGGTACTTTACTGTTTGGGCCGCTAA

'scn_m5_A' TGATTGAATGGCGGGCGGAATTTGGTACTTTACTGTTTGGGCCGCTAA

'scn_m5_B' TGATTGAATGGCGGGCGGAATTTGGTACTTTACTGTTTGGGCCGCTAA

'scn_m7_A' TGATTGAATGGCGGGCGGAATTTGGTACTTTACTGTTTGGGCCGCTAA

'scn_m7_B' TGATTGAATGGCGGGCGGAATTTGGTACTTTACTGTTTGGGCCGCTAA

'scn_m3_A' TGATTGAATGGCGGGCGGAATTTGGTACTTTACTGTTTGGGCCGCTAA

'scn_m3_B' TGATTGAATGGCGGGCGGAATTTGGTACTTTACTGTTTGGGCCGCTAA

'LJS_m1_A' GGATTGAATGGCGGGCGGAATTTGGTACTTAATTGCTTCGGCTACTAA

'LJS_m1_B' GGATTGAATGGCGGGCGGAATTTGGTACTTAATTGCTTCGGCTACTAA

'LJS_m2_A' GGATTGAATGGCGGGCGGAATTTGGTACTTTATTGCTTCGGCTACTAA

'LJS_m2_B' GGATTGAATGGCGGGCGGAATTTGGTACTTTATTGCTTCGGCTACTAA

'LJS_f10_A' GGATTGAATGGCGGGCGGAATTTGGTACTTTATTGCTTCGGCTACTAA

'LJS_f10_B' GGATTGAATGGCGGGCGGAATTTGGTACTTTATTGCTTCGGCTACTAA

'LJS_f9_B' GGATTGAATGGCGGGCGGAATTTGGTACTTAATTGCTTCGGCTACTAA

'LJS_f9_A' GGATTGAATGGCGGGCGGAATTTGGTACTTAATTGCTTCGGCTACTAA

'LJP_m7_B' GGATTGAATGGCGGGCGGAATTTGGTACTTTATTGCTTCGGCTACTAA

'LJP_m7_A' GGATTGAATGGCGGGCGGAATTTGGTACTTTATTGCTTCGGCTACTAA

'LJP_m6_B' GGATTGAATGGCGGGCGGAATTTGGTACTTAATTGCTTCGGCTACTAA

'LJP_m6_A' GGATTGAATGGCGGGCGGAATTTGGTACTTTATTGCTTCGGCTACTAA

'LJP_m2_B' GGATTGAATGGCGGGCGGAATTTGGTACTTTATTGCTTCGGCTACTAA

'LJP_m2_A' GGATTGAATGGCGGGCGGAATTTGGTACTTTATTGCTTCGGCTACTAA

'LJP_m1_B' GGATTGAATGGCGGGCGGAATTTGGTACTTAATTGCTTCGGCTACTAA

'LJP_m1_A' GGATTGAATGGCGGGCGGAATTTGGTACTTAATTGCTTCGGCTACTAA

'BR_m5_A' GGATTGAATGGCGGGCGGAATTTGGTACTTTATTGCTTCGGCTACTAA

'BR_m5_B' GGATTGAATGGCGGGCGGAATTTGGTACTTAATTGCTTCGGCTACTAA

'BR_m6_A' GGATTGAATGGCGGGCGGAATTTGGTACTTTATTGCTTCGGCTACTAA

'BR_m6_B' GGATTGAATGGCGGGCGGAATTTGGTACTTAATTGCTTCGGCTACTAA

'BR_m7_A' GGATTGAATGGCGGGCGGAATTTGGTACTTAATTGCTTCGGCTACTAA

'BR_m7_B' GGATTGAATGGCGGGCGGAATTTGGTACTTAATTGCTTCGGCTACTAA

'BR_f13_B' GGATTGAATGGCGGGCGGAATTTGGTACTTAATTGCTTCGGCTACTAA

'BR_f13_A' GGATTGAATGGCGGGCGGAATTTGGTACTTAATTGCTTCGGCTACTAA

'BR_m8_B' GGATTGAATGGCGGGCGGAATTTGGTACTTAATTGCTTCGGCTACTAA

'BR_m8_A' GGATTGAATGGCGGGCGGAATTTGGTACTTTATTGCTTCGGCTACTAA

'SD_h_a' GGATTGAATGGCGGGCGGAATTTGGTACTTTATTGCTTCGGCTACTAA

'SD_h_b' GGATTGAATGGCGGGCGGAATTTGGTACTTTATTGCTTCGGCTACTAA

'SD_g_a' GGATTGAATGGCGGGCGGAATTTGGTACTTTATTGCTTCGGCTACTAA

'SD_g_b' GGATTGAATGGCGGGCGGAATTTGGTACTTTATTGCTTCGGCTACTAA

'SD_2m_b' GGATTGAATGGCGGGCGGAATTTGGTACTTTATTGCTTCGGCTACTAA

'SD_2m_a' GGATTGAATGGCGGGCGGAATTTGGTACTTTATTGCTTCGGCTACTAA

[ 1110 1120 1130 1140 1150 ]

[ * * * * * ]

'RP1_m1_A' AAGTTGCGATGGTATTGCTAAAATAGGTGTTCATTACCTGGCTAAAAC

'RP1_m1_B' AAGTTGCGATGGTATTGCTAAAATAGGTGTTCATTACCTGGCTAAAAC

'RP1_m5(A)' AAGTTGCGATGGTATTGCTAAAATAGGTGTTCATTACCTGGCTAAAAC

'RP1_m5_B' AAGTTGCGATGGTATTGCTAAAATAGGTGTTCATTACCTGGCTAAAAC

'RP1_m7_A' AAGTTGCGATGGTATTGCTAAAATAGGTGTTCATTACCTGGCTAAAAC

'RP1_m7_B' AAGTTGCGATGGTATTGCTAAAATAGGTGTTCATTACCTGGCTAAAAC

'RP1_m8_A' AAGTTGCGATGGTATTGCTAAAATAGGTGTTCATTACCTGGCTAAAAC

'RP1_m8_B' AAGTTGCGATGGTATTGCTAAAATAGGTGTTCATTACCTGGCTAAAAC

'R_Abg' AAGTTGCGATGGTATTGCTAAAATAGGTGTTCATTACCTGGCTAAAAC

'R_Abg_2' AAGTTGCGATGGTATTGCTAAAATAGGTGTTCATTACCTGGCTAAAAC

'AB_m1_A' AAGTTGCGATGGTATTGCTAAAATAGGTGTTCATTACCTGGCTAAAAC

'AB_m1_B' AAGTTGCGATGGTATTGCTAAAATAGGTGTTCATTACCTGGCTAAAAC

Abh AAGTTGCGATGGTATTGCTAAAATAGGTGTTCATTACCTGGCTAAAAC

'Abh_2' AAGTTGCGATGGTATTGCTAAAATAGGTGTTCATTACCTGGCTAAAAC

'AB_m2_A' AAGTTGCGATGGTATTGCTAAAATAGGTGTTCATTACCTGGCTAAAAC

'AB_m2_B' AAGTTGCGATGGTATTGCTAAAATAGGTGTTCATTACCTGGCTAAAAC

'IP_m8_A' AAGTTGCGATGGTATTGCTAAAATAGGTGTTCATTACCTGGCTAAAAC

'IP_m8_B' AAGTTGCGATGGTATTGCTAAAATAGGTGTTCATTACCTGGCTAAAAC

'IP_f3_A' AAGTTGCGATGGTATTGCTAAAATAGGTGTTCATTACCTGGCTAAAAC

'IP_f3_B' AAGTTGCGATGGTATTGCTAAAATAGGTGTTCATTACCTGGCTAAAAC

'IP_f23_A' AAGTTGCGATGGTATTGCTAAAATAGGTGTTCATTACCTGGCTAAAAC

'IP_f23_B' AAGTTGCGATGGTATTGCTAAAATAGGTGTTCATTACCTGGCTAAAAC

'IP_m7_A' AAGTTGCGATGGTATTGCTAAAATAGGTGTTCATTACCTGGCTAAAAC

'IP_m7_B' AAGTTGCGATGGTATTGCTAAAATAGGTGTTCATTACCTGGCTAAAAC

'PVL_f14_A' AAGTTGCGATGGTATTGCTAAAATAGGTGTTCATTACCTGGCTAAAAC

'PVL_f14_B' AAGTTGCGATGGTATTGCTAAAATAGGTGTTCATTACCTGGCTAAAAC

'PVL_f12_A' AAGTTGCGATGGTATTGCTAAAATAGGTGTTCATTACCTGGCTAAAAC

'PVL_f12_B' AAGTTGCGATGGTATTGCTAAAATAGGTGTTCATTACCTGGCTAAAAC

'PVL_f3_A' AAGTTGCGATGGTATTGCTAAAATAGGTGTTCATTACCTGGCTAAAAC

'PVL_f3_B' AAGTTGCGATGGTATTGCTAAAATAGGTGTTCATTACCTGGCTAAAAC

'PVL_f7_A' AAGTTGCGATGGTATTGCTAAAATAGGTGTTCATTACCTGGCTAAAAC

'PVL_f7_B' AAGTTGCGATGGTATTGCTAAAATAGGTGTTCATTACCTGGCTAAAAC

'Pes_m9_A' AAGTTGCGATGGTATTGGTAAAATAGGTGTTAATTACATGGCTAAAAC

'Pes_m9_B' AAGTTGCGATGGTATTGGTAAAATAGGTGTTAATTACATGGCTAAAAC

'Pes_m10_A' AAGTTGCGATGGTATTGGTAAAATAGGTGTTAATTACATGGCTAAAAC

'Pes_m10_B' AAGTTGCGATGGTATTGGTAAAATAGGTGTTAATTACATGGCTAAAAC

'LH_m1_A' AAGTTGCGATGGTATTGGTAAAATCGGTGTTAATTACATGGCTAAAAC

'LH_m1_B' AAGTTGCGATGGTATTGGTAAAATCGGTGTTAATTACATGGCTAAAAC

'LH_f6_A' AAGTTGCGATGGTATTGGTAAAATCGGTGTTAATTACATGGCTAAAAC

'LH_f6_B' AAGTTGCGATGGTATTGGTAAAATCGGTGTTAATTACATGGCTAAAAC

'LH_f8_A' AAGTTGCGATGGTATTGGTAAAATCGGTGTTAATTACATGGCTAAAAC

'LH_f8_B' AAGTTGCGATGGTATTGGTAAAATCGGTGTTAATTACATGGCTAAAAC

'scn_h_a' AAGTTGCGATGGTATTGGTAAAATCGGTGTTAATTACATGGCTAAAAC

'scn_h_b' AAGTTGCGATGGTATTGGTAAAATCGGTGTTAATTACATGGCTAAAAC

'scn_m5_A' AAGTTGCGATGGTATTGGTAAAATCGGTGTTAATTACATGGCTAAAAC

'scn_m5_B' AAGTTGCGATGGTATTGGTAAAATCGGTGTTAATTACATGGCTAAAAC

'scn_m7_A' AAGTTGCGATGGTATTGGTAAAATCGGTGTTAATTACATGGCTAAAAC

'scn_m7_B' AAGTTGCGATGGTATTGGTAAAATCGGTGTTAATTACATGGCTAAAAC

'scn_m3_A' AAGTTGCGATGGTATTGGTAAAATCGGTGTTAATTACATGGCTAAAAC

'scn_m3_B' AAGTTGCGATGGTATTGGTAAAATCGGTGTTAATTACATGGCTAAAAC

'LJS_m1_A' AAGTTGCGATGGTATTGCTAAAATAGGTGTTAATTACCTGGCTAAAAC

'LJS_m1_B' AAGTTGCGATGGTATTGCTAAAATAGGTGTTAATTACCTGGCTAAAAC

'LJS_m2_A' AAGTTGCGATGGTATTGCTAAAATAGGTGTTAATTACCTGGCTAAAAC

'LJS_m2_B' AAGTTGCGATGGTATTGCTAAAATAGGTGTTAATTACCTGGCTAAAAC

'LJS_f10_A' AAGTTGCGATGGTATTGCTAAAATAGGTGTTAATTACCTGGCTAAAAC

'LJS_f10_B' AAGTTGCGATGGTATTGCTAAAATAGGTGTTAATTACCTGGCTAAAAC

'LJS_f9_B' AAGTTGCGATGGTATTGCTAAAATAGGTGTTAATTACCTGGCTAAAAC

'LJS_f9_A' AAGTTGCGATGGTATTGCTAAAATAGGTGTTAATTACCTGGCTAAAAC

'LJP_m7_B' AAGTTGCGATGGTATTGCTAAAATAGGTGTTAATTACCTGGCTAAAAC

'LJP_m7_A' AAGTTGCGATGGTATTGCTAAAATAGGTGTTAATTACCTGGCTAAAAC

'LJP_m6_B' AAGTTGCGATGGTATTGCTAAAATAGGTGTTAATTACCTGGCTAAAAC

'LJP_m6_A' AAGTTGCAATGGTATTGCTAAAATAGGTGTTAATTACCTGGCTAAAAC

'LJP_m2_B' AAGTTGCGATGGTATTGCTAAAATAGGTGTTAATTACCTGGCTAAAAC

'LJP_m2_A' AAGTTGCGATGGTATTGCTAAAATAGGTGTTAATTACCTGGCTAAAAC

'LJP_m1_B' AAGTTGCGATGGTATTGCTAAAATAGGTGTTAATTACCTGGCTAAAAC

'LJP_m1_A' AAGTTGCGATGGTATTGCTAAAATAGGTGTTAATTACCTGGCTAAAAC

'BR_m5_A' AAGTTGCGATGGTATTGCTAAAATAGGTGTTAATTACCTGGCTAAAAC

'BR_m5_B' AAGTTGCGATGGTATTGCTAAAATAGGTGTTAATTACCTGGCTAAAAC

'BR_m6_A' AAGTTGCGATGGTATTGCTAAAATAGGTGTTAATTACCTGGCTAAAAC

'BR_m6_B' AAGTTGCGATGGTATTGCTAAAATAGGTGTTAATTACCTGGCTAAAAC

'BR_m7_A' AAGTTGCGATGGTATTGCTAAAATAGGTGTTAATTACCTGGCTAAAAC

'BR_m7_B' AAGTTGCGATGGTATTGCTAAAATAGGTGTTAATTACCTGGCTAAAAC

'BR_f13_B' AAGTTGCGATGGTATTGCTAAAATAGGTGTTAATTACCTGGCTAAAAC

'BR_f13_A' AAGTTGCGATGGTATTGCTAAAATAGGTGTTAATTACCTGGCTAAAAC

'BR_m8_B' AAGTTGCGATGGTATTGCTAAAATAGGTGTTAATTACCTGGCTAAAAC

'BR_m8_A' AAGTTGCGATGGTATTGCTAAAATAGGTGTTAATTACCTGGCTAAAAC

'SD_h_a' AAGTTGCGATGGTATTGCTAAAATAGGTGTTAATTACCTGGCTAAAAC

'SD_h_b' AAGTTGCGATGGTATTGCTAAAATAGGTGTTAATTACCTGGCTAAAAC

'SD_g_a' AAGTTGCGATGGTATTGCTAAAATAGGTGTTAATTACCTGGCTAAAAC

'SD_g_b' AAGTTGCGATGGTATTGCTAAAATAGGTGTTAATTACCTGGCTAAAAC

'SD_2m_b' AAGTTGCGATGGTATTGCTAAAATAGGTGTTAATTACCTGGCTAAAAC

'SD_2m_a' AAGTTGCGATGGTATTGCTAAAATAGGTGTTAATTACCTGGCTAAAAC

[ 1160 1170 1180 1190 1200]

[ * * * * *]

'RP1_m1_A' GGTTTAATGCGAACCACCGATGAAAGAAAAA----TAACCCTGGTGAC

'RP1_m1_B' GGTTTAATGCGAACCACCGATGAAAGAAAAA----TAACCCTGGTGAC

'RP1_m5(A)' GGTTTAATGCGAACCACCGATGAAAGAAAAA----TAACCCTGGTGAC

'RP1_m5_B' GGTTTAATGCGAACCACCGATGAAAGAAAAA----TAACCCTGGTGAC

'RP1_m7_A' GGTTTAATGCGAACCACCGATGAAAGAAAAA----TAACCCTGGTGAC

'RP1_m7_B' GGTTTAATGCGAACCACCGATGAAAGAAAAA----TAACCCTGGTGAC

'RP1_m8_A' GGTTTAATGCGAACCACCGATGAAAGAAAAA----TAACCCTGGTGAC

'RP1_m8_B' GGTTTAATGCGAACCACCGATGAAAGAAAAA----TAACCCTGGTGAC

'R_Abg' GGTTTAATGCGAACCACCGATGAAAGAAAAA----TAACCCTGGTGAC

'R_Abg_2' GGTTTAATGCGAACCACCGATGAAAGAAAAA----TAACCCTGGTGAC

'AB_m1_A' GGTTTAATGCGAACCACCGATGAAAGAAAAA----TAACCCTGGTGAC

'AB_m1_B' GGTTTAATGCGAACCACCGATGAAAGAAAAA----TAACCCTGGTGAC

Abh GGTTTAATGCGAACCACCGATGAAAGAAAAA----TAACCCTGGTGAC

'Abh_2' GGTTTAATGCGAACCACCGATGAAAGAAAAA----TAACCCTGGTGAC

'AB_m2_A' GGTTTAATGCGAACCACCGATGAAAGAAAAA----TAACCCTGGTGAC

'AB_m2_B' GGTTTAATGCGAACCACCGATGAAAGAAAAA----TAACCCTGGTGAC

'IP_m8_A' GGTTTAATGCGAACCACCGATGAAAGAAAAA----TAACCCTGGTGAC

'IP_m8_B' GGTTTAATGCGAACCACCGATGAAAGAAAAA----TAACCCTGGTGAC

'IP_f3_A' GGTTTAATGCGAACCACCGATGAAAGAAAAA----TAACCCTGGTGAC

'IP_f3_B' GGTTTAATGCGAACCACCGATGAAAGAAAAA----TAACCCTGGTGAC

'IP_f23_A' GGTTTAATGCGAACCACCGATGAAAGAAAAA----TAACCCTGGTGAC

'IP_f23_B' GGTTTAATGCGAACCACCGATGAAAGAAAAA----TAACCCTGGTGAC

'IP_m7_A' GGTTTAATGCGAACCACCGATGAAAGAAAAA----TAACCCTGGTGAC

'IP_m7_B' GGTTTAATGCGAACCACCGATGAAAGAAAAA----TAACCCTGGTGAC

'PVL_f14_A' GGTTTAATGCGAACCACCGATGAAAGAAAAA----TAACCCTGGTGAC

'PVL_f14_B' GGTTTAATGCGAACCACCGATGAAAGAAAAA----TAACCCTGGTGAC

'PVL_f12_A' GGTTTAATGCGAACCACCGATGAAAGAAAAA----TAACCCTGGTGAC

'PVL_f12_B' GGTTTAATGCGAACCACCGATGAAAGAAAAA----TAACCCTGGTGAC

'PVL_f3_A' GGTTTAATGCGAACCACCGATGAAAGAAAAA----TAACCCTGGTGAC

'PVL_f3_B' GGTTTAATGCGAACCACCGATGAAAGAAAAA----TAACCCTGGTGAC

'PVL_f7_A' GGTTTAATGCGAACCACCGATGAAAGAAAAA----TAACCCTGGTGAC

'PVL_f7_B' GGTTTAATGCGAACCACCGATGAAAGAAAAA----TAACCCTGGTGAC

'Pes_m9_A' GGTTTAGTGCGAACCACCGATGAAAGAATAATTAATAACCCTGCTGAC

'Pes_m9_B' GGTTTAGTGCGAACCACCGATGAAAGAATAATTAATAACCCTGCTGAC

'Pes_m10_A' GGTTTAGTGCGAACCACCGATGAAAGAATAATTAATAACCCTGCTGAC

'Pes_m10_B' GGTTTAGTGCGAACCACCGATGAAAGAATAATTAATAACCCTGCTGAC

'LH_m1_A' GGTTTAATGTGAACCACCGATGAAAGAATAATTAATAACCCTGCTGAC

'LH_m1_B' GGTTTAATGTGAACCACCGATGAAAGAATAATTAATAACCCTGCTGAC

'LH_f6_A' GGTTTAATGTGAACCACCGATGAAAGAATAATTAATAACCCTGCTGAC

'LH_f6_B' GGTTTAATGTGAACCACCGATGAAAGAATAATTAATAACCCTGCTGAC

'LH_f8_A' GGTTTAATGTGAACCACCGATGAAAGAATAATTAATAACCCTGCTGAC

'LH_f8_B' GGTTTAATGTGAACCACCGATGAAAGAATAATTAATAACCCTGCTGAC

'scn_h_a' GGTTTAATGTGAACCACCGATGAAAGAATAATTAATAACCCTGCTGAC

'scn_h_b' GGTTTAATGTGAACCACCGATGAAAGAATAATTAATAACCCTGCTGAC

'scn_m5_A' GGTTTAATGTGAACCACCGATGAAAGAATAATTAATAACCCTGCTGAC

'scn_m5_B' GGTTTAATGTGAACCACCGATGAAAGAATAATTAATAACCCTGCTGAC

'scn_m7_A' GGTTTAATGTGAACCACCGATGAAAGAATAATTAATAACCCTGCTGAC

'scn_m7_B' GGTTTAATGTGAACCACCGATGAAAGAATAATTAATAACCCTGCTGAC

'scn_m3_A' GGTTTAATGTGAACCACCGATGAAAGAATAATTAATAACCCTGCTGAC

'scn_m3_B' GGTTTAATGTGAACCACCGATGAAAGAATAATTAATAACCCTGCTGAC

'LJS_m1_A' GGTTTAATGCGAACCACCGATGAAAGAAAAAA---TAACCGTGCTGAC

'LJS_m1_B' GGTTTAATGCGAACCACCGATGAAAGAAAAAA---TAACCGTGCTGAC

'LJS_m2_A' GGTTTAATGCGAACCACCGATGAAAGAAAAA----TAACCGTGCTGAC

'LJS_m2_B' GGTTTAATGCGAACCACCGATGAAAGAAAAA----TAACCGTGCTGAC

'LJS_f10_A' GGTTTAATGCGAACCACCGATGAAAGAAAAA----TAACCGTGCTGAC

'LJS_f10_B' GGTTTAATGCGAACCACCGATGAAAGAAAAAA---TAACCGTGCTGAC

'LJS_f9_B' GGTTTAATGCGAACCACCGATGAAAGAAAAAA---TAACCGTGCTGAC

'LJS_f9_A' GGTTTAATGCGAACCACCGATGAAAGAAAAA----TAACCGTGCTGAC

'LJP_m7_B' GGTTTAATGCGAACCACCGATGAAAGAAAAA----TAACCGTGCTGAC

'LJP_m7_A' GGTTTAATGCGAACCACCGATGAAAGAAAAA----TAACCGTGCTGAC

'LJP_m6_B' GGTTTAATGCGAACCACCGATGAAAGAAAAA----TAACCGTGCTGAC

'LJP_m6_A' GGTTTAATGCGAACCACCGATGAAAGAAAAAA---TAACCGTGCTGAC

'LJP_m2_B' GGTTTAATGCGAACCACCGATGAAAGAAAAA----TAACCGTGCTGAC

'LJP_m2_A' GGTTTAATGCGAACCACCGATGAAAGAAAAA----TAACCGTGCTGAC

'LJP_m1_B' GGTTTAATGCGAACCACCGATGAAAGAAAAAA---TAACCGTGCTGAC

'LJP_m1_A' GGTTTAATGCGAACCACCGATGAAAGAAAAAA---TAACCGTGCTGAC

'BR_m5_A' GGTTTAATGCGAACCACCGATGAAAGAAAAA----TAACCGTGCTGAC

'BR_m5_B' GGTTTAATGCGAACCACCGATGAAAGAAAAAA---TAACCGTGCTGAC

'BR_m6_A' GGTTTAATGCGAACCACCGATGAAAGAAAAA----TAACCGTGCTGAC

'BR_m6_B' GGTTTAATGCGAACCACCGATGAAAGAAAAA----TAACCGTGCTGAC

'BR_m7_A' GGTTTAATGCGAACCACCGATGAAAGAAAAA----TAACCGTGCTGAC

'BR_m7_B' GGTTTAATGCGAACCACCGATGAAAGAAAAA----TAACCGTGCTGAC

'BR_f13_B' GGTTTAATGCGAACCACCGATGAAAGAAAAAA---TAACCGTGCTGAC

'BR_f13_A' GGTTTAATGCGAACCACCGATGAAAGAAAAA----TAACCGTGCTGAC

'BR_m8_B' GGTTTAATGCGAACCACCGATGAAAGAAAAA----TAACCGTGCTGAC

'BR_m8_A' GGTTTAATGCGAACCACCGATGAAAGAAAAA----TAACCGTGCTGAC

'SD_h_a' GGTTTAATGCGAACCACCGATGAAAGAAAAA----TAACCGTGCTGAC

'SD_h_b' GGTTTAATGCGAACCACCGATGAAAGAAAAA----TAACCGTGCTGAC

'SD_g_a' GGTTTAATGCGAACCACCGATGAAAGAAAAA----TAACCGTGCTGAC

'SD_g_b' GGTTTAATGCGAACCACCGATGAAAGAAAAA----TAACCGTGCTGAC

'SD_2m_b' GGTTTAATGCGAACCACCGATGAAAGAAAAA----TAACCGTGCTGAC

'SD_2m_a' GGTTTAATGCGAACCACCGATGAAAGAAAAA----TAACCGTGCTGAC

[ 1210 1220 1230 1240 ]

[ * * * * ]

'RP1_m1_A' TCAAGTTCTCCCACATTCCATTCTTTCAGTTTGCAGTCAGATCCGGTT

'RP1_m1_B' TCAAGTTCTCCCACATTCCATTCTTTCAGTTTGCAGTCAGATCCGGTT

'RP1_m5(A)' TCAAGTTCTCCCACATTCCATTCTTTCAGTTTGCAGTCAGATCCGGTT

'RP1_m5_B' TCAAGTTCTCCCACATTCCATTCTTTCAGTTTGCAGTCAGATCCGGTT

'RP1_m7_A' TCAAGTTCTCCCACATTCCATTCTTTCAGTTTGCAGTCAGATCCGGTT

'RP1_m7_B' TCAAGTTCTCCCACATTCCATTCTTTCAGTTTGCAGTCAGATCCGGTT

'RP1_m8_A' TCAAGTTCTCCCACATTCCATTCTTTCAGTTTGCAGTCAGATCCGGTT

'RP1_m8_B' TCAAGTTCTCCCACATTCCATTCTTTCAGTTTGCAGTCAGATCCGGTT

'R_Abg' TCAAGTTCTCCCACATTCCATTCTTTCAGTTTGCAGTCAGATCCGGTT

'R_Abg_2' TCAAGTTCTCCCACATTCCATTCTTTCAGTTTGCAGTCAGATCCGGTT

'AB_m1_A' TCAAGTTCTCCCACATTCCATTCTTTCAGTTTGCAGTCAGATCCGGTT

'AB_m1_B' TCAAGTTCTCCCACATTCCATTCTTTCAGTTTGCAGTCAGATCCGGTT

Abh TCAAGTTCTCCCACATTCCATTCTTTCAGTTTGCAGTCAGATCCGGTT

'Abh_2' TCAAGTTCTCCCACATTCCATTCTTTCAGTTTGCAGTCAGATCCGGTT

'AB_m2_A' TCAAGTTCTCCCACATTCCATTCTTTCAGTTTGCAGTCAGATCCGGTT

'AB_m2_B' TCAAGTTCTCCCACATTCCATTCTTTCAGTTTGCAGTCAGATCCGGTT

'IP_m8_A' TCAAGTTCTCCCACATTCCATTCTTTCAGTTTGCAGTCAGATCCGGTT

'IP_m8_B' TCAAGTTCTCCCACATTCCATTCTTTCAGTTTGCAGTCAGATCCGATT

'IP_f3_A' TCAAGTTCTCCCACATTCCATTCTTTCAGTTTGCAGTCAGATCCGGTT

'IP_f3_B' TCAAGTTCTCCCACATTCCATTCTTTCAGTTTGCAGTCAGATCCGGTT

'IP_f23_A' TCAAGTTCTCCCACATTCCATTCTTTCAGTTTGCAGTCAGATCCGGTT

'IP_f23_B' TCAAGTTCTCCCACATTCCATTCTTTCAGTTTGCAGTCAGATCCGGTT

'IP_m7_A' TCAAGTTCTCCCACATTCCATTCTTTCAGTTTGCAGTCAGATCCGGTT

'IP_m7_B' TCAAGTTCTCCCACATTCCATTCTTTCAGTTTGCAGTCAGATCCGGTT

'PVL_f14_A' TCAAGTTCTCCCACATTCCATTCTTTCAGTTTGCAGTCAGATCCGGTT

'PVL_f14_B' TCAAGTTCTCCCACATTCCATTCTTTCAGTTTGCAGTCAGATCCGGTT

'PVL_f12_A' TCAAGTTCTCCCACATTCCATTCTTTCAGTTTGCAGTCAGATCCGGTT

'PVL_f12_B' TCAAGTTCTCCCACATTCCATTCTTTCAGTTTGCAGTCAGATCCGGTT

'PVL_f3_A' TCAAGTTCTCCCACATTCCATTCTTTCAGTTTGCAGTCAGATCCGGTT

'PVL_f3_B' TCAAGTTCTCCCACATTCCATTCTTTCAGTTTGCAGTCAGATCCGGTT

'PVL_f7_A' TCAAGTTCTCCCACATTCCATTCTTTCAGTTTGCAGTCAGATCCGGTT

'PVL_f7_B' TCAAGTTCTCCCACATTCCATTCTTTCAGTTTGCAGTCAGATCCGGTT

'Pes_m9_A' TAAAGTTTTCCCAC-CTCCATTCTTTCAGTATGCAGTCAGATCCGGTT

'Pes_m9_B' TAAAGTTTTCCCAC-CTCCATTCTTTCAGTATGCAGTCAGATCCGGTT

'Pes_m10_A' TAAAGTTTTCCCAC-CTCCATTCTTTCAGTATGCAGTCAGATCCGGTT

'Pes_m10_B' TAAAGTTTTCCCAC-CTCCATTCTTTCAGTATGCAGTCAGATCCGGTT

'LH_m1_A' TAAAGTTTTCCCAC-CTCCATTCTTTCAGTATGCAGTCAGATCCGGTT

'LH_m1_B' TAAAGTTTTCCCAC-CTCCATTCTTTCAGTATGCAGTCAGATCCGGTT

'LH_f6_A' TAAAGTTTTCCCAC-CTCCATTCTTTCAGTATGCAGTCAGATCCGGTT

'LH_f6_B' TAAAGTTTTCCCAC-CTCCATTCTTTCAGTATGCAGTCAGATCCGGTT

'LH_f8_A' TAAAGTTTTCCCAC-CTCCATTCTTTCAGTATGCAGTCAGATCCGGTT

'LH_f8_B' TAAAGTTTTCCCAC-CTCCATTCTTTCAGTATGCAGTCAGATCCGGTT

'scn_h_a' TAAAGTTTTCCCAC-CTCCATTCTTTCAGTATGCAGTCAGATCCGGTT

'scn_h_b' TAAAGTTTTCCCAC-CTCCATTCTTTCAGTATGCAGTCAGATCCGGTT

'scn_m5_A' TAAAGTTTTCCCAC-CTCCATTCTTTCAGTATGCAGTCAGATCCGGTT

'scn_m5_B' TAAAGTTTTCCCAC-CTCCATTCTTTCAGTATGCAGTCAGATCCGGTT

'scn_m7_A' TAAAGTTTTCCCAC-CTCCATTCTTTCAGTATGCAGTCAGATCCGGTT

'scn_m7_B' TAAAGTTTTCCCAC-CTCCATTCTTTCAGTATGCAGTCAGATCCGGTT

'scn_m3_A' TAAAGTTTTCCCAC-CTCCATTCTTTCAGTATGCAGTCAGATCCGGTT

'scn_m3_B' TAAAGTTTTCCCAC-CTCCATTCTTTCAGTATGCAGTCAGATCCGGTT

'LJS_m1_A' TCAAGTTCTCCCACATTCGATTCTTTCAGTTTGCAGTCAGATCCGGTT

'LJS_m1_B' TCAAGTTCTCCCACATTCGATTCTTTCAGTTTGCAGTCAGATCCGGTT

'LJS_m2_A' TCAAGTTCTCCCACATTCGATTCTTTCAGTTTGCAGTCAGATCCGGTT

'LJS_m2_B' TCAAGTTCTCCCACATTCGATTCTTTCAGTTTGCAGTCAGATCCGGTT

'LJS_f10_A' TCAAGTTCTCCCACATTCGATTCTTTCAGTTTGCAGTCAGATCCGGTT

'LJS_f10_B' TCAAGTTCTCCCACATTCGATTCTTTCAGTTTGCAGTCAGATCCGGTT

'LJS_f9_B' TCAAGTTCTCCCACATTCGATTCTTTCAGTTTGCAGTCAGATCCGGTT

'LJS_f9_A' TCAAGTTCTCCCACATTCGATTCTTTCAGTTTGCAGTCAGATCCGGTT

'LJP_m7_B' TCAAGTTCTCCCACATTCGATTCTTTCAGTTTGCAGTCAGATCCGGTT

'LJP_m7_A' TCAAGTTCTCCCACATTCGATTCTTTCAGTTTGCAGTCAGATCCGGTT

'LJP_m6_B' TCAAGTTCTCCCACATTCGATTCTTTCAGTTTGCAGTCAGATCCGGTT

'LJP_m6_A' TCAAGTTCTCCCACATTCGATTCTTTCAGTTTGCAGTCAGATCCGGTT

'LJP_m2_B' TCAAGTTCTCCCACATTCGATTCTTTCAGTTTGCAGTCAGATCCGGTT

'LJP_m2_A' TCAAGTTCTCCCACATTCGATTCTTTCAGTTTGCAGTCAGATCCGGTT

'LJP_m1_B' TCAAGTTCTCCCACATTCGATTCTTTCAGTTTGCAGTCAGATCCGGTT

'LJP_m1_A' TCAAGTTCTCCCACATTCGATTCTTTCAGTTTGCAGTCAGATCCGGTT

'BR_m5_A' TCAAGTTCTCCCACATTCGATTCTTTCAGTTTGCAGTCAGATCCGGTT

'BR_m5_B' TCAAGTTCTCCCACATTCGATTCTTTCAGTTTGCAGTCAGATCCGGTT

'BR_m6_A' TCAAGTTCTCCCACATTCGATTCTTTCAGTTTGCAGTCAGATCCGGTT

'BR_m6_B' TCAAGTTCTCCCACATTCGATTCTTTCAGTTTGCAGTCAGATCCGGTT

'BR_m7_A' TCAAGTTCTCCCACATTCGATTCTTTCAGTTTGCAGTCAGATCCGGTT

'BR_m7_B' TCAAGTTCTCCCACATTCGATTCTTTCAGTTTGCAGTCAGATCCGGTT

'BR_f13_B' TCAAGTTCTCCCACATTCGATTCTTTCAGTTTGCAGTCAGATCCGGTT

'BR_f13_A' TCAAGTTCTCCCACATTCGATTCTTTCAGTTTGCAGTCAGATCCGGTT

'BR_m8_B' TCAAGTTCTCCCACATTCGATTCTTTCAGTTTGCAGTCAGATCCGGTT

'BR_m8_A' TCAAGTTCTCCCACATTCGATTCTTTCAGTTTGCAGTCAGATCCGGTT

'SD_h_a' TCAAGTTCTCCCACATTCGATTCTTTCAGTTTGCAGTCAGATCCGGTT

'SD_h_b' TCAAGTTCTCCCACATTCGATTCTTTCAGTTTGCAGTCAGATCCGGTT

'SD_g_a' TCAAGTTCTCCCACATTCGATTCTTTCAGTTTGCAGTCAGATCCGGTT

'SD_g_b' TCAAGTTCTCCCACATTCGATTCTTTCAGTTTGCAGTCAGATCCGGTT

'SD_2m_b' TCAAGTTCTCCCACATTCGATTCTTTCAGTTTGCAGTCAGATCCGGTT

'SD_2m_a' TCAAGTTCTCCCACATTCGATTCTTTCAGTTTGCAGTCAGATCCGGTT

[ 1250 1260 1270 1280 1290 ]

[ * * * * * ]

'RP1_m1_A' GGCCCACACGGATATGGAGGTGCCTGACTTCACCTATTACCGTCGCTC

'RP1_m1_B' GGCCCACACGGATATGGAGGTGCCTGACTTCACCTATTACCGTCGCTC

'RP1_m5(A)' GGCCCACACGGATATGGAGGTGCCTGACTTCACCTATTACCGTCGCTC

'RP1_m5_B' GGCCCACACGGATATGGAGGTGCCTGACTTCACCTATTACCGTCGCTC

'RP1_m7_A' GGCCCACACGGATATGGAGGTGCCTGACTTCACCTATTACCGTCGCTC

'RP1_m7_B' GGCCCACACGGATATGGAGGTGCCTGACTTCACCTATTACCGTCGCTC

'RP1_m8_A' GGCCCACACGGATATGGAGGTGCCTGACTTCACCTATTACCGTCGCTC

'RP1_m8_B' GGCCCACACGGATATGGAGGTGCCTGACTTCACCTATTACCGTCGCTC

'R_Abg' GGCCCACACGGATATGGAGGTGCCTGACTTCACCTATTACCGTCGCTC

'R_Abg_2' GGCCCACACGGATATGGAGGTGCCTGACTTCACCTATTACCGTCGCTC

'AB_m1_A' GGCCCACACGGATATGGAGGTGCCTGACTTCACCTATTACCGTCGCTC

'AB_m1_B' GGCCCACACGGATATGGAGGTGCCTGACTTCACCTATTACCGTCGCTC

Abh GGCCCACACGGATATGGAGGTGCCTGACTTCACCTAT?ACCGTCGCTC

'Abh_2' GGCCCACACGGATATGGAGGTGCCTGACTTCACCTAT?ACCGTCGCTC

'AB_m2_A' GGCCCACACGGATATGGAGGTGCCTGACTTCACCTATTACCGTCGCTC

'AB_m2_B' GGCCCACACGGATATGGAGGTGCCTGACTTCACCTATTACCGTCGCTC

'IP_m8_A' GGCCCACACGGATATGGAGGTGCCTGACTTCACCTATTACCGTCGCTC

'IP_m8_B' GGCCCACACGGATATGGAGGTGCCTGACTTCACCTATTACCGTCGCTC

'IP_f3_A' GGCCCACACGGATATGGAGGTGCCTGACTTCACCTATTACCGTCGCTC

'IP_f3_B' GGCCCACACGGATATGGAGGTGCCTGACTTCACCTATTACCGTCGCTC

'IP_f23_A' GGCCCACACGGATATGGAGGTGCCTGACTTCACCTATTACCGTCGCTC

'IP_f23_B' GGCCCACACGGATATGGAGGTGCCTGACTTCACCTATTACCGTCGCTC

'IP_m7_A' GGCCCACACGGATATGGAGGTGCCTGACTTCACCTATTACCGTCGCTC

'IP_m7_B' GGCCCACACGGATATGGAGGTGCCTGACTTCACCTATTACCGTCGCTC

'PVL_f14_A' GGCCCACACGGATATGGAGGTGCCTGACTTCACCTATTACCGTCGCTC

'PVL_f14_B' GGCCCACACGGATATGGAGGTGCCTGACTTCACCTATTACCGTCGCTC

'PVL_f12_A' GGCCCACACGGATATGGAGGTGCCTGACTTCACCTATTACCGTCGCTC

'PVL_f12_B' GGCCCACACGGATATGGAGGTGCCTGACTTCACCTATTACCGTCGCTC

'PVL_f3_A' GGCCCACACGGATATGGAGGTGCCTGACTTCACCTATTACCGTCGCTC

'PVL_f3_B' GGCCCACACGGATATGGAGGTGCCTGACTTCACCTATTACCGTCGCTC

'PVL_f7_A' GGCCCACACGGATATGGAGGTGCCTGACTTCACCTATTACCGTCGCTC

'PVL_f7_B' GGCCCACACGGATATGGAGGTGCCTGACTTCACCTATTACCGTCGCTC

'Pes_m9_A' AGCCCACACGGATATGGAGGTGCCTGACTTCACCTATTACCGTCGCTC

'Pes_m9_B' AGCCCACACGGATATGGAGGTGCCTGACTTCACCTATTACCGTCGCTC

'Pes_m10_A' AGCCCACACGGATATGGAGGTGCCTGACTTCACCTATTACCGTCGCTC

'Pes_m10_B' AGCCCACACGGATATGGAGGTGCCTGACTTCACCTATTACCGTCGCTC

'LH_m1_A' GGCCCACACGGATATGGAGGTGCCTGACTTCACCTATTACCGTCGCTC

'LH_m1_B' GGCCCACACGGATATGGAGGTGCCTGACTTCACCTATTACCGTCGCTC

'LH_f6_A' GGCCCACACGGATATGGAGGTGCCTGACTTCACCTATTACCGTCGCTC

'LH_f6_B' GGCCCACACGGATATGGAGGTGCCTGACTTCACCTATTACCGTCGCTC

'LH_f8_A' GGCCCACACGGATATGGAGGTGCCTGACTTCACCTATTACCGTCGCTC

'LH_f8_B' GGCCCACACGGATATGGAGGTGCCTGACTTCACCTATTACCGTCGCTC

'scn_h_a' GGCCCACACGGATATGGAGGTGCCTGACTTCACCTATTACCGTCGCTC

'scn_h_b' GGCCCACACGGATATGGAGGTGCCTGACTTCACCTATTACCGTCGCTC

'scn_m5_A' GGCCCACACGGATATGGAGGTGCCTGACTTCACCTATTACCGTCGCTC

'scn_m5_B' GGCCCACACGGATATGGAGGTGCCTGACTTCACCTATTACCGTCGCTC

'scn_m7_A' GGCCCACACGGATATGGAGGTGCCTGACTTCACCTATTACCGTCGCTC

'scn_m7_B' GGCCCACACGGATATGGAGGTGCCTGACTTCACCTATTACCGTCGCTC

'scn_m3_A' GGCCCACACGGATATGGAGGTGCCTGACTTCACCTATTACCGTCGCTC

'scn_m3_B' GGCCCACACGGATATGGAGGTGCCTGACTTCACCTATTACCGTCGCTC

'LJS_m1_A' GGCCCACACGGATATGGAGGTGCCTGACTTCACCTATTACCGTCGCTC

'LJS_m1_B' GGCCCACACGGATATGGAGGTGCCTGACTTCACCTATTACCGTCGCTC

'LJS_m2_A' GGCCCACACGGATATGGAGGTGCCTGACTTCACCTATTACCGTCGCTC

'LJS_m2_B' GGCCCACACGGATATGGAGGTGCCTGACTTCACCTATTACCGTCGCTC

'LJS_f10_A' GGCCCACACGGATATGGAGGTGCCTGACTTCACCTATTACCGTCGCTC

'LJS_f10_B' GGCCCACACGGATATGGAGGTGCCTGACTTCACCTATTACCGTCGCTC

'LJS_f9_B' GGCCCACACGGATATGGAGGTGCCTGACTTCACCTATTACCGTCGCTC

'LJS_f9_A' GGCCCACACGGATATGGAGGTGCCTGACTTCACCTATTACCGTCGCTC

'LJP_m7_B' GGCCCACACGGATATGGAGGTGCCTGACTTCACCTATTACCGTCGCTC

'LJP_m7_A' GGCCCACACGGATATGGAGGTGCCTGACTTCACCTATTACCGTCGCTC

'LJP_m6_B' GGCCCACACGGATATGGAGGTGCCTGACTTCACCTATTACCGTCGCTC

'LJP_m6_A' GGCCCACACGGATATGGAGGTGCCTGACTTCACCTATTACCGTCGCTC

'LJP_m2_B' GGCCCACACGGATATGGAGGTGCCTGACTTCACCTATTACCGTCGCTC

'LJP_m2_A' GGCCCACACGGATATGGAGGTGCCTGACTTCACCTATTACCGTCGCTC

'LJP_m1_B' GGCCCACACGGATATGGAGGTGCCTGACTTCACCTATTACCGTCGCTC

'LJP_m1_A' GGCCCACACGGATATGGAGGTGCCTGACTTCACCTATTACCGTCGCTC

'BR_m5_A' GGCCCACACGGATATGGAGGTGCCTGACTTCACCTATTACCGTCGCTC

'BR_m5_B' GGCCCACACGGATATGGAGGTGCCTGACTTCACCTATTACCGTCGCTC

'BR_m6_A' GGCCCACACGGATATGGAGGTGCCTGACTTCACCTATTACCGTCGCTC

'BR_m6_B' GGCCCACACGGATATGGAGGTGCCTGACTTCACCTATTACCGTCGCTC

'BR_m7_A' GGCCCACACGGATATGGAGGTGCCTGACTTCACCTATTACCGTCGCTC

'BR_m7_B' GGCCCACACGGATATGGAGGTGCCTGACTTCACCTATTACCGTCGCTC

'BR_f13_B' GGCCCACACGGATATGGAGGTGCCTGACTTCACCTATTACCGTCGCTC

'BR_f13_A' GGCCCACACGGATATGGAGGTGCCTGACTTCACCTATTACCGTCGCTC

'BR_m8_B' GGCCCACACGGATATGGAGGTGCCTGACTTCACCTATTACCGTCGCTC

'BR_m8_A' GGCCCACACGGATATGGAGGTGCCTGACTTCACCTATTACCGTCGCTC

'SD_h_a' GGCCCACACGGATATGGAGGTGCCTGACTTCACCTATTACCGTCGCTC

'SD_h_b' GGCCCACACGGATATGGAGGTGCCTGACTTCACCTATTACCGTCGCTC

'SD_g_a' GGCCCACACGGATATGGAGGTGCCTGACTTCACCTATTACCGTCGCTC

'SD_g_b' GGCCCACACGGATATGGAGGTGCCTGACTTCACCTATTACCGTCGCTC

'SD_2m_b' GGCCCACACGGATATGGAGGTGCCTGACTTCACCTATTACCGTCGCTC

'SD_2m_a' GGCCCACACGGATATGGAGGTGCCTGACTTCACCTATTACCGTCGCTC

[ 1300 1310 1320 1330 1340 ]

[ * * * * * ]

'RP1_m1_A' CTCGACCAAGGACAGTACCGCCAACAATCGCGAGTCCCACGCTAACCG

'RP1_m1_B' CTCGACCAAGGACAGTACCGCCAACAATCGCGAGTCCCACGCTAACCG

'RP1_m5(A)' CTCGACCAAGGACAGTACCGCCAACAATCGCGAGTCCCACGCTAACCG

'RP1_m5_B' CTCGACCAAGGACAGTACCGCCAACAATCGCGAGTCCCACGCTAACCG

'RP1_m7_A' CTCGACCAAGGACAGTACCGCCAACAATCGCGAGTCCCACGCTAACCG

'RP1_m7_B' CTCGACCAAGGACAGTACCGCCAACAATCGCGAGTCCCACGCTAACCG

'RP1_m8_A' CTCGACCAAGGACAGTACCGCCAACAATCGCGAGTCCCACGCTAACCG

'RP1_m8_B' CTCGACCAAGGACAGTACCGCCAACAATCGCGAGTCCCACGCTAACCG

'R_Abg' CTCGACCAAGGACAGTACCGCCAACAATCGCGAGTCCCACGCTAACCG

'R_Abg_2' CTCGACCAAGGACAGTACCGCCAACAATCGCGAGTCCCACGCTAACCG

'AB_m1_A' CTCGACCAAGGACAGTACCGCCAACAATCGCGAGTCCCACGCTAACCG

'AB_m1_B' CTCGACCAAGGACAGTACCGCCAACAATCGCGAGTCCCACGCTAACCG

Abh CTCGACCAAGGACAGTACCGCCAACAATCGCGAGTCCCACGCTAACCG

'Abh_2' CTCGACCAAGGACAGTACCGCCAACAATCGCGAGTCCCACGCTAACCG

'AB_m2_A' CTCGACCAAGGACAGTACCGCCAACAATCGCGAGTCCCACGCTAACCG

'AB_m2_B' CTCGACCAAGGACAGTACCGCCAACAATCGCGAGTCCCACGCTAACCG

'IP_m8_A' CTCGACCAAGGACAGTACCGCCAACAATCGCGAGTCCCACGCTAACCG

'IP_m8_B' CTCGACCAAGGACAGTACCGCCAACAATCGCGAGTCCCACGCTAACCG

'IP_f3_A' CTCGACCAAGGACAGTACCGCCAACAATCGCGAGTCCCACGCTAACCG

'IP_f3_B' CTCGACCAAGGACAGTACCGCCAACAATCGCGAGTCCCACGCTAACCG

'IP_f23_A' CTCGACCAAGGACAGTACCGCCAACAATCGCGAGTCCCACGCTAACCG

'IP_f23_B' CTCGACCAAGGACAGTACCGCCAACAATCGCGAGTCCCACGCTAACCG

'IP_m7_A' CTCGACCAAGGACAGTACCGCCAACAATCGCGAGTCCCACGCTAACCG

'IP_m7_B' CTCGACCAAGGACAGTACCGCCAACAATCGCGAGTCCCACGCTAACCG

'PVL_f14_A' CTCGACCAAGGACAGTACCGCCAACAATCGCGAGTCCCACGCTAACCG

'PVL_f14_B' CTCGACCAAGGACAGTACCGCCAACAATCGCGAGTCCCACGCTAACCG

'PVL_f12_A' CTCGACCAAGGACAGTACCGCCAACAATCGCGAGTCCCACGCTAACCG

'PVL_f12_B' CTCGACCAAGGACAGTACCGCCAACAATCGCGAGTCCCACGCTAACCG

'PVL_f3_A' CTCGACCAAGGACAGTACCGCCAACAATCGCGAGTCCCACGCTAACCG

'PVL_f3_B' CTCGACCAAGGACAGTACCGCCAACAATCGCGAGTCCCACGCTAACCG

'PVL_f7_A' CTCGACCAAGGACAGTACCGCCAACAATCGCGAGTCCCACGCTAACCG

'PVL_f7_B' CTCGACCAAGGACAGTACCGCCAACAATCGCGAGTCCCACGCTAACCG

'Pes_m9_A' CTCGACCAAGGACAGTACCGCCAAGAATCGCGAGTCCCACGCCAACCG

'Pes_m9_B' CTCGACCAAGGACAGTACCGCCAAGAATCGCGAGTCCCACGCCAACCG

'Pes_m10_A' CTCGACCAAGGACAGTACCGCCAAGAATCGCGAGTCCCACGCCAACCG

'Pes_m10_B' CTCGACCAAGGACAGTACCGCCAAGAATCGCGAGTCCCACGCCAACCG

'LH_m1_A' CTCGACCAAGGACAGTACCGCCAAGAATCGCGAGTCCCACGCCAACCG

'LH_m1_B' CTCGACCAAGGACAGTACCGCCAAGAATCGCGAGTCCCACGCCAACCG

'LH_f6_A' CTCGACCAAGGACAGTACCGCCAAGAATCGCGAGTCCCACGCCAACCG

'LH_f6_B' CTCGACCAAGGACAGTACCGCCAAGAATCGCGAGTCCCACGCCAACCG

'LH_f8_A' CTCGACCAAGGACAGTACCGCCAAGAATCGCGAGTCCCACGCCAACCG

'LH_f8_B' CTCGACCAAGGACAGTACCGCCAAGAATCGCGAGTCCCACGCCAACCG

'scn_h_a' CTCGACCAAGGACAGTACCGCCAAGAATCGCGAGTCCCACGCCAACCG

'scn_h_b' CTCGACCAAGGACAGTACCGCCAAGAATCGCGAGTCCCACGCCAACCG

'scn_m5_A' CTCGACCAAGGACAGTACCGCCAAGAATCGCGAGTCCCACGCCAACCG

'scn_m5_B' CTCGACCAAGGACAGTACCGCCAAGAATCGCGAGTCCCACGCCAACCG

'scn_m7_A' CTCGACCAAGGACAGTACCGCCAAGAATCGCGAGTCCCACGCCAACCG

'scn_m7_B' CTCGACCAAGGACAGTACCGCCAAGAATCGCGAGTCCCACGCCAACCG

'scn_m3_A' CTCGACCAAGGACAGTACCGCCAAGAATCGCGAGTCCCACGCCAACCG

'scn_m3_B' CTCGACCAAGGACAGTACCGCCAAGAATCGCGAGTCCCACGCCAACCG

'LJS_m1_A' CTCGACCAAGGACAGTACCGCCAAGAATCGCGAGTCCCACGCCAACCG

'LJS_m1_B' CTCGACCAAGGACAGTACCGCCAAGAATCGCGAGTCCCACGCCAACCG

'LJS_m2_A' CTCGACCAAGGACAGTACCGCCAAGAATCGCGAGTCCCACGCCAACCG

'LJS_m2_B' CTCGACCAAGGACAGTACCGCCAAGAATCGCGAGTCCCACGCCAACCG

'LJS_f10_A' CTCGACCAAGGACAGTACCGCCAAGAATCGCGAGTCCCACGCCAACCG

'LJS_f10_B' CTCGACCAAGGACAGTACCGCCAAGAATCGCGAGTCCCACGCCAACCG

'LJS_f9_B' CTCGACCAAGGACAGTACCGCCAAGAATCGCGAGTCCCACGCCAACCG

'LJS_f9_A' CTCGACCAAGGACAGTACCGCCAAGAATCGCGAGTCCCACGCCAACCG

'LJP_m7_B' CTCGACCAAGGACAGTACCGCCAAGAATCGCGAGTCCCACGCCAACCG

'LJP_m7_A' CTCGACCAAGGACAGTACCGCCAAGAATCGCGAGTCCCACGCCAACCG

'LJP_m6_B' CTCGACCAAGGACAGTACCGCCAAGAATCGCGAGTCCCACGCCAACCG

'LJP_m6_A' CTCGACCAAGGACAGTACCGCCAAGAATCGCGAGTCCCACGCCAACCG

'LJP_m2_B' CTCGACCAAGGACAGTACCGCCAAGAATCGCGAGTCCCACGCCAACCG

'LJP_m2_A' CTCGACCAAGGACAGTACCGCCAAGAATCGCGAGTCCCACGCCAACCG

'LJP_m1_B' CTCGACCAAGGACAGTACCGCCAAGAATCGCGAGTCCCACGCCAACCG

'LJP_m1_A' CTCGACCAAGGACAGTACCGCCAAGAATCGCGAGTCCCACGCCAACCG

'BR_m5_A' CTCGACCAAGGACAGTACCGCCAAGAATCGCGAGTCCCACGCCAACCG

'BR_m5_B' CTCGACCAAGGACAGTACCGCCAAGAATCGCGAGTCCCACGCCAACCG

'BR_m6_A' CTCGACCAAGGACAGTACCGCCAAGAATCGCGAGTCCCACGCCAACCG

'BR_m6_B' CTCGACCAAGGACAGTACCGCCAAGAATCGCGAGTCCCACGCCAACCG

'BR_m7_A' CTCGACCAAGGACAGTACCGCCAAGAATCGCGAGTCCCACGCCAACCG

'BR_m7_B' CTCGACCAAGGACAGTACCGCCAAGAATCGCGAGTCCCACGCCAACCG

'BR_f13_B' CTCGACCAAGGACAGTACCGCCAAGAATCGCGAGTCCCACGCCAACCG

'BR_f13_A' CTCGACCAAGGACAGTACCGCCAAGAATCGCGAGTCCCACGCCAACCG

'BR_m8_B' CTCGACCAAGGACAGTACCGCCAAGAATCGCGAGTCCCACGCCAACCG

'BR_m8_A' CTCGACCAAGGACAGTACCGCCAAGAATCGCGAGTCCCACGCCAACCG

'SD_h_a' CTCGACCAAGGACAGTACCGCCAAGAATCGCGAGTCCCACGCCAACCG

'SD_h_b' CTCGACCAAGGACAGTACCGCCAAGAATCGCGAGTCCCACGCCAACCG

'SD_g_a' CTCGACCAAGGACAGTACCGCCAAGAATCGCGAGTCCCACGCCAACCG

'SD_g_b' CTCGACCAAGGACAGTACCGCCAAGAATCGCGAGTCCCACGCCAACCG

'SD_2m_b' CTCGACCAAGGACAGTACCGCCAAGAATCGCGAGTCCCACGCCAACCG

'SD_2m_a' CTCGACCAAGGACAGTACCGCCAAGAATCGCGAGTCCCACGCCAACCG

[ 1350 1360 1370 1380 1390 ]

[ * * * * * ]

'RP1_m1_A' CAATGGTTTCGCTTACTTGATGACGGCCGGAGCGGCCATTCCCTCCGT

'RP1_m1_B' CAATGGTTTCGCTTACTTGATGACGGCCGGAGCGGCCATTCCCTCCGT

'RP1_m5(A)' CAATGGTTTCGCTTACTTGATGACGGCCGGAGCGGCCATTCCCTCCGT

'RP1_m5_B' CAATGGTTTCGCTTACTTGATGACGGCCGGAGCGGCCATTCCCTCCGT

'RP1_m7_A' CAATGGTTTCGCTTACTTGATGACGGCCGGAGCGGCCATTCCCTCCGT

'RP1_m7_B' CAATGGTTTCGCTTACTTGATGACGGCCGGAGCGGCCATTCCCTCCGT

'RP1_m8_A' CAATGGTTTCGCTTACTTGATGACGGCCGGAGCGGCCATTCCCTCCGT

'RP1_m8_B' CAATGGTTTCGCTTACTTGATGACGGCCGGAGCGGCCATTCCCTCCGT

'R_Abg' CAATGGTTTCGCTTACTTGATGACGGCCGGAGCGGCCATTCCCTCCGT

'R_Abg_2' CAATGGTTTCGCTTACTTGATGACGGCCGGAGCGGCCATTCCCTCCGT

'AB_m1_A' CAATGGTTTCGCTTACTTGATGACGGCCGGAGCGGCCATTCCCTCCGT

'AB_m1_B' CAATGGTTTCGCTTACTTGATGACGGCCGGAGCGGCCATTCCCTCCGT

Abh CAATGGTTTCGCTTACTTGATGACGGCCGGAGCGGCCATTCCCTCCGT

'Abh_2' CAATGGTTTCGCTTACTTGATGACGGCCGGAGCGGCCATTCCCTCCGT

'AB_m2_A' CAATGGTTTCGCTTACTTGATGACGGCCGGAGCGGCCATTCCCTCCGT

'AB_m2_B' CAATGGTTTCGCTTACTTGATGACGGCCGGAGCGGCCATTCCCTCCGT

'IP_m8_A' CAATGGTTTCGCTTACTTGATGACGGCCGGAGCGGCCATTCCCTCCGT

'IP_m8_B' CAATGGTTTCGCTTACTTGATGACGGCCGGAGCGGCCATTCCCTCCGT

'IP_f3_A' CAATGGTTTCGCTTACTTGATGACGGCCGGAGCGGCCATTCCCTCCGT

'IP_f3_B' CAATGGTTTCGCTTACTTGATGACGGCCGGAGCGGCCATTCCCTCCGT

'IP_f23_A' CAATGGTTTCGCTTACTTGATGACGGCCGGAGCGGCCATTCCCTCCGT

'IP_f23_B' CAATGGTTTCGCTTACTTGATGACGGCCGGAGCGGCCATTCCCTCCGT

'IP_m7_A' CAATGGTTTCGCTTACTTGATGACGGCCGGAGCGGCCATTCCCTCCGT

'IP_m7_B' CAATGGTTTCGCTTACTTGATGACGGCCGGAGCGGCCATTCCCTCCGT

'PVL_f14_A' CAATGGTTTCGCTTACTTGATGACGGCCGGAGCGGCCATTCCCTCCGT

'PVL_f14_B' CAATGGTTTCGCTTACTTGATGACGGCCGGAGCGGCCATTCCCTCCGT

'PVL_f12_A' CAATGGTTTCGCTTACTTGATGACGGCCGGAGCGGCCATTCCCTCCGT

'PVL_f12_B' CAATGGTTTCGCTTACTTGATGACGGCCGGAGCGGCCATTCCCTCCGT

'PVL_f3_A' CAATGGTTTCGCTTACTTGATGACGGCCGGAGCGGCCATTCCCTCCGT

'PVL_f3_B' CAATGGTTTCGCTTACTTGATGACGGCCGGAGCGGCCATTCCCTCCGT

'PVL_f7_A' CAATGGTTTCGCTTACTTGATGACGGCCGGAGCGGCCATTCCCTCCGT

'PVL_f7_B' CAATGGTTTCGCTTACTTGATGACGGCCGGAGCGGCCATTCCCTCCGT

'Pes_m9_A' CAATGGTTTCGCTTACTTGATGACGGCCGGAGCGGCCATTCCCTCCGT

'Pes_m9_B' CAATGGTTTCGCTTACTTGATGACGGCCGGAGCGGCCATTCCCTCCGT

'Pes_m10_A' CAATGGTTTCGCTTACTTGATGACGGCCGGAGCGGCCATTCCCTCCGT

'Pes_m10_B' CAATGGTTTCGCTTACTTGATGACGGCCGGAGCGGCCATTCCCTCCGT

'LH_m1_A' CAATGGTTTCGCTTACTTGATGACGGCCGGAGCGGCCATTCCCTCCGT

'LH_m1_B' CAATGGTTTCGCTTACTTGATGACGGCCGGAGCGGCCATTCCCTCCGT

'LH_f6_A' CAATGGTTTCGCTTACTTGATGACGGCCGGAGCGGCCATTCCCTCCGT

'LH_f6_B' CAATGGTTTCGCTTACTTGATGACGGCCGGAGCGGCCATTCCCTCCGT

'LH_f8_A' CAATGGTTTCGCTTACTTGATGACGGCCGGAGCGGCCATTCCCTCCGT

'LH_f8_B' CAATGGTTTCGCTTACTTGATGACGGCCGGAGCGGCCATTCCCTCCGT

'scn_h_a' CAATGGTTTCGCTTACTTGATGACGGCCGGAGCGGCCATTCCCTCCGT

'scn_h_b' CAATGGTTTCGCTTACTTGATGACGGCCGGAGCGGCCATTCCCTCCGT

'scn_m5_A' CAATGGTTTCGCTTACTTGATGACGGCCGGAGCGGCCATTCCCTCCGT

'scn_m5_B' CAATGGTTTCGCTTACTTGATGACGGCCGGAGCGGCCATTCCCTCCGT

'scn_m7_A' CAATGGTTTCGCTTACTTGATGACGGCCGGAGCGGCCATTCCCTCCGT

'scn_m7_B' CAATGGTTTCGCTTACTTGATGACGGCCGGAGCGGCCATTCCCTCCGT

'scn_m3_A' CAATGGTTTCGCTTACTTGATGACGGCCGGAGCGGCCATTCCCTCCGT

'scn_m3_B' CAATGGTTTCGCTTACTTGATGACGGCCGGAGCGGCCATTCCCTCCGT

'LJS_m1_A' CAATGGTTTCGCTTACTTGATGACGGCCGGAGCGGCCATTCCCTCCGT

'LJS_m1_B' CAATGGTTTCGCTTACTTGATGACGGCCGGAGCGGCCATTCCCTCCGT

'LJS_m2_A' CAATGGTTTCGCTTACTTGATGACGGCCGGAGCGGCCATTCCCTCCGT

'LJS_m2_B' CAATGGTTTCGCTTACTTGATGACGGCCGGAGCGGCCATTCCCTCCGT

'LJS_f10_A' CAATGGTTTCGCTTACTTGATGACGGCCGGAGCGGCCATTCCCTCCGT

'LJS_f10_B' CAATGGTTTCGCTTACTTGATGACGGCCGGAGCGGCCATTCCCTCCGT

'LJS_f9_B' CAATGGTTTCGCTTACTTGATGACGGCCGGAGCGGCCATTCCCTCCGT

'LJS_f9_A' CAATGGTTTCGCTTACTTGATGACGGCCGGAGCGGCCATTCCCTCCGT

'LJP_m7_B' CAATGGTTTCGCTTACTTGATGACGGCCGGAGCGGCCATTCCCTCCGT

'LJP_m7_A' CAATGGTTTCGCTTACTTGATGACGGCCGGAGCGGCCATTCCCTCCGT

'LJP_m6_B' CAATGGTTTCGCTTACTTGATGACGGCCGGAGCGGCCATTCCCTCCGT

'LJP_m6_A' CAATGGTTTCGCTTACTTGATGACGGCCGGAGCGGCCATTCCCTCCGT

'LJP_m2_B' CAATGGTTTCGCTTACTTGATGACGGCCGGAGCGGCCATTCCCTCCGT

'LJP_m2_A' CAATGGTTTCGCTTACTTGATGACGGCCGGAGCGGCCATTCCCTCCGT

'LJP_m1_B' CAATGGTTTCGCTTACTTGATGACGGCCGGAGCGGCCATTCCCTCCGT

'LJP_m1_A' CAATGGTTTCGCTTACTTGATGACGGCCGGAGCGGCCATTCCCTCCGT

'BR_m5_A' CAATGGTTTCGCTTACTTGATGACGGCCGGAGCGGCCATTCCCTCCGT

'BR_m5_B' CAATGGTTTCGCTTACTTGATGACGGCCGGAGCGGCCATTCCCTCCGT

'BR_m6_A' CAATGGTTTCGCTTACTTGATGACGGCCGGAGCGGCCATTCCCTCCGT

'BR_m6_B' CAATGGTTTCGCTTACTTGATGACGGCCGGAGCGGCCATTCCCTCCGT

'BR_m7_A' CAATGGTTTCGCTTACTTGATGACGGCCGGAGCGGCCATTCCCTCCGT

'BR_m7_B' CAATGGTTTCGCTTACTTGATGACGGCCGGAGCGGCCATTCCCTCCGT

'BR_f13_B' CAATGGTTTCGCTTACTTGATGACGGCCGGAGCGGCCATTCCCTCCGT

'BR_f13_A' CAATGGTTTCGCTTACTTGATGACGGCCGGAGCGGCCATTCCCTCCGT

'BR_m8_B' CAATGGTTTCGCTTACTTGATGACGGCCGGAGCGGCCATTCCCTCCGT

'BR_m8_A' CAATGGTTTCGCTTACTTGATGACGGCCGGAGCGGCCATTCCCTCCGT

'SD_h_a' CAATGGTTTCGCTTACTTGATGACGGCCGGAGCGGCCATTCCCTCCGT

'SD_h_b' CAATGGTTTCGCTTACTTGATGACGGCCGGAGCGGCCATTCCCTCCGT

'SD_g_a' CAATGGTTTCGCTTACTTGATGACGGCCGGAGCGGCCATTCCCTCCGT

'SD_g_b' CAATGGTTTCGCTTACTTGATGACGGCCGGAGCGGCCATTCCCTCCGT

'SD_2m_b' CAATGGTTTCGCTTACTTGATGACGGCCGGAGCGGCCATTCCCTCCGT

'SD_2m_a' CAATGGTTTCGCTTACTTGATGACGGCCGGAGCGGCCATTCCCTCCGT

[ 1400 1410 1420 1430 1440]

[ * * * * *]

'RP1_m1_A' GTACGCTGCTACCAAGTTCGTCAACGTGTTCATTTCGAACTTGTCGCC

'RP1_m1_B' GTACGCTGCTACCAAGTTCGTCAACGTGTTCATTTCGAACTTGTCGCC

'RP1_m5(A)' GTACGCTGCTACCAAGTTCGTCAACGTGTTCATTTCGAACTTGTCGCC

'RP1_m5_B' GTACGCTGCTACCAAGTTCGTCAACGTGTTCATTTCGAACTTGTCGCC

'RP1_m7_A' GTACGCTGCTACCAAGTTCGTCAACGTGTTCATTTCGAACTTGTCGCC

'RP1_m7_B' GTACGCTGCTACCAAGTTCGTCAACGTGTTCATTTCGAACTTGTCGCC

'RP1_m8_A' GTACGCTGCTACCAAGTTCGTCAACGTGTTCATTTCGAACTTGTCGCC

'RP1_m8_B' GTACGCTGCTACCAAGTTCGTCAACGTGTTCATTTCGAACTTGTCGCC

'R_Abg' GTACGCTGCTACCAAGTTCGTCAACGTGTTCATTTCGAACTTGTCGCC

'R_Abg_2' GTACGCTGCTACCAAGTTCGTCAACGTGTTCATTTCGAACTTGTCGCC

'AB_m1_A' GTACGCTGCTACCAAGTTCGTCAACGTGTTCATTTCGAACTTGTCGCC

'AB_m1_B' GTACGCTGCTACCAAGTTCGTCAACGTGTTCATTTCGAACTTGTCGCC

Abh GTACGCTGCTACCAAGTTCGTCAACGTGTTCATTTCGAACTTGTCGCC

'Abh_2' GTACGCTGCTACCAAGTTCGTCAACGTGTTCATTTCGAACTTGTCGCC

'AB_m2_A' GTACGCTGCTACCAAGTTCGTCAACGTGTTCATTTCGAACTTGTCGCC

'AB_m2_B' GTACGCTGCTACCAAGTTCGTCAACGTGTTCATTTCGAACTTGTCGCC

'IP_m8_A' GTACGCTGCTACCAAGTTCGTCAACGTGTTCATTTCGAACTTGTCGCC

'IP_m8_B' GTACGCTGCTACCAAGTTCGTCAACGTGTTCATTTCGAACTTGTCGCC

'IP_f3_A' GTACGCTGCTACCAAGTTCGTCAACGTGTTCATTTCGAACTTGTCGCC

'IP_f3_B' GTACGCTGCTACCAAGTTCGTCAACGTGTTCATTTCGAACTTGTCGCC

'IP_f23_A' GTACGCTGCTACCAAGTTCGTCAACGTGTTCATTTCGAACTTGTCGCC

'IP_f23_B' GTACGCTGCTACCAAGTTCGTCAACGTGTTCATTTCGAACTTGTCGCC

'IP_m7_A' GTACGCTGCTACCAAGTTCGTCAACGTGTTCATTTCGAACTTGTCGCC

'IP_m7_B' GTACGCTGCTACCAAGTTCGTCAACGTGTTCATTTCGAACTTGTCGCC

'PVL_f14_A' GTACGCTGCTACCAAGTTCGTCAACGTGTTCATTTCGAACTTGTCGCC

'PVL_f14_B' GTACGCTGCTACCAAGTTCGTCAACGTGTTCATTTCGAACTTGTCGCC

'PVL_f12_A' GTACGCTGCTACCAAGTTCGTCAACGTGTTCATTTCGAACTTGTCGCC

'PVL_f12_B' GTACGCTGCTACCAAGTTCGTCAACGTGTTCATTTCGAACTTGTCGCC

'PVL_f3_A' GTACGCTGCTACCAAGTTCGTCAACGTGTTCATTTCGAACTTGTCGCC

'PVL_f3_B' GTACGCTGCTACCAAGTTCGTCAACGTGTTCATTTCGAACTTGTCGCC

'PVL_f7_A' GTACGCTGCTACCAAGTTCGTCAACGTGTTCATTTCGAACTTGTCGCC

'PVL_f7_B' GTACGCTGCTACCAAGTTCGTCAACGTGTTCATTTCGAACTTGTCGCC

'Pes_m9_A' GTACGCTGCCACCAAGTTCGTCAACGTGTTCATTTCGAACTTGTCGCC

'Pes_m9_B' GTACGCTGCCACCAAGTTCGTCAACGTGTTCATTTCGAACTTGTCGCC

'Pes_m10_A' GTACGCTGCCACCAAGTTCGTCAACGTGTTCATTTCGAACTTGTCGCC

'Pes_m10_B' GTACGCTGCCACCAAGTTCGTCAACGTGTTCATTTCGAACTTGTCGCC

'LH_m1_A' GTACGCTGCCACCAAGTTCGTCAACGTGTTCATTTCGAACTTGTCGCC

'LH_m1_B' GTACGCTGCCACCAAGTTCGTCAACGTGTTCATTTCGAACTTGTCGCC

'LH_f6_A' GTACGCTGCCACCAAGTTCGTCAACGTGTTCATTTCGAACTTGTCGCC

'LH_f6_B' GTACGCTGCCACCAAGTTCGTCAACGTGTTCATTTCGAACTTGTCGCC

'LH_f8_A' GTACGCTGCCACCAAGTTCGTCAACGTGTTCATTTCGAACTTGTCGCC

'LH_f8_B' GTACGCTGCCACCAAGTTCGTCAACGTGTTCATTTCGAACTTGTCGCC

'scn_h_a' GTACGCTGCCACCAAGTTCGTCAACGTGTTCATTTCGAACTTGTCGCC

'scn_h_b' GTACGCTGCCACCAAGTTCGTCAACGTGTTCATTTCGAACTTGTCGCC

'scn_m5_A' GTACGCTGCCACCAAGTTCGTCAACGTGTTCATTTCGAACTTGTCGCC

'scn_m5_B' GTACGCTGCCACCAAGTTCGTCAACGTGTTCATTTCGAACTTGTCGCC

'scn_m7_A' GTACGCTGCCACCAAGTTCGTCAACGTGTTCATTTCGAACTTGTCGCC

'scn_m7_B' GTACGCTGCCACCAAGTTCGTCAACGTGTTCATTTCGAACTTGTCGCC

'scn_m3_A' GTACGCTGCCACCAAGTTCGTCAACGTGTTCATTTCGAACTTGTCGCC

'scn_m3_B' GTACGCTGCCACCAAGTTCGTCAACGTGTTCATTTCGAACTTGTCGCC

'LJS_m1_A' GTACGGTGCTACCAAGTTCGTCAACGTGTTCATTTCGAACTTGTCGCC

'LJS_m1_B' GTACGGTGCTACCAAGTTCGTCAACGTGTTCATTTCGAACTTGTCGCC

'LJS_m2_A' GTACGGTGCTACCAAGTTCGTCAACGTGTTCATTTCGAACTTGTCGCC

'LJS_m2_B' GTACGGTGCTACCAAGTTCGTCAACGTGTTCATTTCGAACTTGTCGCC

'LJS_f10_A' GTACGGTGCTACCAAGTTCGTCAACGTGTTCATTTCGAACTTGTCGCC

'LJS_f10_B' GTACGGTGCTACCAAGTTCGTCAACGTGTTCATTTCGAACTTGTCGCC

'LJS_f9_B' GTACGGTGCTACCAAGTTCGTCAACGTGTTCATTTCGAACTTGTCGCC

'LJS_f9_A' GTACGGTGCTACCAAGTTCGTCAACGTGTTCATTTCGAACTTGTCGCC

'LJP_m7_B' GTACGGTGCTACCAAGTTCGTCAACGTGTTCATTTCGAACTTGTCGCC

'LJP_m7_A' GTACGGTGCTACCAAGTTCGTCAACGTGTTCATTTCGAACTTGTCGCC

'LJP_m6_B' GTACGGTGCTACCAAGTTCGTCAACGTGTTCATTTCGAACTTGTCGCC

'LJP_m6_A' GTACGGTGCTACCAAGTTCGTCAACGTGTTCATTTCGAACTTGTCGCC

'LJP_m2_B' GTACGGTGCTACCAAGTTCGTCAACGTGTTCATTTCGAACTTGTCGCC

'LJP_m2_A' GTACGGTGCTACCAAGTTCGTCAACGTGTTCATTTCGAACTTGTCGCC

'LJP_m1_B' GTACGGTGCTACCAAGTTCGTCAACGTGTTCATTTCGAACTTGTCGCC

'LJP_m1_A' GTACGGTGCTACCAAGTTCGTCAACGTGTTCATTTCGAACTTGTCGCC

'BR_m5_A' GTACGGTGCTACCAAGTTCGTCAACGTGTTCATTTCGAACTTGTCGCC

'BR_m5_B' GTACGGTGCTACCAAGTTCGTCAACGTGTTCATTTCGAACTTGTCGCC

'BR_m6_A' GTACGGTGCTACCAAGTTCGTCAACGTGTTCATTTCGAACTTGTCGCC

'BR_m6_B' GTACGGTGCTACCAAGTTCGTCAACGTGTTCATTTCGAACTTGTCGCC

'BR_m7_A' GTACGGTGCTACCAAGTTCGTCAACGTGTTCATTTCGAACTTGTCGCC

'BR_m7_B' GTACGGTGCTACCAAGTTCGTCAACGTGTTCATTTCGAACTTGTCGCC

'BR_f13_B' GTACGGTGCTACCAAGTTCGTCAACGTGTTCATTTCGAACTTGTCGCC

'BR_f13_A' GTACGGTGCTACCAAGTTCGTCAACGTGTTCATTTCGAACTTGTCGCC

'BR_m8_B' GTACGGTGCTACCAAGTTCGTCAACGTGTTCATTTCGAACTTGTCGCC

'BR_m8_A' GTACGGTGCTACCAAGTTCGTCAACGTGTTCATTTCGAACTTGTCGCC

'SD_h_a' GTACGGTGCTACCAAGTTCGTCAACGTGTTCATTTCGAACTTGTCGCC

'SD_h_b' GTACGGTGCTACCAAGTTCGTCAACGTGTTCATTTCGAACTTGTCGCC

'SD_g_a' GTACGGTGCTACCAAGTTCGTCAACGTGTTCATTTCGAACTTGTCGCC

'SD_g_b' GTACGGTGCTACCAAGTTCGTCAACGTGTTCATTTCGAACTTGTCGCC

'SD_2m_b' GTACGGTGCTACCAAGTTCGTCAACGTGTTCATTTCGAACTTGTCGCC

'SD_2m_a' GTACGGTGCTACCAAGTTCGTCAACGTGTTCATTTCGAACTTGTCGCC

[ 1450 1460 1470 1480 ]

[ * * * * ]

'RP1_m1_A' CTCGCGCGATGTCTTGGCCATGGCCAAGATTGAAGTCAAACTGTCCGA

'RP1_m1_B' CTCGCGCGATGTCTTGGCCATGGCCAAGATTGAAGTCAAACTGTCCGA

'RP1_m5(A)' CTCGCGCGATGTCTTGGCCATGGCCAAGATTGAAGTCAAACTGTCCGA

'RP1_m5_B' CTCGCGCGATGTCTTGGCCATGGCCAAGATTGAAGTCAAACTGTCCGA

'RP1_m7_A' CTCGCGCGATGTCTTGGCCATGGCCAAGATTGAAGTCAAACTGTCCGA

'RP1_m7_B' CTCGCGCGATGTCTTGGCCATGGCCAAGATTGAAGTCAAACTGTCCGA

'RP1_m8_A' CTCGCGCGATGTCTTGGCCATGGCCAAGATTGAAGTCAAACTGTCCGA

'RP1_m8_B' CTCGCGCGATGTCTTGGCCATGGCCAAGATTGAAGTCAAACTGTCCGA

'R_Abg' CTCGCGCGATGTCTTGGCCATGGCCAAGATTGAAGTCAAACTGTCCGA

'R_Abg_2' CTCGCGCGATGTCTTGGCCATGGCCAAGATTGAAGTCAAACTGTCCGA

'AB_m1_A' CTCGCGCGATGTCTTGGCCATGGCCAAGATTGAAGTCAAACTGTCCGA

'AB_m1_B' CTCGCGCGATGTCTTGGCCATGGCCAAGATTGAAGTCAAACTGTCCGA

Abh CTCGCGCGATGTCTTGGCCATGGCCAAGATTGAAGTCAAACTGTCCGA

'Abh_2' CTCGCGCGATGTCTTGGCCATGGCCAAGATTGAAGTCAAACTGTCCGA

'AB_m2_A' CTCGCGCGATGTCTTGGCCATGGCCAAGATTGAAGTCAAACTGTCCGA

'AB_m2_B' CTCGCGCGATGTCTTGGCCATGGCCAAGATTGAAGTCAAACTGTCCGA

'IP_m8_A' CTCGCGCGATGTCTTGGCCATGGCCAAGATTGAAGTCAAACTGTCCGA

'IP_m8_B' CTCGCGCGATGTCTTGGCCATGGCCAAGATTGAAGTCAAACTGTCCGA

'IP_f3_A' CTCGCGCGATGTCTTGGCCATGGCCAAGATTGAAGTCAAACTGTCCGA

'IP_f3_B' CTCGCGCGATGTCTTGGCCATGGCCAAGATTGAAGTCAAACTGTCCGA

'IP_f23_A' CTCGCGCGATGTCTTGGCCATGGCCAAGATTGAAGTCAAACTGTCCGA

'IP_f23_B' CTCGCGCGATGTCTTGGCCATGGCCAAGATTGAAGTCAAACTGTCCGA

'IP_m7_A' CTCGCGCGATGTCTTGGCCATGGCCAAGATTGAAGTCAAACTGTCCGA

'IP_m7_B' CTCGCGCGATGTCTTGGCCATGGCCAAGATTGAAGTCAAACTGTCCGA

'PVL_f14_A' CTCGCGCGATGTCTTGGCCATGGCCAAGATTGAAGTCAAACTGTCCGA

'PVL_f14_B' CTCGCGCGATGTCTTGGCCATGGCCAAGATTGAAGTCAAACTGTCCGA

'PVL_f12_A' CTCGCGCGATGTCTTGGCCATGGCCAAGATTGAAGTCAAACTGTCCGA

'PVL_f12_B' CTCGCGCGATGTCTTGGCCATGGCCAAGATTGAAGTCAAACTGTCCGA

'PVL_f3_A' CTCGCGCGATGTCTTGGCCATGGCCAAGATTGAAGTCAAACTGTCCGA

'PVL_f3_B' CTCGCGCGATGTCTTGGCCATGGCCAAGATTGAAGTCAAACTGTCCGA

'PVL_f7_A' CTCGCGCGATGTCTTGGCCATGGCCAAGATTGAAGTCAAACTGTCCGA

'PVL_f7_B' CTCGCGCGATGTCTTGGCCATGGCCAAGATTGAAGTCAAACTGTCCGA

'Pes_m9_A' CTCGCGCGATGTCTTGGCCATGGCCAAGATTGAAGTCAAACTGTCCGA

'Pes_m9_B' CTCGCGCGATGTCTTGGCCATGGCCAAGATTGAAGTCAAACTGTCCGA

'Pes_m10_A' CTCGCGCGATGTCTTGGCCATGGCCAAGATTGAAGTCAAACTGTCCGA

'Pes_m10_B' CTCGCGCGATGTCTTGGCCATGGCCAAGATTGAAGTCAAACTGTCCGA

'LH_m1_A' CTCGCGCGATGTCTTGGCCATGGCCAAGATTGAAGTCAAACTGTCCGA

'LH_m1_B' CTCGCGCGATGTCTTGGCCATGGCCAAGATTGAAGTCAAACTGTCCGA

'LH_f6_A' CTCGCGCGATGTCTTGGCCATGGCCAAGATTGAAGTCAAACTGTCCGA

'LH_f6_B' CTCGCGCGATGTCTTGGCCATGGCCAAGATTGAAGTCAAACTGTCCGA

'LH_f8_A' CTCGCGCGATGTCTTGGCCATGGCCAAGATTGAAGTCAAACTGTCCGA

'LH_f8_B' CTCGCGCGATGTCTTGGCCATGGCCAAGATTGAAGTCAAACTGTCCGA

'scn_h_a' CTCGCGCGATGTCTTGGCCATGGCCAAGATTGAAGTCAAACTGTCCGA

'scn_h_b' CTCGCGCGATGTCTTGGCCATGGCCAAGATTGAAGTCAAACTGTCCGA

'scn_m5_A' CTCGCGCGATGTCTTGGCCATGGCCAAGATTGAAGTCAAACTGTCCGA

'scn_m5_B' CTCGCGCGATGTCTTGGCCATGGCCAAGATTGAAGTCAAACTGTCCGA

'scn_m7_A' CTCGCGCGATGTCTTGGCCATGGCCAAGATTGAAGTCAAACTGTCCGA

'scn_m7_B' CTCGCGCGATGTCTTGGCCATGGCCAAGATTGAAGTCAAACTGTCCGA

'scn_m3_A' CTCGCGCGATGTCTTGGCCATGGCCAAGATTGAAGTCAAACTGTCCGA

'scn_m3_B' CTCGCGCGATGTCTTGGCCATGGCCAAGATTGAAGTCAAACTGTCCGA

'LJS_m1_A' CTCGCGCGATGTCTTGGCCATGGCCAAGATTGAAGTCAAACTGTCCGA

'LJS_m1_B' CTCGCGCGATGTCTTGGCCATGGCCAAGATTGAAGTCAAACTGTCCGA

'LJS_m2_A' CTCGCGCGATGTCTTGGCCATGGCCAAGATTGAAGTCAAACTGTCCGA

'LJS_m2_B' CTCGCGCGATGTCTTGGCCATGGCCAAGATTGAAGTCAAACTGTCCGA

'LJS_f10_A' CTCGCGCGATGTCTTGGCCATGGCCAAGATTGAAGTCAAACTGTCCGA

'LJS_f10_B' CTCGCGCGATGTCTTGGCCATGGCCAAGATTGAAGTCAAACTGTCCGA

'LJS_f9_B' CTCGCGCGATGTCTTGGCCATGGCCAAGATTGAAGTCAAACTGTCCGA

'LJS_f9_A' CTCGCGCGATGTCTTGGCCATGGCCAAGATTGAAGTCAAACTGTCCGA

'LJP_m7_B' CTCGCGCGATGTCTTGGCCATGGCCAAGATTGAAGTCAAACTGTCCGA

'LJP_m7_A' CTCGCGCGATGTCTTGGCCATGGCCAAGATTGAAGTCAAACTGTCCGA

'LJP_m6_B' CTCGCGCGATGTCTTGGCCATGGCCAAGATTGAAGTCAAACTGTCCGA

'LJP_m6_A' CTCGCGCGATGTCTTGGCCATGGCCAAGATTGAAGTCAAACTGTCCGA

'LJP_m2_B' CTCGCGCGATGTCTTGGCCATGGCCAAGATTGAAGTCAAACTGTCCGA

'LJP_m2_A' CTCGCGCGATGTCTTGGCCATGGCCAAGATTGAAGTCAAACTGTCCGA

'LJP_m1_B' CTCGCGCGATGTCTTGGCCATGGCCAAGATTGAAGTCAAACTGTCCGA

'LJP_m1_A' CTCGCGCGATGTCTTGGCCATGGCCAAGATTGAAGTCAAACTGTCCGA

'BR_m5_A' CTCGCGCGATGTCTTGGCCATGGCCAAGATTGAAGTCAAACTGTCCGA

'BR_m5_B' CTCGCGCGATGTCTTGGCCATGGCCAAGATTGAAGTCAAACTGTCCGA

'BR_m6_A' CTCGCGCGATGTCTTGGCCATGGCCAAGATTGAAGTCAAACTGTCCGA

'BR_m6_B' CTCGCGCGATGTCTTGGCCATGGCCAAGATTGAAGTCAAACTGTCCGA

'BR_m7_A' CTCGCGCGATGTCTTGGCCATGGCCAAGATTGAAGTCAAACTGTCCGA

'BR_m7_B' CTCGCGCGATGTCTTGGCCATGGCCAAGATTGAAGTCAAACTGTCCGA

'BR_f13_B' CTCGCGCGATGTCTTGGCCATGGCCAAGATTGAAGTCAAACTGTCCGA

'BR_f13_A' CTCGCGCGATGTCTTGGCCATGGCCAAGATTGAAGTCAAACTGTCCGA

'BR_m8_B' CTCGCGCGATGTCTTGGCCATGGCCAAGATTGAAGTCAAACTGTCCGA

'BR_m8_A' CTCGCGCGATGTCTTGGCCATGGCCAAGATTGAAGTCAAACTGTCCGA

'SD_h_a' CTCGCGCGATGTCTTGGCCATGGCCAAGATTGAAGTCAAACTGTCCGA

'SD_h_b' CTCGCGCGATGTCTTGGCCATGGCCAAGATTGAAGTCAAACTGTCCGA

'SD_g_a' CTCGCGCGATGTCTTGGCCATGGCCAAGATTGAAGTCAAACTGTCCGA

'SD_g_b' CTCGCGCGATGTCTTGGCCATGGCCAAGATTGAAGTCAAACTGTCCGA

'SD_2m_b' CTCGCGCGATGTCTTGGCCATGGCCAAGATTGAAGTCAAACTGTCCGA

'SD_2m_a' CTCGCGCGATGTCTTGGCCATGGCCAAGATTGAAGTCAAACTGTCCGA

[ 1490 1500 1510 1520 1530 ]

[ * * * * * ]

'RP1_m1_A' TATTCCCGAGGGCAAGAATATGACCTTCAAATGGCGGGGCAAGCCCCT

'RP1_m1_B' TATTCCCGAGGGCAAGAATATGACCTTCAAATGGCGGGGCAAGCCCCT

'RP1_m5(A)' TATTCCCGAGGGCAAGAATATGACCTTCAAATGGCGGGGCAAGCCCCT

'RP1_m5_B' TATTCCCGAGGGCAAGAATATGACCTTCAAATGGCGGGGCAAGCCCCT

'RP1_m7_A' TATTCCCGAGGGCAAGAATATGACCTTCAAATGGCGGGGCAAGCCCCT

'RP1_m7_B' TATTCCCGAGGGCAAGAATATGACCTTCAAATGGCGGGGCAAGCCCCT

'RP1_m8_A' TATTCCCGAGGGCAAGAATATGACCTTCAAATGGCGGGGCAAGCCCCT

'RP1_m8_B' TATTCCCGAGGGCAAGAATATGACCTTCAAATGGCGGGGCAAGCCCCT

'R_Abg' TATTCCCGAGGGCAAGAATATGACCTTCAAATGGCGGGGCAAGCCCCT

'R_Abg_2' TATTCCCGAGGGCAAGAATATGACCTTCAAATGGCGGGGCAAGCCCCT

'AB_m1_A' TATTCCCGAGGGCAAGAATATGACCTTCAAATGGCGGGGCAAGCCCCT

'AB_m1_B' TATTCCCGAGGGCAAGAATATGACCTTCAAATGGCGGGGCAAGCCCCT

Abh TATTCCCGAGGGCAAGAATATGACCTTCAAATGGCGGGGCAAGCCCCT

'Abh_2' TATTCCCGAGGGCAAGAATATGACCTTCAAATGGCGGGGCAAGCCCCT

'AB_m2_A' TATTCCCGAGGGCAAGAATATGACCTTCAAATGGCGGGGCAAGCCCCT

'AB_m2_B' TATTCCCGAGGGCAAGAATATGACCTTCAAATGGCGGGGCAAGCCCCT

'IP_m8_A' TATTCCCGAGGGCAAGAATATGACCTTCAAATGGCGGGGCAAGCCCCT

'IP_m8_B' TATTCCCGAGGGCAAGAATATGACCTTCAAATGGCGGGGCAAGCCCCT

'IP_f3_A' TATTCCCGAGGGCAAGAATATGACCTTCAAATGGCGGGGCAAGCCCCT

'IP_f3_B' TATTCCCGAGGGCAAGAATATGACCTTCAAATGGCGGGGCAAGCCCCT

'IP_f23_A' TATTCCCGAGGGCAAGAATATGACCTTCAAATGGCGGGGCAAGCCCCT

'IP_f23_B' TATTCCCGAGGGCAAGAATATGACCTTCAAATGGCGGGGCAAGCCCCT

'IP_m7_A' TATTCCCGAGGGCAAGAATATGACCTTCAAATGGCGGGGCAAGCCCCT

'IP_m7_B' TATTCCCGAGGGCAAGAATATGACCTTCAAATGGCGGGGCAAGCCCCT

'PVL_f14_A' TATTCCCGAGGGCAAGAATATGACCTTCAAATGGCGGGGCAAGCCCCT

'PVL_f14_B' TATTCCCGAGGGCAAGAATATGACCTTCAAATGGCGAGGCAAGCCCCT

'PVL_f12_A' TATTCCCGAGGGCAAGAATATGACCTTCAAATGGCGGGGCAAGCCCCT

'PVL_f12_B' TATTCCCGAGGGCAAGAATATGACCTTCAAATGGCGGGGCAAGCCCCT

'PVL_f3_A' TATTCCCGAGGGCAAGAATATGACCTTCAAATGGCGAGGCAAGCCCCT

'PVL_f3_B' TATTCCCGAGGGCAAGAATATGACCTTCAAATGGCGGGGCAAGCCCCT

'PVL_f7_A' TATTCCCGAGGGCAAGAATATGACCTTCAAATGGCGGGGCAAGCCCCT

'PVL_f7_B' TATTCCCGAGGGCAAGAATATGACCTTCAAATGGCGGGGCAAGCCCCT

'Pes_m9_A' TATTCCCGAGGGCAAGAATATGACCTTCAAATGGCGAGGCAAGCCCCT

'Pes_m9_B' TATTCCCGAGGGCAAGAATATGACCTTCAAATGGCGAGGCAAGCCCCT

'Pes_m10_A' TATTCCCGAGGGCAAGAATATGACCTTCAAATGGCGAGGCAAGCCCCT

'Pes_m10_B' TATTCCCGAGGGCAAGAATATGACCTTCAAATGGCGAGGCAAGCCCCT

'LH_m1_A' TATTCCCGAGGGCAAGAATATGACCTTCAAATGGCGAGGCAAGCCCCT

'LH_m1_B' TATTCCCGAGGGCAAGAATATGACCTTCAAATGGCGAGGCAAGCCCCT

'LH_f6_A' TATTCCCGAGGGCAAGAATATGACCTTCAAATGGCGAGGCAAGCCCCT

'LH_f6_B' TATTCCCGAGGGCAAGAATATGACCTTCAAATGGCGAGGCAAGCCCCT

'LH_f8_A' TATTCCCGAGGGCAAGAATATGACCTTCAAATGGCGAGGCAAGCCCCT

'LH_f8_B' TATTCCCGAGGGCAAGAATATGACCTTCAAATGGCGAGGCAAGCCCCT

'scn_h_a' TATTCCCGAGGGCAAGAATATGACCTTCAAATGGCGAGGCAAGCCCCT

'scn_h_b' TATTCCCGAGGGCAAGAATATGACCTTCAAATGGCGAGGCAAGCCCCT

'scn_m5_A' TATTCCCGAGGGCAAGAATATGACCTTCAAATGGCGAGGCAAGCCCCT

'scn_m5_B' TATTCCCGAGGGCAAGAATATGACCTTCAAATGGCGAGGCAAGCCCCT

'scn_m7_A' TATTCCCGAGGGCAAGAATATGACCTTCAAATGGCGAGGCAAGCCCCT

'scn_m7_B' TATTCCCGAGGGCAAGAATATGACCTTCAAATGGCGAGGCAAGCCCCT

'scn_m3_A' TATTCCCGAGGGCAAGAATATGACCTTCAAATGGCGAGGCAAGCCCCT

'scn_m3_B' TATTCCCGAGGGCAAGAATATGACCTTCAAATGGCGAGGCAAGCCCCT

'LJS_m1_A' TATTCCCGAGGGCAAGAATATGACCTTCAAATGGCGAGGCAAGCCCCT

'LJS_m1_B' TATTCCCGAGGGCAAGAATATGACCTTCAAATGGCGAGGCAAGCCCCT

'LJS_m2_A' TATTCCCGAGGGCAAGAATATGACCTTCAAATGGCGAGGCAAGCCCCT

'LJS_m2_B' TATTCCCGAGGGCAAGAATATGACCTTCAAATGGCGAGGCAAGCCCCT

'LJS_f10_A' TATTCCCGAGGGCAAGAATATGACCTTCAAATGGCGAGGCAAGCCCCT

'LJS_f10_B' TATTCCCGAGGGCAAGAATATGACCTTCAAATGGCGAGGCAAGCCCCT

'LJS_f9_B' TATTCCCGAGGGCAAGAATATGACCTTCAAATGGCGAGGCAAGCCCCT

'LJS_f9_A' TATTCCCGAGGGCAAGAATATGACCTTCAAATGGCGAGGCAAGCCCCT

'LJP_m7_B' TATTCCCGAGGGCAAGAATATGACCTTCAAATGGCGAGGCAAGCCCCT

'LJP_m7_A' TATTCCCGAGGGCAAGAATATGACCTTCAAATGGCGAGGCAAGCCCCT

'LJP_m6_B' TATTCCCGAGGGCAAGAATATGACCTTCAAATGGCGAGGCAAGCCCCT

'LJP_m6_A' TATTCCCGAGGGCAAAAATATGACCTTCAAATGGCGAGGCAAGCCCCT

'LJP_m2_B' TATTCCCGAGGGCAAGAATATGACCTTCAAATGGCGAGGCAAGCCCCT

'LJP_m2_A' TATTCCCGAGGGCAAGAATATGACCTTCAAATGGCGAGGCAAGCCCCT

'LJP_m1_B' TATTCCCGAGGGCAAGAATATGACCTTCAAATGGCGAGGCAAGCCCCT

'LJP_m1_A' TATTCCCGAGGGCAAGAATATGACCTTCAAATGGCGAGGCAAGCCCCT

'BR_m5_A' TATTCCCGAGGGCAAGAATATGACCTTCAAATGGCGAGGCAAGCCCCT

'BR_m5_B' TATTCCCGAGGGCAAGAATATGACCTTCAAATGGCGAGGCAAGCCCCT

'BR_m6_A' TATTCCCGAGGGCAAGAATATGACCTTCAAATGGCGAGGCAAGCCCCT

'BR_m6_B' TATTCCCGAGGGCAAGAATATGACCTTCAAATGGCGAGGCAAGCCCCT

'BR_m7_A' TATTCCCGAGGGCAAGAATATGACCTTCAAATGGCGAGGCAAGCCCCT

'BR_m7_B' TATTCCCGAGGGCAAGAATATGACCTTCAAATGGCGAGGCAAGCCCCT

'BR_f13_B' TATTCCCGAGGGCAAGAATATGACCTTCAAATGGCGAGGCAAGCCCCT

'BR_f13_A' TATTCCCGAGGGCAAGAATATGACCTTCAAATGGCGAGGCAAGCCCCT

'BR_m8_B' TATTCCCGAGGGCAAGAATATGACCTTCAAATGGCGAGGCAAGCCCCT

'BR_m8_A' TATTCCCGAGGGCAAGAATATGACCTTCAAATGGCGAGGCAAGCCCCT

'SD_h_a' TATTCCCGAGGGCAAGAATATGACCTTCAAATGGCGAGGCAAGCCCCT

'SD_h_b' TATTCCCGAGGGCAAGAATATGACCTTCAAATGGCGAGGCAAGCCCCT

'SD_g_a' TATTCCCGAGGGCAAGAATATGACCTTCAAATGGCGAGGCAAGCCCCT

'SD_g_b' TATTCCCGAGGGCAAGAATATGACCTTCAAATGGCGAGGCAAGCCCCT

'SD_2m_b' TATTCCCGAGGGCAAGAATATGACCTTCAAATGGCGAGGCAAGCCCCT

'SD_2m_a' TATTCCCGAGGGCAAGAATATGACCTTCAAATGGCGAGGCAAGCCCCT

[ 1540 1550 1560 1570 1580 ]

[ * * * * * ]

'RP1_m1_A' TTTCGTCCGACATAGGTGGGTGGAGCACGGTTTGGGTGGGACCTAATT

'RP1_m1_B' TTTCGTCCGACATAGGTGGGTGGAGCACGGTTTGGGTGGGACCTAATT

'RP1_m5(A)' TTTCGTCCGACATAGGTGGGTGGAGCACGGTTTGGGTGGGACCTAATT

'RP1_m5_B' TTTCGTCCGACATAGGTGGGTGGAGCACGGTTTGGGTGGGACCTAATT

'RP1_m7_A' TTTCGTCCGACATAGGTGGGTGGAGCACGGTTTGGGTGGGACCTAATT

'RP1_m7_B' TTTCGTCCGACATAGGTGGGTGGAGCACGGTTTGGGTGGGACCTAATT

'RP1_m8_A' TTTCGTCCGACATAGGTGGGTGGAGCACGGTTTGGGTGGGACCTAATT

'RP1_m8_B' TTTCGTCCGACATAGGTGGGTGGAGCACGGTTTGGGTGGGACCTAATT

'R_Abg' TTTCGTCCGACATAGGTGGGTGGAGCACGGTTTGGGTGGGACCTAATT

'R_Abg_2' TTTCGTCCGACATAGGTGGGTGGAGCACGGTTTGGGTGGGACCTAATT

'AB_m1_A' TTTCGTCCGACATAGGTGGGTGGAGCACGGTTTGGGTGGGACCTAATT

'AB_m1_B' TTTCGTCCGACATAGGTGGGTGGAGCACGGTTTGGGTGGGACCTAATT

Abh TTTCGTCCGACATAGGTGGGTGGAGCACGGTTTGGGTGGGACCTAATT

'Abh_2' TTTCGTCCGACATAGGTGGGTGGAGCACGGTTTGGGTGGGACCTAATT

'AB_m2_A' TTTCGTCCGACATAGGTGGGTGGAGCACGGTTTGGGTGGGACCTAATT

'AB_m2_B' TTTCGTCCGACATAGGTGGGTGGAGCACGGTTTGGGTGGGACCTAATT

'IP_m8_A' TTTCGTCCGACATAGGTGGGTGGAGCACGGTTTGGGTGGGACCTAATT

'IP_m8_B' TTTCGTCCGACATAGGTGGGTGGAGCACGGTTTGGGTGGGACCTAATT

'IP_f3_A' TTTCGTCCGACATAGGTGGGTGGAGCACGGTTTGGGTGGGACCTAATT

'IP_f3_B' TTTCGTCCGACATAGGTGGGTGGAGCACGGTTTGGGTGGGACCTAATT

'IP_f23_A' TTTCGTCCGACATAGGTGGGTGGAGCACGGTTTGGGTGGGACCTAATT

'IP_f23_B' TTTCGTCCGACATAGGTGGGTGGAGCACGGTTTGGGTGGGACCTAATT

'IP_m7_A' TTTCGTCCGACATAGGTGGGTGGAGCACGGTTTGGGTGGGACCTAATT

'IP_m7_B' TTTCGTCCGACATAGGTGGGTGGAGCACGGTTTGGGTGGGACCTAATT

'PVL_f14_A' TTTCGTCCGACATAGGTGGGTGGAGCACGGTTTGGGTGGGACCTAATT

'PVL_f14_B' TTTTGTCCGACATAGGTGGGTGGAGCACGGTTTGGGTGGGACCTAATT

'PVL_f12_A' TTTCGTCCGACATAGGTGGGTGGAGCACGGTTTGGGTGGGACCTAATT

'PVL_f12_B' TTTCGTCCGACATAGGTGGGTGGAGCACGGTTTGGGTGGGACCTAATT

'PVL_f3_A' TTTTGTCCGACATAGGTGGGTGGAGCACGGTTTGGGTGGGACCTAATT

'PVL_f3_B' TTTCGTCCGACATAGGTGGGTGGAGCACGGTTTGGGTGGGACCTAATT

'PVL_f7_A' TTTCGTCCGACATAGGTGGGTGGAGCACGGTTTGGGTGGGACCTAATT

'PVL_f7_B' TTTCGTCCGACATAGGTGGGTGGAGCACGGTTTGGGTGGGACCTAATT

'Pes_m9_A' TTTCGTCCGACATAGGTGGGTGGAGCACGGTTTGGGCGGGACCTAATT

'Pes_m9_B' TTTCGTCCGACATAGGTGGGTGGAGCACGGTTTGGGCGGGACCTAATT

'Pes_m10_A' TTTCGTCCGACATAGGTGGGTGGAGCACGGTTTGGGCGGGACCTAATT

'Pes_m10_B' TTTCGTCCGACATAGGTGGGTGGAGCACGGTTTGGGCGGGACCTAATT

'LH_m1_A' TTTCGTCCGACATAGGTGGGTGGAGCACGGTTTGGGCTGGACCTAATT

'LH_m1_B' TTTCGTCCGACATAGGTGGGTGGAGCACGGTTTGGGCTGGACCTAATT

'LH_f6_A' TTTCGTCCGACATAGGTGGGTGGAGCACGGTTTGGGCTGGACCTAATT

'LH_f6_B' TTTCGTCCGACATAGGTGGGTGGAGCACGGTTTGGGCTGGACCTAATT

'LH_f8_A' TTTCGTCCGACATAGGTGGGTGGAGCACGGTTTGGGCTGGACCTAATT

'LH_f8_B' TTTCGTCCGACATAGGTGGGTGGAGCACGGTTTGGGCTGGACCTAATT

'scn_h_a' TTTCGTCCGACATAGGTGGGTGGAGCACGGTTTGGGCTGGACCTAATT

'scn_h_b' TTTCGTCCGACATAGGTGGGTGGAGCACGGTTTGGGCTGGACCTAATT

'scn_m5_A' TTTCGTCCGACATAGGTGGGTGGAGCACGGTTTGGGCTGGACCTAATT

'scn_m5_B' TTTCGTCCGACATAGGTGGGTGGAGCACGGTTTGGGCTGGACCTAATT

'scn_m7_A' TTTCGTCCGACATAGGTGGGTGGAGCACGGTTTGGGCTGGACCTAATT

'scn_m7_B' TTTCGTCCGACATAGGTGGGTGGAGCACGGTTTGGGCTGGACCTAATT

'scn_m3_A' TTTCGTCCGACATAGGTGGGTGGAGCACGGTTTGGGCTGGACCTAATT

'scn_m3_B' TTTCGTCCGACATAGGTGGGTGGAGCACGGTTTGGGCTGGACCTAATT

'LJS_m1_A' TTTCGTCCGACATAGGTGTGTGGAGCACGGTTTGGGCGGGACTTAATT

'LJS_m1_B' TTTCGTCCGACATAGGTTTGTGGAGCACGGTTTGGGCGGGACTTAATT

'LJS_m2_A' TTTCGTCCGACATAGGTGGGTGGAGCACGGTTTGGGCGGGACTTAATT

'LJS_m2_B' TTTCGTCCGACATAGGTGGGTGGAGCACGGTTTGAGCGGGACTTAATT

'LJS_f10_A' TTTCGTCCGACATAGGTGGGTGGAGCACGGTTTGAGCGGGACTTAATT

'LJS_f10_B' TTTCGTCCGACATAGGTGTGTGGAGCACGGTTTGGGCGGGACTTAATT

'LJS_f9_B' TTTCGTCCGACATAGGTGGGTGGAGCACGGTTTGAGCGGGACTTAATT

'LJS_f9_A' TTTCGTCCGACATAGGTGGGTGGAGCACGGTTTGAGCGGGACTTAATT

'LJP_m7_B' TTTCGTCCGACATAGGTGGGTGGAGCACGGTTTGGGCGGGACTTAATT

'LJP_m7_A' TTTCGTCCGACATAGGTGGGTGGAGCACGGTTTGGGCGGGACTTAATT

'LJP_m6_B' TTTCGTCCGACATAGGTGGGTGGAGCACGGTTTGGGCGGGACTTAATT

'LJP_m6_A' TTTCGTCCGACATAGGTGGGTGGAGCACGGTTTGGGCGGGACTTAATT

'LJP_m2_B' TTTCGTCCGACATAGGTGGGTGGAGCACGGTTTGGGCGGGACTTAATT

'LJP_m2_A' TTTCGTCCGACATAGGTGGGTGGAGCACGGTTTGGGCGGGACTTAATT

'LJP_m1_B' TTTCGTCCGACATAGGTGGGTGGAGCACGGTTTGGGCGGGACTTAATT

'LJP_m1_A' TTTCGTCCGACATAGGTGGGTGGAGCACGGTTTGGGCGGGACTTAATT

'BR_m5_A' TTTCGTCCGACATAGGTGGGTGGAGCACGGTTTGGGCGAGACTTAATT

'BR_m5_B' TTTCGTCCGACATAGGTGGGTGGAGCACGGTTTGGGCGAGACTTAATT

'BR_m6_A' TTTCGTCCGACATAGGTGGGTGGAGCACGGTTTGGGCGGGACTTAATT

'BR_m6_B' TTTCGTCCGACATAGGTGGGTGGAGCACGGTTTGGGCGGGACTTAATT

'BR_m7_A' TTTCGTCCGACATAGGTGGGTGGAGCACGGTTTGGGCGGGACTTAATT

'BR_m7_B' TTTCGTCCGACATAGGTGGGTGGAGCACGGTTTGGGCGGGACTTAATT

'BR_f13_B' TTTCGTCCGACATAGGTGGGTGGAGCACGGTTTGGGCGGGACTTAATT

'BR_f13_A' TTTCGTCCGACATAGGTGGGTGGAGCACGGTTTGGGCGGGACTTAATT

'BR_m8_B' TTTCGTCCGACATAGGTGGGTGGAGCACGGTTTGGGCGGGACTTAATT

'BR_m8_A' TTTCGTCCGACATAGGTGGGTGGAGCACGGTTTGGGCGGGACTTAATT

'SD_h_a' TTTCGTCCGACATAGGTGGGTGGAGCACGGTTTGGGCGGGACTTAATT

'SD_h_b' TTTCGTCCGACATAGGTGGGTGGAGCACGGTTTGGGCGGGACTTAATT

'SD_g_a' TTTCGTCCGACATAGGTGGGTGGAGCACGGTTTGGGCGGGACTTAATT

'SD_g_b' TTTCGTCCGACATAGGTGGGTGGAGCACGGTTTGGGCGGGACTTAATT

'SD_2m_b' TTTCGTCCGACATAGGTGGGTGGAGCACGGTTTGGGCGGGACTTAATT

'SD_2m_a' TTTCGTCCGACATAGGTGGGTGGAGCACGGTTTGGGCGGGACTTAATT

[ 1590 1600 1610 1620 1630 ]

[ * * * * * ]

'RP1_m1_A' GAAGGTGA---TTGAAACGCCATATTTCAACTGATCTCAATTGTGTTC

'RP1_m1_B' GAAGGTGA---TTGAAACGCCATATTTCAACTGATCTCAATTGTGTTC

'RP1_m5(A)' GAAGGTGA---TTGAAACGCCATATTTCAACTGATCTCAATTGTGTTC

'RP1_m5_B' GAAGGTGA---TTGAAACGCCATATTTCAACTGATCTCAATTGTGTTC

'RP1_m7_A' GAAGGTGA---TTGAAACGCCATATTTCAACTGATCTCAATTGTGTTC

'RP1_m7_B' GAAGGTGA---TTGAAACGCCATATTTCAACTGATCTCAATTGTGTTC

'RP1_m8_A' GAAGGTGA---TTGAAACGCCATATTTCAACTGATCTCAATTGTGTTC

'RP1_m8_B' GAAGGTGA---TTGAAACGCCATATTTCAACTGATCTCAATTGTGTTC

'R_Abg' GAAGGTGA---TTGAAACGCCATATTTCAACTGATCTCAATTGCGTTC

'R_Abg_2' GAAGGTGA---TTGAAACGCCATATTTCAACTGATCTCAATTGCGTTC

'AB_m1_A' GAAGGTGA---TTGAAACGCCATATTTCAACTGATCTCAATTGCGTTC

'AB_m1_B' GAAGGTGA---TTGAAACGCCATATTTCAACTGATCTCAATTGCGTTC

Abh GAAGGTGA---TTGAAACGCCATATTTCAACTGATCTCAATTGCGTTC

'Abh_2' GAAGGTGA---TTGAAACGCCATATTTCAACTGATCTCAATTGCGTTC

'AB_m2_A' GAAGGTGA---TTGAAACGCCATATTTCAACTGATCTCAATTGCGTTC

'AB_m2_B' GAAGGTGA---TTGAAACGCCATATTTCAACTGATCTCAATTGCGTTC

'IP_m8_A' GAAGGTGA---TTGAAACGCCATATTTCAACTGATCTCAATTGTGTTC

'IP_m8_B' GAAGGTGA---TTGAAACGCCATATTTCAACTGATCTCAATTGTGTTC

'IP_f3_A' GAAGGTGA---TTGAAACGCCATATTTCAACTGATCTCAATTGTGTTC

'IP_f3_B' GAAGGTGA---TTGAAACGCCATATTTCAACTGATCTCAATTGTGTTC

'IP_f23_A' GAAGGTGA---TTGAAACGCCATATTTCAACTGATCTCAATTGTGTTC

'IP_f23_B' GAAGGTGA---TTGAAACGCCATATTTCAACTGATCTCAATTGTGTTC

'IP_m7_A' GAAGGTGA---TTGAAACGCCATATTTCAACTGATCTCAATTGTGTTC

'IP_m7_B' GAAGGTGA---TTGAAACGCCATATTTCAACTGATCTCAATTGTGTTC

'PVL_f14_A' GAAGGTGA---TTGAAACGCCATATTTCAACTGATCTCAATTGTGTTC

'PVL_f14_B' GAAGGTGA---TTGAAACGCCATATTTCAACTGATCTCAATTGTGTTC

'PVL_f12_A' GAAGGTGA---TTGAAACGCCATATTTCAACTGATCTCAATTGTGTTC

'PVL_f12_B' GAAGGTGA---TTGAAACGCCATATTTCAACTGATCTCAATTGTGTTC

'PVL_f3_A' GAAGGTGA---TTGAAACGCCATATTTCAACTGATCTCAATTGTGTTC

'PVL_f3_B' GAAGGTGA---TTGAAACGCCATATTTCAACTGATCTCAATTGTGTTC

'PVL_f7_A' GAAGGTGA---TTGAAACGCCATATTTCAACTGATCTCAATTGTGTTC

'PVL_f7_B' GAAGGTGA---TTGAAACGCCATATTTCAACTGATCTCAATTGTGTTC

'Pes_m9_A' GAAGGTGA---TTGAAACGCCATATTTCAACTGATTTCAATTGCATTC

'Pes_m9_B' GAAGGTGA---TTGAAACGCCATATTTCAACTGATTTCAATTGCATTC

'Pes_m10_A' GAAGGTGA---TTGAAACGCCATATTTCAACTGATTTCAATTGCATTC

'Pes_m10_B' GAAGGTGA---TTGAAACGCCATATTTCAACTGATTTCAATTGCATTC

'LH_m1_A' GAAGGTGA---TTGAAACGCCATATTTCAACTGATCTCAATTGCATTC

'LH_m1_B' GAAGGTGA---TTGAAACGCCATATTTCAACTGATCTCAATTGCATTC

'LH_f6_A' GAAGGTGA---TTGAAACGCCATATTTCAACTGATCTCAATTGCATTC

'LH_f6_B' GAAGGTGA---TTGAAACGCCATATTTCAACTGATCTCAATTGCATTC

'LH_f8_A' GAAGGTGA---TTGAAACGCCATATTTCAACTGATCTCAATTGCATTC

'LH_f8_B' GAAGGTGA---TTGAAACGCCATATTTCAACTGATCTCAATTGCATTC

'scn_h_a' GAAGGTGAAGGTTGAAACGCCATATTTCAACTGATCTCAATTGCATTC

'scn_h_b' GAAGGCGA---TGGAAACGCCATATTTCAACTGATCTCAATTGCATTC

'scn_m5_A' GAAGGTGA---TTGAAACGCCATATTTCAACTGATCTCAATTGCATTC

'scn_m5_B' GAAGGTGA---TTGAAACGCCATATTTCAACTGATCTCAATTGCATTC

'scn_m7_A' GAAGGTGA---TTGAAACGCCATATTTCAACTGATCTCAATTGCATTC

'scn_m7_B' GAAGGTGA---TTGAAACGCCATATTTCAACTGATCTCAATTGCATTC

'scn_m3_A' GAAGGTGA---TTGAAACGCCATATTTCAACTGATCTCAATTGCATTC

'scn_m3_B' GAAGGTGA---TTGAAACGCCATATTTCAACTGATCTCAATTGCATTC

'LJS_m1_A' GAAGGTGA---TTGAAACGCCATATTTCAACTGATCTCAATTGCGTTC

'LJS_m1_B' GAAGGTGA---TTGAAACGCCATATTTCAACTGATCTCAATTGCGTTC

'LJS_m2_A' GAAGGTGA---TTGAAACGCCATATTTCAACTGATCTCAATTGCGTTC

'LJS_m2_B' GAAGGTGA---TTGAAACGCCATATTTCAACTGATCTCAATTGCGTTC

'LJS_f10_A' GAAGGTGA---TTGAAACGCCATATTTCAACTGATCTCAATTGCGTTC

'LJS_f10_B' GAAGGTGA---TTGAAACGCCATATTTCAACTGATCTCAATTGCGTTC

'LJS_f9_B' GAAGGTGA---TTGAAACGCCATATTTCAACTGATCTCAATTGCGTTC

'LJS_f9_A' GAAGGTGA---TTGAAACGCCATATTTCAACTGATCTCAATTGCGTTC

'LJP_m7_B' GAAGGTGA---TTGAAACGCCATATTTCAACTGATCTCAATTGCGTTC

'LJP_m7_A' GAAGGTGA---TTGAAACGCCATATTTCAACTGATCTCAATTGCGTTC

'LJP_m6_B' GAAGGTGA---TTGAAACGCCATATTTCAACTGATCTCAATTGCGTTC

'LJP_m6_A' GAAGGTGA---TTGAAACGCCATATTTCAACTGATCTCAATTGCGTTC

'LJP_m2_B' GAAGGTGA---TTGAAACGCCATATTTCAACTGATCTCAATTGCGTTC

'LJP_m2_A' GAAGGTGA---TTGAAACGCCATATTTCAACTGATCTCAATTGCGTTC

'LJP_m1_B' GAAGGTGA---TTGAAACGCCATATTTCAACTGATCTCAATTGCGTTC

'LJP_m1_A' GAAGGTGA---TTGAAACGCCATATTTCAACTGATCTCAATTGCGTTC

'BR_m5_A' GAAGGTGA---TTGAAACGCCATATTTCAACTGATCTCAATTGCGTTC

'BR_m5_B' GAAGGTGA---TTGAAACGCCATATTTCAACTGATCTCAATTGCGTTC

'BR_m6_A' GAAGGTGA---TTGAAACGCCATATTTCAACTGATCTCAATTGCGTTC

'BR_m6_B' GAAGGTGA---TTGAAACGCCATATTTCAACTGATCTCAATTGCGTTC

'BR_m7_A' GAAGGTGA---TTGAAACGCCATATTTCAACTGATCTCAATTGCGTTC

'BR_m7_B' GAAGGTGA---TTGAAACGCCATATTTCAACTGATCTCAATTGCGTTC

'BR_f13_B' GAAGGTGA---TTGAAACGCCATATTTCAACTGATCTCAATTGCGTTC

'BR_f13_A' GAAGGTGA---TTGAAACGCCATATTTCAACTGATCTCAATTGCGTTC

'BR_m8_B' GAAGGTGA---TTGAAACGCCATATTTCAACTGATCTCAATTGCGTTC

'BR_m8_A' GAAGGTGA---TTGAAACGCCATATTTCAACTGATCTCAATTGCGTTC

'SD_h_a' GAAGGTGA---TTGAAACGCCATATTTCAACTGATCTCGATTGCGTTC

'SD_h_b' GAAGGTGA---TTGAAACGCCATATTTCAACTGATCTCGATTGCGTTC

'SD_g_a' GAAGGTGA---TTGAAACGCCATATTTCAACTGATCTCGATTGCGTTC

'SD_g_b' GAAGGTGA---TTGAAACGCCATATTTCAACTGATCTCGATTGCGTTC

'SD_2m_b' GAAGGTGA---TTGAAACGCCATATTTCAACTGATCTCGATTGCGTTC

'SD_2m_a' GAAGGTGA---TTGAAACGCCATATTTCAACTGATCTCGATTGCGTTC

[ 1640 1650 1660 1670 1680]

[ * * * * *]

'RP1_m1_A' CAGGACCAGCGCCGAAATTGACACGGAGAACAAAGTGGACGCCACGTC

'RP1_m1_B' CAGGACCAGCGCCGAAATTGACACGGAGAACAAAGTGGACGCCACGTC

'RP1_m5(A)' CAGGACCAGCGCCGAAATTGACACGGAGAACAAAGTGGACGCCACGTC

'RP1_m5_B' CAGGACCAGCGCCGAAATTGACACGGAGAACAAAGTGGACGCCACGTC

'RP1_m7_A' CAGGACCAGCGCCGAAATTGACACGGAGAACAAAGTGGACGCCACGTC

'RP1_m7_B' CAGGACCAGCGCCGAAATTGACACGGAGAACAAAGTGGACGCCACGTC

'RP1_m8_A' CAGGACCAGCGCCGAAATTGACACGGAGAACAAAGTGGACGCCACGTC

'RP1_m8_B' CAGGACCAGCGCCGAAATTGACACGGAGAACAAAGTGGACGCCACGTC

'R_Abg' CAGGACCAGCGCCGAAATTGACACGGAGAACAAAGTGGACGCCACGTC

'R_Abg_2' CAGGACCAGCGCCGAAATTGACACGGAGAACAAAGTGGACGCCACGTC

'AB_m1_A' CAGGACCAGCGCCGAAATTGACACGGAGAACAAAGTGGACGCCACGTC

'AB_m1_B' CAGGACCAGCGCCGAAATTGACACGGAGAACAAAGTGGACGCCACGTC

Abh CAGGACCAGCGCCGAAATTGACACGGAGAACAAAGTGGACGCCACGTC

'Abh_2' CAGGACCAGCGCCGAAATTGACACGGAGAACAAAGTGGACGCCACGTC

'AB_m2_A' CAGGACCAGCGCCGAAATTGACACGGAGAACAAAGTGGACGCCACGTC

'AB_m2_B' CAGGACCAGCGCCGAAATTGACACGGAGAACAAAGTGGACGCCACGTC

'IP_m8_A' CAGGACCAGCGCCGAAATTGACACGGAGAACAAAGTGGACGCCACGTC

'IP_m8_B' CAGGACCAGCGCCGAAATTGACACGGAGAACAAAGTGGACGCCACGTC

'IP_f3_A' CAGGACCAGCGCCGAAATTGACACGGAGAACAAAGTGGACGCCACGTC

'IP_f3_B' CAGGACCAGCGCCGAAATTGACACGGAGAACAAAGTGGACGCCACGTC

'IP_f23_A' CAGGACCAGCGCCGAAATTGACACGGAGAACAAAGTGGACGCCACGTC

'IP_f23_B' CAGGACCAGCGCCGAAATTGACACGGAGAACAAAGTGGACGCCACGTC

'IP_m7_A' CAGGACCAGCGCCGAAATTGACACGGAGAACAAAGTGGACGCCACGTC

'IP_m7_B' CAGGACCAGCGCCGAAATTGACACGGAGAACAAAGTGGACGCCACGTC

'PVL_f14_A' CAGGACCAGCGCCGAAATTGACACGGAGAACAAAGTGGACGCCACGTC

'PVL_f14_B' CAGGACCAGCGCCGAAATTGACACGGAGAACAAAGTGGACGCCACGTC

'PVL_f12_A' CAGGACCAGCGCCGAAATTGACACGGAGAACAAAGTGGACGCCACGTC

'PVL_f12_B' CAGGACCAGCGCCGAAATTGACACGGAGAACAAAGTGGACGCCACGTC

'PVL_f3_A' CAGGACCAGCGCCGAAATTGACACGGAGAACAAAGTGGACGCCACGTC

'PVL_f3_B' CAGGACCAGCGCCGAAATTGACACGGAGAACAAAGTGGACGCCACGTC

'PVL_f7_A' CAGGACCAGCGCCGAAATTGACACGGAGAACAAAGTGGACGCCACGTC

'PVL_f7_B' CAGGACCAGCGCCGAAATTGACACGGAGAACAAAGTGGACGCCACGTC

'Pes_m9_A' CAGGACCAGCGCCGAAATTGACACGGAGAACAAAGTGGACGCCACGTC

'Pes_m9_B' CAGGACCAGCGCCGAAATTGACACGGAGAACAAAGTGGACGCCACGTC

'Pes_m10_A' CAGGACCAGCGCCGAAATTGACACGGAGAACAAAGTGGACGCCACGTC

'Pes_m10_B' CAGGACCAGCGCCGAAATTGACACGGAGAACAAAGTGGACGCCACGTC

'LH_m1_A' CAGGACCAGCGCCGAAATTGACACGGAGAACAAAGTGGACGCCACGTC

'LH_m1_B' CAGGACCAGCGCCGAAATTGACACGGAGAACAAAGTGGACGCCACGTC

'LH_f6_A' CAGGACCAGCGCCGAAATTGACACGGAGAACAAAGTGGACGCCACGTC

'LH_f6_B' CAGGACCAGCGCCGAAATTGACACGGAGAACAAAGTGGACGCCACGTC

'LH_f8_A' CAGGACCAGCGCCGAAATTGACACGGAGAACAAAGTGGACGCCACGTC

'LH_f8_B' CAGGACCAGCGCCGAAATTGACACGGAGAACAAAGTGGACGCCACGTC

'scn_h_a' CAGGACCAGCGCCGAAATTGACACGGAGAACAAAGTGGACGCCACGTC

'scn_h_b' CAGGACCAGCGCCGAAATTGACACGGAGAACAAAGTGGACGCCACGTC

'scn_m5_A' CAGGACCAGCGCCGAAATTGACACGGAGAACAAAGTGGACGCCACGTC

'scn_m5_B' CAGGACCAGCGCCGAAATTGACACGGAGAACAAAGTGGACGCCACGTC

'scn_m7_A' CAGGACCAGCGCCGAAATTGACACGGAGAACAAAGTGGACGCCACGTC

'scn_m7_B' CAGGACCAGCGCCGAAATTGACACGGAGAACAAAGTGGACGCCACGTC

'scn_m3_A' CAGGACCAGCGCCGAAATTGACACGGAGAACAAAGTGGACGCCACGTC

'scn_m3_B' CAGGACCAGCGCCGAAATTGACACGGAGAACAAAGTGGACGCCACGTC

'LJS_m1_A' CAGGACCAGCGCCGAAATTGACACGGAGAATAAAGTGGACGCCACGTC

'LJS_m1_B' CAGGACCAGCGCCGAAATTGACACGGAGAATAAAGTGGACGCCACGTC

'LJS_m2_A' CAGGACCAGCGCCGAAATTGACACGGAGAATAAAGTGGACGCCACGTC

'LJS_m2_B' CAGGACCAGCGCCGAAATTGACACGGAGAATAAAGTGGACGCCACGTC

'LJS_f10_A' CAGGACCAGCGCCGAAATTGACACGGAGAATAAAGTGGACGCCACGTC

'LJS_f10_B' CAGGACCAGCGCCGAAATTGACACGGAGAATAAAGTGGACGCCACGTC

'LJS_f9_B' CAGGACCAGCGCCGAAATTGACACGGAGAATAAAGTGGACGCCACGTC

'LJS_f9_A' CAGGACCAGCGCCGAAATTGACACGGAGAATAAAGTGGACGCCACGTC

'LJP_m7_B' CAGGACCAGCGCCGAAATTGACACGGAGAATAAAGTGGACGCCACGTC

'LJP_m7_A' CAGGACCAGCGCCGAAATTGACACGGAGAATAAAGTGGACGCCACGTC

'LJP_m6_B' CAGGACCAGCGCCGAAATTGACACGGAGAATAAAGTGGACGCCACGTC

'LJP_m6_A' CAGGACCAGCGCCGAAATTGACACGGAGAATAAAGTGGACGCCACGTC

'LJP_m2_B' CAGGACCAGCGCCGAAATTGACACGGAGAATAAAGTGGACGCCACGTC

'LJP_m2_A' CAGGACCAGCGCCGAAATTGACACGGAGAATAAAGTGGACGCCACGTC

'LJP_m1_B' CAGGACCAGCGCCGAAATTGACACGGAGAATAAAGTGGACGCCACGTC

'LJP_m1_A' CAGGACCAGCGCCGAAATTGACACGGAGAATAAAGTGGACGCCACGTC

'BR_m5_A' CAGGACCAGCGCCGAAATTGACACGGAGAATAAAGTGGACGCCACGTC

'BR_m5_B' CAGGACCAGCGCCGAAATTGACACGGAGAATAAAGTGGACGCCACGTC

'BR_m6_A' CAGGACCAGCGCCGAAATTGACACGGAGAATAAAGTGGACGCCACGTC

'BR_m6_B' CAGGACCAGCGCCGAAATTGACACGGAGAATAAAGTGGACGCCACGTC

'BR_m7_A' CAGGACCAGCGCCGAAATTGACACGGAGAATAAAGTGGACGCCACGTC

'BR_m7_B' CAGGACCAGCGCCGAAATTGACACGGAGAATAAAGTGGACGCCACGTC

'BR_f13_B' CAGGACCAGCGCCGAAATTGACACGGAGAATAAAGTGGACGCCACGTC

'BR_f13_A' CAGGACCAGCGCCGAAATTGACACGGAGAATAAAGTGGACGCCACGTC

'BR_m8_B' CAGGACCAGCGCCGAAATTGACACGGAGAATAAAGTGGACGCCACGTC

'BR_m8_A' CAGGACCAGCGCCGAAATTGACACGGAGAATAAAGTGGACGCCACGTC

'SD_h_a' CAGGACCAGCGCCGAAATTGACACGGAGAATAAAGTGGACGCCACGTC

'SD_h_b' CAGGACCAGCGCCGAAATTGACACGGAGAATAAAGTGGACGCCACGTC

'SD_g_a' CAGGACCAGCGCCGAAATTGACACGGAGAATAAAGTGGACGCCACGTC

'SD_g_b' CAGGACCAGCGCCGAAATTGACACGGAGAATAAAGTGGACGCCACGTC

'SD_2m_b' CAGGACCAGCGCCGAAATTGACACGGAGAATAAAGTGGACGCCACGTC

'SD_2m_a' CAGGACCAGCGCCGAAATTGACACGGAGAATAAAGTGGACGCCACGTC

[ 1690 1700 1710 1720 ]

[ * * * * ]

'RP1_m1_A' GTTGCGCGATCCTCAAACCGACGAAGAGCGCGTCAAGGACCCCAAGTT

'RP1_m1_B' GTTGCGCGATCCTCAAACCGACGAAGAGCGCGTCAAGGACCCCAAGTT

'RP1_m5(A)' GTTGCGCGATCCTCAAACCGACGAAGAGCGCGTCAAGGACCCCAAGTT

'RP1_m5_B' GTTGCGCGATCCTCAAACCGACGAAGAGCGCGTCAAGGACCCCAAGTT

'RP1_m7_A' GTTGCGCGATCCTCAAACCGACGAAGAGCGCGTCAAGGACCCCAAGTT

'RP1_m7_B' GTTGCGCGATCCTCAAACCGACGAAGAGCGCGTCAAGGACCCCAAGTT

'RP1_m8_A' GTTGCGCGATCCTCAAACCGACGAAGAGCGCGTCAAGGACCCCAAGTT

'RP1_m8_B' GTTGCGCGATCCTCAAACCGACGAAGAGCGCGTCAAGGACCCCAAGTT

'R_Abg' GTTGCGCGATCCTCAAACCGACGAAGAGCGGGTCAAGGACCCCAAGTT

'R_Abg_2' GTTGCGCGATCCTCAAACCGACGAAGAGCGGGTCAAGGACCCCAAGTT

'AB_m1_A' GTTGCGCGATCCTCAAACCGACGAAGAGCGGGTCAAGGACCCCAAGTT

'AB_m1_B' GTTGCGCGATCCTCAAACCGACGAAGAGCGGGTCAAGGACCCCAAGTT

Abh GTTGCGCGATCCTCAAACCGACGAAGAGCGGGTCAAGGACCCCAAGTT

'Abh_2' GTTGCGCGATCCTCAAACCGACGAAGAGCGGGTCAAGGACCCCAAGTT

'AB_m2_A' GTTGCGCGATCCTCAAACCGACGAAGAGCGGGTCAAGGACCCCAAGTT

'AB_m2_B' GTTGCGCGATCCTCAAACCGACGAAGAGCGGGTCAAGGACCCCAAGTT

'IP_m8_A' GTTGCGCGATCCTCAAACCGACGAAGAGCGCGTCAAGGACCCCAAGTT

'IP_m8_B' GTTGCGCGATCCTCAAACCGACGAAGAGCGCGTCAAGGACCCCAAGTT

'IP_f3_A' GTTGCGCGATCCTCAAACCGACGAAGAGCGCGTCAAGGACCCCAAGTT

'IP_f3_B' GTTGCGCGATCCTCAAACCGACGAAGAGCGCGTCAAGGACCCCAAGTT

'IP_f23_A' GTTGCGCGATCCTCAAACCGACGAAGAGCGCGTCAAGGACCCCAAGTT

'IP_f23_B' GTTGCGCGATCCTCAAACCGACGAAGAGCGGGTCAAGGACCCCAAGTT

'IP_m7_A' GTTGCGCGATCCTCAAACCGACGAAGAGCGCGTCAAGGACCCCAAGTT

'IP_m7_B' GTTGCGCGATCCTCAAACCGACGAAGAGCGCGTCAAGGACCCCAAGTT

'PVL_f14_A' GTTGCGCGATCCTCAAACCGACGAAGAGCGCGTCAAGGACCCCAAGTT

'PVL_f14_B' GTTGCGCGATCCTCAAACCGACGAAGAGCGCGTCAAGGACCCCAAGTT

'PVL_f12_A' GTTGCGCGATCCTCAAACCGACGAAGAGCGCGTCAAGGACCCCAAGTT

'PVL_f12_B' GTTGCGCGATCCTCAAACCGACGAAGAGCGCGTCAAGGACCCCAAGTT

'PVL_f3_A' GTTGCGCGATCCTCAAACCGACGAAGAGCGCGTCAAGGACCCCAAGTT

'PVL_f3_B' GTTGCGCGATCCTCAAACCGACGAAGAGCGCGTCAAGGACCCCAAGTT

'PVL_f7_A' GTTGCGCGATCCTCAAACCGACGAAGAGCGCGTCAAGGACCCCAAGTT

'PVL_f7_B' GTTGCGCGATCCTCAAACCGACGAAGAGCGCGTCAAGGACCCCAAGTT

'Pes_m9_A' GTTGCGCGATCCTCAAACCGACGAAGAGCGCGTCAAGGACCCCAAGTT

'Pes_m9_B' GTTGCGCGATCCTCAAACCGACGAAGAGCGCGTCAAGGACCCCAAGTT

'Pes_m10_A' GTTGCGCGATCCTCAAACCGACGAAGAGCGCGTCAAGGACCCCAAGTT

'Pes_m10_B' GTTGCGCGATCCTCAAACCGACGAAGAGCGCGTCAAGGACCCCAAGTT

'LH_m1_A' GTTGCGCGATCCTCAAACCGACGAAGAGCGCGTCAAGGACCCCAAGTT

'LH_m1_B' GTTGCGCGATCCTCAAACCGACGAAGAGCGCGTCAAGGACCCCAAGTT

'LH_f6_A' GTTGCGCGATCCTCAAACCGACGAAGAGCGCGTCAAGGACCCCAAGTT

'LH_f6_B' GTTGCGCGATCCTCAAACCGACGAAGAGCGCGTCAAGGACCCCAAGTT

'LH_f8_A' GTTGCGCGATCCTCAAACCGACGAAGAGCGCGTCAAGGACCCCAAGTT

'LH_f8_B' GTTGCGCGATCCTCAAACCGACGAAGAGCGCGTCAAGGACCCCAAGTT

'scn_h_a' GTTGCGCGATCCTCAAACCGACGAAGAGCGCGTCAAGGACCCCAAGTT

'scn_h_b' GTTGCGCGATCCTCAAACCGACGAAGAGCGCGTCAAGGACCCCAAGTT

'scn_m5_A' GTTGCGCGATCCTCAAACCGACGAAGAGCGCGTCAAGGACCCCAAGTT

'scn_m5_B' GTTGCGCGATCCTCAAACCGACGAAGAGCGCGTCAAGGACCCCAAGTT

'scn_m7_A' GTTGCGCGATCCTCAAACCGACGAAGAGCGCGTCAAGGACCCCAAGTT

'scn_m7_B' GTTGCGCGATCCTCAAACCGACGAAGAGCGCGTCAAGGACCCCAAGTT

'scn_m3_A' GTTGCGCGATCCTCAAACCGACGAAGAGCGCGTCAAGGACCCCAAGTT

'scn_m3_B' GTTGCGCGATCCTCAAACCGACGAAGAGCGCGTCAAGGACCCCAAGTT

'LJS_m1_A' GTTGCGCGATCCTCAAACCGACGAAGAGCGCGTTAAGGACCCCAAGTT

'LJS_m1_B' GTTGCGCGATCCTCAAACCGACGAAGAGCGCGTTAAGGACCCCAAGTT

'LJS_m2_A' GTTGCGCGATCCTCAAACCGACGAAGAGCGCGTTAAGGACCCCAAGTT

'LJS_m2_B' GTTGCGCGATCCTCAAACCGACGAAGAGCGCGTTAAGGACCCCAAGTT

'LJS_f10_A' GTTGCGCGATCCTCAAACCGACGAAGAGCGCGTTAAGGACCCCAAGTT

'LJS_f10_B' GTTGCGCGATCCTCAAACCGACGAAGAGCGCGTTAAGGACCCCAAGTT

'LJS_f9_B' GTTGCGCGATCCTCAAACCGACGAAGAGCGCGTTAAGGACCCCAAGTT

'LJS_f9_A' GTTGCGCGATCCTCAAACCGACGAAGAGCGCGTTAAGGACCCCAAGTT

'LJP_m7_B' GTTGCGCGATCCTCAAACCGACGAAGAGCGCGTTAAGGACCCCAAGTT

'LJP_m7_A' GTTGCGCGATCCTCAAACCGACGAAGAGCGCGTTAAGGACCCCAAGTT

'LJP_m6_B' GTTGCGCGATCCTCAAACCGACGAAGAGCGCGTTAAGGACCCCAAGTT

'LJP_m6_A' GTTGCGCGATCCTCAAACCGACGAAGAGCGCGTTAAGGACCCCAAGTT

'LJP_m2_B' GTTGCGCGATCCTCAAACCGACGAAGAGCGCGTTAAGGACCCCAAGTT

'LJP_m2_A' GTTGCGCGATCCTCAAACCGACGAAGAGCGCGTTAAGGACCCCAAGTT

'LJP_m1_B' GTTGCGCGATCCTCAAACCGACGAAGAGCGCGTTAAGGACCCCAAGTT

'LJP_m1_A' GTTGCGCGATCCTCAAACCGACGAAGAGCGCGTTAAGGACCCCAAGTT

'BR_m5_A' GTTGCGCGATCCTCAAACCGACGAAGAGCGCGTTAAGGACCCCAAGTT

'BR_m5_B' GTTGCGCGATCCTCAAACCGACGAAGAGCGCGTTAAGGACCCCAAGTT

'BR_m6_A' GTTGCGCGATCCTCAAACCGACGAAGAGCGCGTTAAGGACCCCAAGTT

'BR_m6_B' GTTGCGCGATCCTCAAACCGACGAAGAGCGCGTTAAGGACCCCAAGTT

'BR_m7_A' GTTGCGCGATCCTCAAACCGACGAAGAGCGCGTTAAGGACCCCAAGTT

'BR_m7_B' GTTGCGCGATCCTCAAACCGACGAAGAGCGCGTTAAGGACCCCAAGTT

'BR_f13_B' GTTGCGCGATCCTCAAACCGACGAAGAGCGCGTTAAGGACCCCAAGTT

'BR_f13_A' GTTGCGCGATCCTCAAACCGACGAAGAGCGCGTTAAGGACCCCAAGTT

'BR_m8_B' GTTGCGCGATCCTCAAACCGACGAAGAGCGCGTTAAGGACCCCAAGTT

'BR_m8_A' GTTGCGCGATCCTCAAACCGACGAAGAGCGCGTTAAGGACCCCAAGTT

'SD_h_a' GTTGCGCGATCCTCAAACCGACGAAGAGCGCGTTAAGGACCCCAAGTT

'SD_h_b' GTTGCGCGATCCTCAAACCGACGAAGAGCGCGTTAAGGACCCCAAGTT

'SD_g_a' GTTGCGCGATCCTCAAACCGACGAAGAGCGCGTTAAGGACCCCAAGTT

'SD_g_b' GTTGCGCGATCCTCAAACCGACGAAGAGCGCGTTAAGGACCCCAAGTT

'SD_2m_b' GTTGCGCGATCCTCAAACCGACGAAGAGCGCGTTAAGGACCCCAAGTT

'SD_2m_a' GTTGCGCGATCCTCAAACCGACGAAGAGCGCGTTAAGGACCCCAAGTT

[ 1730 1740 1750 1760 1770 ]

[ * * * * * ]

'RP1_m1_A' CTTGATTGTGATCGGTGTGTGCACTCATTTGGGTTGTGTGCCTATTGC

'RP1_m1_B' CTTGATTGTGATCGGTGTGTGCACTCATTTGGGTTGTGTGCCTATTGC

'RP1_m5(A)' CTTGATTGTGATCGGTGTGTGCACTCATTTGGGTTGTGTGCCTATTGC

'RP1_m5_B' CTTGATTGTGATCGGTGTGTGCACTCATTTGGGTTGTGTGCCTATTGC

'RP1_m7_A' CTTGATTGTGATCGGTGTGTGCACTCATTTGGGTTGTGTGCCTATTGC

'RP1_m7_B' CTTGATTGTGATCGGTGTGTGCACTCATTTGGGTTGTGTGCCTATTGC

'RP1_m8_A' CTTGATTGTGATCGGTGTGTGCACTCATTTGGGTTGTGTGCCTATTGC

'RP1_m8_B' CTTGATTGTGATCGGTGTGTGCACTCATTTGGGTTGTGTGCCTATTGC

'R_Abg' CTTGATTGTGATCGGTGTGTGCACTCATTTGGGTTGTGTGCCTATTGC

'R_Abg_2' CTTGATTGTGATCGGTGTGTGCACTCATTTGGGTTGTGTGCCTATTGC

'AB_m1_A' CTTGATTGTGATCGGTGTGTGCACTCATTTGGGTTGTGTGCCTATTGC

'AB_m1_B' CTTGATTGTGATCGGTGTGTGCACTCATTTGGGTTGTGTGCCTATTGC

Abh CTTGATTGTGATCGGTGTGTGCACTCATTTGGGTTGTGTGCCTATTGC

'Abh_2' CTTGATTGTGATCGGTGTGTGCACTCATTTGGGTTGTGTGCCTATTGC

'AB_m2_A' CTTGATTGTGATCGGTGTGTGCACTCATTTGGGTTGTGTGCCTATTGC

'AB_m2_B' CTTGATTGTGATCGGTGTGTGCACTCATTTGGGTTGTGTGCCTATTGC

'IP_m8_A' CTTGATTGTGATCGGTGTGTGCACTCATTTGGGTTGTGTGCCTATTGC

'IP_m8_B' CTTGATTGTGATCGGTGTGTGCACTCATTTGGGTTGTGTGCCTATTGC

'IP_f3_A' CTTGATTGTGATCGGTGTGTGCACTCATTTGGGTTGTGTGCCTATTGC

'IP_f3_B' CTTGATTGTGATCGGTGTGTGCACTCATTTGGGTTGTGTGCCTATTGC

'IP_f23_A' CTTGATTGTGATCGGTGTGTGCACTCATTTGGGTTGTGTGCCTATTGC

'IP_f23_B' CTTGATTGTGATCGGTGTGTGCACTCATTTGGGTTGTGTGCCTATTGC

'IP_m7_A' CTTGATTGTGATCGGTGTGTGCACTCATTTGGGTTGTGTGCCTATTGC

'IP_m7_B' CTTGATTGTGATCGGTGTGTGCACTCATTTGGGTTGTGTGCCTATTGC

'PVL_f14_A' CTTGATTGTGATCGGTGTGTGCACTCATTTGGGTTGTGTGCCTATTGC

'PVL_f14_B' CTTGATTGTGATCGGTGTGTGCACTCATTTGGGTTGTGTGCCTATTGC

'PVL_f12_A' CTTGATTGTGATCGGTGTGTGCACTCATTTGGGTTGTGTGCCTATTGC

'PVL_f12_B' CTTGATTGTGATCGGTGTGTGCACTCATTTGGGTTGTGTGCCTATTGC

'PVL_f3_A' CTTGATTGTGATCGGTGTGTGCACTCATTTGGGTTGTGTGCCTATTGC

'PVL_f3_B' CTTGATTGTGATCGGTGTGTGCACTCATTTGGGTTGTGTGCCTATTGC

'PVL_f7_A' CTTGATTGTGATCGGTGTGTGCACTCATTTGGGTTGTGTGCCTATTGC

'PVL_f7_B' CTTGATTGTGATCGGTGTGTGCACTCATTTGGGTTGTGTGCCTATTGC

'Pes_m9_A' CTTGATTGTGATCGGTGTGTGCACTCACTTGGGTTGTGTGCCTATTGC

'Pes_m9_B' CTTGATTGTGATCGGTGTGTGCACTCACTTGGGTTGTGTGCCTATTGC

'Pes_m10_A' CTTGATTGTGATCGGTGTGTGCACTCACTTGGGTTGTGTGCCTATTGC

'Pes_m10_B' CTTGATTGTGATCGGTGTGTGCACTCACTTGGGTTGTGTGCCTATTGC

'LH_m1_A' CTTGATTGTGATCGGTGTGTGCACTCACTTGGGTTGTGTGCCTATTGC

'LH_m1_B' CTTGATTGTGATCGGTGTGTGCACTCACTTGGGTTGTGTGCCTATTGC

'LH_f6_A' CTTGATTGTGATCGGTGTGTGCACTCACTTGGGTTGTGTGCCTATTGC

'LH_f6_B' CTTGATTGTGATCGGTGTGTGCACTCACTTGGGTTGTGTGCCTATTGC

'LH_f8_A' CTTGATTGTGATCGGTGTGTGCACTCACTTGGGTTGTGTGCCTATTGC

'LH_f8_B' CTTGATTGTGATCGGTGTGTGCACTCACTTGGGTTGTGTGCCTATTGC

'scn_h_a' CTTGATTGTGATCGGTGTGTGCACTCACTTGGGTTGTGTGCCTATTGC

'scn_h_b' CTTGATTGTGATCGGTGTGTGCACTCACTTGGGTTGTGTGCCTATTGC

'scn_m5_A' CTTGATTGTGATCGGTGTGTGCACTCACTTGGGTTGTGTGCCTATTGC

'scn_m5_B' CTTGATTGTGATCGGTGTGTGCACTCACTTGGGTTGTGTGCCTATTGC

'scn_m7_A' CTTGATTGTGATCGGTGTGTGCACTCACTTGGGTTGTGTGCCTATTGC

'scn_m7_B' CTTGATTGTGATCGGTGTGTGCACTCACTTGGGTTGTGTGCCTATTGC

'scn_m3_A' CTTGATTGTGATCGGTGTGTGCACTCACTTGGGTTGTGTGCCTATTGC

'scn_m3_B' CTTGATTGTGATCGGTGTGTGCACTCACTTGGGTTGTGTGCCTATTGC

'LJS_m1_A' CTTGATTGTGATCGGTGTGTGCACTCACTTGGGTTGTGTGCCTATTGC

'LJS_m1_B' CTTGATTGTGATCGGTGTGTGCACTCACTTGGGTTGTGTGCCTATTGC

'LJS_m2_A' CTTGATTGTGATCGGTGTGTGCACTCACTTGGGTTGTGTGCCTATTGC

'LJS_m2_B' CTTGATTGTGATCGGTGTGTGCACTCACTTGGGTTGTGTGCCTATTGC

'LJS_f10_A' CTTGATTGTGATCGGTGTGTGCACTCACTTGGGTTGTGTGCCTATTGC

'LJS_f10_B' CTTGATTGTGATCGGTGTGTGCACTCACTTGGGTTGTGTGCCTATTGC

'LJS_f9_B' CTTGATTGTGATCGGTGTGTGCACTCACTTGGGTTGTGTGCCTATTGC

'LJS_f9_A' CTTGATTGTGATCGGTGTGTGCACTCACTTGGGTTGTGTGCCTATTGC

'LJP_m7_B' CTTGATTGTGATCGGTGTGTGCACTCACTTGGGTTGTGTGCCTATTGC

'LJP_m7_A' CTTGATTGTGATCGGTGTGTGCACTCACTTGGGTTGTGTGCCTATTGC

'LJP_m6_B' CTTGATTGTGATCGGTGTGTGCACTCACTTGGGTTGTGTGCCTATTGC

'LJP_m6_A' CTTGATTGTGATCGGTGTGTGCACTCACTTGGGTTGTGTGCCTATTGC

'LJP_m2_B' CTTGATTGTGATCGGTGTGTGCACTCACTTGGGTTGTGTGCCTATTGC

'LJP_m2_A' CTTGATTGTGATCGGTGTGTGCACTCACTTGGGTTGTGTGCCTATTGC

'LJP_m1_B' CTTGATTGTGATCGGTGTGTGCACTCACTTGGGTTGTGTGCCTATTGC

'LJP_m1_A' CTTGATTGTGATCGGTGTGTGCACTCACTTGGGTTGTGTGCCTATTGC

'BR_m5_A' CTTGATTGTGATCGGTGTGTGCACTCACTTGGGTTGTGTGCCTATTGC

'BR_m5_B' CTTGATTGTGATCGGTGTGTGCACTCACTTGGGTTGTGTGCCTATTGC

'BR_m6_A' CTTGATTGTGATCGGTGTGTGCACTCACTTGGGTTGTGTGCCTATTGC

'BR_m6_B' CTTGATTGTGATCGGTGTGTGCACTCACTTGGGTTGTGTGCCTATTGC

'BR_m7_A' CTTGATTGTGATCGGTGTGTGCACTCACTTGGGTTGTGTGCCTATTGC

'BR_m7_B' CTTGATTGTGATCGGTGTGTGCACTCACTTGGGTTGTGTGCCTATTGC

'BR_f13_B' CTTGATTGTGATCGGTGTGTGCACTCACTTGGGTTGTGTGCCTATTGC

'BR_f13_A' CTTGATTGTGATCGGTGTGTGCACTCACTTGGGTTGTGTGCCTATTGC

'BR_m8_B' CTTGATTGTGATCGGTGTGTGCACTCACTTGGGTTGTGTGCCTATTGC

'BR_m8_A' CTTGATTGTGATCGGTGTGTGCACTCACTTGGGTTGTGTGCCTATTGC

'SD_h_a' CTTGATTGTGATCGGTGTGTGCACTCACTTGGGTTGTGTGCCTATTGC

'SD_h_b' CTTGATTGTGATCGGTGTGTGCACTCACTTGGGTTGTGTGCCTATTGC

'SD_g_a' CTTGATTGTGATCGGTGTGTGCACTCACTTGGGTTGTGTGCCTATTGC

'SD_g_b' CTTGATTGTGATCGGTGTGTGCACTCACTTGGGTTGTGTGCCTATTGC

'SD_2m_b' CTTGATTGTGATCGGTGTGTGCACTCACTTGGGTTGTGTGCCTATTGC

'SD_2m_a' CTTGATTGTGATCGGTGTGTGCACTCACTTGGGTTGTGTGCCTATTGC

[ 1780 1790 1800 1810 1820 ]

[ * * * * * ]

'RP1_m1_A' CAACGCCGGTCAATTTGGGGGCTACTACTGCCCTTGCCATGGCTCCCA

'RP1_m1_B' CAACGCCGGTCAATTTGGGGGCTACTACTGCCCTTGCCATGGCTCCCA

'RP1_m5(A)' CAACGCCGGTCAATTTGGGGGCTACTACTGCCCTTGCCATGGCTCCCA

'RP1_m5_B' CAACGCCGGTCAATTTGGGGGCTACTACTGCCCTTGCCATGGCTCCCA

'RP1_m7_A' CAACGCCGGTCAATTTGGGGGCTACTACTGCCCTTGCCATGGCTCCCA

'RP1_m7_B' CAACGCCGGTCAATTTGGGGGCTACTACTGCCCTTGCCATGGTTCCCA

'RP1_m8_A' CAACGCCGGTCAATTTGGGGGCTACTACTGCCCTTGCCATGGTTCCCA

'RP1_m8_B' CAACGCCGGTCAATTTGGGGGCTACTACTGCCCTTGCCATGGCTCCCA

'R_Abg' CAACGCCGGTCAATTTGGGGGCTACTACTGCCCTTGCCATGGTTCCCA

'R_Abg_2' CAACGCCGGTCAATTTGGGGGCTACTACTGCCCTTGCCATGGTTCCCA

'AB_m1_A' CAACGCCGGTCAATTTGGGGGCTACTACTGCCCTTGCCATGGTTCCCA

'AB_m1_B' CAACGCCGGTCAATTTGGGGGCTACTACTGCCCTTGCCATGGTTCCCA

Abh CAACGCCGGTCAATTTGGGGGCTACTACTGCCCTTGCCATGGTTCCCA

'Abh_2' CAACGCCGGTCAATTTGGGGGCTACTACTGCCCTTGCCATGGTTCCCA

'AB_m2_A' CAACGCCGGTCAATTTGGGGGCTACTACTGCCCTTGCCATGGTTCCCA

'AB_m2_B' CAACGCCGGTCAATTTGGGGGCTACTACTGCCCTTGCCATGGTTCCCA

'IP_m8_A' CAACGCCGGTCAATTTGGGGGCTACTACTGCCCTTGCCATGGTTCCCA

'IP_m8_B' CAACGCCGGTCAATTTGGGGGCTACTACTGCCCTTGCCATGGTTCCCA

'IP_f3_A' CAACGCCGGTCAATTTGGGGGCTACTACTGCCCTTGCCATGGTTCCCA

'IP_f3_B' CAACGCCGGTCAATTTGGGGGCTACTACTGCCCTTGCCATGGTTCCCA

'IP_f23_A' CAACGCCGGTCAATTTGGGGGCTACTACTGCCCTTGCCATGGTTCCCA

'IP_f23_B' CAACGCCGGTCAATTTGGGGGCTACTACTGCCCTTGCCATGGTTCCCA

'IP_m7_A' CAACGCCGGTCAATTTGGGGGCTACTACTGCCCTTGCCATGGTTCCCA

'IP_m7_B' CAACGCCGGTCAATTTGGGGGCTACTACTGCCCTTGCCATGGTTCCCA

'PVL_f14_A' CAACGCCGGTCAATTTGGGGGCTACTACTGCCCTTGCCATGGTTCCCA

'PVL_f14_B' CAACGCCGGTCAATTTGGGGGCTACTACTGCCCTTGCCATGGTTCCCA

'PVL_f12_A' CAACGCCGGTCAATTTGGGGGCTACTACTGCCCTTGCCATGGTTCCCA

'PVL_f12_B' CAACGCCGGTCAATTTGGGGGCTACTACTGCCCTTGCCATGGTTCCCA

'PVL_f3_A' CAACGCCGGTCAATTTGGGGGCTACTACTGCCCTTGCCATGGTTCCCA

'PVL_f3_B' CAACGCCGGTCAATTTGGGGGCTACTACTGCCCTTGCCATGGTTCCCA

'PVL_f7_A' CAACGCCGGTCAATTTGGGGGCTACTACTGCCCTTGCCATGGTTCCCA

'PVL_f7_B' CAACGCCGGTCAATTTGGGGGCTACTACTGCCCTTGCCATGGTTCCCA

'Pes_m9_A' CAACGCCGGTCAATTTGGGGGCTACTACTGCCCTTGCCACGGTTCCCA

'Pes_m9_B' CAACGCCGGTCAATTTGGGGGCTACTACTGCCCTTGCCACGGTTCCCA

'Pes_m10_A' CAACGCCGGTCAATTTGGGGGCTACTACTGCCCTTGCCACGGTTCCCA

'Pes_m10_B' CAACGCCGGTCAATTTGGGGGCTACTACTGCCCTTGCCACGGTTCCCA

'LH_m1_A' CAACGCCGGTCAATTTGGGGGCTACTACTGCCCTTGCCACGGTTCCCA

'LH_m1_B' CAACGCCGGTCAATTTGGGGGCTACTACTGCCCTTGCCACGGTTCCCA

'LH_f6_A' CAACGCCGGTCAATTTGGGGGCTACTACTGCCCTTGCCACGGTTCCCA

'LH_f6_B' CAACGCCGGTCAATTTGGGGGCTACTACTGCCCTTGCCACGGTTCCCA

'LH_f8_A' CAACGCCGGTCAATTTGGGGGCTACTACTGCCCTTGCCACGGTTCCCA

'LH_f8_B' CAACGCCGGTCAATTTGGGGGCTACTACTGCCCTTGCCACGGTTCCCA

'scn_h_a' CAACGCCGGTCAATTTGGGGGCTACTACTGCCCTTGCCACGGTTCCCA

'scn_h_b' CAACGCCGGTCAATTTGGGGGCTACTACTGCCCTTGCCACGGTTCCCA

'scn_m5_A' CAACGCCGGTCAATTTGGGGGCTACTACTGCCCTTGCCACGGTTCCCA

'scn_m5_B' CAACGCCGGTCAATTTGGGGGCTACTACTGCCCTTGCCACGGTTCCCA

'scn_m7_A' CAACGCCGGTCAATTTGGGGGCTACTACTGCCCTTGCCACGGTTCCCA

'scn_m7_B' CAACGCCGGTCAATTTGGTGGCTACTACTGCCCTTGCCACGGTTCCCA

'scn_m3_A' CAACGCCGGTCAATTTGGGGGCTACTACTGCCCTTGCCACGGTTCCCA

'scn_m3_B' CAACGCCGGTCAATTTGGGGGCTACTACTGCCCTTGCCACGGTTCCCA

'LJS_m1_A' CAACGCCGGTCAATTTGGGGGCTACTACTGCCCTTGCCATGGTTCCCA

'LJS_m1_B' CAACGCCGGTCAATTTGGGGGCTACTACTGCCCTTGCCATGGTTCCCA

'LJS_m2_A' CAACGCCGGTCAATTTGGGGGCTACTACTGCCCTTGCCATGGTTCCCA

'LJS_m2_B' CAACGCCGGTCAATTTGGGGGCTACTACTGCCCTTGCCATGGTTCCCA

'LJS_f10_A' CAACGCCGGTCAATTTGGGGGCTACTACTGCCCTTGCCATGGTTCCCA

'LJS_f10_B' CAACGCCGGTCAATTTGGGGGCTACTACTGCCCTTGCCATGGTTCCCA

'LJS_f9_B' CAACGCCGGTCAATTTGGGGGCTACTACTGCCCTTGCCATGGTTCCCA

'LJS_f9_A' CAACGCCGGTCAATTTGGGGGCTACTACTGCCCTTGCCATGGTTCCCA

'LJP_m7_B' CAACGCCGGTCAATTTGGGGGCTACTACTGCCCTTGCCATGGTTCCCA

'LJP_m7_A' CAACGCCGGTCAATTTGGGGGCTACTACTGCCCTTGCCATGGTTCCCA

'LJP_m6_B' CAACGCCGGTCAATTTGGGGGCTACTACTGCCCTTGCCATGGTTCCCA

'LJP_m6_A' CAACGCCGGTCAATTTGGGGGCTACTACTGCCCTTGCCATGGTTCCCA

'LJP_m2_B' CAACGCCGGTCAATTTGGGGGCTACTACTGCCCTTGCCATGGTTCCCA

'LJP_m2_A' CAACGCCGGTCAATTTGGGGGCTACTACTGCCCTTGCCATGGTTCCCA

'LJP_m1_B' CAACGCCGGTCAATTTGGGGGCTACTACTGCCCTTGCCATGGTTCCCA

'LJP_m1_A' CAACGCCGGTCAATTTGGGGGCTACTACTGCCCTTGCCATGGTTCCCA

'BR_m5_A' CAACGCCGGTCAATTTGGGGGCTACTACTGCCCTTGCCATGGTTCCCA

'BR_m5_B' CAACGCCGGTCAATTTGGGGGCTACTACTGCCCTTGCCATGGTTCCCA

'BR_m6_A' CAACGCCGGTCAATTTGGGGGCTACTACTGCCCTTGCCATGGTTCCCA

'BR_m6_B' CAACGCCGGTCAATTTGGGGGCTACTACTGCCCTTGCCATGGTTCCCA

'BR_m7_A' CAACGCCGGTCAATTTGGGGGCTACTACTGCCCTTGCCATGGTTCCCA

'BR_m7_B' CAACGCCGGTCAATTTGGGGGCTACTACTGCCCTT?CCATGGTTCCCA

'BR_f13_B' CAACGCCGGTCAATTTGGGGGCTACTACTGCCCTTGCCATGGTTCCCA

'BR_f13_A' CAACGCCGGTCAATTTGGGGGCTACTACTGCCCTTGCCATGGTTCCCA

'BR_m8_B' CAACGCCGGTCAATTTGGGGGCTACTACTGCCCTTGCCATGGTTCCCA

'BR_m8_A' CAACGCCGGTCAATTTGGGGGCTACTACTGCCCTTGCCATGGTTCCCA

'SD_h_a' CAACGCCGGTCAATTTGGGGGCTACTACTGCCCTTGCCATGGTTCCCA

'SD_h_b' CAACGCCGGTCAATTTGGGGGCTACTACTGCCCTTGCCATGGTTCCCA

'SD_g_a' CAACGCCGGTCAATTTGGGGGCTACTACTGCCCTTGCCATGGTTCCCA

'SD_g_b' CAACGCCGGTCAATTTGGGGGCTACTACTGCCCTTGCCATGGTTCCCA

'SD_2m_b' CAACGCCGGTCAATTTGGGGGCTACTACTGCCCTTGCCATGGTTCCCA

'SD_2m_a' CAACGCCGGTCAATTTGGGGGCTACTACTGCCCTTGCCATGGTTCCCA

[ 1830 1840 1850 1860 1870 ]

[ * * * * * ]

'RP1_m1_A' TTACGACGCTTCCGGTCGGATCCGTAAGGGACCGGCTCCCCTCAATTT

'RP1_m1_B' TTACGACGCTTCCGGTCGGATCCGTAAGGGACCGGCTCCCCTCAATTT

'RP1_m5(A)' TTACGACGCTTCCGGTCGGATCCGTAAGGGACCGGCTCCCCTCAATTT

'RP1_m5_B' TTACGACGCTTCCGGTCGGATCCGTAAGGGACCGGCTCCCCTCAATTT

'RP1_m7_A' TTACGACGCTTCCGGTCGGATCCGTAAGGGACCGGCTCCCCTCAATTT

'RP1_m7_B' TTACGACGCTTCCGGTCGGATCCGTAAGGGACCGGCTCCCCTCAATTT

'RP1_m8_A' TTACGACGCTTCCGGTCGGATCCGTAAGGGACCGGCTCCCCTCAATTT

'RP1_m8_B' TTACGACGCTTCCGGTCGGATCCGTAAGGGACCGGCTCCCCTCAATTT

'R_Abg' TTACGACGCTTCCGGTCGGATCCGTAAGGGACCGGCTCCCCTCAATTT

'R_Abg_2' TTACGACGCTTCCGGTCGGATCCGTAAGGGACCGGCTCCCCTCAATTT

'AB_m1_A' TTACGACGCTTCCGGTCGGATCCGTAAGGGACCGGCTCCCCTCAATTT

'AB_m1_B' TTACGACGCTTCCGGTCGGATCCGTAAGGGACCGGCTCCCCTCAATTT

Abh TTACGACGCTTCCGGTCGGATCCGTAAGGGACCGGCTCCCCTCAATTT

'Abh_2' TTACGACGCTTCCGGTCGGATCCGTAAGGGACCGGCTCCCCTCAATTT

'AB_m2_A' TTACGACGCTTCCGGTCGGATCCGTAAGGGACCGGCTCCCCTCAATTT

'AB_m2_B' TTACGACGCTTCCGGTCGGATCCGTAAGGGACCGGCTCCCCTCAATTT

'IP_m8_A' TTACGACGCTTCCGGTCGGATCCGTAAGGGACCGGCTCCCCTCAATTT

'IP_m8_B' TTACGACGCTTCCGGTCGGATCCGTAAGGGACCGGCTCCCCTCAATTT

'IP_f3_A' TTACGACGCTTCCGGTCGGATCCGTAAGGGACCGGCTCCCCTCAATTT

'IP_f3_B' TTACGACGCTTCCGGTCGGATCCGTAAGGGACCGGCTCCCCTCAATTT

'IP_f23_A' TTACGACGCTTCCGGTCGGATCCGTAAGGGACCGGCTCCCCTCAATTT

'IP_f23_B' TTACGACGCTTCCGGTCGGATCCGTAAGGGACCGGCTCCCCTCAATTT

'IP_m7_A' TTACGACGCTTCCGGTCGGATCCGTAAGGGACCGGCTCCCCTCAATTT

'IP_m7_B' TTACGACGCTTCCGGTCGGATCCGTAAGGGACCGGCTCCCCTCAATT?

'PVL_f14_A' TTACGACGCTTCCGGTCGGATCCGTAAGGGACCGGCTCCCCTCAATTT

'PVL_f14_B' TTACGACGCTTCCGGTCGGATCCGTAAGGGACCGGCTCCCCTCAATTT

'PVL_f12_A' TTACGACGCTTCCGGTCGGATCCGTAAGGGACCGGCTCCCCTCAATTT

'PVL_f12_B' TTACGACGCTTCCGGTCGGATCCGTAAGGGACCGGCTCCCCTCAATTT

'PVL_f3_A' TTACGACGCTTCCGGTCGGATCCGTAAGGGACCGGCTCCCCTCAATTT

'PVL_f3_B' TTACGACGCTTCCGGTCGGATCCGTAAGGGACCGGCTCCCCTCAATTT

'PVL_f7_A' TTACGACGCTTCCGGTCGGATCCGTAAGGGACCGGCTCCCCTCAATTT

'PVL_f7_B' TTACGACGCTTCCGGTCGGATCCGTAAGGGACCGGCTCCCCTCAATTT

'Pes_m9_A' TTACGACGCTTCTGGTCGGATCCGTAAGGGACCGGCTCCCCTCAATTT

'Pes_m9_B' TTACGACGCTTCTGGTCGGATCCGTAAGGGACCGGCTCCCCTCAATTT

'Pes_m10_A' TTACGACGCTTCTGGTCGGATCCGTAAGGGACCGGCTCCCCTCAATTT

'Pes_m10_B' TTACGACGCTTCTGGTCGGATCCGTAAGGGACCGGCTCCCCTCAATTT

'LH_m1_A' TTACGACGCTTCTGGTCGAATCCGTAAGGGACCGGCTCCCCTCAATTT

'LH_m1_B' TTACGACGCTTCTGGTCGAATCCGTAAGGGACCGGCTCCCCTCAATTT

'LH_f6_A' TTACGACGCTTCTGGTCGAATCCGTAAGGGACCGGCTCCCCTCAATTT

'LH_f6_B' TTACGACGCTTCTGGTCGAATCCGTAAGGGACCGGCTCCCCTCAATTT

'LH_f8_A' TTACGACGCTTCTGGTCGAATCCGTAAGGGACCGGCTCCCCTCAATTT

'LH_f8_B' TTACGACGCTTCTGGTCGAATCCGTAAGGGACCGGCTCCCCTCAATTT

'scn_h_a' TTACGACGCTTCTGGTCGAATCCGTAAGGGACCGGCTCCCCTCAATTT

'scn_h_b' TTACGACGCTTCTGGTCGGATCCGTAAGGGACCGGCTCCCCTCAATTT

'scn_m5_A' TTACGACGCTTCTGGTCGAATCCGTAAGGGACCGGCTCCCCTCAATTT

'scn_m5_B' TTACGACGCTTCTGGTCGAATCCGTAAGGGACCGGCTCCCCTCAATTT

'scn_m7_A' TTACGACGCTTCTGGTCGAATCCGTAAGGGACCGGCTCCCCTCAATTT

'scn_m7_B' TTACGACGCTTCTGGTCGAATCCGTAAGGGACCGGCTCCCCTCAATTT

'scn_m3_A' TTACGACGCTTCTGGTCGAATCCGTAAGGGACCGGCTCCCCTCAATTT

'scn_m3_B' TTACGACGCTTCTGGTCGAATCCGTAAGGGACCGGCTCCCCTCAATTT

'LJS_m1_A' TTACGACGCTTCCGGTCGGATCCGTAAGGGACCGGCTCCCCTCAATTT

'LJS_m1_B' TTACGACGCTTCCGGTCGGATCCGTAAGGGACCGGCTCCCCTCAATTT

'LJS_m2_A' TTACGACGCTTCCGGTCGGATCCGTAAGGGACCGGCTCCCCTCAATTT

'LJS_m2_B' TTACGACGCTTCCGGTCGGATCCGTAAGGGACCGGCTCCCCTCAATTT

'LJS_f10_A' TTACGACGCTTCCGGTCGGATCCGTAAGGGACCGGCTCCCCTCAATTT

'LJS_f10_B' TTATGACGCTTCCGGTCGGATCCGTAAGGGACCGGCTCCCCTCAATTT

'LJS_f9_B' TTACGACGCTTCCGGTCGGATCCGTAAGGGACCGGCTCCCCTCAATTT

'LJS_f9_A' TTACGACGCTTCCGGTCGGATCCGTAAGGGACCGGCTCCCCTCAATTT

'LJP_m7_B' TTACGACGCTTCCGGTCGGATCCGTAAGGGACCGGCTCCCCTCAATTT

'LJP_m7_A' TTACGACGCTTCCGGTCGGATCCGTAAGGGACCGGCTCCCCTCAATTT

'LJP_m6_B' TTACGACGCTTCCGGTCGGATCCGTAAGGGACCGGCTCCCCTCAATTT

'LJP_m6_A' TTACGACGCTTCCGGTCGGATCCGTAAGGGACCGGCTCCCCTCAATTT

'LJP_m2_B' TTACGACGCTTCCGGTCGGATCCGTAAGGGACCGGCTCCCCTCAATTT

'LJP_m2_A' TTACGACGCTTCCGGTCGGATCCGTAAGGGACCGGCTCCCCTCAATTT

'LJP_m1_B' TTACGACGCTTCCGGTCGGATCCGTAAGGGACCGGCTCCCCTCAATTT

'LJP_m1_A' TTACGACGCTTCCGGTCGGATCCGTAAGGGACCGGCTCCCCTCAATTT

'BR_m5_A' TTACGACGCTTCCGGTCGGATCCGTAAGGGACCGGCTCCCCTCAATTT

'BR_m5_B' TTACGACGCTTCCGGTCGGATCCGTAAGGGACCGGCTCCCCTCAATTT

'BR_m6_A' TTACGACGCTTCCGGTCGGATCCGTAAGGGACCGGCTCCCCTCAATTT

'BR_m6_B' TTACGACGCTTCCGGTCGGATCCGTAAGGGACCGGCTCCCCTCAATTT

'BR_m7_A' TTACGACGCTTCCGGTCGGATCCGTAAGGGACCGGCTCCCCTCAATTT

'BR_m7_B' ?TACGACGCT?CCGGTCGGATCCGTAAGGGACCGGCTCCCCTCAATT?

'BR_f13_B' TTACGACGCTTCCGGTCGGATCCGTAAGGGACCGGCTCCCCTCAATTT

'BR_f13_A' TTACGACGCTTCCGGTCGGATCCGTAAGGGACCGGCTCCCCTCAATTT

'BR_m8_B' TTACGACGCTTCCGGTCGGATCCGTAAGGGACCGGCTCCCCTCAATTT

'BR_m8_A' TTACGACGCTTCCGGTCGGATCCGTAAGGGACCGGCTCCCCTCAATTT

'SD_h_a' TTACGACGCTTCCGGTCGGATCCGTAAGGGACCGGCTCCCCTCAATTT

'SD_h_b' TTACGACGCTTCCGGTCGGATCCGTAAGGGACCGGCTCCCCTCAATTT

'SD_g_a' TTACGACGCTTCCGGTCGGATCCGTAAGGGACCGGCTCCCCTCAATTT

'SD_g_b' TTACGACGCTTCCGGTCGGATCCGTAAGGGACCGGCTCCCCTCAATTT

'SD_2m_b' TTACGACGCTTCCGGTCGGATCCGTAAGGGACCGGCTCCCCTCAATTT

'SD_2m_a' TTACGACGCTTCCGGTCGGATCCGTAAGGGACCGGCTCCCCTCAATTT

[ 1880 1890 ]

[ * * ]

'RP1_m1_A' GGAGGTGCCC??????????

'RP1_m1_B' GGAGGTGCCC??????????

'RP1_m5(A)' GGAGGTGCCC??????????

'RP1_m5_B' GGAGGTGCCC??????????

'RP1_m7_A' GGAGGTGCCCGAATACGAAT

'RP1_m7_B' GGAGGTG?????????????

'RP1_m8_A' GGAGGTGCCCGAATACGAAT

'RP1_m8_B' GGAGGTGCCCGAATACGAAT

'R_Abg' GGAGGTGCCC??????????

'R_Abg_2' GGAGGTGCCC??????????

'AB_m1_A' GGAGGTGCCCGAATACGA??

'AB_m1_B' GGAGGTGCCCGAATACGA??

Abh GGAGGTGCCC??????????

'Abh_2' GGAGGTGCCC??????????

'AB_m2_A' GGAGGTGCCCGAATACGAAT

'AB_m2_B' GGAGGTGCCCGAATACGAAT

'IP_m8_A' GGAGGTGCCCGAATACGAAT

'IP_m8_B' GGAGGTGCCCG?????????

'IP_f3_A' GGAGGTGCCCGAATACGAAT

'IP_f3_B' GGAGGTGCCCGAATACGAAT

'IP_f23_A' GGAGGTGCCCGAATACGAAT

'IP_f23_B' GGAGGTG?CC??????????

'IP_m7_A' GGAGGTGCCCGAATACGAAT

'IP_m7_B' ????????????????????

'PVL_f14_A' GGAGGTGCCCGAATACGAAT

'PVL_f14_B' GGAGGTGCCCGAATACGAAT

'PVL_f12_A' GGAGGTGCCCGAATACGAAT

'PVL_f12_B' GGAGGTGCCCGAATACGAAT

'PVL_f3_A' GGAGGTGCCCGAATACGAAT

'PVL_f3_B' GGAGGTGCCCGAATACGAAT

'PVL_f7_A' GGAGGTGCCCGAATACGAAT

'PVL_f7_B' GGAGGTGCCCGAATACGAAT

'Pes_m9_A' GGAGGTGCCCGAATACGAAT

'Pes_m9_B' GGAGGTGCCCGAATACGAAT

'Pes_m10_A' GGAGGTGCCCGAATACGAAT

'Pes_m10_B' GGAGGTGCCCGAATACGAAT

'LH_m1_A' GGAGGTGCCCGAATACGAAT

'LH_m1_B' GGAGGTGCCCGAATACGAAT

'LH_f6_A' GGAGGTGCCCGAATACGAAT

'LH_f6_B' GGAGGTGCCCGAATACGAAT

'LH_f8_A' GGAGGTGCCCGAATACGAAT

'LH_f8_B' GGAGGTGCCCGAATACGAAT

'scn_h_a' GGAGGTGCCCG?AT?CGAAT

'scn_h_b' GGAGGTGCCCG?AT?CGAAT

'scn_m5_A' GGAGGTGCCCGAATACGAAT

'scn_m5_B' GGAGGTGCCCGAATACGAAT

'scn_m7_A' GGAGGTGCCCGAATACGAAT

'scn_m7_B' GGAGGTGCCCGAATACGAAT

'scn_m3_A' GGAGGTGCCCGAATACGAAT

'scn_m3_B' GGAGGTGCCCGAATACGAAT

'LJS_m1_A' GGAGGTGCCCGAATACGAAT

'LJS_m1_B' GGAGGTGCCCGAATACGAAT

'LJS_m2_A' GGAGGTGCCCGAATACGAAT

'LJS_m2_B' GGAGGTGCCCGAATACGAAT

'LJS_f10_A' GGAGGTGCCCGAATACGAAT

'LJS_f10_B' GGAGGTGCCCGAATACGAAT

'LJS_f9_B' GGAGGTGCCCGAATACGAAT

'LJS_f9_A' GGAGGTGCCCGAATACGAAT

'LJP_m7_B' GGAGGTGCCCG?????????

'LJP_m7_A' GGAGGTGCCCGAATACGAAT

'LJP_m6_B' GGAGGTGCC???????????

'LJP_m6_A' GGAGGTGCCCGAATACGAAT

'LJP_m2_B' GGAGGTGCCCGAATACGAAT

'LJP_m2_A' GGAGGTGCCCGAATACGAAT

'LJP_m1_B' GGAGGTGCCCGAATACGAAT

'LJP_m1_A' GGAGGTGCCCGAATACGAAT

'BR_m5_A' GGAGGTGCCCGAATACGAAT

'BR_m5_B' GGAGGTGCCC??????????

'BR_m6_A' GGAGGTGCCCGAATACGAAT

'BR_m6_B' GGAGGTGCCC???TACGAAT

'BR_m7_A' GGAGGTGCCCGAATACGAAT

'BR_m7_B' GGAGGTG?????????????

'BR_f13_B' GGAGGTGCCC??????????

'BR_f13_A' GGAGGTGCCCGAATACGAAT

'BR_m8_B' GGAGGTGCCCGAA???????

'BR_m8_A' GGAGGTGCCCGAATACGAAT

'SD_h_a' GGAGGTGCCCGAATACGAAT

'SD_h_b' GGAGGTGCCCGAATACGAAT

'SD_g_a' GGAGGTGCCCGAATACGAAT

'SD_g_b' GGAGGTGCCCGAATACGAAT

'SD_2m_b' ???????????????????T

'SD_2m_a' ???????????????????T

;

END;

BEGIN SETS;

TaxSet rp1 = 1-8;

TaxSet ab = 9-16;

TaxSet IP = 17-24;

TaxSet PVL = 25-32;

TaxSet pes = 33-36;

TaxSet LH = 37-42;

TaxSet scn = 43-50;

TaxSet LJS = 51-58;

TaxSet BR = 67-76;

TaxSet SD = 77-82;

END;

BEGIN CODONS;

CODONPOSSET * UNTITLED =

N: 45-1229 1552-1635,

1: 2-44\3 1232-1550\3 1637-1892\3,

2: 3-42\3 1230-1551\3 1638-1890\3,

3: 1-43\3 1231-1549\3 1636-1891\3;

CODESET * UNTITLED = Universal: all;

END;

BEGIN CODONUSAGE;

END;

BEGIN DnaSP;

Genome= Diploid;

ChromosomalLocation= Autosome;

VariationType= DNA_Seq_Pol;

DnaSPversion= Ver. 4.10.9;

END;
