# Supplementary material for: Investigations of fine-scale phylogeography in Tigriopus californicus reveal historical patterns of population divergence
Source: BMC Evol Biol. 2009 Jun 23;9:139. doi: 10.1186/1471-2148-9-139 (PMC2708153; doi:10.1186/1471-2148-9-139)
Supplement: Additional file 5 — Table S3. Pairwise percent sequence divergence between selected haplotypes for CYTB. [file 1471-2148-9-139-S5.pdf]

Supplemental Table S3. Pairwise percent sequence divergence between selected haplotypes for CYTB

		Tjap korea	Tjap japan	RsPt f4	AB1m	SDh	LJP m4	BSB f1	LJ P f4	BSB f3	LJS f2	FR1 m3	BR m5	PVL f16	BHar f3	SCN1m	IP f1
		1	2	3	4	5	6	7	8	9	10	11	12	13	14	15	16
1	Tjap korea	-															
2	Tjap japan	0.28736	-														
3	RsPt f4	0.29177	0.3113	-													
4	AB1m	0.29667	0.31255	0.016	-												
5	SDh	0.31138	0.30341	0.19786	0.20118	-											
6	LJP m4	0.30531	0.30617	0.20284	0.2061	0.1011	-										
7	BSB f1	0.28364	0.30703	0.20161	0.20411	0.2112	0.20906	-									
8	LJ P f4	0.30752	0.30755	0.20004	0.20331	0.1028	0.00533	0.207	-								
9	BSB f3	0.28364	0.30703	0.20161	0.20411	0.2112	0.20906	0	0.207	-							
10	LJS f2	0.3056	0.3073	0.20936	0.21262	0.1031	0.00624	0.2137	0.01069	0.2137	-						
11	FR1 m3	0.29181	0.31229	0.01246	0.01247	0.2024	0.20469	0.20563	0.2019	0.20563	0.21121	-					
12	BR m5	0.30592	0.30858	0.20578	0.20904	0.1035	0.00797	0.20924	0.00886	0.20924	0.00887	0.20763	-				
13	PVL f16	0.29162	0.31374	0.00447	0.01338	0.2015	0.20478	0.20326	0.20197	0.20326	0.21132	0.01158	0.20773	-			
14	BHar f3	0.28529	0.30858	0.20534	0.20783	0.2133	0.21017	0.00994	0.2081	0.00994	0.21484	0.20936	0.21036	0.20733	-		
15	SCN1m	0.30799	0.31763	0.20045	0.20482	0.2072	0.19795	0.15189	0.19955	0.15189	0.19905	0.20252	0.19735	0.20349	0.15365	-	
16	IP f1	0.29647	0.31419	0.01508	0.00267	0.2019	0.20505	0.20743	0.20227	0.20743	0.21158	0.01155	0.208	0.01246	0.21116	0.20731	-
17	Pes m9	0.313	0.32095	0.19954	0.20374	0.207	0.20219	0.15714	0.20379	0.15714	0.20331	0.20322	0.20159	0.2024	0.15891	0.00885	0.20623
18	CCR2 f3	0.30858	0.31919	0.20309	0.20729	0.2061	0.20042	0.15175	0.20202	0.15175	0.20153	0.20499	0.19982	0.20597	0.1535	0.00354	0.20976
19	Pes m10	0.313	0.32095	0.19954	0.20374	0.207	0.20219	0.15714	0.20379	0.15714	0.20331	0.20322	0.20159	0.2024	0.15891	0.00885	0.20623
20	SCN7m	0.30769	0.31919	0.20397	0.20817	0.2078	0.20307	0.15534	0.20467	0.15534	0.20419	0.20588	0.20248	0.20684	0.1571	0.00619	0.21066
21	Sd1f	0.31104	0.30656	0.19651	0.19979	0.0045	0.09952	0.21501	0.10124	0.21501	0.0997	0.20103	0.10189	0.20012	0.21705	0.20484	0.2005
22	LS m1 cytb	0.31211	0.30504	0.19692	0.2002	0.0027	0.10107	0.21366	0.1028	0.21366	0.10127	0.20142	0.10345	0.2005	0.21569	0.20621	0.2009
23	SCL f4	0.31465	0.30927	0.19753	0.20082	0.0045	0.10284	0.21441	0.10366	0.21441	0.10304	0.20207	0.10524	0.20103	0.21645	0.2088	0.20152
24	LgBch m2	0.31183	0.30273	0.1978	0.2002	0.025	0.09529	0.2136	0.09701	0.2136	0.09546	0.20144	0.09766	0.20055	0.21385	0.1982	0.2009
25	LagBch m1	0.31155	0.30269	0.19884	0.20122	0.025	0.09494	0.21385	0.09666	0.21385	0.0951	0.20246	0.09731	0.20158	0.21411	0.20014	0.20191
26	LJP2 f3	0.30474	0.30651	0.20312	0.20638	0.1022	0.00534	0.20918	0.00801	0.20918	0.00624	0.20496	0.0062	0.20505	0.21031	0.19722	0.20535
27	RP1 f5	0.29667	0.31618	0.0151	0.00799	0.2011	0.20609	0.20647	0.2033	0.20647	0.21262	0.01242	0.20903	0.01247	0.21022	0.20392	0.00708
28	AB3 f11	0.29872	0.31295	0.01867	0.00267	0.2022	0.2072	0.20744	0.2044	0.20744	0.21374	0.01511	0.21015	0.01605	0.21117	0.20768	0.00532
29	BHar f2	0.29037	0.31082	0.2085	0.21169	0.2161	0.21297	0.01169	0.21094	0.01169	0.21756	0.21178	0.21315	0.21024	0.00091	0.15733	0.21518
30	Dume m9	0.30918	0.32083	0.20869	0.20614	0.2022	0.21275	0.22659	0.21161	0.22659	0.21387	0.20795	0.21113	0.20546	0.22429	0.21014	0.20969
31	Dume m10	0.30918	0.32173	0.20779	0.20524	0.2013	0.21184	0.22568	0.21071	0.22568	0.21296	0.20705	0.21022	0.20456	0.22339	0.20924	0.20879
32	Cat 7	0.29296	0.31145	0.19512	0.19439	0.1974	0.2024	0.2234	0.2031	0.2234	0.20441	0.19781	0.20261	0.19708	0.22167	0.19861	0.19593
33	Cat 5 Cytb	0.29204	0.31241	0.19267	0.19194	0.1962	0.20086	0.22105	0.20156	0.22105	0.20383	0.19536	0.20021	0.19462	0.21934	0.19637	0.19348

		Pes m9	CCR2 f3	Pes m10	SCN7m	Sd1f	LS m1 cyt	SCL f4	LgBch m	LagBch m	LJP2 f3	RP1 f5	AB3 f11	BHar f2	Dume m9	Dume m1	Cat 7
		17	18	19	20	21	22	23	24	25	26	27	28	29	30	31	32
17	Pes m9	-															
18	CCR2 f3	0.00884	-														
19	Pes m10	0	0.00884	-													
20	SCN7m	0.01149	0.00442	0.01149	-												
21	Sd1f	0.20463	0.20552	0.20463	0.20639	-											
22	LS m1 cytb	0.20601	0.2069	0.20601	0.2069	0.0018	-										
23	SCL f4	0.20849	0.20949	0.20849	0.20949	0.0036	0.00178	-									
24	LgBch m2	0.20156	0.19889	0.20156	0.19978	0.0223	0.02218	0.02414	-								
25	LagBch m1	0.20348	0.20083	0.20348	0.20171	0.0222	0.02211	0.02407	0	-							
26	LJP2 f3	0.20237	0.19969	0.20237	0.20235	0.1006	0.10213	0.10391	0.09633	0.09597	-						
27	RP1 f5	0.20283	0.20637	0.20283	0.20726	0.1998	0.20016	0.20078	0.20016	0.20119	0.20639	-					
28	AB3 f11	0.20659	0.21013	0.20659	0.21103	0.2009	0.20125	0.20008	0.20125	0.20227	0.2075	0.01063	-				
29	BHar f2	0.16251	0.15718	0.16251	0.16072	0.2199	0.21842	0.21919	0.21662	0.21686	0.21311	0.21404	0.21518	-			
30	Dume m9	0.21281	0.211	0.21281	0.21368	0.1989	0.20125	0.20211	0.20762	0.2068	0.21163	0.2067	0.20792	0.22492	-		
31	Dume m10	0.2119	0.2101	0.2119	0.21278	0.198	0.20034	0.2012	0.20671	0.2059	0.21072	0.20579	0.20702	0.22401	0.0009	-	
32	Cat 7	0.19949	0.19763	0.19949	0.19854	0.1944	0.19589	0.19741	0.1994	0.19887	0.20193	0.19399	0.19594	0.22174	0.02971	0.03063	-
33	Cat 5 Cytb	0.19726	0.19541	0.19726	0.19633	0.1938	0.19538	0.1969	0.19912	0.19835	0.20058	0.19154	0.19349	0.2194	0.03048	0.03139	0.00093
