# Supplementary material for: Investigations of fine-scale phylogeography in Tigriopus californicus reveal historical patterns of population divergence
Source: BMC Evol Biol. 2009 Jun 23;9:139. doi: 10.1186/1471-2148-9-139 (PMC2708153; doi:10.1186/1471-2148-9-139)
Supplement: Additional file 6 — Table S4. FST values for CYTB from Arlequin based on pairwise comparisons of populations (pairwise sequence divergence). [file 1471-2148-9-139-S6.pdf]

Supplemental Table S4. Fst values for CYTB from arlequin based on pairwise comparisons of populations (pairwise sequence divergence). Values that were not significant based on permutation analyses are indicated by (NS). Significance is based on a=0.05 in a Sequential Bonferroni procedure.

		1	2	3	4	5	6	7	8	9	10	11	12	13	14	15
		SD	SunCl	LS	LJS	Naut	BR	SIO	LJP	LJP2	LJP3	RP1	RP2	IP	AB3	AB2
SD	1	0														
SunCl	2	0.49216	0													
LS	3	0.39423	0.5698	0												
LJS	4	0.98504	0.9918	0.99364	0											
Naut	5	0.9829	0.98856	0.99064	0.00128(NS)	0										
BR	6	0.9741	0.97371	0.97627	0.65331	0.57549	0									
SIO	7	0.98706	0.99519	0.99689	0.03077(NS)	0.01475(NS)	0.68082	0								
LJP	8	0.97746	0.97883	0.9806	0.74436	0.70249	0.58722	0.76525	0							
LJP2	9	0.97818	0.98051	0.98293	0.72049	0.65922	0.47991	0.75284	0.53345	0						
LJP3	10	0.98689	0.99699	0.99903	-0.0063(NS)	-0.02161(NS)	0.66299	0.02005(NS)	0.75538	0.74396	0					
RP1	11	0.98842	0.98929	0.99084	0.9916	0.99003	0.98378	0.99301	0.98646	0.98626	0.99269	0				
RP2	12	0.98859	0.98962	0.99115	0.99179	0.99024	0.98394	0.99321	0.98659	0.98645	0.99294	0.08497(NS)	0			
IP	13	0.99096	0.99389	0.99517	0.99453	0.99311	0.9867	0.99596	0.98879	0.98939	0.9963	0.72104	0.73923	0		
AB3	14	0.98911	0.99055	0.99204	0.99241	0.99089	0.98458	0.99383	0.9871	0.98712	0.9937	0.70451	0.71878	0.50463	0	
AB2	15	0.98882	0.98992	0.99134	0.99193	0.99046	0.98446	0.99328	0.98691	0.98686	0.99304	0.69994	0.716	0.50636	0.00561(NS)	0
AB	16	0.98871	0.98972	0.99116	0.9918	0.99033	0.98434	0.99315	0.98681	0.98672	0.99288	0.69899	0.71496	0.52457	0.05984(NS)	-0.03807(NS)
ABR	17	0.99339	0.99848	0.99951	0.99756	0.99629	0.98974	0.99899	0.99123	0.99264	1	0.83697	0.84885	0.79587	0.4	0.40435
PVL	18	0.99207	0.99602	0.99719	0.99595	0.99459	0.9881	0.99737	0.98992	0.9909	0.99803	0.84318	0.85108	0.90728	0.87785	0.86855
RsPt	19	0.98961	0.99156	0.99299	0.993	0.99149	0.98506	0.99444	0.98747	0.98768	0.99446	0.80731	0.81504	0.86557	0.8427	0.83629
FR1	20	0.99191	0.99568	0.99687	0.99571	0.99434	0.98786	0.99714	0.98972	0.99064	0.99774	0.85305	0.86137	0.90765	0.87817	0.87088
FR2	21	0.98371	0.98064	0.98268	0.98578	0.98392	0.97785	0.9872	0.98168	0.97996	0.98563	0.66376	0.6745	0.71096	0.70122	0.7008
CCR1	22	0.99141	0.99443	0.99567	0.99451	0.99303	0.98635	0.99601	0.98865	0.98931	0.99644	0.98902	0.98931	0.99282	0.99009	0.98954
CCR2	23	0.99096	0.99355	0.99483	0.99393	0.99244	0.98586	0.99542	0.98827	0.98875	0.99569	0.98838	0.98867	0.99215	0.98944	0.98894
LH	24	0.99133	0.99426	0.99551	0.99448	0.99302	0.98647	0.99596	0.98875	0.98937	0.99635	0.98898	0.98926	0.99275	0.99004	0.9895
NatBr1	25	0.99368	0.99854	0.99953	0.99749	0.99618	0.98952	0.99896	0.99116	0.99257	1	0.99259	0.99287	0.9963	0.99364	0.99292
NatBr2	26	0.99292	0.99712	0.9982	0.9965	0.99515	0.98855	0.99797	0.9904	0.99153	0.99879	0.99141	0.99169	0.99513	0.99245	0.9918
SC3	27	0.99073	0.99317	0.99448	0.99361	0.99209	0.98544	0.99512	0.98793	0.98835	0.99534	0.98798	0.98827	0.99179	0.98905	0.98856
SC4	28	0.99202	0.99547	0.99665	0.99534	0.99393	0.98736	0.99681	0.98946	0.99029	0.99739	0.99004	0.99032	0.99377	0.99109	0.9905
SC5	29	0.99224	0.99597	0.99712	0.99561	0.99418	0.98747	0.99711	0.98953	0.99048	0.99778	0.99041	0.9907	0.99416	0.99147	0.99086
SCN	30	0.9908	0.99269	0.9937	0.99311	0.99186	0.98645	0.99434	0.98838	0.98883	0.99438	0.98867	0.98889	0.99167	0.98951	0.98909
BH	31	0.99318	0.99763	0.99868	0.99685	0.99551	0.98888	0.99832	0.99066	0.99189	0.99923	0.99183	0.99211	0.99555	0.99288	0.9922
SC2	32	0.99222	0.99779	0.99931	0.99668	0.99488	0.98617	0.99862	0.98902	0.99004	1	0.98963	0.99002	0.99482	0.99109	0.99029

		16	17	18	19	20	21	22	23	24	25	26	27	28	29	30	31
		AB	ABR	PVL	RsPt	FR1	FR2	CCR1	CCR2	LH	NatBr1	NatBr2	SC3	SC4	SC5	SCN	BH
AB	16	0															
ABR	17	0.44511	0														
PVL	18	0.86841	0.97046	0													
RsPt	19	0.83666	0.92571	0.46465	0												
FR1	20	0.86932	0.96748	0.92537	0.8733	0											
FR2	21	0.70115	0.78882	0.60544	0.57581	0.34444	0										
CCR1	22	0.98939	0.99656	0.99449	0.99077	0.99416	0.98168	0									
CCR2	23	0.98879	0.99584	0.99379	0.99011	0.99345	0.98111	0.57469	0								
LH	24	0.98935	0.99645	0.9944	0.99072	0.99407	0.98171	0.6859	0.49691	0							
NatBr1	25	0.99277	1	0.998	0.99438	0.9977	0.9854	0.875	0.7963	0.44444(NS)	0						
NatBr2	26	0.99165	0.99882	0.99681	0.99318	0.9965	0.9842	0.83914	0.75381	0.38272(NS)	0.07407(NS)	0					
SC3	27	0.98841	0.99551	0.99344	0.98972	0.9931	0.98064	0.00836(NS)	0.48675	0.6457	0.84641	0.81377	0				
SC4	28	0.99036	0.99746	0.99543	0.99178	0.99511	0.98282	0.77707	0.66287	0.2037(NS)	0(NS)	0.02469(NS)	0.74778	0			
SC5	29	0.99071	0.99787	0.99584	0.99217	0.99552	0.98316	0.72689	0.59402	0.71014	0.91803	0.87847	0.58483(NS)	0.80144	0		
SCN	30	0.98898	0.99457	0.99293	0.98999	0.99265	0.98288	-0.0557(NS)	0.53008	0.63184	0.80426	0.77911	-0.00758(NS)	0.72103	0.64419	0	
BH	31	0.99205	0.99925	0.99724	0.99361	0.99693	0.98462	0.8433	0.75096	0.32593(NS)	0.11111(NS)	0.08889(NS)	0.81481	-0.02339(NS)	0.88512	0.77651	0
SC2	32	0.99008	1	0.9972	0.99213	0.99678	0.97961	0.60742	0.69718	0.79866	1	0.94786	0.46224(NS)	0.88074	0.85692	0.51026	0.96406
