# Supplementary material for: Investigations of fine-scale phylogeography in Tigriopus californicus reveal historical patterns of population divergence
Source: BMC Evol Biol. 2009 Jun 23;9:139. doi: 10.1186/1471-2148-9-139 (PMC2708153; doi:10.1186/1471-2148-9-139)
Supplement: Additional file 7 — Table S5. Measures of the departures of the frequency spectra within regions and sites from neutral expectations. [file 1471-2148-9-139-S7.pdf]

Supplemental Table S5 Measures of the departures of the frequency spectra within regions and sites from neutral expectations. Tajami's D was calculated for all sites and for only sites with non-synonymous changes for CYTB.

**CYT B** Region	Population	Tajima's D all sites	p-value	Tajima's D non-syn. sites	p-value	Fu&Li D unrooted	p value	Fu&Li D rooted	p value	Fu&Li F unrooted	p value	Fu&Li F rooted	p value	Fay/Wu H	p value (no recombination)
SCruz	NB1	no polymorphism		no polymorphism											
	NB2	-0.69	n.s.	-1.11	n.s.	-0.28	n.s.	-0.41	n.s.	-0.42	n.s.	-0.56	n.s.	0.44	n.s.
	BH	0.01	n.s.	no polymorphism		0.8	n.s.	0.73	n.s.	0.68	n.s.	0.67	n.s.	-1.07	n.s.
	SCN	-0.57	n.s.	-0.17	n.s.	1.36	p<0.05	1.47	n.s.	0.96	n.s.	1.06	n.s.	-1.41	n.s.
	SC2	no polymorphism		no polymorphism											
	SC3	-0.06	n.s.	0.01	n.s.	0.77	n.s.	0.76	n.s.	0.64	n.s.	0.65	n.s.	-0.71	n.s.
	SC4	-1.79	p<0.05	-1.56	0.05<p<0.10	-2.08	p<0.05	0.08	n.s.	-2.26	p<0.05	-0.48	n.s.	-6.04	p=0.004
	SC5	-1.74	p<0.05	no polymorphism		-2.01	0.05<p<0.10	-0.18	n.s.	-2.18	0.05<p<0.10	-0.68	n.s.	-4.44	p=0.003
	LH	2.06	p<0.05	-1.84	0.05<p<0.10	1.15	n.s.	1.15	n.s.	1.53	n.s.	1.61	n.s.	0	n.s.
	CCR1	-1.88	p<0.05	-1.40	n.s.	-2.18	p<0.05	-1.73	n.s.	-2.36	p<0.05	-2.13	0.05<p<0.10	-2.13	n.s.
	CCR2	-1.68	0.05<p<0.10	-1.56	0.05<p<0.10	-1.81	0.05<p<0.10	-1.91	0.05<p<0.10	-2	0.05<p<0.10	-2.22	0.05<p<0.10	-1.42	n.s.
	total	1.17	n.s.	0.71	n.s.	-0.07	n.s.	-0.08	n.s.	0.46	n.s.	0.46	n.s.	1.11	n.s.
PVerde	FR1	-0.9	n.s.	-1.09	n.s.	-1.13	n.s.	1.27	n.s.	-1.21	n.s.	0.85	n.s.	-5.42	p=0.003
	FR2	1.3	n.s.	-0.82	n.s.	1.26	n.s.	1.34	n.s.	1.43	n.s.	1.57	n.s.	-1.07	n.s.
	RsPt	0.8	n.s.	no polymorphism		-1	n.s.	0.76	n.s.	-1.06	n.s.	0.6	n.s.	-4.89	p=0.02
	PVL	-0.43	n.s.	1.40	n.s.	-0.8	n.s.	0.74	n.s.	-0.8	n.s.	0.36	n.s.	-1.6	n.s.
	ABR	no polymorphism		no polymorphism											
	AB1	0.22	n.s.	-0.10	n.s.	0.58	n.s.	0.96	n.s.	0.55	n.s.	1.06	n.s.	1.31	n.s.
	AB2	-0.98	n.s.	-0.25	n.s.	-0.48	n.s.	-0.54	n.s.	-0.69	n.s.	-0.78	n.s.	-0.44	n.s.
	AB3	-2	p<0.01	-1.56	0.05<p<0.10	-2.35	p<0.02	-1.89	p<0.05	-2.55	p<0.02	-2.31	p<0.05	-3.2	p=0.05
	IP	-1.64	n.s.	-1.11	n.s.	-1.73	n.s.	-0.31	n.s.	-1.92	n.s.	-0.77	n.s.	-4	p=0.02
	RP1	-0.46	n.s.	0.20	n.s.	0.11	n.s.	1	n.s.	-0.04	n.s.	0.84	n.s.	-2.76	p=0.07
	RP2	0.02	n.s.	-1.51	n.s.	-0.33	n.s.	-1.02	n.s.	-0.28	n.s.	-1.2	n.s.	0.71	n.s.
	total	-1.08	n.s.	-1.77	0.05<p<0.10	-2.26	0.05<p<0.10	-0.11	n.s.	-2.11	0.05<p<0.10	-0.57	n.s.	-40.3	p<0.001
LJolla	SIO	-0.96	n.s.	-0.19	n.s.	-0.45	n.s.	-0.54	n.s.	-0.66	n.s.	-0.75	n.s.	0.35	n.s.
	LJP1	-0.66	n.s.	-0.37	n.s.	-0.05	n.s.	0.05	n.s.	-0.27	n.s.	-0.15	n.s.	-2.85	p=0.08
	LJP2	-0.94	n.s.	-0.35	n.s.	-0.07	n.s.	1.16	n.s.	-0.23	n.s.	0.71	n.s.	-7.02	p=0.01
	LJP3	no polymorphism		no polymorphism											
	LJS	-1.73	n.s.	-1.16	n.s.	-1.88	n.s.	-1.17	n.s.	-2.1	0.05<p<0.10	-1.53	n.s.	-1.09	n.s.
	NAUT	-2.09	p<0.01	no polymorphism		-2.66	p<0.02	-2.18	0.05<p<0.10	-2.86	p<0.02	-2.57	p<0.05	-2.39	p=0.05
	BR	0.33	n.s.	-1.16	n.s.	0.74	n.s.	0.67	n.s.	0.72	n.s.	0.69	n.s.	-3.47	p=0.07
	total	-0.87	n.s.	-1.48	n.s.	-1.62	n.s.	-1.33	n.s.	-1.58	n.s.	-1.37	n.s.	-8.9	p=0.03
PLoma	SD	-1.46	n.s.	no polymorphism		-2.36	0.05<p<0.10	-2.39	p<0.05	-2.44	0.05<p<0.10	-2.53	p<0.05	-1.21	n.s.
	SCL	-1.35	n.s.	no polymorphism		-1.43	n.s.	-0.8	n.s.	-1.52	n.s.	-1.09	n.s.	-0.95	n.s.
	LS	-1.05	n.s.	no polymorphism		-1.12	n.s.	-1.26	n.s.	-1.2	n.s.	-1.41	n.s.	0.21	n.s.
	total	-1.39	n.s.	no polymorphism		-3.15	p<0.02	-3.13	p<0.02	-3.04	p<0.02	-3.05	p<0.02	-0.88	n.s.

**RISP** Outcrop	Pop	Tajima's D	p-value	Fu&Li D unrooted	p value	Fu&Li D rooted	p value	Fu&Li F unrooted	p value	Fu&Li F rooted	p value	Fay/Wu H	p value/recomination value used
Palos Verde	RP1	-1.03	n.s.	-0.92	n.s.	-1.26	n.s.	-1.04	n.s.	-1.43	n.s.	-0.21	n.s.
	IP	-0.86	n.s.	-0.61	n.s.	-0.75	n.s.	-0.74	n.s.	-0.89	n.s.	1.71	n.s.
	AB1	0.34	n.s.	0.75	n.s.	0.17	n.s.	0.72	n.s.	0.1	n.s.	-1.5	n.s.
	PVL	1.2	n.s.	1.56	p<0.02	1.79	p<0.05	1.64	p<0.05	1.99	p<0.05	-3.14	n.s.
	total	0.15	n.s.	0.32	n.s.	0.16	n.s.	0.31	n.s.	0.12	n.s.	-4.19	p=0.052/R=10
Point Loma	SD	-0.25	n.s.	-0.13	n.s.	1.14	n.s.	-0.17	n.s.	0.98	n.s.	-4.53	p=0.032/R=0
La Jolla	LJP1	-0.89	n.s.	-1.15	n.s.	-0.24	n.s.	-1.21	n.s.	-0.48	n.s.	-5.36	p=0.04/R=5
	LJS	0.07	n.s.	-0.03	n.s.	-0.43	n.s.	-0.009	n.s.	-0.39	n.s.	-0.29	n.s.
	BR	-0.71	n.s.	-0.42	n.s.	0.69	n.s.	-0.55	n.s.	0.49	n.s.	-5.24	p=0.05/R=1
	total	-0.68	n.s.	-1.53	n.s.	-1.52	n.s.	-1.48	n.s.	-1.5	n.s.	-2.46	n.s.
Santa Cruz	SCN	-1.6	0.05<p<0.10	-1.73	0.05<p<0.10	-1.54	n.s.	-1.88	0.05<p<0.10	-1.84	n.s.	-1.54	n.s.
	LH	no polymorphism											
