# Supplementary material for: Investigations of fine-scale phylogeography in Tigriopus californicus reveal historical patterns of population divergence
Source: BMC Evol Biol. 2009 Jun 23;9:139. doi: 10.1186/1471-2148-9-139 (PMC2708153; doi:10.1186/1471-2148-9-139)
Supplement: Additional file 8 — Table S6. FST values for RISP from Arlequin based on pairwise comparisons of populations (pairwise sequence divergence). [file 1471-2148-9-139-S8.pdf]

Supplemental Table S6. Fst values for RISP from arlequin based on pairwise comparisons of populations (pairwise sequence divergence). Values that were not significant based on permutation analyses are indicated by (NS). Significance is based on α=0.05 in a Sequential Bonferroni procedure.

		1	2	3	4	5	6	7	8	9	10	11
		RP1	AB	IP	PVL	LH	SCN	LJS	LJP	BR	PES	SD
1	RP1	0										
2	AB	0.75429	0									
3	IP	0.27619	0.56562	0								
4	PVL	0.53356	0.41714	0.35616	0							
5	LH	0.99709	0.9952	0.99394	0.98755	0						
6	SCN	0.99522	0.99361	0.99252	0.98706	-0.02967 (NS)	0					
7	LJS	0.95093	0.9485	0.94659	0.93843	0.9532	0.95671	0				
8	LJP	0.97223	0.96981	0.96811	0.9601	0.97347	0.97416	0.06357	0			
9	BR	0.97566	0.97347	0.97203	0.96491	0.97707	0.97721	0.12096	0.23269	0		
10	PES	0.945	0.94243	0.94051	0.93206	0.77278	0.80061	0.87616	0.90152	0.91641	0	
11	SD	0.9911	0.98823	0.98617	0.9763	0.99404	0.9913	0.64197	0.76682	0.79607	0.90822	0
