# Supplementary material for: Investigations of fine-scale phylogeography in Tigriopus californicus reveal historical patterns of population divergence
Source: BMC Evol Biol. 2009 Jun 23;9:139. doi: 10.1186/1471-2148-9-139 (PMC2708153; doi:10.1186/1471-2148-9-139)
Supplement: Additional file 9 — Table S7. RISP nucleotide and indel variation found in Santa Cruz region and PES for T. californicus. [file 1471-2148-9-139-S9.pdf]

Supplemental Table S7. *RISP* nucleotide and indel variation found in Santa Cruz region and PES for *T. californicus.* Site numbers correspond to supplemental align positions. Shaded changes indicate derived allele determined by sequences found in LJ and PV regions (some ambiguous sites are indicated by a ?). Positions with numbers indicate presence or absence of indel.

		137	149	157	159	161	217	276	283	304	362	405	412	456	470	479	487	518	591	611	683	726	731	753	759	770
LH	m1-A	G	C	T	A	1	1	C	T	A	C	C	1	C	A	G	A	A	T	1	C	G	1	T	C	T
	f6-A	G	C	T	A	1	1	C	T	A	C	C	1	C	A	G	A	A	T	1	C	G	1	T	C	T
	f8-A	G	C	T	A	1	1	C	T	A	C	C	1	C	A	G	A	A	T	1	C	G	1	T	C	T
	m1-B	G	C	T	A	1	1	C	T	A	C	C	1	C	A	G	A	A	T	1	C	G	1	T	C	T
	f6-B	G	C	T	A	1	1	C	T	A	C	C	1	C	A	G	A	A	T	1	C	G	1	T	C	T
	f8-B	G	C	T	A	1	1	C	T	A	C	C	1	C	A	G	A	A	T	1	C	G	1	T	C	T
SCN	h-1	G	C	T	A	1	1	C	T	A	C	C	1	C	A	G	A	A	T	1	C	G	1	T	C	T
	h-2	G	C	T	A	1	1	C	T	A	C	C	1	C	A	G	A	A	T	1	C	G	1	T	C	T
	m3-A	G	C	T	A	1	1	C	T	A	C	C	1	C	A	G	A	A	T	1	C	G	1	T	C	T
	m5-A	G	C	T	A	1	1	C	T	A	C	C	1	C	A	G	A	A	T	1	C	G	1	T	C	T
	m7-A	G	C	T	A	1	1	C	T	A	C	C	1	C	A	G	A	A	T	1	C	G	1	T	C	T
	m5-B	G	C	T	A	1	1	C	T	A	C	C	1	C	A	G	A	A	T	1	C	G	1	T	C	T
	m7-B	G	C	T	A	1	1	C	T	A	C	C	1	C	A	G	A	A	T	1	C	G	1	T	C	T
	m3-B	G	C	T	A	1	1	C	T	A	C	C	1	C	A	G	A	A	T	1	C	G	1	T	C	T
PES	m9-A	T	G	G	T	2	2	T	G	G	A	G	2	G	T	T	T	G	C	2	G	C	2	G	T	G
	m9-B	G	C	T	A	1	1	C	T	A	A	G	2	C	A	G	A	A	T	1	C	G	1	T	C	T
	m10-A	T	G	G	T	2	2	T	G	G	A	G	2	G	T	T	T	G	C	2	G	C	2	G	T	G
	m10-B	T	G	G	T	2	2	T	G	G	A	G	2	G	T	T	T	G	C	2	G	C	2	G	T	G
PV/LJ		T	C	T	A	1	2	C	G	G	C	C	1	C	?	?	?	G	?	?	C	C	1	T	T	T

		811	814	853	911	919	946	952	984	986	1031	1044	1087	1099	1129	1159	1162	1248	1574	1590	1593	1597	1620	1795	1843	hap#
LH	m1-A	G	C	G	1	A	G	T	C	G	G	C	T	C	C	A	T	G	T	T	1	T	C	G	A	1
	f6-A	G	C	G	2	A	G	T	C	G	G	C	T	C	C	A	T	G	T	T	1	T	C	G	A	2
	f8-A	G	C	G	1	A	G	T	C	G	G	C	T	C	C	A	T	G	T	T	1	T	C	G	A	1
	m1-B	G	C	G	1	A	G	T	C	G	G	C	T	C	C	A	T	G	T	T	1	T	C	G	A	1
	f6-B	G	C	G	1	A	G	T	C	G	G	C	T	C	C	A	T	G	T	T	1	T	C	G	A	1
	f8-B	G	C	G	1	A	G	T	C	G	G	C	T	C	C	A	T	G	T	T	1	T	C	G	A	1
SCN	h-1	G	C	G	1	A	G	T	C	G	G	C	T	C	C	A	T	G	T	T	1	T	C	G	A	1
	h-2	G	C	G	1	A	G	T	G	G	G	C	T	C	C	A	T	G	T	C	2	G	C	G	G	3
	m3-A	G	C	G	1	A	G	T	C	G	G	C	T	C	C	A	T	G	T	T	1	T	C	G	A	1
	m5-A	G	C	G	1	A	G	T	C	G	G	C	T	C	C	A	T	G	T	T	1	T	C	G	A	1
	m7-A	G	C	G	1	A	G	T	C	G	G	C	T	C	C	A	T	G	T	T	1	T	C	G	A	1
	m5-B	G	C	G	1	A	G	T	C	G	G	C	T	C	C	A	T	G	T	T	1	T	C	G	A	1
	m7-B	G	C	G	1	A	G	T	C	G	G	C	T	C	C	A	T	G	T	T	1	T	C	T	A	4
	m3-B	G	C	G	1	A	G	T	C	G	G	C	T	C	C	A	T	G	T	T	1	T	C	G	A	1
PES	m9-A	T	A	C	1	G	A	C	C	T	A	C	A	G	A	G	C	A	G	T	1	T	T	G	G	5
	m9-B	G	C	G	1	G	G	T	C	G	A	C	A	G	A	G	C	A	G	T	1	T	T	G	G	6
	m10-A	T	A	C	1	G	A	C	C	T	A	A	A	G	A	G	C	A	G	T	1	T	T	G	G	7
	m10-B	T	A	C	1	G	A	C	C	T	A	C	G	G	A	G	C	A	G	T	1	T	T	G	G	8
PV/LJ		T	A	G	1	G	G	T	C	T	G	A	?	?	A	A	C	G	G	T	1	T	C	G	G	
