# Supplementary material for: Investigations of fine-scale phylogeography in Tigriopus californicus reveal historical patterns of population divergence
Source: BMC Evol Biol. 2009 Jun 23;9:139. doi: 10.1186/1471-2148-9-139 (PMC2708153; doi:10.1186/1471-2148-9-139)
Supplement: Additional file 10 — Table S8. RISP nucleotide and indel variation found in La Jolla region for T. californicus. [file 1471-2148-9-139-S10.pdf]

Supplemental Table S8 *RISP* nucleotide and indel variation found in La Jolla region for*T. californicus.* Site numbers correspond to supplemental align positions. Shaded changes are derived changes as indicated by *RISP* sequence at that position found in SD (consensus sequence from SD RISP listed on last line). Numbers indicate presence or absence of an insertion or deletion.

		95	119	193	265	293	400	401	418	426	445	464	489	496	525	537	538	574	681	699	736	748	754	762	781	803	804	813	814	823	884	920	936	1087	1112	1184	1504	1554	1555	1571	1575	1828	Hap. #
LJP	m1-A	A	G	C	T	A	A	A	T	A	G	C	1	1	1	A	A	G	1	T	C	A	C	C	1	T	A	C	A	1	A	T	T	A	G	1	G	G	G	G	G	C	1
	m7-A	A	G	C	T	A	A	A	T	A	G	C	1	1	1	A	A	G	1	T	T	A	C	C	1	T	A	C	A	1	A	T	T	T	G	2	G	G	G	G	G	C	2
	m2-A	A	G	C	G	A	A	A	T	A	G	C	1	1	1	A	A	G	1	T	T	A	C	T	1	T	A	C	A	1	A	G	T	T	G	2	G	G	G	G	G	C	3
	m6-B	A	G	C	T	A	A	C	T	A	G	C	2	2	2	A	A	G	1	T	T	A	A	C	1	T	A	C	A	1	A	T	T	A	G	2	G	G	G	G	G	C	4
	m7-B	A	G	T	G	A	A	A	T	A	G	C	1	1	1	A	T	G	1	T	T	A	C	C	1	T	A	C	A	1	A	G	T	T	G	2	G	G	G	G	G	C	5
	m2-B	A	G	C	G	A	A	A	G	G	A	C	2	2	1	A	A	G	1	T	T	A	C	C	1	T	A	C	G	2	A	G	T	T	G	2	G	G	G	G	G	C	6
	m1-B	A	G	C	T	A	A	A	T	A	G	C	1	1	1	A	A	G	1	T	C	A	C	C	1	T	A	C	A	1	G	T	T	A	G	1	G	G	G	G	G	C	7
	m6-A	G	A	C	T	A	A	A	T	A	G	C	1	1	1	A	A	G	1	T	C	A	C	C	1	T	A	C	A	1	A	G	T	T	A	1	A	G	G	G	G	C	8
LJS	m1-B	A	G	C	T	A	C	C	G	A	G	C	1	1	1	T	A	G	1	T	C	A	C	C	1	A	T	C	G	2	A	G	T	A	G	1	G	T	T	G	G	C	9
	m1-A	A	G	C	T	A	A	C	T	A	G	C	1	1	1	A	A	G	1	T	C	A	C	C	1	T	T	C	G	2	A	G	T	A	G	1	G	G	T	G	G	C	10
	m2-A	A	G	C	G	A	A	A	G	A	G	C	1	1	1	A	A	G	1	T	T	A	C	C	1	T	T	C	G	2	A	T	T	T	G	2	G	G	G	G	G	C	11
	m2-B	G	G	C	T	A	A	A	T	A	G	C	1	1	1	A	A	G	1	A	C	A	C	C	1	T	A	C	A	1	A	T	T	T	G	2	G	G	G	A	G	C	12
	f9-B	A	G	C	T	A	A	A	T	A	G	C	1	1	1	A	A	G	2	----	C	A	C	C	1	T	A	C	G	2	A	G	T	A	G	1	G	G	G	A	G	C	13
	f9-A	A	G	C	T	A	A	A	T	A	G	C	1	1	1	A	T	C	1	T	C	A	C	C	1	T	T	C	G	2	A	G	T	A	G	2	G	G	G	A	G	C	14
	f10-B	A	G	C	T	A	A	A	T	A	G	C	1	1	1	A	T	C	1	T	C	A	C	C	1	T	A	C	A	1	A	T	T	T	G	1	G	G	T	G	G	T	15
	f10-A	A	G	C	T	A	A	A	T	A	G	C	1	1	1	A	T	G	1	T	T	C	A	C	2	T	A	C	A	1	A	G	T	T	G	2	G	G	G	A	G	C	16
BR	f13-B	A	G	C	T	A	A	C	G	G	A	A	2	2	1	A	T	G	1	T	T	A	C	C	1	T	T	C	G	2	A	G	T	A	G	1	G	G	G	G	G	C	17
	m5-A	A	G	C	G	T	A	A	T	A	G	C	1	1	1	A	A	G	1	T	T	A	C	C	1	T	A	T	A	1	A	T	A	T	G	2	G	G	G	G	A	C	18
	m5-B	A	G	C	G	A	A	A	T	G	A	C	2	2	1	A	A	G	1	T	C	A	C	C	1	T	T	C	G	2	A	G	T	A	G	1	G	G	G	G	A	C	19
	m8-B	A	G	C	G	A	A	C	G	A	G	C	1	1	1	A	T	G	1	A	C	A	C	C	1	T	T	C	G	2	A	G	T	A	G	2	G	G	G	G	G	C	20
	f13-A	A	G	C	G	A	A	C	T	A	G	C	1	1	1	A	T	G	1	A	C	A	C	C	1	T	T	C	G	2	A	G	T	A	G	2	G	G	G	G	G	C	21
	m6-B	A	G	C	G	A	A	C	T	A	G	C	1	1	1	A	T	G	1	A	C	A	C	C	1	T	T	C	G	2	A	G	T	A	G	2	G	G	G	G	G	C	21
	m7-A	A	G	C	G	A	A	C	T	A	G	C	1	1	1	A	T	G	1	A	C	A	C	C	1	T	T	C	G	2	A	G	T	A	G	2	G	G	G	G	G	C	21
	m7-B	A	G	C	G	A	A	C	T	A	G	C	1	1	1	A	T	G	1	A	C	A	C	C	1	T	T	C	G	2	A	G	T	A	G	2	G	G	G	G	G	C	21
	m6-A	A	G	C	G	A	A	C	G	A	G	C	1	1	1	A	T	G	1	A	C	A	C	C	1	T	T	C	G	2	A	G	T	T	G	2	G	G	G	G	G	C	22
	m8-A	A	G	C	G	A	A	C	G	A	G	C	1	1	1	A	T	G	1	A	C	A	C	C	1	T	T	C	G	2	A	G	T	T	G	2	G	G	G	G	G	C	22
SD	cons.	A	A	C	T	C	A	C	G	A	A	C	1	1	1	A	T	G	1	T	T	A	C	C	2	T	A	C	A	2	A	G	T	T	G	1	G	G	G	G	G	C	
