# Supplementary material for: Investigations of fine-scale phylogeography in Tigriopus californicus reveal historical patterns of population divergence
Source: BMC Evol Biol. 2009 Jun 23;9:139. doi: 10.1186/1471-2148-9-139 (PMC2708153; doi:10.1186/1471-2148-9-139)
Supplement: Additional file 11 — Table S9. RISP nucleotide and indel variation found in Palos Verdes region for T. californicus. [file 1471-2148-9-139-S11.pdf]

Supplemental Table S9. *RISP* nucleotide and indel variation found in Palos Verdes region for *T. californicus.* Site numbers correspond to supplemental align positions. Shaded changes are derived changes as indicated by *RISP* sequence at that position found in SD/SCN (listed on last line). Numbers indicate presence or absence of deletion.

		53	54	70	72	95	163	166	168	254	265	293	305	329	351	424	425	426	427	430	829	852	954	1246	1525	1540	1628	1711	1819	Hap. #
RP1	m8-B	C	C	C	G	A	T	1	A	T	T	C	1	1	C	C	T	G	T	G	1	G	A	G	G	C	T	C	C	1
	m7-A	C	C	C	G	A	T	1	A	T	T	C	1	1	C	C	T	G	T	G	1	G	A	G	G	C	T	C	C	1
	m1-A	C	C	C	G	A	T	1	A	T	T	C	1	1	C	C	T	G	T	G	1	G	A	G	G	C	T	C	C	1
	m5-A	C	C	C	G	A	T	1	A	T	T	C	1	1	C	C	T	G	T	G	1	G	A	G	G	C	T	C	C	1
	m1-B	C	C	C	C	A	T	1	A	T	T	C	1	1	C	C	T	G	T	G	1	G	A	G	G	C	T	C	C	2
	m5-B	C	C	G	G	A	T	1	A	T	T	C	1	1	C	C	T	G	T	G	1	G	A	G	G	C	T	C	C	3
	m8-A	C	C	C	G	A	T	1	A	T	T	C	1	1	C	C	T	G	T	G	1	G	A	G	G	C	T	C	T	4
	m7-B	C	C	C	G	A	T	2	T	T	T	C	1	1	C	C	T	G	T	G	1	G	A	G	G	C	T	C	T	5
IP	m7-B	C	C	C	G	A	T	1	A	T	T	C	1	1	C	C	T	G	T	G	1	G	A	G	G	C	T	C	T	4
	f23-A	C	C	C	G	A	T	1	A	T	T	C	1	2	C	C	T	G	T	G	1	G	A	G	G	C	T	C	T	6
	m7-A	C	C	C	G	A	T	1	A	T	T	C	1	2	C	C	T	G	T	G	1	G	A	G	G	C	T	C	T	6
	f3-B	C	C	C	G	A	T	1	A	T	T	C	1	2	C	C	T	G	T	G	1	G	A	G	G	C	T	C	T	6
	m8-B	C	C	C	G	A	T	1	A	T	T	C	1	2	C	C	T	G	T	G	1	G	A	A	G	C	T	C	T	7
	m8-A	C	C	C	G	A	G	1	A	T	T	C	1	2	C	C	T	G	T	G	1	G	A	G	G	C	T	C	T	8
	f3-A	C	T	C	G	G	T	1	A	T	T	C	2	1	T	C	T	G	T	G	1	G	A	G	G	C	T	C	T	9
	f23-B	C	T	C	G	G	T	1	A	C	G	C	2	1	T	C	T	G	T	G	2	G	A	G	G	C	T	G	T	10
AB	g-1	C	T	C	G	G	T	1	A	C	G	C	2	1	T	C	T	G	T	G	1	G	A	G	G	C	C	G	T	11
	g-2	C	T	C	G	G	T	1	A	C	G	T	2	1	T	C	T	G	T	G	1	G	A	G	G	C	C	G	T	12
	h-1	C	T	C	G	G	T	1	A	C	G	T	2	1	T	C	T	G	T	G	1	G	A	G	G	C	C	G	T	12
	h-2	C	T	C	G	G	T	1	A	C	G	T	2	1	T	C	T	G	T	G	1	G	A	G	G	C	C	G	T	12
	m1-A	T	T	C	G	G	T	1	A	T	T	C	1	2	C	C	T	G	T	G	1	G	A	G	G	C	C	G	T	13
	m2-A	C	T	C	G	G	T	1	A	T	T	C	1	2	C	C	T	G	T	G	1	G	A	G	G	C	C	G	T	14
	m1-B	C	T	C	G	G	T	1	A	C	G	T	2	1	T	C	T	G	T	G	1	G	A	G	G	C	C	G	T	12
	m2-B	C	T	C	G	G	T	1	A	C	G	T	2	1	T	C	T	G	T	G	1	G	A	G	G	C	C	G	T	12
PVL	f12-A	C	C	C	G	A	G	1	A	T	T	C	1	2	C	G	T	G	T	G	1	G	A	G	G	C	T	C	T	15
	f12-B	C	C	C	G	A	G	1	A	T	T	C	1	2	C	G	T	G	T	G	1	G	A	G	G	C	T	C	T	15
	f14-B	C	T	C	G	G	T	1	A	C	G	C	2	1	T	C	T	G	T	G	1	A	A	G	A	T	T	C	T	16
	f3-A	C	T	C	G	G	T	1	A	C	G	C	2	1	T	C	T	G	T	G	1	A	A	G	A	T	T	C	T	16
	f7-A	C	T	C	G	G	T	1	A	C	G	C	2	1	T	C	C	A	A	A	1	A	G	G	G	C	T	C	T	17
	f14-A	C	T	C	G	G	T	1	A	C	G	C	2	1	T	C	C	A	A	A	1	A	G	G	G	C	T	C	T	17
	F3-B	C	T	C	G	G	T	1	A	C	G	C	2	1	T	C	C	A	A	A	1	A	G	G	G	C	T	C	T	17
	f7-B	C	T	C	G	G	T	1	A	C	G	C	2	1	T	C	C	A	A	A	1	A	G	G	G	C	T	C	T	17
SD/SCN		C	C	C	G	A	T/A	1	A	-	A/T	C	2	2	C	C	T	G/A	A	G	1	A	A	G	A	C	C	C	T	
